# Optical recognition of the eggs of four Aedine mosquito species (*Aedes albopictus*, *Aedes geniculatus*, *Aedes japonicus*, and *Aedes koreicus*)

**DOI:** 10.1371/journal.pone.0293568

**Published:** 2023-11-01

**Authors:** Nikoleta Anicic, Klaus Steigmiller, Claude Renaux, Damiana Ravasi, Matteo Tanadini, Eleonora Flacio

**Affiliations:** 1 Institute of Microbiology, Department for Environment Constructions and Design, University of Applied Sciences and Arts of Southern Switzerland, Mendrisio, Switzerland; 2 Zurich Data Scientists GmbH, Zurich, Switzerland; Universitat Wien, AUSTRIA

## Abstract

The continuous expansion of exotic Aedine mosquito species potential vectors of pathogens into new areas is a public health concern. In continental Europe, the surveillance of these mosquitoes is hindered by the simultaneous presence of three main invasive species (i.e., *Aedes albopictus*, *Ae*. *japonicus*, and *Ae*. *koreicus*). Standard low-cost surveillance methods (i.e., the deployment of oviposition traps and count of eggs under stereoscopic microscope) fail to distinguish the eggs of the different species. Identification of eggs by molecular methods is costly and time consuming and prevents measuring the density of invasive species and detecting early new invaders. Here we tested whether certain species could be identified by the patterns on the exochorionic membrane of their eggs. In a first step, we examined Aedine eggs of the three mentioned invasive and one indigenous (i.e., *Ae*. *geniculatus*) species with a high-resolution stereomicroscope and we identified each egg by MALDI-TOF mass spectrometry. In a second step, we submitted images of the eggs to 60 entomology experts and non-experts and tested their ability to distinguish among the species after an initial short training. The results obtained were consistent. Participants did not encounter difficulties in determining *Ae*. *albopictus* and *Ae*. *geniculatus*, while they had more difficulties in distinguishing *Ae*. *japonicus* from *Ae*. *koreicus*. In general, the quality of the exochorion seemed to play a more important role than the expertise level of the rater. The feasibility to differentiate *Ae*. *albopictus* from the other two invasive species is a significant achievement, as this is currently the most problematic species at the level of public health in Europe. Due to the presence of multiple invasive species that might prevent the correct quantification of mosquito population densities using standard surveillance methods and due to *Ae*. *aegypti* threat, it is recommended to optically determine also other species.

## Introduction

Since the late 1990s, an increase of invasive mosquitoes of the genus *Aedes* has been observed throughout Europe [1–3]. Their arrival and expansion have been favoured mainly by human activities, such as tire and lucky bamboo trades, and climate change [4–6].

Invasive *Aedes* mosquitoes, in addition to being a nuisance for citizens, also represent a threat to public health as they can potentially carry and transmit arboviruses (e.g., dengue, chikungunya, and Zika viruses) [1, 3, 7] and filarioid parasites (e.g., *Dirofilaria* nematodes) [1, 3, 8–11]. *Aedes albopictus* (Skuse), the tiger mosquito, is the species that poses the greatest threat to public health as it can potentially transmit at least 22 arboviruses [1, 3]. During the last half century, it has spread from Southeast Asia to all the other continents, except Antarctica [1, 12–14]. Its first appearances in Europe were recorded in 1979 in Albania [15] and later in 1990 in Italy [16]. Since then, it has spread, mainly by passive transportation, to all countries around the Mediterranean and northwards [1, 12, 17–20]. In the last two decades, cases of autochthonous transmission of dengue and chikungunya viruses by *Ae*. *albopictus* have been reported in Italy [21], France [22, 23], Spain [24, 25] and Croatia [26]. In Switzerland, *Ae*. *albopictus* is well established in the southern regions of the country [27–29].

Two other invasive *Aedes* species present in Switzerland and other countries in central Europe are *Aedes japonicus* (Theobald), the East Asian bush mosquito, and *Ae*. *koreicus* (Edwards), the Korean bush mosquito. Both species have spread from eastern Asia. *Aedes japonicus* is more widely distributed in Germany, Austria, northeast Italy, Slovenia, north of Croatia, Hungary, north of Spain, and spots in the Netherlands, Belgium, Slovakia, and Romania [1, 19, 30, 31]. In Switzerland, it is present in all cantons [29]. *Aedes koreicus* is found in northeast Italy [32–35], Austria [36] and Hungary [37], and in spots in Belgium [38], Germany [39], Slovenia [40], and in Russia around the Black Sea [41], besides its place of origin [1, 19]. In Switzerland, it is found in Canton of Ticino and in Maloja and Bernina regions of Canton of Grisons and has sporadically appeared along the highway in other cantons, north of the Alps [29]. These two species pose a lower risk to public health than the tiger mosquito. Indeed, for both species, vector competence has been demonstrated mainly only in laboratory experiments. *Aedes japonicus* is competent for the West Nile virus [42, 43], Japanese encephalitis virus [44], La Crosse virus [45], Eastern equine encephalitis virus [46] and St. Louis encephalitis virus [47]. *Aedes koreicus* can potentially transmit the Japanese encephalitis virus and *Dirofilaria* [1, 3, 10].

The establishment of strong surveillance and control programs at local, national, and international levels plays a major role in containing the expansion of invasive mosquitoes and their related public health risk [48–52]. Proactive surveillance is essential within an early warning system for instance for the early discovery of invasive mosquito species populations, to prevent their local establishment and further spread [48].

Mosquito presence and their relative abundance are usually assessed by deployment of oviposition traps (i.e., ovitraps), which attract gravid females of container-breeding mosquitoes, such as *Aedes* species, in search of a spot to lay their eggs [50–54]. Surveillance systems with ovitraps, in contrast to other methods, such as adult traps, larval sampling and human landing collections, allow extensive territorial monitoring because larger areas can be monitored with a similar effort. In addition, the cost of the traps and the labour used for management is lower. Therefore, ovitraps are cost-effective for a rapid detection of species in sensitive areas.

However, the simultaneous presence of *Ae*. *albopictus*, *Ae*. *japonicus* and *Ae*. *koreicus* in the same areas constitutes a major drawback for this surveillance approach. This because the eggs of these three *Aedes* species are morphologically very similar. Only the eggs laid by the local species *Ae*. *geniculatus* (Olivier) are easily distinguishable from the other three [29, 55]. Since the eggs of the invasive *Aedes* species cannot be distinguished from each other, density data can be misleading. If the presence of eggs of more than one invasive species in an ovitrap is suspected, eggs can be hatched to enable the identification of larvae [50, 51]. However, this is a costly and time-consuming operation and hatching is frequently underestimated and delayed due to the presence of diapausing eggs and many other factors [55]. Molecular identification methods, such as DNA barcoding, can be applied as well, but are costly and also time consuming. The latest molecular technique, Matrix Assisted Laser Desorption/Ionization Time of Flight Mass Spectrometry (MALDI-TOF MS), is cheaper compared to DNA barcoding. But, even if it is widely used in clinical diagnostic, it is still time consuming, expensive when the entire ovitrap sample is analysed. With all these methodologies, only a small part of ovitraps with eggs can be analysed, which is not suitable for widespread monitoring, because some species may not be identified and there is no clear numerical relationship between eggs belonging to different species anyway.

On account of the above, a faster and affordable method is needed to differentiate eggs of different invasive species present in an ovitrap and their seasonal abundance. Morphological identification of Aedine eggs based on the observation of the exochorion (i.e., the outer layer of the chorion of the insect egg) structures might represent a solution. The exochorion of mosquito eggs has been previously considered [56–58]. Most studies, starting with Hinton and Service (1969) and Matsuo et al. (1974), focused on scanning electron microscopy (SEM), which allows to distinguish all the details present on the exochorion [59–61]. In general, researchers have taken eggs from both the field and laboratory colonies [59–68]. In addition, eggs are treated with silver prior SEM analyses, which prevents the identification of the same egg with different methods. Fewer studies have been conducted using cameras attached to stereomicroscopes. Most of these studies aimed at enabling the development of automatic egg counting systems [69, 70–77]. In 2009, Obenauer and colleagues used the imaging software Auto-Montage^TM^ to combine several photomicrographs focused on different levels in order to obtain a unique image that had everything in focus [78]. However, the analysis did not focus on differences present on the exochorion. Bova and colleagues (2014) compared images of morphological features of lab colonies of *Ae*. *aegypti* (Linnaeus), *Ae*. *albopictus*, *Ae*. *japonicus*, and *Ae*. *triseriatus* (Say) eggs taken by a video camera installed on a stereomicroscope (magnification of 64 times) and SEM [79, 80]. The difference found in the eggs was focused more on the colour, size of the egg and of chorionic cells, rather that the detailed pattern of the exochorion.

Here, we developed a technique for the optical identification of the eggs of three invasive (i.e., *Ae*. *albopictus*, *Ae*. *japonicus* and *Ae*. *koreicus*) and one local (*Ae*. *geniculatus*) Aedine species present in Switzerland, based on their exochorionic structure. During the initial phase, eggs collected in the field with ovitraps [27, 51] were analysed optically using a high-resolution stereomicroscope. The routine identification method MALDI-TOF MS was used to identify the species of the eggs [55]. The method was then validated by presenting images of the eggs of the four Aedine species to 60 observers (experts and non-experts) who had to select the correct species after a short training. We demonstrated that certain *Aedes* species are easily recognisable by their eggs. We believe that this technique could improve the surveillance of invasive *Aedes* mosquitoes.

## Materials and methods

### Sample collection

Mosquito eggs of *Ae*. *albopictus*, *Ae*. *geniculatus*, *Ae*. *japonicus* and *Ae*. *koreicus* were collected from the field with ovitraps. The ovitraps used consisted of a black plastic container with a capacity of 1.5 L (Ramona Ø13/H12, Luwasa® Interhydro AG, Allmendingen, Switzerland) and a wooden paddle (steamed beechwood, 200 x 25 x 5 mm) with the function of oviposition substrate. Ovitraps were deployed throughout Switzerland following the methodology described by Flacio et al. (2015) [28]. Several locations, based on the known distribution of the species of interest [27–29], were chosen to take into account the morphological variation potentially present in eggs obtained from different mosquito populations. Eggs of all four species were collected during monitoring conducted in Canton of Ticino and nationwide in summer 2020 and 2021 (Table 1). All data from ovitraps are stored in a national database managed by the Institute of Microbiology (SUPSI) and info fauna (http://www.infofauna.ch/ (accessed on 16 September 2023)).

**Table 1:** Number of eggs optically analysed in relation-the species and geographical location. The species was determined via MALDI-TOF.

Geographical location					
Location	*Ae*. *albopictus*	*Ae*. *geniculatus*	*Ae*. *japonicus*	*Ae*. *koreicus*	Total
Ticino	40	10	19	25	94
Grisons	12	16	24	50	102
Geneva	10	0	0	0	10
Vaud	0	4	0	0	4
Schaffhausen	0	2	0	0	2
Schwyz	0	0	14	0	14
Zurich	0	0	11	0	11
Total	62	32	68	75	237

### Optical and MALDI-TOF MS species analyses

For the optical analysis of the eggs, we used a Zeiss Axio Zoom V.16 high-resolution stereomicroscope with a Schott VisiLED Intense Brightfield Ring Light S80-25 (Schott AG, Mainz, Germany) at maximum light intensity, and ZEN 3.0 blue edition software (Carl Zeiss Microscopy GmbH, München, Germany). The analysis process was executed in two steps, each with different settings, with the help of a ZEISS technician.

In the first step, we acquired an image of one side of the wooden paddle at low resolution with a magnification of 7 times. This allowed us to obtain the position of a single egg or small group of eggs, called batch, on the wooden paddle. We were also considering batches, because we found that in most cases eggs were laid by a single female. To obtain the image, an initial autofocus and light adjustment were performed with the software controls. Afterwards, in order to have an image of the entire side of the paddle, the software did an automated merging of smaller images, called tiles. The number of tiles depends on the side of the wooden paddle analysed: the narrowest and the widest sides have 14 and 42 tiles each, respectively.

In the second step, we imported the merged image in the software and used it as a reference for egg location on the wooden paddle. For each egg or small batch of eggs analysed, the two limits of a high-resolution Z-stack image with a magnification of 112 times were set: the lower limit was set at the level of the paddle still in focus and the upper limit immediately after the egg began to be blurred. Within this range, the software generated a single image in which each slice was spaced 9 μm apart.

Each photographed egg was then identified at the species level with MALDI-TOF MS. To do this, the egg was retrieved from the wooden paddle with a scalpel and prepared for MALDI-TOF MS analysis following the protocol of Schaffner et al. [55]. The egg (or single egg from batches) was analysed using an AXIMA^TM^ Confidence machine (Shimadzu-Biotech Corp., Kyoto, Japan) that detects in a linear, positive mode using a laser frequency of 50 Hz and a mass range of 3’000 to 20’000 Da. The extraction delay time was 200 ns, and the acceleration voltage was 20 kV. Each ion spectrum was created using a minimum of 50 laser flashes. The obtained spectra were sent to Mabritec AG (Riehen, Switzerland) for species identification.

The origin of mosquito eggs from the field implies that the structure of the exochorion can be subjected to biotic (e.g., deposition of organic matter on the surface of the egg) and abiotic (e.g., weather conditions) factors. This has an impact on the quality of the egg structure, which must be considered when determining the species by optical means. Therefore, the quality of the exochorion was included in the analysis. We classified eggs in three quality levels. In eggs of ‘high quality’, the structure of the exochorion could be clearly observed throughout the entire length of the egg. In ‘medium quality’ eggs, only a few details of the exochorion could be seen. In ‘low quality’ eggs, the exochorionic structure was almost entirely damaged.

Compared to other studies [62, 66, 80], measures of length, width, and ratio of both were not considered in this project. As observed by Bova et al. (2016), lengths are not relevant if eggs were collected from the field. We have also observed that size of the eggs is not always relevant. S1 Fig illustrates an *Ae*. *albopictus* egg that we collected during our surveillance program.

### Inter-rater reliability tests

To validate the optical system, we assessed the effectiveness of the optical recognition by running inter-rater reliability tests. Since it was logistically not feasible for every participant to use the microscope individually, images taken by the same device were used. Internally, rather than seeing directly into the microscope, the same work was done on the images obtained from it. First, we created questionnaires to be submitted to the raters. Each participant (rater) received two images for each combination of the four species (*Ae*. *albopictus*, *Ae*. *geniculatus*, *Ae*. *japonicus*, and *Ae*. *koreicus*) and the three different qualities of images (high, medium, and low), which results in a total of 24 distinct images. This means that the questionnaires are balanced. For some combinations, many images were available. In these cases, we randomly selected some of the images and excluded others in order to satisfy the aforementioned rule. Each individual participant then received the questionnaires in form of a PDF file with the 24 images and corresponding check boxes under the images where they could select one of the four species as an option. Two inter-rater reliability test sessions were held. The procedure for creating the questionnaires was used independently for the two tests. The first test was held from May to September 2022. In this test, 30 questionnaires were submitted to 10 entomologists, considered as experts, and to 20 general biologists, considered as non-experts. Together with the questionnaire, each participant received by e-mail a 7-minute video presentation of the project. The presentation contained the main characteristics of each egg in order to distinguish them, instructions on how to complete the questionnaire and how to record the time invested in completing it; S2 and S3 Figs show an extract of the presentation for exochorion characteristics of each species.

The second test included participants of the training course of the 10^th^ European Mosquito Control Association (EMCA) workshop, held in Mendrisio (Switzerland) on 30^th^ November 2022. In this occasion, we distributed 25 questionnaires to entomologists (experts) and the remaining five to biologists (non-experts). The participants of the EMCA training course did not watch the video but were shown the same presentation used for the video. The five non-experts received the same documentation by e-mail as in the first test.

### Statistical analyses

Data obtained from questionnaire was converted in Excel tables and analysed by R version 4.2.2 [81] on Windows 10 for all calculations. The package *epiR* version 2.0.54 [82] was used for calculating the measures of diagnostic accuracy described in detail in the S1 Table (all analyses are detailed in S1 and S2 Texts). Each species’ present was analysed separately. Incorrect answers (with respect to the given image or species) were considered as negative results and correct answers as positive results for the calculation of diagnostic accuracy measures. Wilson 95% confidence intervals (95%CI) were used for proportions and Wald 95%CI on the log scale. Missing or multiple answers for a given image and rater were considered missing values and were removed from the analysis. The following pre-specified subgroup analyses were performed: expert level of the raters, exochorion qualities, and only for the second test, per rater. The results from the MALDI-TOF MS reference test are unambiguous.

## Results

### Image dataset of exochorion identified optically and with MALDI-TOF MS

We obtained 237 images of single eggs, or batches of eggs, identified at species levels with high-resolution stereomicroscope and by MALDI-TOF MS (Tables 1 and 2). In other to define differences in the exochorion, we considered exochorionic network, outer chorionic cells, and their central tubercule. Faull and Williams have illustrated these exochorionic structures in 2016 [62].

**Table 2:** Number of eggs optically analysed in relation-the species and the exochorion quality. The species was determined via MALDI-TOF.

Exochorion quality				
Species	high quality	medium quality	low quality	Total
*Ae*. *albopictus*	23	29	10	62
*Ae*. *geniculatus*	16	8	8	32
*Ae*. *japonicus*	48	26	9	83
*Ae*. *koreicus*	18	19	23	60
Total	105	82	50	237

**Fig 1:**
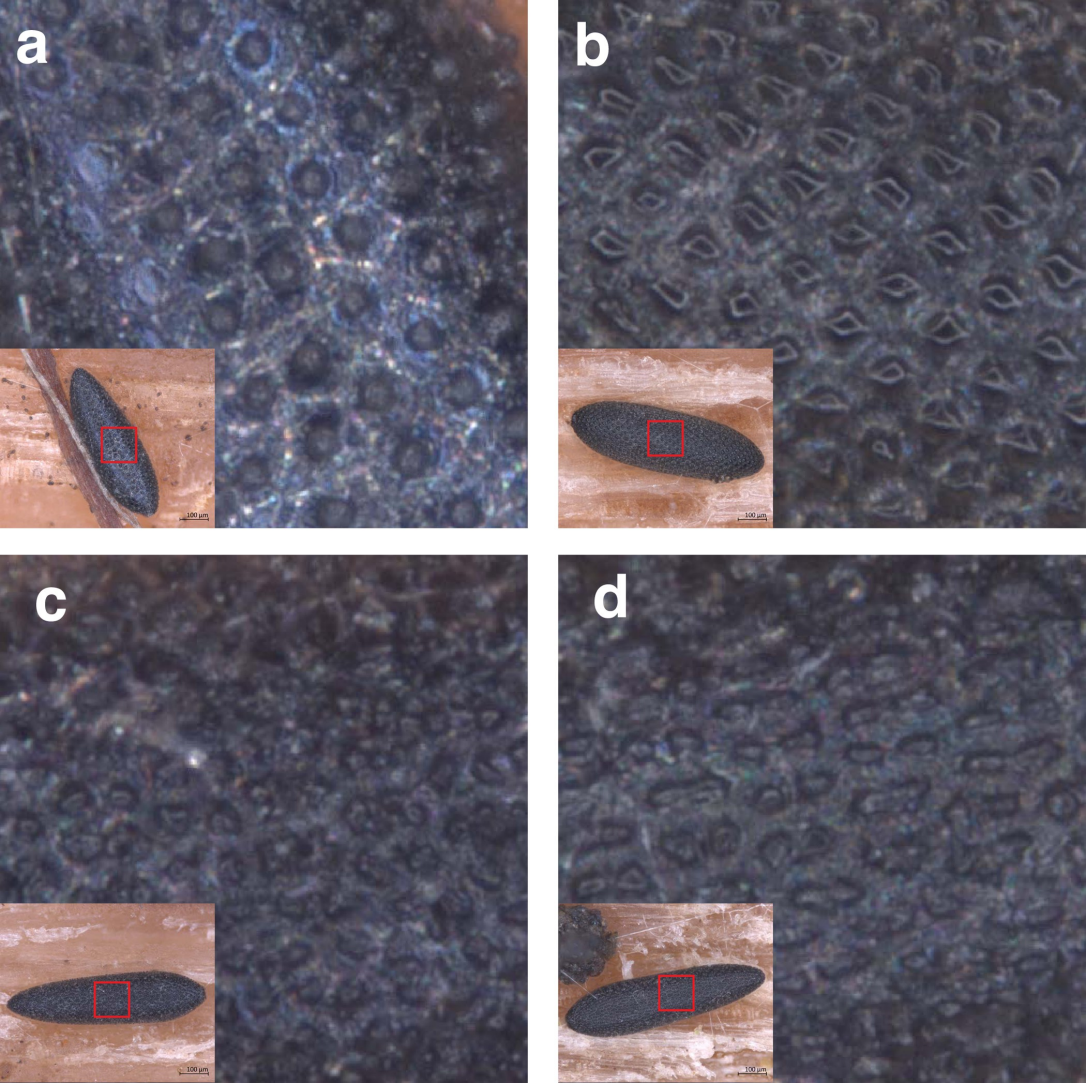
Patterns present on exochorion membrane. Egg of *Aedes albopictus* (a), *Aedes geniculatus* (b), *Aedes japonicus* (c), and *Aedes koreicus* (d).

In S2 and S3 Figs we show the main exochorionic structures that were also presented to participants of both tests. *Aedes albopictus* and *Ae*. *geniculatus* eggs present one large spherical and more or less rectangular central tubercle, respectively. In *Ae*. *albopictus*, the exochorionic network between the outer chorionic cells is large and creates space between them, while for *Ae*. *geniculatus* the network is small and creates less space between the chorionic cells (S2 Fig). When the exochorion of *Ae*. *japonicus* and *Ae*. *koreicus* eggs are compared, the outer chorionic cells consist of a small group of 2–4 round tubercles, which give an undefined texture. The exochorionic network between the outer chorionic cells is small and creates less space between them for *Ae*. *japonicus*, whereas for *Ae*. *koreicus* the outer chorionic cells are more spaced (S3 Fig). Figs 1 and S4 illustrate the exochorion structures of ‘high quality’ eggs of the four *Aedes* species, whereas ‘medium’ and ‘low qualities’ are shown in S5 and S6 Figs. Definitions are specified in S3 Text, and raw data for both tests is available in S1 and S2 Datasets.

### Test 1

The questionnaires of tests 1 and 2 were created using 188 images of identified eggs, out of the 237 images obtained. For the test 1, 14 of the 720 answers received were not considered valid due to missing values. The determination accuracy of *Ae*. *albopictus* and *Ae*. *geniculatus* was high, more than 92% of eggs were correctly identified. For *Ae*. *japonicus* and *Ae*. *koreicus*, less than 63% of eggs were correctly identified, the determination accuracy was low (Table 3).

**Table 3:** Test 1: number and percentage of eggs correctly identified by the raters. Percentages of each column sum to 100%.

MALDI-TOF MS determination				
Answer of the rater	*Ae*. *albopictus*	*Ae*. *geniculatus*	*Ae*. *japonicus*	*Ae*. *koreicus*
*Ae*. *albopictus*	167 (94.4%)	8 (4.5%)	17 (9.7%)	0 (0.0%)
*Ae*. *geniculatus*	4 (2.3%)	165 (92.7%)	25 (14.3%)	9 (5.1%)
*Ae*. *japonicus*	6 (3.4%)	2 (1.1%)	110 (62.9%)	78 (44.3%)
*Ae*. *koreicus*	0 (0.0%)	3 (1.7%)	23 (13.1%)	89 (50.6%)
Total	177 (100.0%)	178 (100.0%)	175 (100.0%)	176 (100.0%)

**Fig 2:**
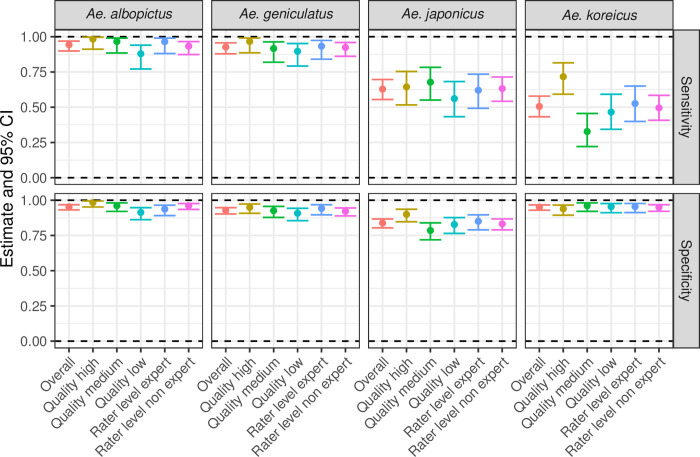
Test 1: Estimates (solid dots) and 95% confidence intervals (bars) for sensitivity and specificity for each group (overall, exochorion qualities, and levels of the rater) and each species.

Table 4 and Fig 2 illustrate sensitivity and specificity for all groups: overall species determination, the three quality levels of the exochorion and the two expertise levels of the raters. *Aedes albopictus* and *Ae*. *geniculatus* are easily distinguished and show a very high sensitivity (i.e., proportion of true positives), while it is still just acceptable for *Ae*. *japonicus* and *Ae*. *koreicus*. The estimates for specificity (i.e., proportion of true negatives) are very high for classifying three species, above 92%, but it is still just acceptable for *Ae*. *japonicus* (84%).

**Table 4:** Test 1: Estimates and 95% confidence intervals (95%CI) for sensitivity and specificity for each group (overall, exochorion qualities, and levels of the rater) and each species.

Estimates (95%CI)					
Analyses	Accuracy measures	*Ae*. *albopictus*	*Ae*. *geniculatus*	*Ae*. *japonicus*	*Ae*. *koreicus*
Overall	Sensitivity	94.4% (89.9–96.9)	92.7% (87.9–95.7)	62.9% (55.5–69.7)	50.6% (43.2–57.9)
	Specificity	95.3% (93.1–96.8)	92.8% (90.3–94.7)	83.8% (80.4–86.7)	95.1% (92.9–96.6)
High quality	Sensitivity	98.3% (91.1–99.7)	96.7% (88.6–99.1)	64.4% (51.7–75.4)	71.7% (59.2–81.5)
	Specificity	98.3% (95.2–99.4)	95.0% (90.7–97.3)	90.0% (84.7–93.6)	93.9% (89.3–96.5)
Medium quality	Sensitivity	96.6% (88.5–99.1)	91.7% (81.9–96.4)	67.8% (55.1–78.3)	32.8% (22.1–45.6)
	Specificity	96.0% (92.1–98.1)	92.6% (87.8–95.6)	78.5% (71.9–83.9)	96.1% (92.1–98.1)
Low quality	Sensitivity	87.9% (77.1–94.0)	89.7% (79.2–95.2)	56.1% (43.3–68.2)	46.6% (34.3–59.2)
	Specificity	91.3% (86.2–94.7)	90.8% (85.5–94.2)	82.8% (76.5–87.6)	95.4% (91.1–97.6)
Experts	Sensitivity	96.5% (88.1–99.0)	93.3% (84.1–97.4)	62.1% (49.2–73.4)	52.6% (39.9–65.0)
	Specificity	93.7% (89.1–96.5)	94.2% (89.6–96.8)	85.1% (79.0–89.6)	95.4% (91.2–97.7)
Non-experts	Sensitivity	93.3% (87.4–96.6)	92.4% (86.1–95.9)	63.2% (54.2–71.4)	49.6% (40.8–58.4)
	Specificity	96.0% (93.5–97.6)	92.1% (88.9–94.5)	83.2% (79.0–86.7)	94.9% (92.1–96.8)

In general, the quality of the exochorion seemed to be more important than the level of entomological expertise of the rater. For high and medium quality, the estimates for sensitivity are higher than 92% for *Ae*. *albopictus* and *Ae*. *geniculatus*, but they are lower than 72% for *Ae*. *japonicus* and *Ae*. *koreicus*. When we consider the low quality of the exochorion, we see the same trend: sensitivity is higher and lower for the same species aforesaid, but in this case the estimates are lower than 90%. For all three exochorion qualities, the estimates of specificity are higher than 90%, except when the exochorion quality of *Ae*. *japonicus* is medium and low, less than 68% (Table 4).

No evidence for a difference between experts and non-experts is found when the two groups are compared. Both had less difficulties in identifying *Ae*. *albopictus* and *Ae*. *geniculatus*, for which estimates of sensitivity and specificity are higher than 92%, compared to the other two species: Table 4 shows more details. Additional accuracy measures, such as correctly classified proportion and positive and negative predictive values, were analysed and their results are shown in the S1 Table and S7 and S8 Figs. Both figures display the same data but with different emphasis. As a general result, the estimates for the additional accuracy measures are higher for high quality exochorions and there is no difference between experts and non-experts. Raters spent on median 13 minutes (interquartile range from 9.0 to 16.5) to complete the questionnaires.

### Test 2

We obtained 720 answers provided by the raters. Of these, 18 answers were omitted due to missing values. For *Ae*. *albopictus* and *Ae*. *geniculatus*, the determination accuracy is very high, more than 86% of eggs were correctly identified. In the case of *Ae*. *japonicus* and *Ae*. *koreicus*, the determination accuracy is low, less than 59% of eggs are correctly identified (Table 5).

**Table 5:** Test 2: Number and percentage of eggs correctly identified by the raters. Percentages of each column sum to 100%.

MALDI-TOF MS determination				
Answer of the rater	*Ae*. *albopictus*	*Ae*. *geniculatus*	*Ae*. *japonicus*	*Ae*. *koreicus*
*Ae*. *albopictus*	155 (88.6%)	17(9.7%)	32 (18.2%)	7 (4.0%)
*Ae*. *geniculatus*	13 (7.4%)	151 (86.3%)	19 (10.8%)	2 (1.1%)
*Ae*. *japonicus*	7 (4.0%)	3 (1.7%)	103 (58.5%)	67 (38.1%)
*Ae*. *koreicus*	0 (0.0%)	4 (2.3%)	22 (12.5%)	100 (56.8%)
Total	175 (100.0%)	175 (100.0%)	176 (100.0%)	176 (100.0%)

**Fig 3:**
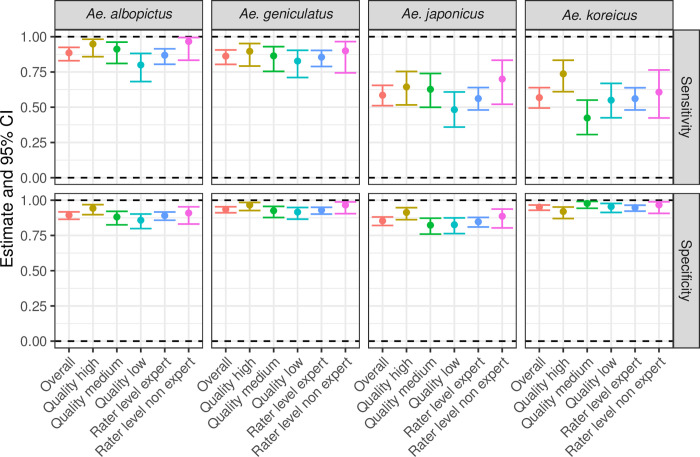
Test 2: Estimates (solid dots) and 95% confidence intervals (bars) for sensitivity and specificity for each group (overall, exochorion qualities, and levels of the rater) and each species.

Most images were correctly identified by experts and non-experts in entomology. Fig 3 and Table 5 show the estimates values for sensitivity and specificity for the same groups mentioned in the test 1. For overall identification, the estimates for specificity are very high, above 85%. *Aedes koreicus and Ae*. *japonicus* seemed to be more difficult to distinguish.

The quality of the exochorion appeared to have more importance than the level of the rater. It is easier to distinguish *Ae*. *albopictus* and *Ae*. *geniculatus* regardless of the exochorion quality. Their sensitivity estimates for all three qualities are higher (range 80–95%) compared to those of *Ae*. *japonicus* and *Ae*. *koreicus* (range 42–74%). The estimates of specificity for all three exochorion qualities are greater than 90%, except when the exochorion quality of *Ae*. *albopictus* and *Ae*. *japonicus* is medium or low (range 82–88%); for detailed estimates see Table 6.

**Table 6:** Test 2: Estimates and 95% confidence intervals (95%CI) for sensitivity and specificity for each group (overall, exochorion qualities, and levels of the rater) and each species.

Estimates (95%CI)					
Analyses	Accuracy measures	*Ae*. *albopictus*	*Ae*. *geniculatus*	*Ae*. *japonicus*	*Ae*. *koreicus*
Overall	Sensitivity	88.6% (83.0–92.5)	86.3% (80.4–90.6)	58.5% (51.1–65.5)	56.8% (49.4–63.9)
	Specificity	89.4% (86.5–91.7)	93.5% (91.1–95.3)	85.4% (82.1–88.1)	95.1% (92.9–96.6)
High quality	Sensitivity	94.8% (85.9–98.2)	89.7% (79.2–95.2)	64.4% (51.7–75.4)	73.7% (61.0–83.4)
	Specificity	94.3% (89.7–96.8)	96.6% (92.7–98.4)	91.3% (86.2–94.7)	92.0% (87.0–95.2)
Medium quality	Sensitivity	91.2% (81.1–96.2)	86.4% (75.5–93.0)	62.7% (50.0–73.9)	42.4% (30.6–55.1)
	Specificity	88.1% (82.5–92.1)	92.6% (87.7–95.6)	82.3% (76.0–87.2)	97.7% (94.3–99.1)
Low quality	Sensitivity	80.0% (68.2–88.2)	82.8% (71.1–90.4)	48.3% (35.9–60.8)	55.0% (42.5–66.9)
	Specificity	85.8% (79.9–90.2)	91.6% (86.6–94.8)	82.6% (76.3–87.5)	95.5% (91.3–97.7)
Experts	Sensitivity	86.9% (80.4–91.4)	85.5% (78.9–90.3)	56.2% (48.1–64.0)	56.1% (48.0–63.8)
	Specificity	89.1% (85.8–91.7)	92.9% (90.2–95.0)	84.7% (81.0–87.8)	94.7% (92.2–96.5)
Non-experts	Sensitivity	96.7% (83.3–99.4)	90.0% (74.4–96.5)	70.0% (52.1–83.3)	60.7% (42.4–76.4)
	Specificity	90.9% (83.1–95.3)	96.6% (90.5–98.8)	88.6% (80.3–93.7)	96.7% (90.7–98.9)

The species determination done by experts and non-experts did not present any differences when compared. Non-experts had less difficulties to assess the species of *Ae*. *albopictus* and *Ae*. *geniculatus*, whose estimates for sensitivity and specificity are higher than 90%. Same as in the first test, additional accuracy measures are listed in the S1 Table and S9 and S10 Figs. Both representations depict the same information, but with different emphasis. In general, the estimates for the additional accuracy measures are higher for high quality exochorions and non-experts. In addition, we wanted to see if there were differences when we compared individual raters. S11 Fig of the additional file does not show a specific pattern, we see that few raters had problems with certain species. On median, 11.6 minutes (interquartile range from 10.0 to 13.0) were spent to assess the species.

## Discussion

In this study, we demonstrated that with an appropriate training, eggs of certain *Aedes* species can be easily distinguished independently of entomological expertise. Answers given by experts (entomologists) and non-experts (general biologists) in both tests showed consistency in the results. *Aedes albopictus* and *Ae*. *geniculatus* were easily identified compared to *Ae*. *japonicus* and *Ae*. *koreicus*. This demonstration was achieved by the identification of unique traits of the exochorion using a high-resolution stereomicroscope after assessing their species via MALDI-TOF MS. The images obtained with a magnification of 112 times show differences in the exochorion of four Aedine species. Despite being from different sites, these distinct features remain conserved in the same species. *Aedes albopictus* and *Ae*. *geniculatus* eggs have a very unique chorionic cell, whereas *Ae*. *japonicus* and *Ae*. *koreicus* eggs are often interchanged, given their similar pattern, which could explain their low sensitivity and sensibility. These exochorionic traits are confirmed by previous studies that were mostly done using scanning electron microscope (SEM), which allows to see more details at a very high magnification [59–68]. However, SEM has important limitations. Samples must be silver-coated beforehand, which is expensive and takes time, and cannot be analysed further by other methods. In 2016, results of Bova et al. [80] focused more on the appearance of the egg, while we explored in detail the structure of the exochorion by looking at the chorionic cells with a higher magnitude (112 times). Another difference from their study is that measures of egg length, width, and their ratio were not considered.

The present study has some limitations. Firstly, the participants did not receive any intermediate feedback during their ratings, which may have resulted in some raters mixing up some species and worsen measures of accuracy. Secondly, the "per rater" analysis done in the second test was not balanced with respect to the quality of the images and the species and should therefore be considered more exploratory.

Several strengths are also shown in the study. Firstly, the images were randomized within the questionnaires, minimizing any potential biases that may have arisen from the order of image presentation. Secondly, the results were consistent across both tests, indicating the robustness of the findings. Additionally, the data collection worked overall well, and the data only had very few missing values. Although the sample size in the subgroups was smaller, resulting in larger confidence intervals, the subgroup analyses were pre-specified.

This study focused on eggs collected from the field, where biotic and abiotic environmental conditions cannot be controlled. They have influences on female mosquitoes and on their eggs [83–86]. In fact, during our surveillance programs we have sometimes found small and round eggs of *Ae*. *albopictus*and damaged exochorionic membranes. Therefore, the species could be confused when observing eggs with the naked eye and/or with the stereomicroscope. For this reason, it was important to include different states of degradation of the eggs in order to mimic the egg diversity that could be found on substrates during a surveillance program. The presence of a wide range of eggs makes it possible to find common characteristics in the three chorion qualities. The results confirm that when the exochorion membrane is intact, i.e., high quality, or has only a few characteristic traits, i.e., medium quality, it is easier for an examiner to determine the species. Clearly, it is possible to find different eggs whose species cannot be assessed due to their very bad condition, and it is an important limitation for optical recognition technique. Since wild female mosquitoes lay their eggs on substrates, the side of the egg that we examine under the stereomicroscope could be damaged, but the opposite side, which is in contact with the substrate, could have preserved its exochorionic structure. If all non-determinable eggs have to be rechecked, the time required to optically analyse the entire substrate would be very high. In addition to the degradation states, we included eggs from different locations in order to include potential morphological variations.

In surveillance programs, ovitraps do not allow a correct quantification of single species due to simultaneous presence of different Aedine species, since each species can lay single eggs. For this reason, new techniques have to be developed. Compared to optical recognition, PCR and MALDI-TOF MS allow the identification of only a small portion of the entire sample, which cannot be reused for further analyses and the likelihood of identifying potentially new species is very low [55]. With the increasing number of samples and new locations being occupied, MALDI-TOF MS is not sufficient to overcome the high demand for analysis. In addition, the freshness and the integrity of the laid egg are important factors to be considered. When eggs are not fresh enough and/or when the larvae have hatched, MALDI-TOF MS is not the best instrument. When the eggs collected from the field were not analysed during the following two weeks and left at room temperature, the spectra obtained were bad. To prevent sample degradation, it is advisable to store them at 4°C. Furthermore, by the end of the summer season, the spectra tend to be incorrect due to the presence of diapause eggs, which cannot easily be recognised within all eggs. It happened that few samples resulted *Ae*. *aegypti* but when the samples were reanalyses, it turned out to be a false alarm.

The optical analysis of the exochorion would not be adequate as a first line of defence in the event of the introduction of a new, unidentified invasive species. Molecular analysis, MALDI-TOF MS or PCR, will still be needed to verify the high-resolution image of the egg. To help improving surveillance and control, it will be useful to optically check the chorion of other *Aedes* species. Since they have similar shaped eggs, it could be advantageous to analyse samples present in countries were different *Aedes* species coexist. The presence of *Ae*. *cretinus* (Edwards) prevents researchers from accurately estimating the population density of *Ae*. *albopictus* in Greece, while *Ae*. *aegypti* does the same around the Black Sea region and the same may be the case in French Polynesia for eggs of *Ae*. *aegypti* and *Ae*. *polynesiensis* (Marks).

## Conclusions

Many countries in temperate regions have been exposed with the arrival and expansion of invasive mosquito that are potentially competent to transmit pathogens. In parallel, due to global tourism, tropical diseases, such as dengue and chikungunya, are increasingly recorded in temperate climates. Governmental health authorities are concerned and are constantly trying improving surveillance and control of invasive species. Here, we demonstrated that, with a good training done on images taken with a high-resolution stereomicroscope, any observer could potentially be able to assess the species of some Aedine mosquitoes. From our results, we saw that for participants it was easier to identify *Ae*. *albopictus* and *Ae*. *geniculatus* than *Ae*. *japonicus* and *Ae*. *koreicus*.

Being able to easily distinguish *Ae*. *albopictus* from other species is a major achievement since this species is the most concerning from a public health view. As a future perspective, it would be useful to optically analyse any other native or invasive container-inhabiting Aedine species laying eggs in ovitraps (e.g., *Ae*. *aegypti*, seen its higher vectorial competence and the recent observations in Europe).

## Supporting information

S1 Dataset Raw data set of test 1 with information regarding answers of each participant, optical determination done by the principal investigator and MALDI-TOF results. https://doi.org/10.6084/m9.figshare.24207942.
(CSV)

S2 Dataset Raw data set of test 2with information regarding answers of each participant, optical determination done by the principal investigator and MALDI-TOF results. https://doi.org/10.6084/m9.figshare.24207960.
(CSV)

S1 Fig Example of a small and round *Ae*. *albopictus* egg collected during the surveillance program. https://doi.org/10.6084/m9.figshare.24207930.
(TIF)

S2 Fig Examples of slides from the presentation given to participants. The main characteristics of the exochorion found for *Ae*. *albopictus* and *Ae*. *geniculatus* are shown. https://doi.org/10.6084/m9.figshare.24207945.
(TIF)

S3 Fig Examples of slides from the presentation given to participants. The main characteristics of the exochorion found for *Ae*. *japonicus* and *Ae*. *koreicus* are shown. https://doi.org/10.6084/m9.figshare.24207969.
(TIF)

S4 Fig Example of ‘high quality’ exochorions. Egg of *Aedes albopictus* (a), *Aedes geniculatus* (b), *Aedes japonicus* (c), and *Aedes koreicus* (d). https://doi.org/10.6084/m9.figshare.24207939.
(TIF)

S5 Fig Example of ‘medium quality’ exochorions. Egg of *Aedes albopictus* (a), *Aedes geniculatus* (b), *Aedes japonicus* (c), and *Aedes koreicus* (d). https://doi.org/10.6084/m9.figshare.24207954.
(TIF)

S6 Fig Example of ‘low quality’ exochorions. Egg of *Aedes albopictus* (a), *Aedes geniculatus* (b), *Aedes japonicus* (c), and *Aedes koreicus* (d). https://doi.org/10.6084/m9.figshare.24207957.
(TIF)

S7 Fig Test 1: Estimates (solid dots) and 95% confidence intervals (bars) for all accuracy measures for different groups and each species. https://doi.org/10.6084/m9.figshare.24207948.
(TIF)

S8 Fig Test 1: Estimates (solid dots) and 95% confidence intervals (bars) for all accuracy measures for species and different groups. https://doi.org/10.6084/m9.figshare.24207963.
(TIF)

S9 Fig Test 2: Estimates (solid dots) and 95% confidence intervals (bars) for all accuracy measures for different groups and each species. https://doi.org/10.6084/m9.figshare.24207972.
(TIF)

S10 Fig Test 2: Estimates (solid dots) and 95% confidence intervals (bars) for all accuracy measures for species and different groups. https://doi.org/10.6084/m9.figshare.24207951.
(TIF)

S11 Fig Test 2: Estimates (solid dots) and 95% confidence intervals (bars) for all accuracy measures for each rater and each species. https://doi.org/10.6084/m9.figshare.24207966.
(TIF)

S1 Table Descriptions (Table 1), estimates and 95% confidence intervals (95%CI) for all diagnostic accuracy measures (Tables 2 and 3). https://doi.org/10.6084/m9.figshare.24208149.v3.
(DOCX)

S1 Text R-script for all analyses of test 1. https://doi.org/10.6084/m9.figshare.24367597.v2.
(PDF)

S2 Text R-script for all analyses of test 2. https://doi.org/10.6084/m9.figshare.24368251.
(PDF)

S3 Text Descriptions and short explanations of S1 and S2 Datasets. https://doi.org/10.6084/m9.figshare.24207933.v2.
(PDF)

## References

[1] Medlock JM, Hansford KM, Schaffner F, Versteirt V, Hendrickx G, Zeller H, et al. A review of the invasive mosquitoes in Europe: Ecology, public health risks, and control options. Vol. 12, Vector-Borne and Zoonotic Diseases. 2012. p. 435–47. doi: 10.1089/vbz.2011.0814 22448724 PMC3366101

[2] Butler D. Europe on alert for flying invaders. Nature [Internet]. 2012 Sep 13 [cited 2023 Feb 21];489:187–8. Available from: https://www.nature.com/articles/489187a doi: 10.1038/489187a 22972271

[3] Schaffner F, Medlock JM, Van Bortel W. Public health significance of invasive mosquitoes in Europe. Vol. 19, Clinical Microbiology and Infection. Blackwell Publishing Ltd; 2013. p. 685–92. 23574618 10.1111/1469-0691.12189

[4] Gould EA, Higgs S. Impact of climate change and other factors on emerging arbovirus diseases. Trans R Soc Trop Med Hyg. 2009;103(2):109–21. doi: 10.1016/j.trstmh.2008.07.025 18799177 PMC2915563

[5] Hales S, De Wet N, Maindonald J, Woodward A. Potential effect of population and climate changes on global distribution of dengue fever: an empirical model. The Lancet. 2002;360(9336):830–4. doi: 10.1016/S0140-6736(02)09964-6 12243917

[6] Vanwambeke SO, Lambin EF. Environmental change and vector-borne diseases: The contribution of remote sensing and spatial analyses. Med Health Sci. 2010;8:231–9.

[7] European Centre for Disease Prevention and Control (ECDC). Consultation on mosquito-borne disease transmission risk in Europe. [Internet]. Paris; 2010 Nov [cited 2023 Feb 21]. Available from: https://www.ecdc.europa.eu/sites/default/files/media/en/publications/Publications/1102_MER_Consultation_on_%20mosquito-borne_diseases.pdf

[8] Cancrini G, Romi R, Gabrielli S, Toma L, Di Paolo M, Scaramozzino P. First finding of Dirofilaria repens in a natural population of Aedes albopictus. Med Vet Entomol. 2003;17(4):448–51. doi: 10.1111/j.1365-2915.2003.00463.x 14651660

[9] Cancrini G, Di Regalbono AF, Ricci I, Tessarin C, Gabrielli S, Pietrobelli M. Aedes albopictus is a natural vector of Dirofilaria immitis in Italy. Vet Parasitol. 2003;118(3–4):195–202. doi: 10.1016/j.vetpar.2003.10.011 14729167

[10] Feng LC. The tree hole species of mosquitoes of Peiping, China. Chin Med J. 1938;2(Suppl 2):503–25.

[11] Pampiglione S, Rivasi F, Angeli G, Boldorini R, Incensati RM, Pastormerlo M, et al. Dirofilariasis due to Dirofilaria repens in Italy, an emergent zoonosis: report of 60 new cases. Histopathology. 2001;38(4):344–54. doi: 10.1046/j.1365-2559.2001.01099.x 11318900

[12] Paupy C, Delatte H, Bagny L, Corbel V, Fontenille D. Aedes albopictus, an arbovirus vector: from the darkness to the light. Microbes Infect. 2009;11(14–15):1177–85. doi: 10.1016/j.micinf.2009.05.005 19450706

[13] Kraemer MUG, Sinka ME, Duda KA, Mylne AQN, Shearer FM, Barker CM, et al. The global distribution of the arbovirus vectors Aedes aegypti and Ae. albopictus. Elife. 2015;4:e08347. doi: 10.7554/eLife.08347 26126267 PMC4493616

[14] Laporta GZ, Potter AM, Oliveira JFA, Bourke BP, Pecor DB, Linton YM. Global Distribution of Aedes aegypti and Aedes albopictus in a Climate Change Scenario of Regional Rivalry. Insects. 2023;14(1):49. doi: 10.3390/insects14010049 36661976 PMC9860750

[15] Adhami J, Reiter P. Introduction and establishment of Aedes (Stegomyia) albopictus skuse (Diptera: Culicidae) in Albania. J Am Mosq Control Assoc. 1998;14(3):340–3. 9813831

[16] Sabatini A, Raineri V, Trovato G, Coluzzi M. Aedes albopictus in Italy and possible diffusion of the species into the Mediterranean area. Parassitologia. 1990;32(3):301–4. 2132441

[17] Scholte EJ, Schaffner F. 14. Waiting for the tiger: establishment and spread of the Aedes albopictus mosquito in Europe. Emerging pests and vector-borne diseases in Europe. 2007;1:241.

[18] Scholte EJ, Jacobs F, Linton YM, Dijkstra E, Fransen J, Takken W. First record of Aedes (Stegomyia) albopictus in the Netherlands. Eur Mosq Bull. 2007;22(5):9.

[19] European Centre for Disease Prevention and Control (ECDC), European Safety Authority. Mosquito maps [internet]. [Internet]. Stockholm; 2023 [cited 2023 Mar 20]. Available from: https://www.ecdc.europa.eu/en/disease-vectors/surveillance-and-disease-data/mosquito-maps

[20] Aranda C, Eritja R, Roiz D. First record and establishment of the mosquito Aedes albopictus in Spain. Med Vet Entomol. 2006;20(1):150–2. doi: 10.1111/j.1365-2915.2006.00605.x 16608499

[21] Angelini R, Finarelli AC, Angelini P, Po C, Petropulacos K, Macini P, et al. An outbreak of chikungunya fever in the province of Ravenna, Italy. Weekly releases (1997–2007). 2007;12(36):3260. doi: 10.2807/esw.12.36.03260-en 17900424

[22] Gould EA, Gallian P, De Lamballerie X, Charrel RN. First cases of autochthonous dengue fever and chikungunya fever in France: from bad dream to reality! Clinical microbiology and infection. 2010;16(12):1702–4. doi: 10.1111/j.1469-0691.2010.03386.x 21040155

[23] La Ruche G, Souarès Y, Armengaud A, Peloux-Petiot F, Delaunay P, Desprès P, et al. First two autochthonous dengue virus infections in metropolitan France, September 2010. Eurosurveillance. 2010;15(39):19676. 20929659

[24] Herrero-Martínez JM, Sánchez-Ledesma M, Ramos-Rincón JM. Imported and autochthonous dengue in Spain. Revista Clínica Española (English Edition). 2023; doi: 10.1016/j.rceng.2023.07.007 37507047

[25] Monge S, García-Ortúzar V, Hernández BL, Pérez MÁL, Delacour-Estrella S, Sánchez-Seco MP, et al. Characterization of the first autochthonous dengue outbreak in Spain (August–September 2018). Acta Trop. 2020;205:105402. doi: 10.1016/j.actatropica.2020.105402 32088276

[26] Gjenero-Margan I, Aleraj B, Krajcar D, Lesnikar V, Klobučar A, Pem-Novosel I, et al. Autochthonous dengue fever in Croatia, august–September 2010. Eurosurveillance. 2011;16(9):19805. 21392489

[27] Flacio E, Engeler L, Tonolla M, Müller P. Spread and establishment of Aedes albopictus in southern Switzerland between 2003 and 2014: an analysis of oviposition data and weather conditions. Parasit Vectors. 2016;9:1–14. 27229686 10.1186/s13071-016-1577-3 PMC4882898

[28] Flacio E, Engeler L, Tonolla M, Lüthy P, Patocchi N. Strategies of a thirteen year surveillance programme on Aedes albopictus (Stegomyia albopicta) in southern Switzerland. Parasit Vectors. 2015;8:1–18. 25890173 10.1186/s13071-015-0793-6 PMC4406169

[29] Müller P, Engeler L, Vavassori L, Suter T, Guidi V, Gschwind M, et al. Surveillance of invasive Aedes mosquitoes along Swiss traffic axes reveals different dispersal modes for Aedes albopictus and Ae. japonicus. PLoS Negl Trop Dis. 2020;14(9):e0008705. 10.1371/journal.pntd.0008705 PMC7544034 32986704

[30] Schaffner F, Kaufmann C, Hegglin D, Mathis A. The invasive mosquito Aedes japonicus in Central Europe. Med Vet Entomol. 2009;23(4):448–51. doi: 10.1111/j.1365-2915.2009.00825.x 19941611

[31] Kaufman MG, Fonseca DM. Invasion biology of Aedes japonicus japonicus (Diptera: Culicidae). Annu Rev Entomol. 2014;59:31–49. doi: 10.1146/annurev-ento-011613-162012 24397520 PMC4106299

[32] Capelli G, Drago A, Martini S, Montarsi F, Soppelsa M, Delai N, et al. First report in Italy of the exotic mosquito species Aedes (Finlaya) koreicus, a potential vector of arboviruses and filariae. Parasit Vectors. 2011;4:1–5. 21951867 10.1186/1756-3305-4-188 PMC3203849

[33] Montarsi F, Drago A, Martini S, Calzolari M, De Filippo F, Bianchi A, et al. Current distribution of the invasive mosquito species, Aedes koreicus [Hulecoeteomyia koreica] in northern Italy. Parasit Vectors. 2015 Dec 1;8(1). doi: 10.1186/s13071-015-1208-4 26626019 PMC4666031

[34] Montarsi F, Drago A, Dal Pont M, Delai N, Carlin S, Cazzin S, et al. Current knowledge on the distribution and biology of the recently introduced invasive mosquito Aedes koreicus (Diptera: Culicidae). Atti Accademia Nazionale Italiana di Entomologia Anno LXII. 2014.

[35] Arnoldi I, Negri A, Soresinetti L, Brambilla M, Carraretto D, Montarsi F, et al. Assessing the distribution of invasive Asian mosquitoes in Northern Italy and modelling the potential spread of Aedes koreicus in Europe. Acta Trop. 2022;232:106536. doi: 10.1016/j.actatropica.2022.106536 35609630

[36] Fuehrer HP, Schoener E, Weiler S, Barogh BS, Zittra C, Walder G. Monitoring of alien mosquitoes in western Austria (Tyrol, Austria, 2018). PLoS Negl Trop Dis. 2020;14(6):e0008433. doi: 10.1371/journal.pntd.0008433 32574163 PMC7337398

[37] Kurucz K, Kiss V, Zana B, Schmieder V, Kepner A, Jakab F, et al. Emergence of Aedes koreicus (Diptera: Culicidae) in an urban area, Hungary, 2016. Parasitol Res. 2016;115:4687–9. doi: 10.1007/s00436-016-5229-5 27511369

[38] Versteirt V, Pecor JE, Fonseca DM, Coosemans M, Van Bortel W. Confirmation of Aedes koreicus (Diptera: Culicidae) in Belgium and description of morphological differences between Korean and Belgian specimens validated by molecular identification. Zootaxa. 2012;3191(1):21–32.

[39] Steinbrink A, Zotzmann S, Cunze S, Klimpel S. Aedes koreicus—a new member of the genus Aedes establishing in Germany? Parasitol Res. 2019 Mar 14;118(3):1073–6. doi: 10.1007/s00436-019-06232-x 30734861

[40] Kalan K, Šušnjar J, Ivović V, Buzan E. First record of Aedes koreicus (Diptera, Culicidae) in Slovenia. Parasitol Res. 2017;116:2355–8. doi: 10.1007/s00436-017-5532-9 28624875

[41] Ganushkina LA, Patraman I V, Rezza G, Migliorini L, Litvinov SK, Sergiev VP. Detection of Aedes aegypti, Aedes albopictus, and Aedes koreicus in the Area of Sochi, Russia. Vector-borne and zoonotic diseases. 2016;16(1):58–60. doi: 10.1089/vbz.2014.1761 26741323

[42] Turell MJ, O’Guinn ML, Dohm DJ, Jones JW. Vector competence of North American mosquitoes (diptera: culicidae) for West Nile virus. J Med Entomol. 2001;38(2):130–4. doi: 10.1603/0022-2585-38.2.130 11296813

[43] Sardelis MR, Turell MJ. Ochlerotatus j. japonicus in Frederick County, Maryland: discovery, distribution, and vector competence for West Nile virus. Journal of the American Mosquito Control Association-Mosquito News. 2001;17(2):137–41. 11480822

[44] Takashima I, Rosen L. Horizontal and vertical transmission of Japanese encephalitis virus by Aedes japonicus (Diptera: Culicidae). J Med Entomol. 1989;26(5):454–8. doi: 10.1093/jmedent/26.5.454 2552120

[45] Sardelis MR, Turell MJ, Andre RG. Laboratory transmission of La Crosse virus by Ochlerotatus j. japonicus (Diptera: Culicidae). J Med Entomol. 2002;39(4):635–9. doi: 10.1603/0022-2585-39.4.635 12144295

[46] Sardelis MR, Dohm DJ, Pagac B, Andre RG, Turell MJ. Experimental transmission of eastern equine encephalitis virus by Ochlerotatus j. japonicus (Diptera: Culicidae). J Med Entomol. 2002;39(3):480–4. doi: 10.1603/0022-2585-39.3.480 12061444

[47] Sardelis MR, Turell MJ, Andre RG. Experimental transmission of St. Louis encephalitis virus by Ochlerotatus j. japonicus. J Am Mosq Control Assoc. 2003;19(2):159–62. 12825669

[48] Schaffner F, Bellini R, Petrić D, Scholte EJ, Zeller H, Marrama Rakotoarivony L. Development of guidelines for the surveillance of invasive mosquitoes in Europe. Parasit Vectors. 2013;6(1). doi: 10.1186/1756-3305-6-209 23866915 PMC3724590

[49] Marrama Rakotoarivony L, Schaffner F. ECDC guidelines for the surveillance of invasive mosquitoes in Europe. European Centre for Disease Prevention and Control (ECDC); 2012. 22971331

[50] Bellini Centro Agricoltura Ambiente R, Nicoli G, Veljko Petrić D, Schaffner F. Practical management plan for invasive mosquito species in Europe: I. Asian tiger mosquito (Aedes albopictus)—ANNEX 2: Standard Operational Procedures for egg counting. Travel Med Infect Dis [Internet]. 2020; Available from: https://www.researchgate.net/publication/340814360 10.1016/j.tmaid.2020.101691 32334085

[51] Bellini Centro Agricoltura Ambiente R, Nicoli G, Veljko Petrić D, Schaffner F. Practical management plan for invasive mosquito species in Europe: I. Asian tiger mosquito (Aedes albopictus)—ANNEX 1: Standard Operational Procedures for ovitrap field management. Travel Med Infect Dis [Internet]. 2020; Available from: https://www.researchgate.net/publication/340813851 10.1016/j.tmaid.2020.101691 32334085

[52] Bellini R, Michaelakis A, Petrić D, Schaffner F, Alten B, Angelini P, et al. Practical management plan for invasive mosquito species in Europe: I. Asian tiger mosquito (Aedes albopictus). Travel Med Infect Dis. 2020;35:101691. doi: 10.1016/j.tmaid.2020.101691 32334085

[53] Thaggard CW, Eliason DA. Field evaluation of components for an Aedes aegypti (L.) oviposition trap. Mosq News. 1969;29(4).

[54] Bellini R, Carrieri M, Burgio G, Bacchi M. Efficacy of different ovitraps and binomial sampling in Aedes albopictus surveillance activity. J Am Mosq Control Assoc. 1996;12(4):632–6. 9046468

[55] Schaffner F, Kaufmann C, Pflüger V, Mathis A. Rapid protein profiling facilitates surveillance of invasive mosquito species. Parasit Vectors. 2014 Mar 31;7(1). 10.1186/1756-3305-7-142 PMC4022357 24685094

[56] Harbach RE, Knight KL. A mosquito taxonomic glossary. XV. The egg. Mosquito Systematics (USA). 1978;

[57] Mattingly PF. Mosquito eggs VIII. Mosq Syst Newsl. 1970;2:87–91.

[58] Harbach RE, Knight KL. Taxonomists’ glossary of mosquito anatomy. Plexus Publishing Inc.; 1980.

[59] Matsuo K, Yoshida Y, Lien J. Scanning electron microscopy of mosquitoes. II. The egg surface structure of 13 species of Aedes from Taiwan. J Med Entomol. 1974;11(2):179–88. doi: 10.1093/jmedent/11.2.179 4462617

[60] Matsuo K. Scanning electron microscopy of mosquitoes. 1975;26(1):49–53.

[61] Hinton HE, Service MW. The surface structure of aedine eggs as seen with the scanning electron microscope. Ann Trop Med Parasitol. 1969;63(4):409–12. doi: 10.1080/00034983.1969.11686643 5394017

[62] Faull KJ, Williams CR. Differentiation of Aedes aegypti and Aedes notoscriptus (Diptera: Culicidae) eggs using scanning electron microscopy. Arthropod Struct Dev. 2016 May 1;45(3):273–80. doi: 10.1016/j.asd.2016.01.009 26845557

[63] Kim H, Sun Y, Bae CG, Moon MJ. Structural characterization of the micropatterned egg plastron in the mosquito, Aedes albopictus. Entomol Res. 2020 Apr 1;50(4):189–98.

[64] Linley JR. Scanning electron microscopy of the egg of Aedes (Protomacleaya) triseriatus (Diptera: Culicidae). J Med Entomol. 1989;26(5):474–8. doi: 10.1093/jmedent/26.5.474 2795619

[65] Linley JR. Comparative fine structure of the eggs of Aedes albopictus, Ae. aegypti, and Ae. bahamensis (Diptera: Culicidae). J Med Entomol. 1989;26(6):510–21. doi: 10.1093/jmedent/26.6.510 2585445

[66] Linley JR, Craig GB. Morphology of long- and short-day eggs of Aedes atropalpus and A. epactius (Diptera: Culicidae). J Med Entomol. 1994;31(6):855–67. doi: 10.1093/jmedent/31.6.855 7815398

[67] Suman DS, Shrivastava AR, Pant SC, Parashar BD. Differentiation of Aedes aegypti and Aedes albopictus (Diptera: Culicidae) with egg surface morphology and morphometrics using scanning electron microscopy. Arthropod Struct Dev [Internet]. 2011;40(5):479–83. Available from: doi: 10.1016/j.asd.2011.04.003 21920819

[68] Win MM, Swe TT, Sett KM, Mya MM, Latt AZ, Soe K, et al. Structural differentiation of Aedes aegypti and Aedes albopictus eggs using scanning electron microscope. Journal of Biological Engineering Research and Review. 2018;5(1):9–12.

[69] Garcia PSC, Martins R, Coelho GLLM, Cámara-Chávez G. Acquisition of digital images and identification of Aedes Aegypti mosquito eggs using classification and deep learning. In: 2019 32nd SIBGRAPI Conference on Graphics, Patterns and Images (SIBGRAPI). IEEE; 2019. p. 47–53.

[70] Elpídio FGG, Costa LFR, Pucci GL, Andrade MM, Costa EA. Automatic Identification of Aedes Aegypti Eggs Deposited in Ovitraps Slides Using Image Processing Techniques. In: XXII Brazilian Congress of Biomedical Engineering. 2010.

[71] Beumier C, Rubio J, Andreo V, Guzman C, Porcasi X, Scavuzzo CM, et al. Semi-Automatic Tool to Count Mosquito Eggs in Ovitrap Stick Images. In: 2021 IEEE International Geoscience and Remote Sensing Symposium IGARSS. IEEE; 2021. p. 80–3.

[72] Mollahosseini A, Rossignol M, Pennetier C, Cohuet A, Anjos A dos, Chandre F, et al. A user-friendly software to easily count Anopheles egg batches. Parasit Vectors. 2012;5:1–7. 22713553 10.1186/1756-3305-5-122 PMC3464736

[73] Gaburro J, Duchemin JB, Paradkar PN, Nahavandi S, Bhatti A. Assessment of ICount software, a precise and fast egg counting tool for the mosquito vector Aedes aegypti. Parasit Vectors. 2016;9(1):1–9. doi: 10.1186/s13071-016-1870-1 27863526 PMC5116143

[74] Mello CAB, Dos Santos WP, Rodrigues MAB, Candeias ALB, Gusmão CMG. Image segmentation of ovitraps for automatic counting of Aedes Aegypti eggs. Proceedings of the 30th Annual International Conference of the IEEE Engineering in Medicine and Biology Society, EMBS’08 - “Personalized Healthcare through Technology.” 2008;000:3103–6. doi: 10.1109/IEMBS.2008.4649860 19163363

[75] Mains JW, Mercer DR, Dobson SL. Digital Image Analysis to Estimate Numbers of Aedes Eggs Oviposited in Containers. J Am Mosq Control Assoc. 2008;24(4):496–501. doi: 10.2987/5740.1 19181055 PMC2705870

[76] Gusmão G, Machado SCS, Rodrigues MAB, Portela NM, Mello CAB, Dos Santos WP, et al. A new algorithm for segmenting and counting Aedes aegypti eggs in Ovitraps. Proceedings of the 31st Annual International Conference of the IEEE Engineering in Medicine and Biology Society: Engineering the Future of Biomedicine, EMBC 2009. 2009;6714–7. doi: 10.1109/IEMBS.2009.5333759 19964446

[77] Da Silva MGNM, Rodrigues MAB, De Araujo RE. Aedes aegypti egg counting system. Proceedings of the Annual International Conference of the IEEE Engineering in Medicine and Biology Society, EMBS. 2011;6810–2. doi: 10.1109/IEMBS.2011.6091679 22255902

[78] Obenauer PJ, Buss LJ, Kaufman PE. Utilizing Auto-Montage^TM^ Technology for Identifying Field-Collected Container-Inhabiting Mosquito Eggs1. J Am Mosq Control Assoc. 2009;25(4):517–20. 20099602 10.2987/09-5899.1

[79] Bova JE, Hawley DM. Morphological differentiation of eggs and comparative efficacy of oviposition and gravid traps for Aedes vectors at different habitats. 2014.

[80] Bova J, Paulson S, Paulson G. Morphological Differentiation of the Eggs of North American Container-Inhabiting Aedes Mosquitoes. J Am Mosq Control Assoc. 2016;32(3):244–6. doi: 10.2987/15-6535.1 27802396

[81] R Core Team. R: A language and environment for statistical computing. [Internet]. Vienna (Austria): R Foundation for Statistical Computing; 2022 [cited 2023 Mar 30]. Available from: https://www.R-project.org/

[82] Stevenson M, Sergeant E, Nunes T, Heuer C, Marshall J, Sanchez J, et al. epiR: Tools for the Analysis of Epidemiological Data [Internet]. 2022 [cited 2023 Apr 3]. Available from: https://CRAN.R-project.org/package=epiR

[83] Farnesi LC, Barbosa CS, Araripe LO, Bruno RV. The influence of a light and dark cycle on the egg laying activity of Aedes aegypti (Linnaeus, 1762)(Diptera: Culicidae). Mem Inst Oswaldo Cruz. 2018;113. doi: 10.1590/0074-02760170362 29412343 PMC5851057

[84] Aïssaoui L, Boudjelida H. Larvicidal activity and influence of Bacillus thuringiensis (Vectobac G), on longevity and fecundity of mosquito species. Eur J Exp Biol. 2014;4(1):104–9.

[85] Foster WA. Mosquito sugar feeding and reproductive energetics. Annu Rev Entomol. 1995;40(1):443–74. doi: 10.1146/annurev.en.40.010195.002303 7810991

[86] Costa EAP de A, Santos EM de M, Correia JC, Albuquerque CMR de. Impact of small variations in temperature and humidity on the reproductive activity and survival of Aedes aegypti (Diptera, Culicidae). Rev Bras Entomol. 2010;54:488–93.

