# Supplementary figures and images for: Optical recognition of the eggs of four Aedine mosquito species (Aedes albopictus, Aedes geniculatus, Aedes japonicus, and Aedes koreicus)
Source: PLoS One. 2023 Nov 1;18(11):e0293568. doi: 10.1371/journal.pone.0293568 (PMC10619821; doi:10.1371/journal.pone.0293568)

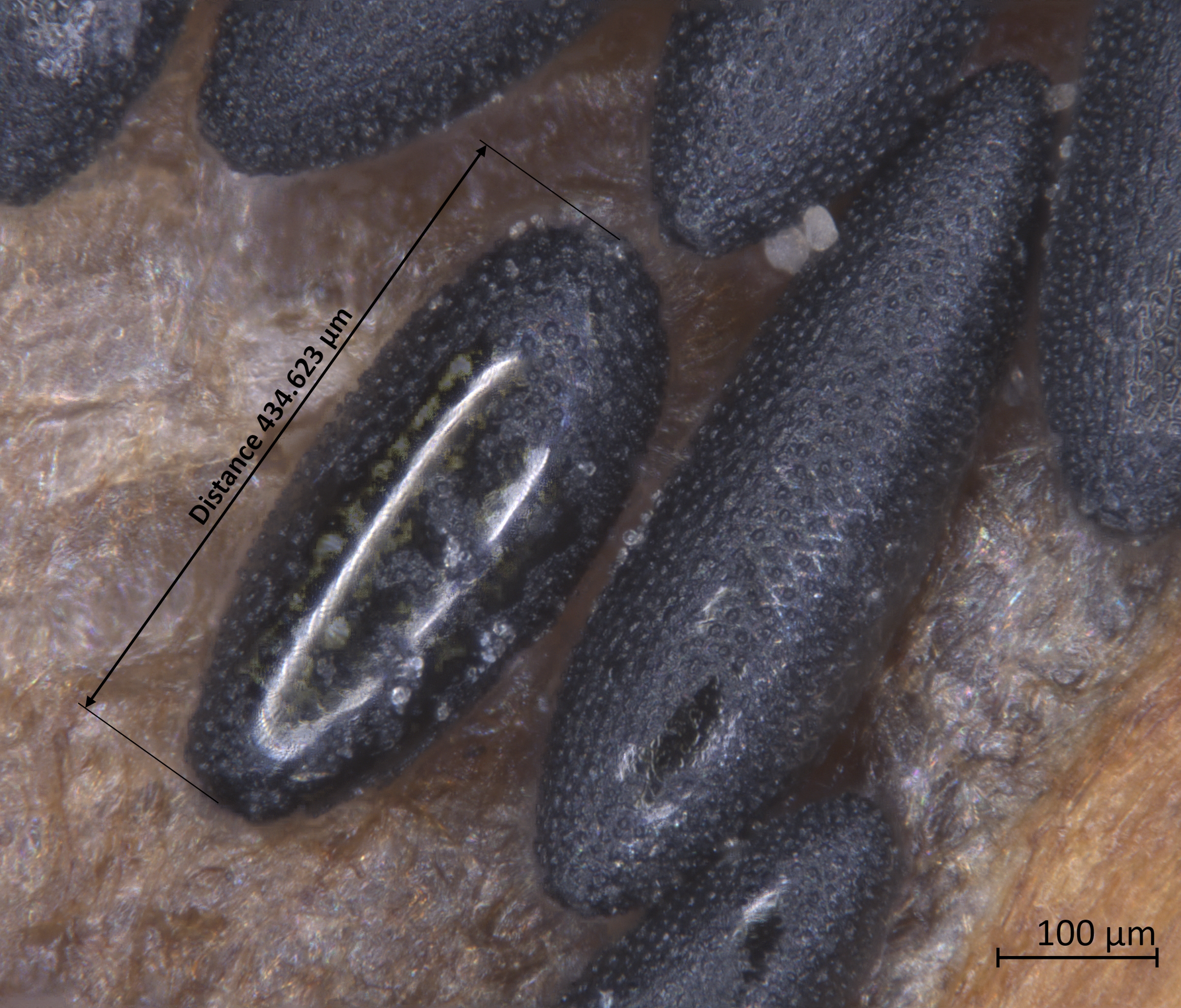

Supplement: S1 Fig — https://doi.org/10.6084/m9.figshare.24207930. (TIF) [file pone.0293568.s003.tif]

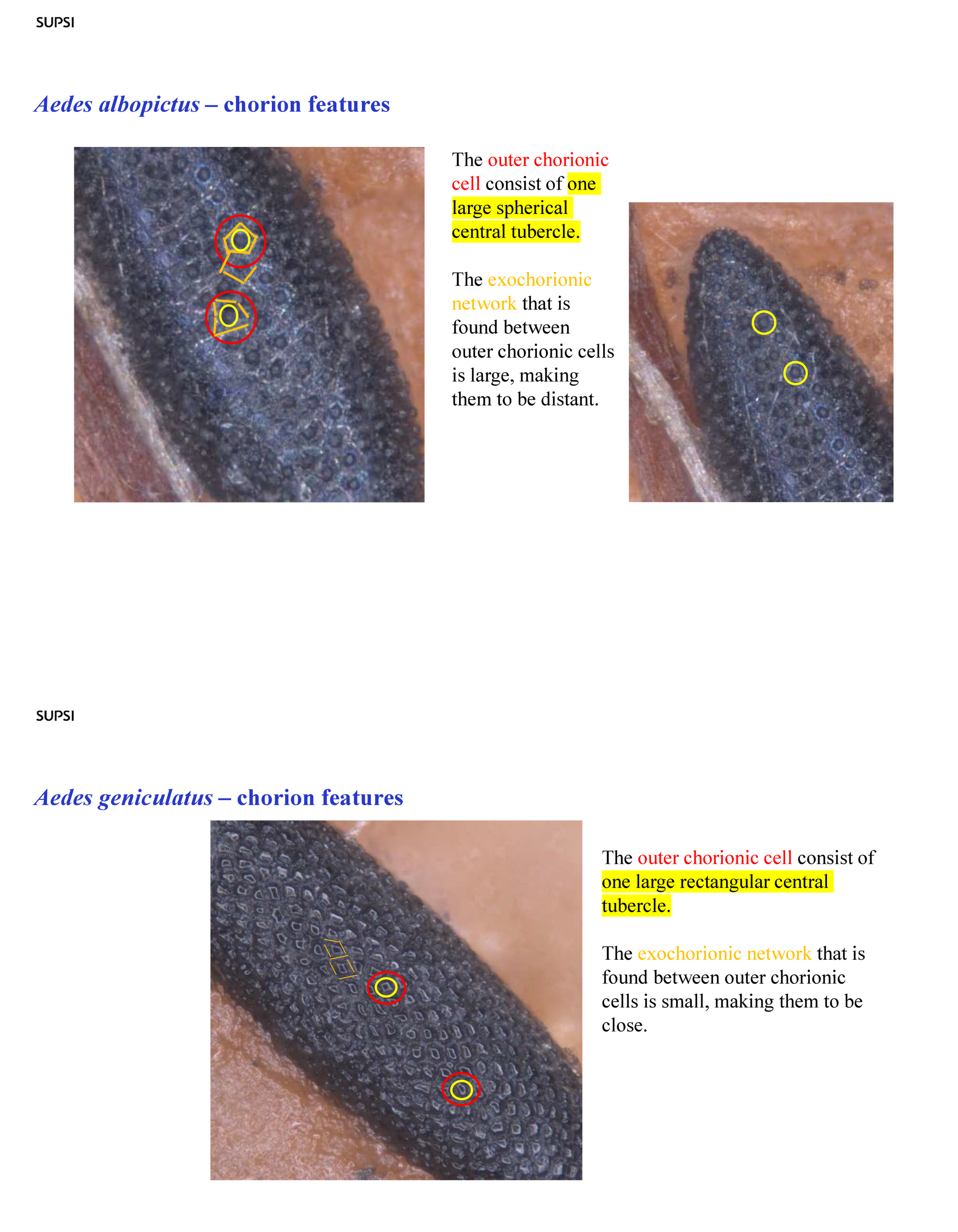

Supplement: S2 Fig — The main characteristics of the exochorion found for Ae. albopictus and Ae. geniculatus are shown. https://doi.org/10.6084/m9.figshare.24207945. (TIF) [file pone.0293568.s004.tif]

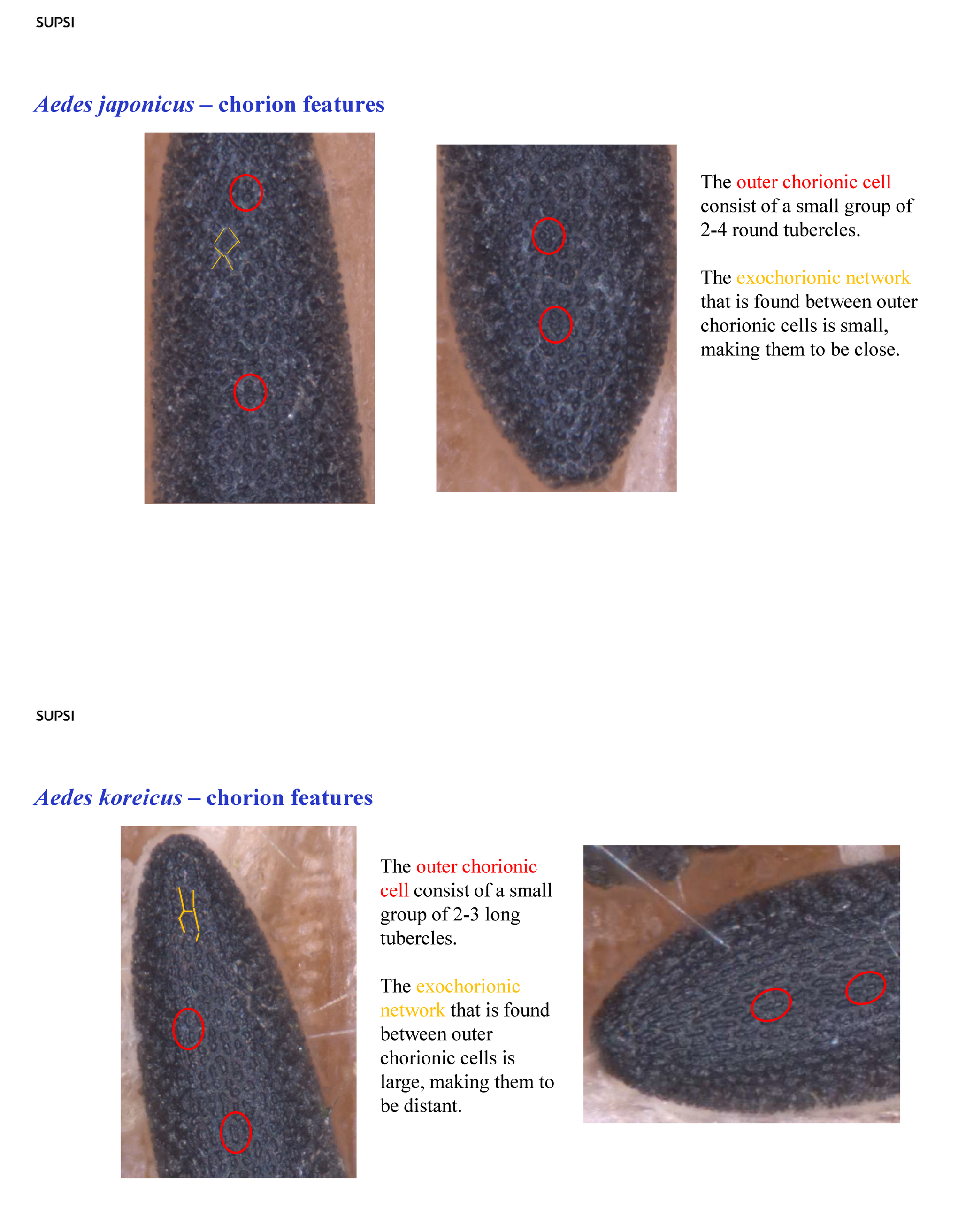

Supplement: S3 Fig — The main characteristics of the exochorion found for Ae. japonicus and Ae. koreicus are shown. https://doi.org/10.6084/m9.figshare.24207969. (TIF) [file pone.0293568.s005.tif]

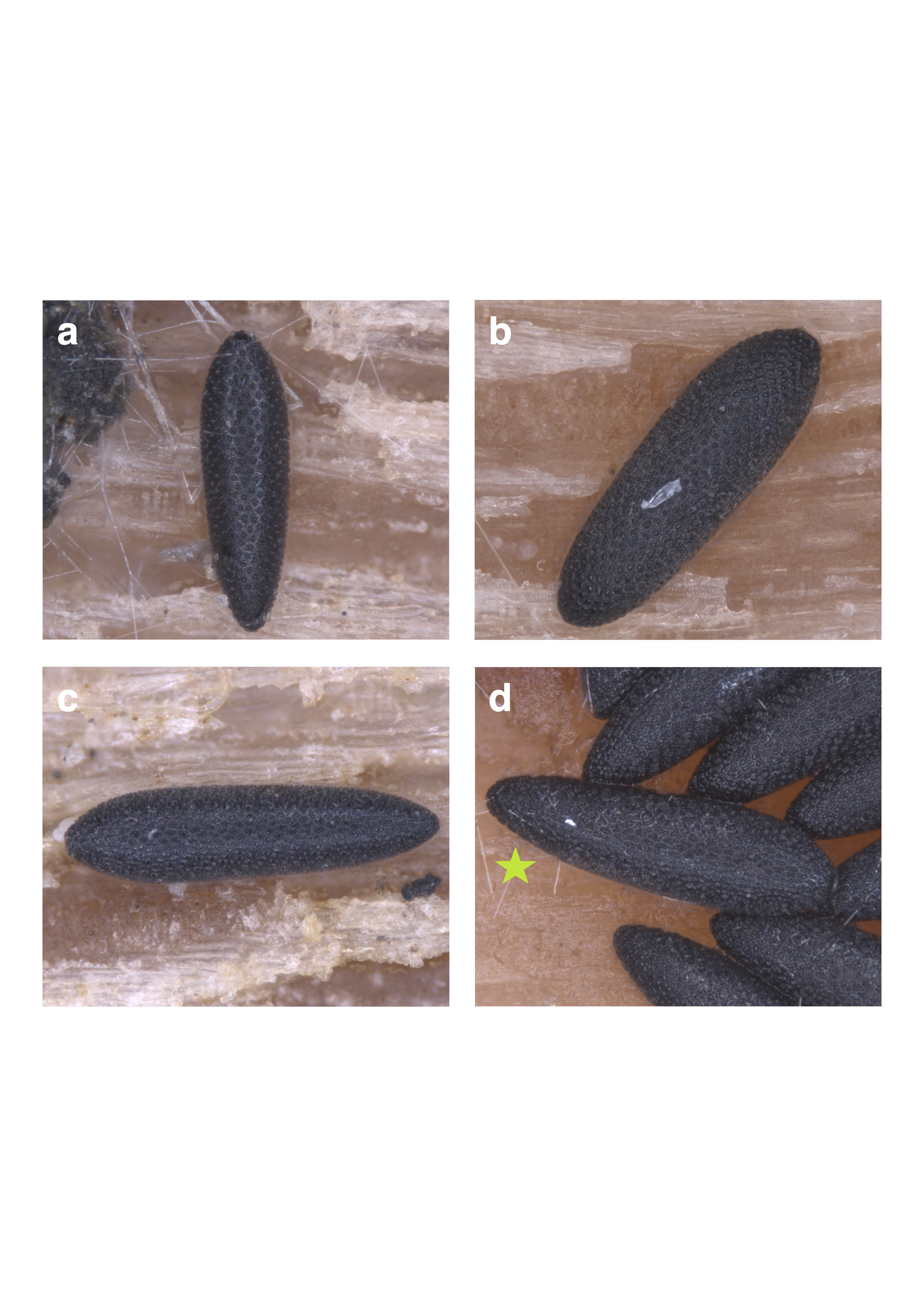

Supplement: S4 Fig — Egg of Aedes albopictus (a), Aedes geniculatus (b), Aedes japonicus (c), and Aedes koreicus (d). https://doi.org/10.6084/m9.figshare.24207939. (TIF) [file pone.0293568.s006.tif]

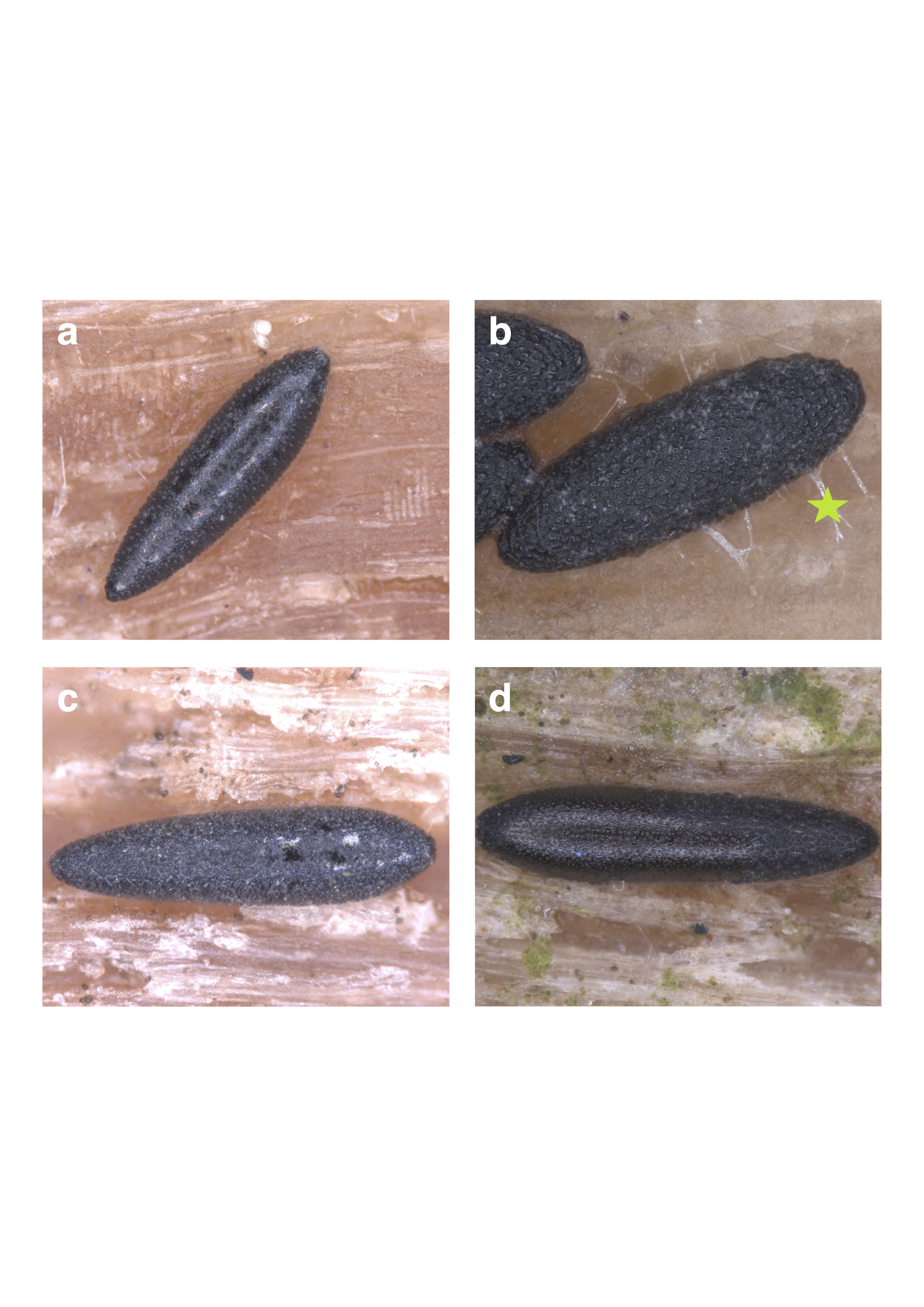

Supplement: S5 Fig — Egg of Aedes albopictus (a), Aedes geniculatus (b), Aedes japonicus (c), and Aedes koreicus (d). https://doi.org/10.6084/m9.figshare.24207954. (TIF) [file pone.0293568.s007.tif]

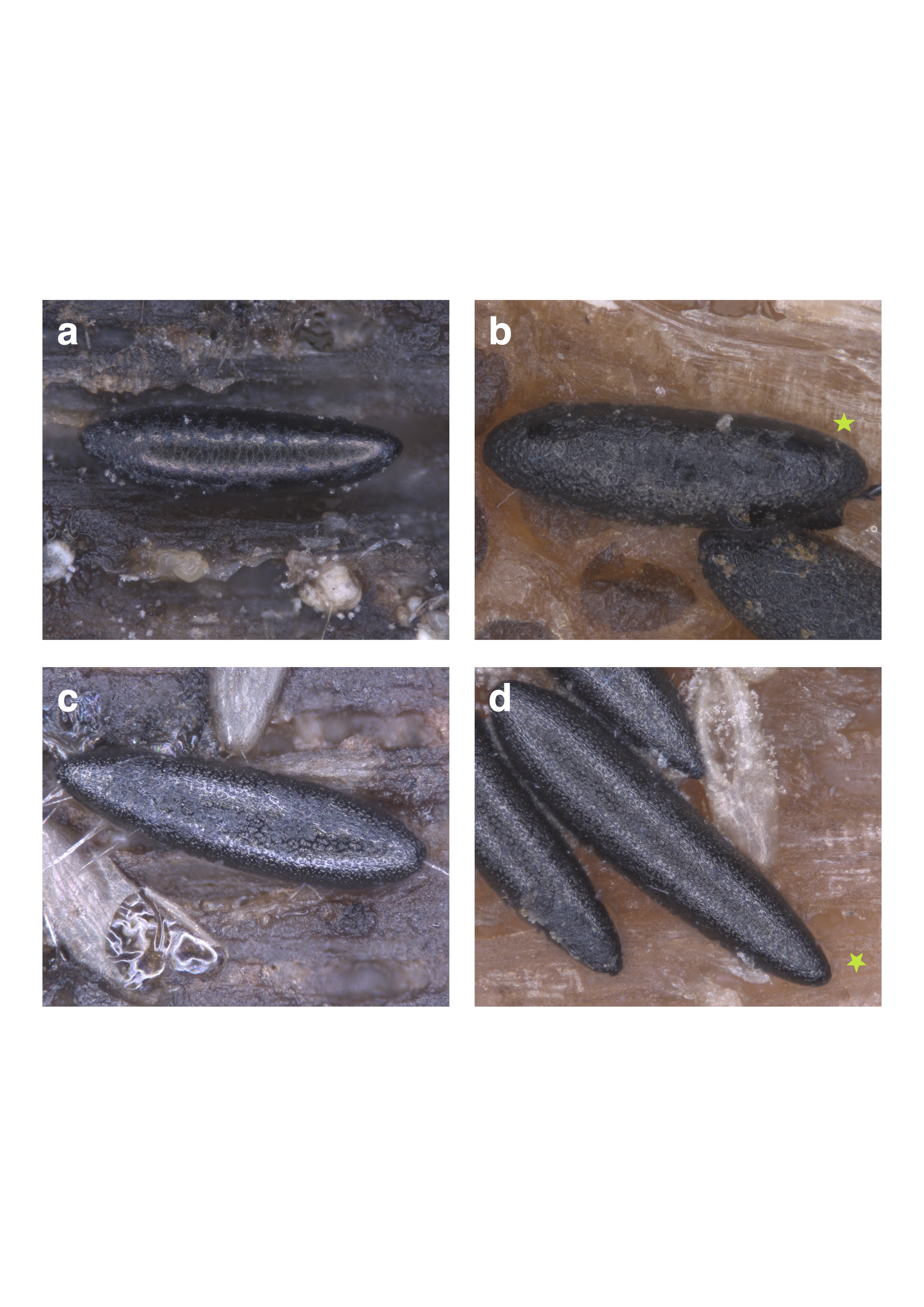

Supplement: S6 Fig — Egg of Aedes albopictus (a), Aedes geniculatus (b), Aedes japonicus (c), and Aedes koreicus (d). https://doi.org/10.6084/m9.figshare.24207957. (TIF) [file pone.0293568.s008.tif]

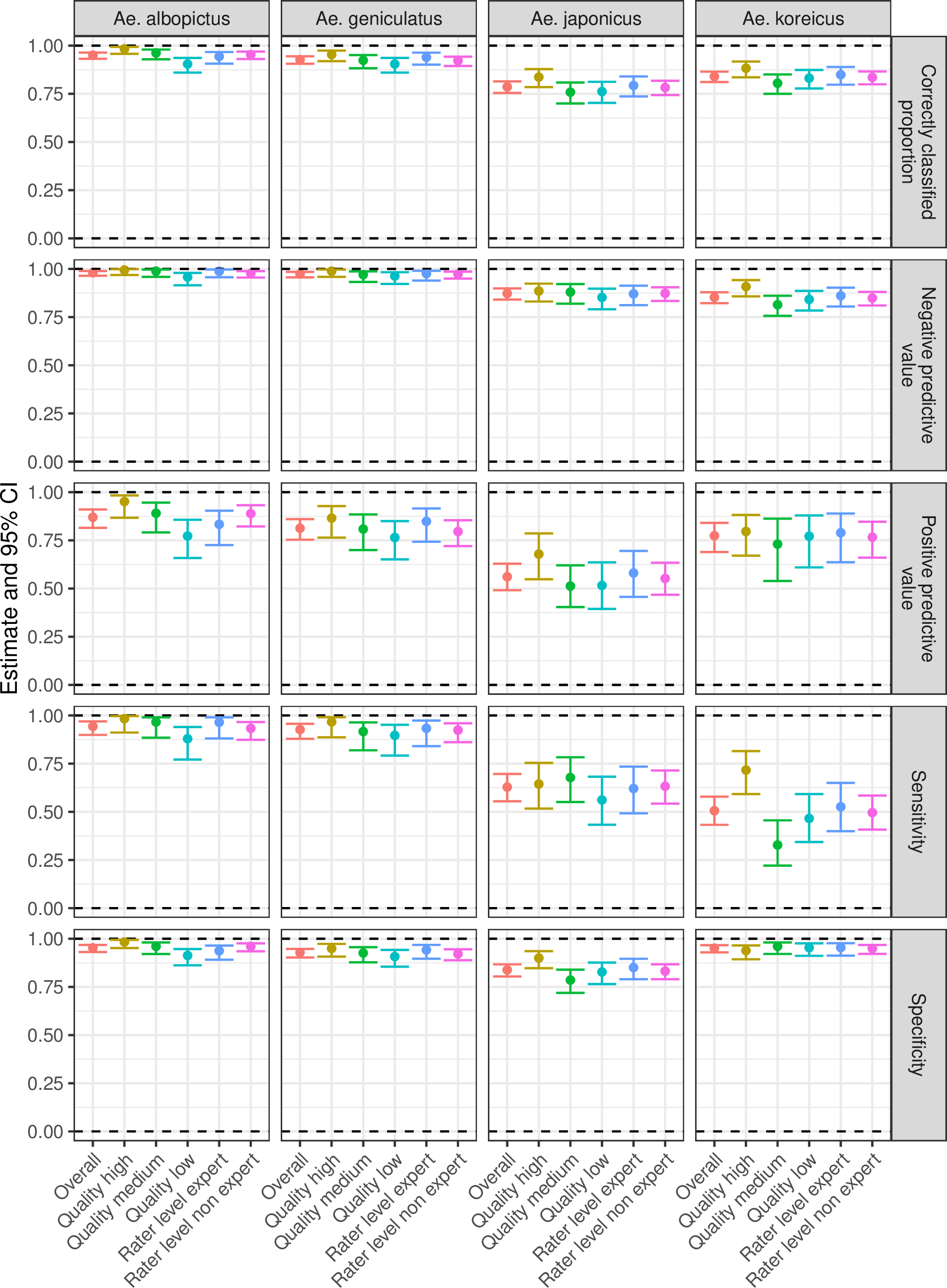

Supplement: S7 Fig — https://doi.org/10.6084/m9.figshare.24207948. (TIF) [file pone.0293568.s009.tif]

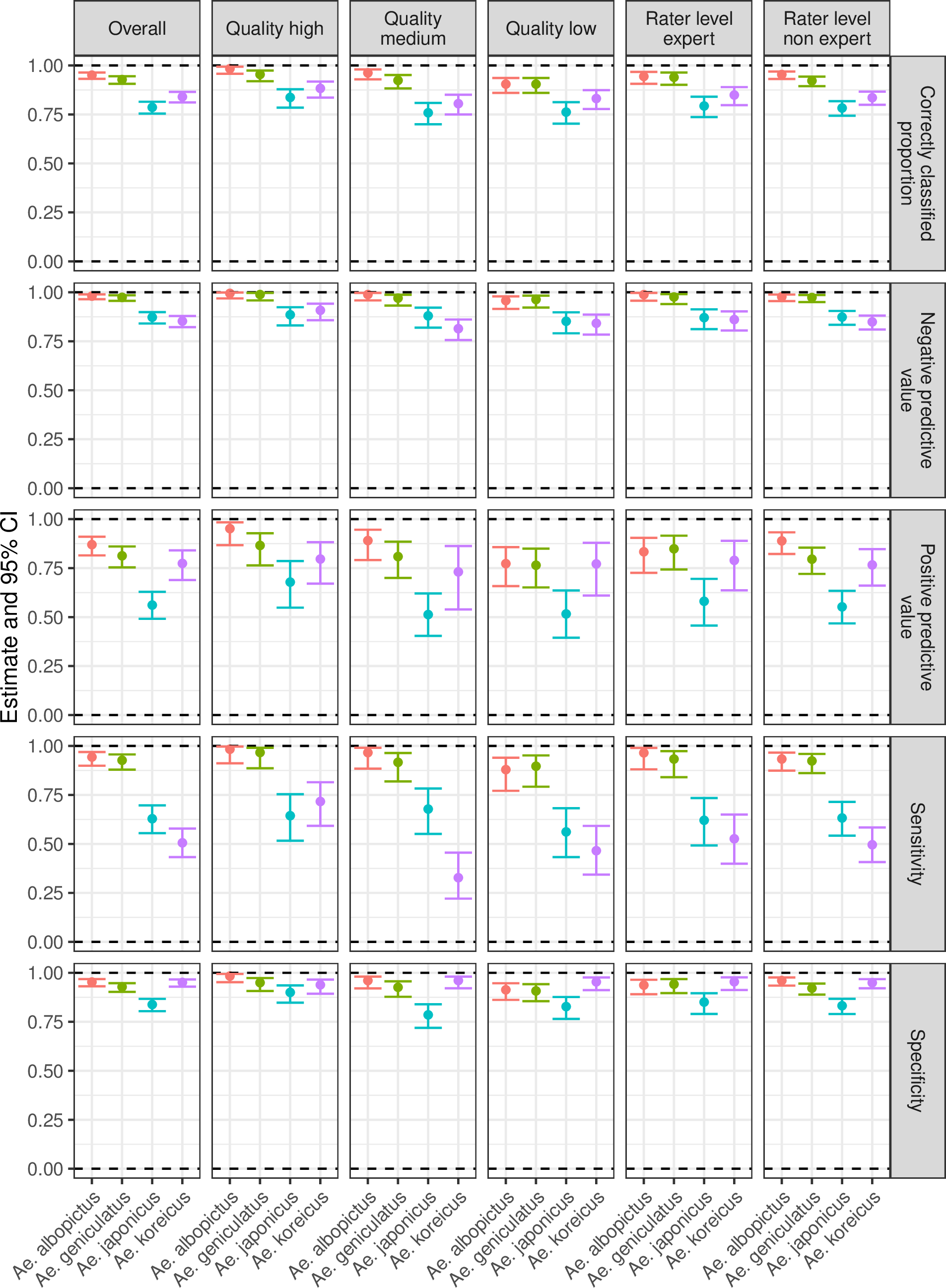

Supplement: S8 Fig — https://doi.org/10.6084/m9.figshare.24207963. (TIF) [file pone.0293568.s010.tif]

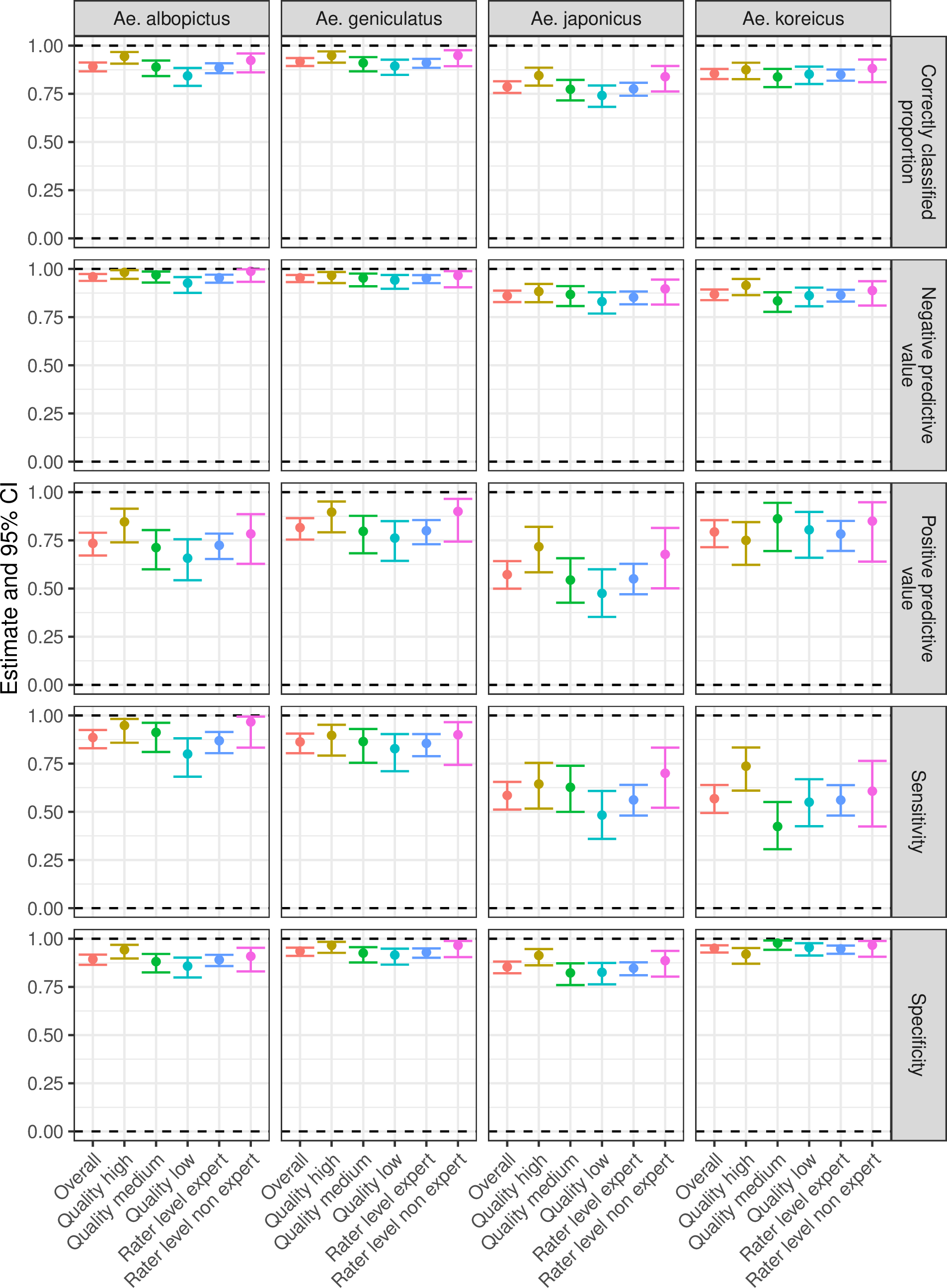

Supplement: S9 Fig — https://doi.org/10.6084/m9.figshare.24207972. (TIF) [file pone.0293568.s011.tif]

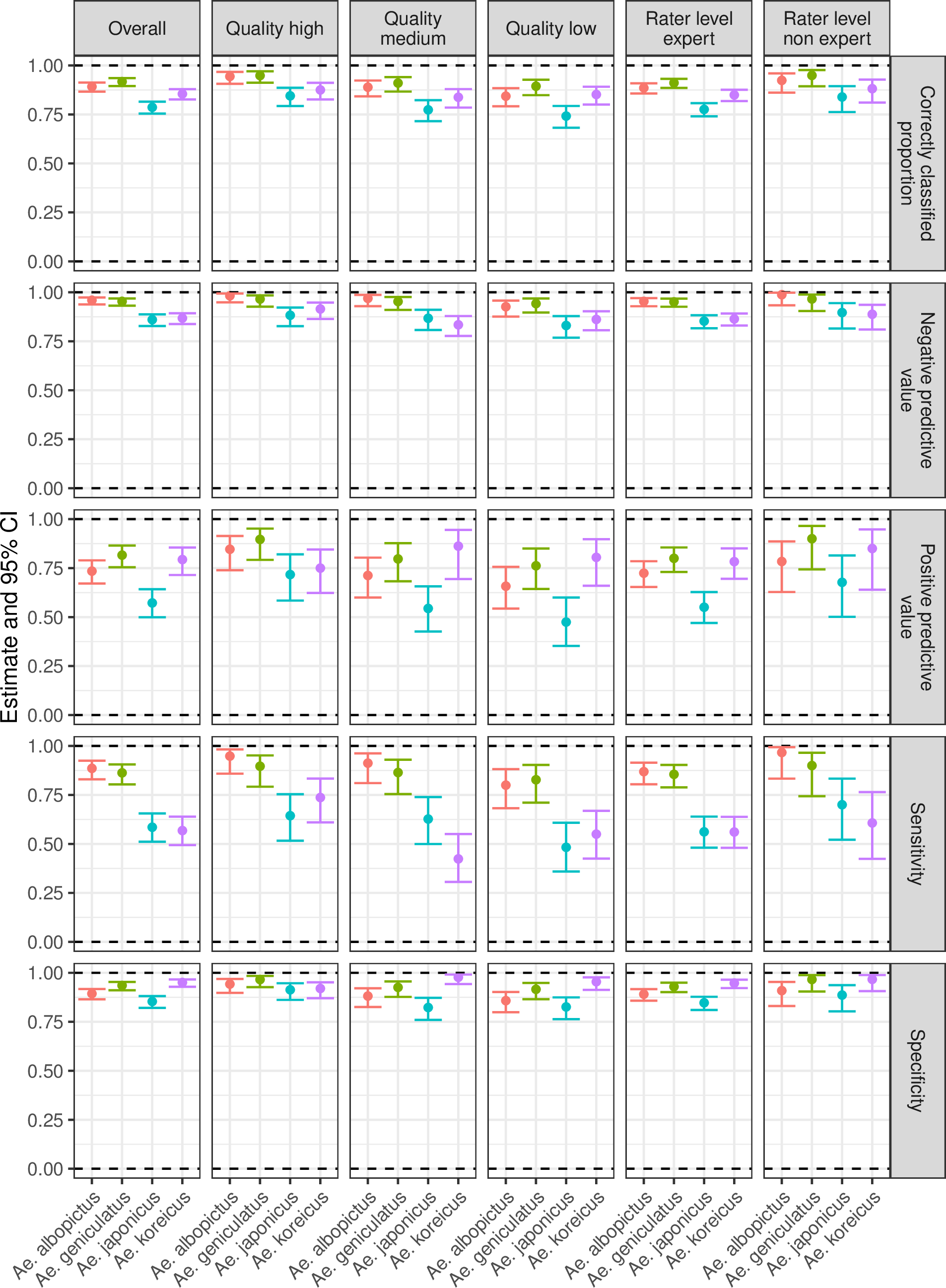

Supplement: S10 Fig — https://doi.org/10.6084/m9.figshare.24207951. (TIF) [file pone.0293568.s012.tif]

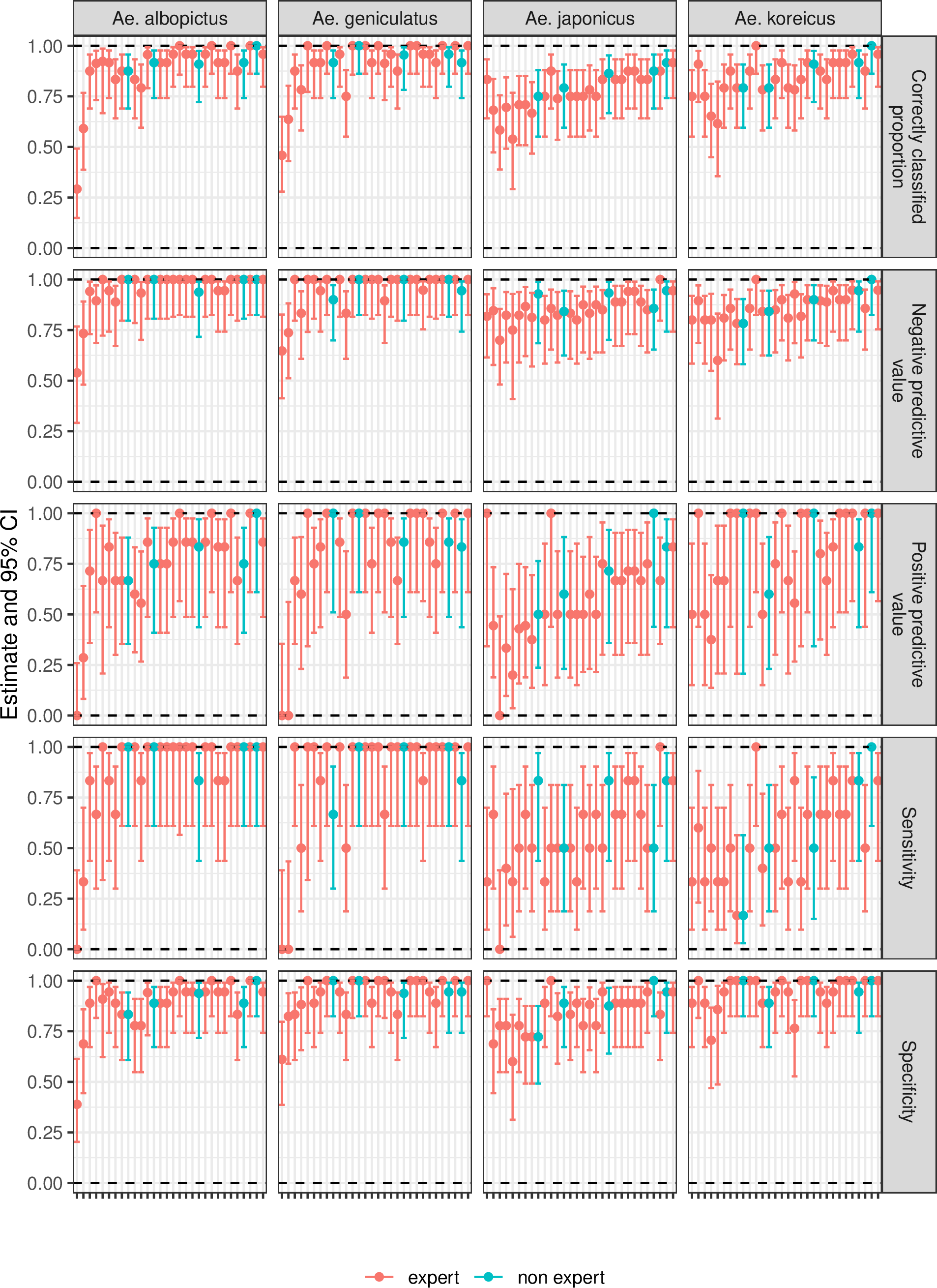

Supplement: S11 Fig — https://doi.org/10.6084/m9.figshare.24207966. (TIF) [file pone.0293568.s013.tif]
