# Supplementary material for: Optical recognition of the eggs of four Aedine mosquito species (Aedes albopictus, Aedes geniculatus, Aedes japonicus, and Aedes koreicus)
Source: PLoS One. 2023 Nov 1;18(11):e0293568. doi: 10.1371/journal.pone.0293568 (PMC10619821; doi:10.1371/journal.pone.0293568)
Supplement: S1 Table — https://doi.org/10.6084/m9.figshare.24208149.v3. (DOCX) [file pone.0293568.s014.docx]

**Table 1. Description of all diagnostic accuracy measures.** Assume that our species of interest is *Ae. albopictus.* This means that all the measures compare *Ae. albopictus* against all the others. The same definition holds for the other species too.

| Name of the accuracy measure | Description |
| --- | --- |
| Sensitivity | The probability that the rater correctly identifies *Ae. albopictus* if the image shows *Ae. albopictus* (i.e., proportion of true positives). |
| Specificity | The probability that the rater selected any of the other species (irrespective if the other species is correctly identified or not) if the image does not show *Ae. albopictus* (i.e., proportion of true negatives). |
| Correctly classified proportion / diagnostic accuracy | Proportion of correctly identifying *Ae. albopictus* or correctly identifying not being *Ae. albopictus* (irrespective if the other species is correctly identified or not) with respect to all answers. |
| Positive predictive value (PPV) | Probability that the species in the image shows *Ae. albopictus* if the answer of the rater is *Ae. albopictus.* |
| Negative predictive value (NPV) | Probability that the species in the image does not show *Ae. albopictus* if the answer of the rater is any of the other species. |

**Table 2. Test 1: estimates and 95% confidence intervals (95%CI) for additional accuracy measures for each group (overall, exochorion qualities, and levels of the rater) and each species.**

| Estimates (95%CI) | | | | | |
| --- | --- | --- | --- | --- | --- |
| Analyses | Accuracy measures | *Ae. albopictus* | *Ae. geniculatus* | *Ae. japonicus* | *Ae. koreicus* |
| Overall | Correctly classified proportion | 95.0%  (93.2-96.4) | 92.8%  (90.6-94.5) | 78.6%  (75.4-81.5) | 84.0%  (81.1-86.5) |
|  | PPV | 87.0%  (81.5-91.0) | 81.3%  (75.4-86.0) | 56.1%  (49.1-62.9) | 77.4%  (68.9-84.1) |
|  | NPV | 98.1%  (96.5-98.9) | 97.4%  (95.6-98.5) | 87.3%  (84.1-89.9) | 85.3%  (82.2-87.9) |
| High quality | Correctly classified proportion | 98.3%  (95.8-99.3) | 95.4%  (91.9-97.4) | 83.7%  (78.5to 87.8) | 88.3%  (83.6-91.8) |
|  | PPV | 95.2%  (86.7-98.3) | 86.6%  (76.4-92.8) | 67.9%  (54.8-78.6) | 79.6%  (67.1-88.2) |
|  | NPV | 99.4%  (96.9-99.9) | 98.8%  (95.9-99.7) | 88.5%  (83.1-92.4) | 90.8%  (85.8-94.2) |
| Medium quality | Correctly classified proportion | 96.2%  (92.9-98.0) | 92.4%  (88.3-95.1) | 75.8%  (70.0-80.9) | 80.5%  (75.0-85.1) |
|  | PPV | 89.1%  (79.1-94.6) | 80.9%  (70.0-88.5) | 51.3%  (40.4-62.1) | 73.1%  (53.9-86.3) |
|  | NPV | 98.8%  (95.9-99.7) | 97.0%  (93.2-98.7) | 88.0%  (82.0-92.2) | 81.4%  (75.6-86.1) |
| Low quality | Correctly classified proportion | 90.5%  (86.0-93.6) | 90.5%  (86.0-93.6) | 76.2%  (70.3-81.2) | 83.1%  (77.8-87.4) |
|  | PPV | 77.3%  (65.8-85.7) | 76.5%  (65.1-85.0) | 51.6%  (39.4-63.6) | 77.1%  (61.0-87.9) |
|  | NPV | 95.8%  (91.5-97.9) | 96.3%  (92.2-98.3) | 85.2%  (79.1-89.8) | 84.2%  (78.4-88.6) |
| Experts | Correctly classified proportion | 94.4%  (90.7-96.7) | 94.0%  (90.1-96.4) | 79.3%  (73.6-84.0) | 84.9%  (79.7-88.9) |
|  | PPV | 83.3%  (72.6-90.4) | 84.8%  (74.3-91.6) | 58.1%  (45.7-69.5) | 78.9%  (63.7-88.9) |
|  | NPV | 98.8%  (95.7-99.7) | 97.6%  (94.0-99.1) | 87.1%  (81.2-91.3) | 86.1%  (80.5-90.3) |
| Non-experts | Correctly classified proportion | 95.4%  (93.1-96.9) | 92.2%  (89.4-94.3) | 78.3%  (74.3-81.7) | 83.5%  (79.9-86.6) |
|  | PPV | 88.9%  (82.2-93.3) | 79.6%  (72.0-85.5) | 55.2%  (46.8-63.4) | 76.6%  (66.0-84.7) |
|  | NPV | 97.7%  (95.5-98.8) | 97.3%  (95.0-98.6) | 87.4%  (83.4-90.5) | 84.9%  (81.0-88.1) |

**Table 3. Test 2: Estimates and 95% confidence intervals (95%CI) for additional accuracy measures for each group (overall, exochorion qualities, and levels of the rater) and each species.**

| Estimates (95%CI) | | | | | |
| --- | --- | --- | --- | --- | --- |
| Analyses | Accuracy measures | *Ae. albopictus* | *Ae. geniculatus* | *Ae. japonicus* | *Ae. koreicus* |
| Overall | Correctly classified proportion | 89.2%  (86.7-91.3) | 91.7%  (89.5-93.6) | 78.6%  (75.4-81.5) | 85.5%  (82.7-87.9) |
|  | PPV | 73.5%  (67.1-79.0) | 81.6%  (75.4-86.5) | 57.2%  (49.9-64.2) | 79.4%  (71.5-85.5) |
|  | NPV | 95.9%  (93.8-97.3) | 95.4%  (93.2-96.9) | 86.0%  (82.8-88.7) | 86.8%  (83.8-89.3) |
| High quality | Correctly classified proportion | 94.4%  (90.7-96.7) | 94.8%  (91.2-97.0) | 84.5%  (79.3-88.6) | 87.5%  (82.6-91.2) |
|  | PPV | 84.6%  (73.9-91.4) | 89.7%  (79.2-95.2) | 71.7%  (58.4-82.0) | 75.0%  (62.3-84.5) |
|  | NPV | 98.2%  (94.9-99.4) | 96.6%  (92.7-98.4) | 88.3%  (82.7-92.2) | 91.5%  (86.4-94.8) |
| Medium quality | Correctly classified proportion | 88.9%  (84.2-92.3) | 91.0%  (86.7-94.1) | 77.4%  (71.6-82.2) | 83.8%  (78.5-87.9) |
|  | PPV | 71.2%  (60.0-80.3) | 79.7%  (68.3-87.7) | 54.4%  (42.7-65.7) | 86.2%  (69.4-94.5) |
|  | NPV | 96.9%  (92.9-98.7) | 95.3%  (91.0-97.6) | 86.7%  (80.7-91.1) | 83.4%  (77.7-87.9) |
| Low quality | Correctly classified proportion | 84.3%  (79.1-88.4) | 89.4%  (84.8-92.7) | 74.2%  (68.2-79.3) | 85.2%  (80.1-89.1) |
|  | PPV | 65.8%  (54.3-75.6) | 76.2%  (64.4-85.0) | 47.5%  (35.3-60.0) | 80.5%  (66.0-89.8) |
|  | NPV | 92.6%  (87.6-95.7) | 94.2%  (89.7-96.8) | 83.1%  (76.8-87.9) | 86.2%  (80.6-90.3) |
| Experts | Correctly classified proportion | 88.5%  (85.7-90.9) | 91.1%  (88.5-93.1) | 77.6%  (74.0-80.8) | 84.9%  (81.8-87.6) |
|  | PPV | 72.4%  (65.3-78.5) | 80.0%  (73.0-85.5) | 55.0%  (47.0-62.8) | 78.3%  (69.5-85.1) |
|  | NPV | 95.4%  (92.9-97.0) | 95.1%  (92.6-96.8) | 85.3%  (81.6-88.3) | 86.4%  (83.0-89.2) |
| Non-experts | Correctly classified proportion | 92.4%  (86.1-95.9) | 94.9%  (89.3-97.6) | 83.9%  (76.2-89.4) | 88.1%  (81.1-92.8) |
|  | PPV | 78.4%  (62.8-88.6) | 90.0%  (74.4-96.5) | 67.7%  (50.1-81.4) | 85.0%  (64.0-94.8) |
|  | NPV | 98.8%  (93.3-99.8) | 96.6%  (90.5-98.8) | 89.7%  (81.5-94.5) | 88.8%  (81.0-93.6) |
