# Supplementary material for: Optical recognition of the eggs of four Aedine mosquito species (Aedes albopictus, Aedes geniculatus, Aedes japonicus, and Aedes koreicus)
Source: PLoS One. 2023 Nov 1;18(11):e0293568. doi: 10.1371/journal.pone.0293568 (PMC10619821; doi:10.1371/journal.pone.0293568)
Supplement: S1 Text — https://doi.org/10.6084/m9.figshare.24367597.v2. (PDF) [file pone.0293568.s015.pdf]

Graphical Analysis: Discriminate Eggs Project

Workshop 2

Author: Klaus Steigmiller | Zurich Data Scientists
Reviewer: Dr. Claude Renaux | Zurich Data Scientists

October 1, 2023

Contents

1 Freezing Package versions **3**

2 Load packages **3**

3 Settings **3**

4 Statistical Methods **3**

5 Getting data **5**

6 Preparation **5**

7 Analysis **7**
7.1 We look at *Overall* . . . 8
7.1.1 Dataset . . . 8
7.1.2 Missing values . . . 8
7.1.3 Confusion Matrix . . . 8
7.1.4 Measures of diagnostic accuracy . . . 12
7.2 We look at *Quality high* . . . 16
7.2.1 Dataset . . . 16
7.2.2 Missing values . . . 16
7.2.3 Confusion Matrix . . . 16
7.2.4 Measures of diagnostic accuracy . . . 20
7.3 We look at *Quality low* . . . 24
7.3.1 Dataset . . . 24
7.3.2 Missing values . . . 24
7.3.3 Confusion Matrix . . . 24
7.3.4 Measures of diagnostic accuracy . . . 28
7.4 We look at *Quality medium* . . . 32
7.4.1 Dataset . . . 32
7.4.2 Missing values . . . 32
7.4.3 Confusion Matrix . . . 32
7.4.4 Measures of diagnostic accuracy . . . 36
7.5 We look at *Rater level expert* . . . 40
7.5.1 Dataset . . . 40
7.5.2 Missing values . . . 40
7.5.3 Confusion Matrix . . . 40
7.5.4 Measures of diagnostic accuracy . . . 44

7.6 We look at *Rater level non expert* 48
7.6.1 Dataset 48
7.6.2 Missing values 48
7.6.3 Confusion Matrix 48
7.6.4 Measures of diagnostic accuracy 52

8 Per person **56**

9 Graphical overview **68**
9.1 Compare groups (overall, quality, rater level) 68
9.2 Compare species 70
9.3 Compare rater levels 73
9.4 Compare quality levels 76
9.5 Compare per person 78

10 Time spent on rating **80**

11 Session information **82**

1 Freezing Package versions

```
## (messages are omitted in this chunk)
##
library(checkpoint)
checkpoint(snapshot_date = "2022-11-15")
```

2 Load packages

```
## (messages are omitted from this chunk)
##
library(dplyr)
library(kableExtra)
library(ggplot2)
library(epiR)
library(kableExtra) ## for displaying tables in this report
library(purrr)
library(forcats)
library(tidyr)
library(openxlsx) ## for exporting results to Excel
library(binom) ## to calculate Wilson CI for percentages
```

3 Settings

Global settings:

```
Sys.setenv(lang = "en_US")
theme_set(theme_bw())

if (!dir.exists("Prepared_data_and_models")) {
  dir.create("Prepared_data_and_models")
}
```

4 Statistical Methods

Definitions:

- Sensitivity: is probability of testing positive if the condition is really present, i.e. the proportion of true positives.
- Specificity: is probability of testing negative if the patient has not the condition, i.e. the proportion of true negatives.
- PPV (positive predictive value): is the probability of having the condition if the test is positive. This measure is affected by the prevalence of the condition.
- NPV (negative predictive value): is the probability of not having the condition if the test is negative. This measure is affected by the prevalence of the condition.
- LR+ (positive likelihood ratio): is the ratio of 'sensitivity' over '1−specificity'. It summarizes how many times it is more/less likely to have a positive test finding when having the condition compared to not having the condition.
- LR- (negative likelihood ratio): is the ratio of '1−sensitivity' over 'specificity'. It summarizes how many times it is more/less likely to have a negative test finding when having the condition compared to not having the condition
- Diagnostic accuracy: proportion of all tests that give a correct result.

- Diagnostic odds ratio is defined as how much more likely will the test make a correct diagnosis than an incorrect diagnosis in patients with the disease.
- The number needed to diagnose: the number of paitents that need to be tested to give one correct positive test.
- Youdens index is defined as 'sensitivy + specificity -1'. Youdens index ranges from -1 to $+1$ with values closer to 1 if both sensitivity and specificity are high (i.e., close to 1).

Method "wilson" was used for the confidence intervals for sensitivity, specificity, and positive and negative predictive value. Details for this method and the other choices of confidence intervals can be found on https://rdrr.io/cran/epiR/man/epi.tests.html.

Missing values are handled by *complete case analysis* (pairwise deletion of index/reference test observations).

5 Getting data

```
d.disc.eggs.l <- readRDS(file = paste0("Prepared_data_and_models/",
                                        "0_PreDataPreparationd.disc.eggs_multiple.long_workshoop2.rds"))
```

6 Preparation

We set the variable "answer.rater" as the index test. We set the variable "MALDI-TOF determination" as the reference test.

```
reference.test.chr <- "MALDI-TOF determination"
index.test.chr <- "answer.rater"
reference.vec <- d.disc.eggs.l$`MALDI-TOF determination`
index.vec <- d.disc.eggs.l$`answer.rater`
```

We first prepare the data

```
## auxiliary object to re-format output
.match <- structure(list(
  name.raw = c("ap", "tp", "se", "sp", "diag.or", "nndx",
               "youden", "pv.pos", "pv.neg", "lr.pos", "lr.neg", "p.rout", "p.rin",
               "p.tpdn", "p.tndp", "p.dntp", "p.dptn", "diag.ac"),
  Measure = c("Apparent prevalence", "True prevalence", "Sensitivity", "Specificity",
              "Diagnostic odds ratio", "Number needed to diagnose", "Youdens index",
              "Positive predictive value", "Negative predictive value",
              "Positive likelihood ratio", "Negative likelihood ratio",
              "the proportion of subjects with the outcome ruled out",
              "the proportion of subjects with the outcome ruled in",
              "False T+ proportion for trueD-", "False T- proportion for trueD+",
              "False T+ proportion for T+", "False T- proportion for T-",
              "Correctly classified proportion" ),
  bounded = c(TRUE, TRUE, TRUE, TRUE, FALSE, FALSE, FALSE, TRUE, TRUE,
              FALSE, FALSE, TRUE, TRUE, TRUE, TRUE, TRUE, TRUE, TRUE)),
  class = c("tbl_df", "tbl", "data.frame"), row.names = c(NA, -18L))
```

```
.match %>%
  kable(caption = paste0("Auxiliary coding table to assign the appropriate abbreviations to measure typ
        label = "tab:TableMatch",
        booktabs = TRUE,
        longtable = TRUE,
        linesep = c("")) %>%
  kable_styling(font_size = 7,
                latex_options = c("striped", "repeat_header", "hold_position"))
```

Table 1: Auxiliary coding table to assign the appropriate abbreviations to measure types.

name.raw	Measure	bounded
ap	Apparent prevalence	TRUE
tp	True prevalence	TRUE
se	Sensitivity	TRUE
sp	Specificity	TRUE
diag.or	Diagnostic odds ratio	FALSE
nndx	Number needed to diagnose	FALSE
youden	Youdens index	FALSE
pv.pos	Positive predictive value	TRUE

Table 1: Auxiliary coding table to assign the appropriate abbreviations to measure types. *(continued)*

name.raw	Measure	bounded
pv.neg	Negative predictive value	TRUE
lr.pos	Positive likelihood ratio	FALSE
lr.neg	Negative likelihood ratio	FALSE
p.rout	the proportion of subjects with the outcome ruled out	TRUE
p.rin	the proportion of subjects with the outcome ruled in	TRUE
p.tpdn	False T+ proportion for trueD-	TRUE
p.tndp	False T- proportion for trueD+	TRUE
p.dntp	False T+ proportion for T+	TRUE
p.dptn	False T- proportion for T-	TRUE
diag.ac	Correctly classified proportion	TRUE

Specify the number of digits to which the results should be rounded.

```
digits.rounding <- 2
```

We define some auxiliary functions to create the Confusion Matrix.

```
## function to round results
f.round <- function(x, digits.rounding = 2){
  format(x = round(x = x, digits = digits.rounding),
         scientific = FALSE,
         nsmall = digits.rounding)
}
## function to create the confusion matrix
f.create.confusion.matrix <- function(x){
  ConfusionMatrix <- cbind(x, rowSums(x))
  ConfusionMatrix <- rbind(ConfusionMatrix, colSums(ConfusionMatrix))
  colnames(ConfusionMatrix) <- c(colnames(x), "Total")
  rownames(ConfusionMatrix) <- c(rownames(x), "Total")
  return(ConfusionMatrix)
}
## function to restructure confusion matrix for printing
f.create.confusion.matrix.print <- function(x, data = c("TP", "FN", "FP", "TN")){
  ConfusionMatrix.print <- x
  ConfusionMatrix.print[1:2, 1:2] <-
    paste0(ConfusionMatrix.print[1:2, 1:2],
           " (", array(data = data, dim = c(2, 2)), ")")
  ConfusionMatrix.print
}
## auxiliary function for checking package version:
f.check.version <- function(x){
  max(grep(pattern = x, x = sort(c(x, c("2.0.50"))))) > 1
}
```

We fix/check the factor levels of index and reference test.

```
## collect all levels/categories
levels.combined <- unique(union(reference.vec, index.vec))
if_reference.vec_notfactor <- class(reference.vec) != "factor"
if_index.vec_notfactor <- class(index.vec) != "factor"
if(if_reference.vec_notfactor){
  levels.combined <- unique(union(levels.combined, levels(reference.vec)))
}
if(if_index.vec_notfactor){
```

```r
  levels.combined <- unique(union(levels.combined, levels(index.vec)))
}
levels.combined <- na.omit(levels.combined)

if(if_reference.vec_notfactor){
  reference.vec <- factor(x = reference.vec, levels = levels.combined)
}
if(if_index.vec_notfactor){
  index.vec <- factor(x = index.vec, levels = levels.combined)
}
```

```r
## relevel factor variables (needed for *True Positve*, etc.)
reference.vec <- stats::relevel(x = reference.vec, ref = "albopictus")
index.vec <- stats::relevel(x = index.vec, ref = "albopictus")

## re-assign to data.frame
d.disc.eggs.l[, "MALDI-TOF determination", drop = TRUE] <- reference.vec
d.disc.eggs.l[, "answer.rater", drop = TRUE] <- index.vec
```

7 Analysis

In this section, we calculate the confusion matrix, followed by measures of diagnostic accuracy.

First, we create lists to store the calculated objects.

```r
df.groups_list_dataset <- list()
df.groups_list_diag.measures <- list()
```

7.1 We look at *Overall*

7.1.1 Dataset

```
## Dataset
`d.disc.eggs.l_Overall` <- d.disc.eggs.l %>% filter(`group` == "Overall")
## Number of observations
(nr.obs <- nrow(`d.disc.eggs.l_Overall`))
```

```
[1] 720
```

This dataset contains 720 observations.

7.1.2 Missing values

Next, we give an overview of the missing values.

```
`d.disc.eggs.l_Overall` %>%
  select(`answer.rater`, `MALDI-TOF determination`) %>%
  table(useNA = "always")
```

```
             MALDI-TOF determination
answer.rater  albopictus geniculatus japonicus koreicus <NA>
  albopictus         155          17        32        7    0
  geniculatus         13         151        19        2    0
  japonicus            7           3       103       67    0
  koreicus             0           4        22      100    0
  <NA>                 5           5         4        4    0
```

```
## Calculate the number of missing values in both index/reference test:
nr.NA <- `d.disc.eggs.l_Overall` %>%
  select(`answer.rater`, `MALDI-TOF determination`) %>%
  (\(x){sum(is.na(x))})
## Calculate the number of observations that will be removed:
(nr.deleted.obs <- `d.disc.eggs.l_Overall` %>%
    filter(is.na(`answer.rater`) | is.na(`MALDI-TOF determination`)) %>%
    nrow())
```

```
[1] 18
```

```
`d.disc.eggs.l_Overall` <- `d.disc.eggs.l_Overall` %>%
  filter(!is.na(`answer.rater`) & !is.na(`MALDI-TOF determination`))
```

There were 18 observations (rows) deleted due to missing values.

7.1.3 Confusion Matrix

We calculate the confusion matrices in the following steps.

```
tab <- xtabs(formula = ~ `answer.rater` + `MALDI-TOF determination`,
             data = `d.disc.eggs.l_Overall`)
tab %>%
  f.create.confusion.matrix() %>%
  as_tibble(rownames = "answer.rater") %>%
  kable(caption = paste0("Overall cross table."),
        label = "tab:CrossTable1",
```

```
        booktabs = TRUE,
        longtable = TRUE,
        linesep = c("")) %>%
  kable_styling(font_size = 7,
                latex_options = c("striped", "repeat_header", "hold_position")) %>%
  row_spec(row = 5, bold = TRUE) %>%
  column_spec(column = 6, bold = TRUE) %>%
  add_header_above(c("", "MALDI-TOF determination" = 4, ""))
```

Table 2: Overall cross table.

	MALDI-TOF determination				
answer.rater	albopictus	geniculatus	japonicus	koreicus	Total
albopictus	155	17	32	7	**211**
geniculatus	13	151	19	2	**185**
japonicus	7	3	103	67	**180**
koreicus	0	4	22	100	**126**
**Total**	**175**	**175**	**176**	**176**	**702**

Example how to read this table:

- There were 155 cases of *albopictus* that were correctly classified as *albopictus*, 13 cases of *albopictus* were wrongly classified as *geniculatus*.
- There were 151 cases of *geniculatus* that were correctly classified as *geniculatus*, 17 cases of *geniculatus* were wrongly classified as *albopictus*.

We present the same table in percentages. Note that all columns add to 100%.

```
tab <- xtabs(formula = ~ `answer.rater` + `MALDI-TOF determination`,
             data = `d.disc.eggs.l_Overall`)
prop.table(x = tab, margin = 2) %>%
  '*'(100) %>%
  rbind(., colSums(.)) %>%
  f.round(digits = digits.rounding) %>%
  kable(caption = paste0("Overall cross table in percentages. All columns add up to 100\\%."),
        label = "tab:CrossTablePercentage1",
        booktabs = TRUE,
        longtable = TRUE,
        linesep = c("")) %>%
  kable_styling(font_size = 7,
                latex_options = c("striped", "repeat_header", "hold_position")) %>%
  row_spec(row = 5, bold = TRUE) %>%
  add_header_above(c("", "MALDI-TOF determination" = 4))
```

Table 3: Overall cross table in percentages. All columns add up to 100%.

	MALDI-TOF determination			
	albopictus	geniculatus	japonicus	koreicus
albopictus	88.57	9.71	18.18	3.98
geniculatus	7.43	86.29	10.80	1.14
japonicus	4.00	1.71	58.52	38.07
koreicus	0.00	2.29	12.50	56.82
	**100.00**	**100.00**	**100.00**	**100.00**

```
levels.chr <- levels(`d.disc.eggs.l_Overall`$`answer.rater`)
if(length(levels.chr) == 2){
```

```
  classes <- c("")
}else{
  classes <- levels.chr
}

## calculate tables
if(identical(classes, c(""))){
  l.tab <- list(xtabs(formula = ~ `answer.rater` + `MALDI-TOF determination`,
                      data = `d.disc.eggs.l_Overall`))
}else{
  l.tab <- lapply(X = classes, FUN = function(x){
    xtabs(formula = ~ `answer.rater` + `MALDI-TOF determination`,
          data = `d.disc.eggs.l_Overall` %>%
            mutate(across(.cols = c(`answer.rater`, `MALDI-TOF determination`), .fns = function(y){
              ## we merge categories into the category "neg."
              y %>%
                fct_collapse("neg." = setdiff(levels(`d.disc.eggs.l_Overall`$`answer.rater`), x)) %>%
                fct_relevel(x)
            })))
  })
}

## calculate confusion matrix
l.confusion.matrix <- lapply(X = l.tab,
                             FUN = f.create.confusion.matrix)
## calculate tables for printing
l.confusion.matrix.print <- lapply(X = l.confusion.matrix,
                                   FUN = f.create.confusion.matrix.print)
```

The next tables show the confusion matrices for the index test ("answer.rater") against the reference test ("MALDI-TOF determination").

```
for(class in seq_along(classes)){
  l.confusion.matrix.print[[class]] %>%
    as_tibble(rownames = "answer.rater") %>%
    kable(caption = paste0("Confusion matrix for class ‘",
                           levels(`d.disc.eggs.l_Overall`$`answer.rater`)[class], "’.
          Category ‘", levels(`d.disc.eggs.l_Overall`$`answer.rater`)[class],
          "’ is considered a positive result.
          Category ‘neg.’ is considered a negative result and
          contains the levels ",
          paste0(setdiff(levels(`d.disc.eggs.l_Overall`$`answer.rater`),
                         classes[class]), collapse = ", "), "."),
          label = paste0("tab:ConfusionMatrix1Class", class),
          booktabs = TRUE,
          longtable = TRUE,
          linesep = c("")) %>%
    kable_styling(font_size = 7,
                  latex_options = c("striped", "repeat_header", "hold_position")) %>%
    row_spec(row = nrow(l.confusion.matrix.print[[class]]), bold = TRUE) %>%
    column_spec(column = ncol(l.confusion.matrix.print[[class]]) + 1, bold = TRUE) %>%
    add_header_above(c("", "MALDI-TOF determination" =
                         ncol(l.confusion.matrix.print[[class]]) - 1, "")) %>%
```

```
    print()
}
```

Table 4: Confusion matrix for class 'albopictus'. Category 'albopictus' is considered a positive result. Category 'neg.' is considered a negative result and contains the levels geniculatus, japonicus, koreicus.

	MALDI-TOF determination		
answer.rater	albopictus	neg.	Total
albopictus	155 (TP)	56 (FP)	**211**
neg.	20 (FN)	471 (TN)	**491**
**Total**	**175**	**527**	**702**

Table 5: Confusion matrix for class 'geniculatus'. Category 'geniculatus' is considered a positive result. Category 'neg.' is considered a negative result and contains the levels albopictus, japonicus, koreicus.

	MALDI-TOF determination		
answer.rater	geniculatus	neg.	Total
geniculatus	151 (TP)	34 (FP)	**185**
neg.	24 (FN)	493 (TN)	**517**
**Total**	**175**	**527**	**702**

Table 6: Confusion matrix for class 'japonicus'. Category 'japonicus' is considered a positive result. Category 'neg.' is considered a negative result and contains the levels albopictus, geniculatus, koreicus.

	MALDI-TOF determination		
answer.rater	japonicus	neg.	Total
japonicus	103 (TP)	77 (FP)	**180**
neg.	73 (FN)	449 (TN)	**522**
**Total**	**176**	**526**	**702**

Table 7: Confusion matrix for class 'koreicus'. Category 'koreicus' is considered a positive result. Category 'neg.' is considered a negative result and contains the levels albopictus, geniculatus, japonicus.

	MALDI-TOF determination		
answer.rater	koreicus	neg.	Total
koreicus	100 (TP)	26 (FP)	**126**
neg.	76 (FN)	500 (TN)	**576**
**Total**	**176**	**526**	**702**

7.1.4 Measures of diagnostic accuracy

We now calculate measures of diagnostic accuracy.

```
res.diagnostic.measures <- tibble("Class" = classes) %>%
  mutate(tabs = l.tab) %>%

  ## epiR wants it in the format: c(TP, FP, FN, TN)
  mutate(confusion.vector = map(.x = tabs, .f = function(x){
    c("tp" = x[1, 1], "fp" = x[1, 2], "fn" = x[2, 1], "tn" = x[2, 2])
  })) %>%

  ## Calculate measures of diagnostic accuracy
  mutate(out.EpiR = map(.x = confusion.vector, .f = function(x){
    epi.tests(dat = x,
              method = "wilson",
              conf.level = 0.95)
  })) %>%

  ## reformat output, filter diag. measures of interest
  mutate(diag.measures = map(.x = out.EpiR, .f = function(x){
    x <- x %>%
      summary() %>%
      as_tibble(rownames = "name.raw") %>%
      mutate()
    if(f.check.version(x = packageVersion(pkg = "epiR"))){
      x <- x %>%
        mutate(name.raw = statistic) %>%
        select(-statistic)
    }
    x %>%
      left_join(y = .match, by = c("name.raw")) %>%
      filter(name.raw %in% c("ap", "tp", "se", "sp", "diag.or", "pv.pos", "pv.neg", "lr.pos", "lr.neg",
  })) %>%

  ## ensure boundaries of estimates and CI
  mutate(diag.measures = map(.x = diag.measures, .f = function(x){
    x %>%
      mutate(across(.cols = c(est, lower, upper), .fns = function(x){
        if_else(condition = bounded, true = pmin(1, pmax(0, x)), false = x)
      }))
  })) %>%

  ## put together output
  mutate(diag.measures.print = map(.x = diag.measures, .f = function(x){
    x %>%
      mutate(across(.cols = c("est", "lower", "upper"),
                    .fns = \(x){format(x = round(x = x, digits = digits.rounding + 1),
                                       scientific = FALSE,
                                       nsmall = digits.rounding + 1)})) %>%
      mutate("Estimate (95% CI)" = paste0(est, " (from ", lower, " to ", upper, ")")) %>%
      select(Measure, `Estimate (95% CI)`)
  }))
```

We restructure the results that are to be printed.

```
aux.df.rows <- res.diagnostic.measures %>%
  select(Class, diag.measures.print) %>%
  mutate(Class = paste0("Class ", Class)) %>%
  unnest(cols = diag.measures.print) %>%
  mutate(row.nr = seq_len(n())) %>%
  group_by(Class) %>%
  summarize(min = min(row.nr),
            max = max(row.nr)) %>%
  ungroup()
```

We prepare the printing of the table.

```
res.diagnostic.measures_print1 <- res.diagnostic.measures %>%
  select(Class, diag.measures.print) %>%
  unnest(cols = diag.measures.print) %>%
  select(-Class) %>%

  kable(caption = paste0("Estimate and 95\\% confidence intervals for prevalence
                         and different measures of accuracy."),
        label = "tab:ResultTable1",
        booktabs = TRUE,
        longtable = TRUE,
        linesep = c("")) %>%
  kable_styling(
    font_size = 7,
    repeat_header_method = "replace",
    repeat_header_text = paste0("Estimate and 95\\% confidence intervals for prevalence
                                and different measures of accuracy. (continued)"),
    latex_options = c("striped", "repeat_header", "hold_position")
  ) %>%

  group_rows(group_label = aux.df.rows$Class[1],
             start_row = aux.df.rows$min[1],
             end_row = aux.df.rows$max[1]) %>%
  group_rows(group_label = aux.df.rows$Class[2],
             start_row = aux.df.rows$min[2],
             end_row = aux.df.rows$max[2]) %>%
  group_rows(group_label = aux.df.rows$Class[3],
             start_row = aux.df.rows$min[3],
             end_row = aux.df.rows$max[3]) %>%
  group_rows(group_label = aux.df.rows$Class[4],
             start_row = aux.df.rows$min[4],
             end_row = aux.df.rows$max[4]) %>%
  collapse_rows(columns = 1, latex_hline = "major", valign = "middle")
```

The next table shows point estimate and 95% confidence intervals for prevalence and different measures of diagnostic accuracy.

```
res.diagnostic.measures_print1
```

Table 8: Estimate and 95% confidence intervals for prevalence and different measures of accuracy.

Measure	Estimate (95% CI)
**Class albopictus**	
Apparent prevalence	0.301 (from 0.268 to 0.335)

Table 8: Estimate and 95% confidence intervals for prevalence and different measures of accuracy. (continued)

Measure	Estimate (95% CI)
True prevalence	0.249 (from 0.219 to 0.283)
Sensitivity	0.886 (from 0.830 to 0.925)
Specificity	0.894 (from 0.865 to 0.917)
Correctly classified proportion	0.892 (from 0.867 to 0.913)
Diagnostic odds ratio	65.183 (from 37.915 to 112.063)
Positive predictive value	0.735 (from 0.671 to 0.790)
Negative predictive value	0.959 (from 0.938 to 0.973)
Positive likelihood ratio	8.335 (from 6.470 to 10.738)
Negative likelihood ratio	0.128 (from 0.085 to 0.193)
**Class geniculatus**	
Apparent prevalence	0.264 (from 0.232 to 0.297)
True prevalence	0.249 (from 0.219 to 0.283)
Sensitivity	0.863 (from 0.804 to 0.906)
Specificity	0.935 (from 0.911 to 0.953)
Correctly classified proportion	0.917 (from 0.895 to 0.936)
Diagnostic odds ratio	91.229 (from 52.455 to 158.665)
Positive predictive value	0.816 (from 0.754 to 0.865)
Negative predictive value	0.954 (from 0.932 to 0.969)
Positive likelihood ratio	13.374 (from 9.611 to 18.611)
Negative likelihood ratio	0.147 (from 0.101 to 0.213)
**Class japonicus**	
Apparent prevalence	0.256 (from 0.225 to 0.290)
True prevalence	0.251 (from 0.220 to 0.284)
Sensitivity	0.585 (from 0.511 to 0.655)
Specificity	0.854 (from 0.821 to 0.881)
Correctly classified proportion	0.786 (from 0.754 to 0.815)
Diagnostic odds ratio	8.228 (from 5.597 to 12.093)
Positive predictive value	0.572 (from 0.499 to 0.642)
Negative predictive value	0.860 (from 0.828 to 0.887)
Positive likelihood ratio	3.998 (from 3.142 to 5.087)
Negative likelihood ratio	0.486 (from 0.406 to 0.581)
**Class koreicus**	
Apparent prevalence	0.179 (from 0.153 to 0.210)
True prevalence	0.251 (from 0.220 to 0.284)
Sensitivity	0.568 (from 0.494 to 0.639)
Specificity	0.951 (from 0.929 to 0.966)
Correctly classified proportion	0.855 (from 0.827 to 0.879)
Diagnostic odds ratio	25.304 (from 15.434 to 41.484)
Positive predictive value	0.794 (from 0.715 to 0.855)
Negative predictive value	0.868 (from 0.838 to 0.893)
Positive likelihood ratio	11.495 (from 7.734 to 17.084)
Negative likelihood ratio	0.454 (from 0.383 to 0.539)

We save the created objects in a list.

```
df.groups_list_dataset[["Overall"]] <- `d.disc.eggs.l_Overall`
df.groups_list_diag.measures[["Overall"]] <- res.diagnostic.measures
```

We clean up the programming environment.

```
rm(tab, nr.obs, l.confusion.matrix,
   l.confusion.matrix.print, res.diagnostic.measures,
   aux.df.rows)
```

7.2 We look at *Quality high*

7.2.1 Dataset

```
## Dataset
`d.disc.eggs.l_Quality high` <- d.disc.eggs.l %>% filter(`group` == "Quality high")
## Number of observations
(nr.obs <- nrow(`d.disc.eggs.l_Quality high`))
```

```
[1] 240
```

This dataset contains 240 observations.

7.2.2 Missing values

Next, we give an overview of the missing values.

```
`d.disc.eggs.l_Quality high` %>%
  select(`answer.rater`, `MALDI-TOF determination`) %>%
  table(useNA = "always")
```

```
             MALDI-TOF determination
answer.rater  albopictus geniculatus japonicus koreicus <NA>
  albopictus          55           5         4        1    0
  geniculatus          2          52         4        0    0
  japonicus            1           0        38       14    0
  koreicus             0           1        13       42    0
  <NA>                 2           2         1        3    0
```

```
## Calculate the number of missing values in both index/reference test:
nr.NA <- `d.disc.eggs.l_Quality high` %>%
  select(`answer.rater`, `MALDI-TOF determination`) %>%
  (\(x){sum(is.na(x))})
## Calculate the number of observations that will be removed:
(nr.deleted.obs <- `d.disc.eggs.l_Quality high` %>%
    filter(is.na(`answer.rater`) | is.na(`MALDI-TOF determination`)) %>%
    nrow())
```

```
[1] 8
```

```
`d.disc.eggs.l_Quality high` <- `d.disc.eggs.l_Quality high` %>%
  filter(!is.na(`answer.rater`) & !is.na(`MALDI-TOF determination`))
```

There were 8 observations (rows) deleted due to missing values.

7.2.3 Confusion Matrix

We calculate the confusion matrices in the following steps.

```
tab <- xtabs(formula = ~ `answer.rater` + `MALDI-TOF determination`,
             data = `d.disc.eggs.l_Quality high`)
tab %>%
  f.create.confusion.matrix() %>%
  as_tibble(rownames = "answer.rater") %>%
  kable(caption = paste0("Overall cross table."),
        label = "tab:CrossTable2",
```

```
         booktabs = TRUE,
         longtable = TRUE,
         linesep = c("")) %>%
  kable_styling(font_size = 7,
                latex_options = c("striped", "repeat_header", "hold_position")) %>%
  row_spec(row = 5, bold = TRUE) %>%
  column_spec(column = 6, bold = TRUE) %>%
  add_header_above(c("", "MALDI-TOF determination" = 4, ""))
```

Table 9: Overall cross table.

	MALDI-TOF determination				
answer.rater	albopictus	geniculatus	japonicus	koreicus	Total
albopictus	55	5	4	1	**65**
geniculatus	2	52	4	0	**58**
japonicus	1	0	38	14	**53**
koreicus	0	1	13	42	**56**
**Total**	**58**	**58**	**59**	**57**	**232**

Example how to read this table:

- There were 55 cases of *albopictus* that were correctly classified as *albopictus*, 2 cases of *albopictus* were wrongly classified as *geniculatus*.
- There were 52 cases of *geniculatus* that were correctly classified as *geniculatus*, 5 cases of *geniculatus* were wrongly classified as *albopictus*.

We present the same table in percentages. Note that all columns add to 100%.

```
tab <- xtabs(formula = ~ `answer.rater` + `MALDI-TOF determination`,
             data = `d.disc.eggs.l_Quality high`)
prop.table(x = tab, margin = 2) %>%
  '*'(100) %>%
  rbind(., colSums(.)) %>%
  f.round(digits = digits.rounding) %>%
  kable(caption = paste0("Overall cross table in percentages. All columns add up to 100\\%."),
        label = "tab:CrossTablePercentage2",
        booktabs = TRUE,
        longtable = TRUE,
        linesep = c("")) %>%
  kable_styling(font_size = 7,
                latex_options = c("striped", "repeat_header", "hold_position")) %>%
  row_spec(row = 5, bold = TRUE) %>%
  add_header_above(c("", "MALDI-TOF determination" = 4))
```

Table 10: Overall cross table in percentages. All columns add up to 100%.

	MALDI-TOF determination			
	albopictus	geniculatus	japonicus	koreicus
albopictus	94.83	8.62	6.78	1.75
geniculatus	3.45	89.66	6.78	0.00
japonicus	1.72	0.00	64.41	24.56
koreicus	0.00	1.72	22.03	73.68
	**100.00**	**100.00**	**100.00**	**100.00**

```
levels.chr <- levels(`d.disc.eggs.l_Quality high`$`answer.rater`)
if(length(levels.chr) == 2){
```

```
  classes <- c("")
}else{
  classes <- levels.chr
}

## calculate tables
if(identical(classes, c(""))){
  l.tab <- list(xtabs(formula = ~ `answer.rater` + `MALDI-TOF determination`,
                      data = `d.disc.eggs.l_Quality high`))
}else{
  l.tab <- lapply(X = classes, FUN = function(x){
    xtabs(formula = ~ `answer.rater` + `MALDI-TOF determination`,
          data = `d.disc.eggs.l_Quality high` %>%
            mutate(across(.cols = c(`answer.rater`, `MALDI-TOF determination`), .fns = function(y){
              ## we merge categories into the category "neg."
              y %>%
                fct_collapse("neg." = setdiff(levels(`d.disc.eggs.l_Quality high`$`answer.rater`), x)) [illegible]
                fct_relevel(x)
            })))
  })
}
## calculate confusion matrix
l.confusion.matrix <- lapply(X = l.tab,
                             FUN = f.create.confusion.matrix)
## calculate tables for printing
l.confusion.matrix.print <- lapply(X = l.confusion.matrix,
                                   FUN = f.create.confusion.matrix.print)
```

The next tables show the confusion matrices for the index test ("answer.rater") against the reference test ("MALDI-TOF determination").

```
for(class in seq_along(classes)){
  l.confusion.matrix.print[[class]] %>%
    as_tibble(rownames = "answer.rater") %>%
    kable(caption = paste0("Confusion matrix for class ‘",
                           levels(`d.disc.eggs.l_Quality high`$`answer.rater`)[class], "’.
          Category ‘", levels(`d.disc.eggs.l_Quality high`$`answer.rater`)[class],
          "’ is considered a positive result.
          Category ‘neg.’ is considered a negative result and
          contains the levels ",
          paste0(setdiff(levels(`d.disc.eggs.l_Quality high`$`answer.rater`),
                         classes[class]), collapse = ", "), "."),
          label = paste0("tab:ConfusionMatrix2Class", class),
          booktabs = TRUE,
          longtable = TRUE,
          linesep = c("")) %>%
    kable_styling(font_size = 7,
                  latex_options = c("striped", "repeat_header", "hold_position")) %>%
    row_spec(row = nrow(l.confusion.matrix.print[[class]]), bold = TRUE) %>%
    column_spec(column = ncol(l.confusion.matrix.print[[class]]) + 1, bold = TRUE) %>%
    add_header_above(c("", "MALDI-TOF determination" =
                         ncol(l.confusion.matrix.print[[class]]) - 1, "")) %>%
    print()
```

```
}
```

Table 11: Confusion matrix for class ‘albopictus’. Category ‘albopictus’ is considered a positive result. Category ‘neg.’ is considered a negative result and contains the levels geniculatus, japonicus, koreicus.

	MALDI-TOF determination		
answer.rater	albopictus	neg.	Total
albopictus	55 (TP)	10 (FP)	**65**
neg.	3 (FN)	164 (TN)	**167**
**Total**	**58**	**174**	**232**

Table 12: Confusion matrix for class ‘geniculatus’. Category ‘geniculatus’ is considered a positive result. Category ‘neg.’ is considered a negative result and contains the levels albopictus, japonicus, koreicus.

	MALDI-TOF determination		
answer.rater	geniculatus	neg.	Total
geniculatus	52 (TP)	6 (FP)	**58**
neg.	6 (FN)	168 (TN)	**174**
**Total**	**58**	**174**	**232**

Table 13: Confusion matrix for class ‘japonicus’. Category ‘japonicus’ is considered a positive result. Category ‘neg.’ is considered a negative result and contains the levels albopictus, geniculatus, koreicus.

	MALDI-TOF determination		
answer.rater	japonicus	neg.	Total
japonicus	38 (TP)	15 (FP)	**53**
neg.	21 (FN)	158 (TN)	**179**
**Total**	**59**	**173**	**232**

Table 14: Confusion matrix for class ‘koreicus’. Category ‘koreicus’ is considered a positive result. Category ‘neg.’ is considered a negative result and contains the levels albopictus, geniculatus, japonicus.

	MALDI-TOF determination		
answer.rater	koreicus	neg.	Total
koreicus	42 (TP)	14 (FP)	**56**
neg.	15 (FN)	161 (TN)	**176**
**Total**	**57**	**175**	**232**

7.2.4 Measures of diagnostic accuracy

We now calculate measures of diagnostic accuracy.

```
res.diagnostic.measures <- tibble("Class" = classes) %>%
  mutate(tabs = l.tab) %>%

  ## epiR wants it in the format: c(TP, FP, FN, TN)
  mutate(confusion.vector = map(.x = tabs, .f = function(x){
    c("tp" = x[1, 1], "fp" = x[1, 2], "fn" = x[2, 1], "tn" = x[2, 2])
  })) %>%

  ## Calculate measures of diagnostic accuracy
  mutate(out.EpiR = map(.x = confusion.vector, .f = function(x){
    epi.tests(dat = x,
              method = "wilson",
              conf.level = 0.95)
  })) %>%

  ## reformat output, filter diag. measures of interest
  mutate(diag.measures = map(.x = out.EpiR, .f = function(x){
    x <- x %>%
      summary() %>%
      as_tibble(rownames = "name.raw") %>%
      mutate()
    if(f.check.version(x = packageVersion(pkg = "epiR"))){
      x <- x %>%
        mutate(name.raw = statistic) %>%
        select(-statistic)
    }
    x %>%
      left_join(y = .match, by = c("name.raw")) %>%
      filter(name.raw %in% c("ap", "tp", "se", "sp", "diag.or", "pv.pos", "pv.neg", "lr.pos", "lr.neg",
  })) %>%

  ## ensure boundaries of estimates and CI
  mutate(diag.measures = map(.x = diag.measures, .f = function(x){
    x %>%
      mutate(across(.cols = c(est, lower, upper), .fns = function(x){
        if_else(condition = bounded, true = pmin(1, pmax(0, x)), false = x)
      }))
  })) %>%

  ## put together output
  mutate(diag.measures.print = map(.x = diag.measures, .f = function(x){
    x %>%
      mutate(across(.cols = c("est", "lower", "upper"),
                    .fns = \(x){format(x = round(x = x, digits = digits.rounding + 1),
                                       scientific = FALSE,
                                       nsmall = digits.rounding + 1)})) %>%
      mutate("Estimate (95% CI)" = paste0(est, " (from ", lower, " to ", upper, ")")) %>%
      select(Measure, `Estimate (95% CI)`)
  }))
```

We restructure the results that are to be printed.

```r
aux.df.rows <- res.diagnostic.measures %>%
  select(Class, diag.measures.print) %>%
  mutate(Class = paste0("Class ", Class)) %>%
  unnest(cols = diag.measures.print) %>%
  mutate(row.nr = seq_len(n())) %>%
  group_by(Class) %>%
  summarize(min = min(row.nr),
            max = max(row.nr)) %>%
  ungroup()
```

We prepare the printing of the table.

```r
res.diagnostic.measures_print2 <- res.diagnostic.measures %>%
  select(Class, diag.measures.print) %>%
  unnest(cols = diag.measures.print) %>%
  select(-Class) %>%

  kable(caption = paste0("Estimate and 95\\% confidence intervals for prevalence
                         and different measures of accuracy."),
        label = "tab:ResultTable2",
        booktabs = TRUE,
        longtable = TRUE,
        linesep = c("")) %>%
  kable_styling(
    font_size = 7,
    repeat_header_method = "replace",
    repeat_header_text = paste0("Estimate and 95\\% confidence intervals for prevalence
                                and different measures of accuracy. (continued)"),
    latex_options = c("striped", "repeat_header", "hold_position")
  ) %>%

  group_rows(group_label = aux.df.rows$Class[1],
             start_row = aux.df.rows$min[1],
             end_row = aux.df.rows$max[1]) %>%
  group_rows(group_label = aux.df.rows$Class[2],
             start_row = aux.df.rows$min[2],
             end_row = aux.df.rows$max[2]) %>%
  group_rows(group_label = aux.df.rows$Class[3],
             start_row = aux.df.rows$min[3],
             end_row = aux.df.rows$max[3]) %>%
  group_rows(group_label = aux.df.rows$Class[4],
             start_row = aux.df.rows$min[4],
             end_row = aux.df.rows$max[4]) %>%
  collapse_rows(columns = 1, latex_hline = "major", valign = "middle")
```

The next table shows point estimate and 95% confidence intervals for prevalence and different measures of diagnostic accuracy.

```r
res.diagnostic.measures_print2
```

Table 15: Estimate and 95% confidence intervals for prevalence and different measures of accuracy.

Measure	Estimate (95% CI)
**Class albopictus**	
Apparent prevalence	0.280 (from 0.226 to 0.341)

Table 15: Estimate and 95% confidence intervals for prevalence and different measures of accuracy. (continued)

Measure	Estimate (95% CI)
True prevalence	0.250 (from 0.199 to 0.309)
Sensitivity	0.948 (from 0.859 to 0.982)
Specificity	0.943 (from 0.897 to 0.968)
Correctly classified proportion	0.944 (from 0.907 to 0.967)
Diagnostic odds ratio	300.667 (from 79.850 to 1132.135)
Positive predictive value	0.846 (from 0.739 to 0.914)
Negative predictive value	0.982 (from 0.949 to 0.994)
Positive likelihood ratio	16.500 (from 9.013 to 30.207)
Negative likelihood ratio	0.055 (from 0.018 to 0.165)
**Class geniculatus**	
Apparent prevalence	0.250 (from 0.199 to 0.309)
True prevalence	0.250 (from 0.199 to 0.309)
Sensitivity	0.897 (from 0.792 to 0.952)
Specificity	0.966 (from 0.927 to 0.984)
Correctly classified proportion	0.948 (from 0.912 to 0.970)
Diagnostic odds ratio	242.667 (from 75.049 to 784.653)
Positive predictive value	0.897 (from 0.792 to 0.952)
Negative predictive value	0.966 (from 0.927 to 0.984)
Positive likelihood ratio	26.000 (from 11.787 to 57.350)
Negative likelihood ratio	0.107 (from 0.050 to 0.229)
**Class japonicus**	
Apparent prevalence	0.228 (from 0.179 to 0.287)
True prevalence	0.254 (from 0.203 to 0.314)
Sensitivity	0.644 (from 0.517 to 0.754)
Specificity	0.913 (from 0.862 to 0.947)
Correctly classified proportion	0.845 (from 0.793 to 0.886)
Diagnostic odds ratio	19.060 (from 8.992 to 40.403)
Positive predictive value	0.717 (from 0.584 to 0.820)
Negative predictive value	0.883 (from 0.827 to 0.922)
Positive likelihood ratio	7.428 (from 4.418 to 12.488)
Negative likelihood ratio	0.390 (from 0.276 to 0.551)
**Class koreicus**	
Apparent prevalence	0.241 (from 0.191 to 0.300)
True prevalence	0.246 (from 0.195 to 0.305)
Sensitivity	0.737 (from 0.610 to 0.834)
Specificity	0.920 (from 0.870 to 0.952)
Correctly classified proportion	0.875 (from 0.826 to 0.912)
Diagnostic odds ratio	32.200 (from 14.416 to 71.923)
Positive predictive value	0.750 (from 0.623 to 0.845)
Negative predictive value	0.915 (from 0.864 to 0.948)
Positive likelihood ratio	9.211 (from 5.444 to 15.583)
Negative likelihood ratio	0.286 (from 0.185 to 0.443)

We save the created objects in a list.

```
df.groups_list_dataset[["Quality high"]] <- `d.disc.eggs.l_Quality high`
df.groups_list_diag.measures[["Quality high"]] <- res.diagnostic.measures
```

We clean up the programming environment.

```
rm(tab, nr.obs, l.confusion.matrix,
   l.confusion.matrix.print, res.diagnostic.measures,
   aux.df.rows)
```

7.3 We look at *Quality low*

7.3.1 Dataset

```
## Dataset
`d.disc.eggs.l_Quality low` <- d.disc.eggs.l %>% filter(`group` == "Quality low")
## Number of observations
(nr.obs <- nrow(`d.disc.eggs.l_Quality low`))
```

```
[1] 240
```

This dataset contains 240 observations.

7.3.2 Missing values

Next, we give an overview of the missing values.

```
`d.disc.eggs.l_Quality low` %>%
  select(`answer.rater`, `MALDI-TOF determination`) %>%
  table(useNA = "always")
```

```
             MALDI-TOF determination
answer.rater  albopictus geniculatus japonicus koreicus <NA>
  albopictus          48           7        18        0    0
  geniculatus          7          48         6        2    0
  japonicus            5           1        28       25    0
  koreicus             0           2         6       33    0
  <NA>                 0           2         2        0    0
```

```
## Calculate the number of missing values in both index/reference test:
nr.NA <- `d.disc.eggs.l_Quality low` %>%
  select(`answer.rater`, `MALDI-TOF determination`) %>%
  (\(x){sum(is.na(x))})
## Calculate the number of observations that will be removed:
(nr.deleted.obs <- `d.disc.eggs.l_Quality low` %>%
    filter(is.na(`answer.rater`) | is.na(`MALDI-TOF determination`)) %>%
    nrow())
```

```
[1] 4
```

```
`d.disc.eggs.l_Quality low` <- `d.disc.eggs.l_Quality low` %>%
  filter(!is.na(`answer.rater`) & !is.na(`MALDI-TOF determination`))
```

There were 4 observations (rows) deleted due to missing values.

7.3.3 Confusion Matrix

We calculate the confusion matrices in the following steps.

```
tab <- xtabs(formula = ~ `answer.rater` + `MALDI-TOF determination`,
             data = `d.disc.eggs.l_Quality low`)
tab %>%
  f.create.confusion.matrix() %>%
  as_tibble(rownames = "answer.rater") %>%
  kable(caption = paste0("Overall cross table."),
        label = "tab:CrossTable3",
```

```
        booktabs = TRUE,
        longtable = TRUE,
        linesep = c("")) %>%
  kable_styling(font_size = 7,
                latex_options = c("striped", "repeat_header", "hold_position")) %>%
  row_spec(row = 5, bold = TRUE) %>%
  column_spec(column = 6, bold = TRUE) %>%
  add_header_above(c("", "MALDI-TOF determination" = 4, ""))
```

Table 16: Overall cross table.

	MALDI-TOF determination				
answer.rater	albopictus	geniculatus	japonicus	koreicus	Total
albopictus	48	7	18	0	**73**
geniculatus	7	48	6	2	**63**
japonicus	5	1	28	25	**59**
koreicus	0	2	6	33	**41**
**Total**	**60**	**58**	**58**	**60**	**236**

Example how to read this table:

- There were 48 cases of *albopictus* that were correctly classified as *albopictus*, 7 cases of *albopictus* were wrongly classified as *geniculatus*.
- There were 48 cases of *geniculatus* that were correctly classified as *geniculatus*, 7 cases of *geniculatus* were wrongly classified as *albopictus*.

We present the same table in percentages. Note that all columns add to 100%.

```
tab <- xtabs(formula = ~ `answer.rater` + `MALDI-TOF determination`,
             data = `d.disc.eggs.l_Quality low`)
prop.table(x = tab, margin = 2) %>%
  '*'(100) %>%
  rbind(., colSums(.)) %>%
  f.round(digits = digits.rounding) %>%
  kable(caption = paste0("Overall cross table in percentages. All columns add up to 100\\%."),
        label = "tab:CrossTablePercentage3",
        booktabs = TRUE,
        longtable = TRUE,
        linesep = c("")) %>%
  kable_styling(font_size = 7,
                latex_options = c("striped", "repeat_header", "hold_position")) %>%
  row_spec(row = 5, bold = TRUE) %>%
  add_header_above(c("", "MALDI-TOF determination" = 4))
```

Table 17: Overall cross table in percentages. All columns add up to 100%.

	MALDI-TOF determination			
	albopictus	geniculatus	japonicus	koreicus
albopictus	80.00	12.07	31.03	0.00
geniculatus	11.67	82.76	10.34	3.33
japonicus	8.33	1.72	48.28	41.67
koreicus	0.00	3.45	10.34	55.00
	**100.00**	**100.00**	**100.00**	**100.00**

```
levels.chr <- levels(`d.disc.eggs.l_Quality low`$`answer.rater`)
if(length(levels.chr) == 2){
```

```
  classes <- c("")
}else{
  classes <- levels.chr
}

## calculate tables
if(identical(classes, c(""))){
  l.tab <- list(xtabs(formula = ~ `answer.rater` + `MALDI-TOF determination`,
                      data = `d.disc.eggs.l_Quality low`))
}else{
  l.tab <- lapply(X = classes, FUN = function(x){
    xtabs(formula = ~ `answer.rater` + `MALDI-TOF determination`,
          data = `d.disc.eggs.l_Quality low` %>%
            mutate(across(.cols = c(`answer.rater`, `MALDI-TOF determination`), .fns = function(y){
              ## we merge categories into the category "neg."
              y %>%
                fct_collapse("neg." = setdiff(levels(`d.disc.eggs.l_Quality low`$`answer.rater`), x)) %[illegible]
                fct_relevel(x)
            })))
  })
}
## calculate confusion matrix
l.confusion.matrix <- lapply(X = l.tab,
                             FUN = f.create.confusion.matrix)
## calculate tables for printing
l.confusion.matrix.print <- lapply(X = l.confusion.matrix,
                                   FUN = f.create.confusion.matrix.print)
```

The next tables show the confusion matrices for the index test (“answer.rater”) against the reference test (“MALDI-TOF determination”).

```
for(class in seq_along(classes)){
  l.confusion.matrix.print[[class]] %>%
    as_tibble(rownames = "answer.rater") %>%
    kable(caption = paste0("Confusion matrix for class ‘",
                           levels(`d.disc.eggs.l_Quality low`$`answer.rater`)[class], "’.
          Category ‘", levels(`d.disc.eggs.l_Quality low`$`answer.rater`)[class],
          "’ is considered a positive result.
          Category ‘neg.’ is considered a negative result and
          contains the levels ",
          paste0(setdiff(levels(`d.disc.eggs.l_Quality low`$`answer.rater`),
                         classes[class]), collapse = ", "), "."),
          label = paste0("tab:ConfusionMatrix3Class", class),
          booktabs = TRUE,
          longtable = TRUE,
          linesep = c("")) %>%
    kable_styling(font_size = 7,
                  latex_options = c("striped", "repeat_header", "hold_position")) %>%
    row_spec(row = nrow(l.confusion.matrix.print[[class]]), bold = TRUE) %>%
    column_spec(column = ncol(l.confusion.matrix.print[[class]]) + 1, bold = TRUE) %>%
    add_header_above(c("", "MALDI-TOF determination" =
                         ncol(l.confusion.matrix.print[[class]]) - 1, "")) %>%
    print()
```

```
}
```

Table 18: Confusion matrix for class 'albopictus'. Category 'albopictus' is considered a positive result. Category 'neg.' is considered a negative result and contains the levels geniculatus, japonicus, koreicus.

	MALDI-TOF determination		
answer.rater	albopictus	neg.	Total
albopictus	48 (TP)	25 (FP)	**73**
neg.	12 (FN)	151 (TN)	**163**
**Total**	**60**	**176**	**236**

Table 19: Confusion matrix for class 'geniculatus'. Category 'geniculatus' is considered a positive result. Category 'neg.' is considered a negative result and contains the levels albopictus, japonicus, koreicus.

	MALDI-TOF determination		
answer.rater	geniculatus	neg.	Total
geniculatus	48 (TP)	15 (FP)	**63**
neg.	10 (FN)	163 (TN)	**173**
**Total**	**58**	**178**	**236**

Table 20: Confusion matrix for class 'japonicus'. Category 'japonicus' is considered a positive result. Category 'neg.' is considered a negative result and contains the levels albopictus, geniculatus, koreicus.

	MALDI-TOF determination		
answer.rater	japonicus	neg.	Total
japonicus	28 (TP)	31 (FP)	**59**
neg.	30 (FN)	147 (TN)	**177**
**Total**	**58**	**178**	**236**

Table 21: Confusion matrix for class 'koreicus'. Category 'koreicus' is considered a positive result. Category 'neg.' is considered a negative result and contains the levels albopictus, geniculatus, japonicus.

	MALDI-TOF determination		
answer.rater	koreicus	neg.	Total
koreicus	33 (TP)	8 (FP)	**41**
neg.	27 (FN)	168 (TN)	**195**
**Total**	**60**	**176**	**236**

7.3.4 Measures of diagnostic accuracy

We now calculate measures of diagnostic accuracy.

```
res.diagnostic.measures <- tibble("Class" = classes) %>%
  mutate(tabs = l.tab) %>%

  ## epiR wants it in the format: c(TP, FP, FN, TN)
  mutate(confusion.vector = map(.x = tabs, .f = function(x){
    c("tp" = x[1, 1], "fp" = x[1, 2], "fn" = x[2, 1], "tn" = x[2, 2])
  })) %>%

  ## Calculate measures of diagnostic accuracy
  mutate(out.EpiR = map(.x = confusion.vector, .f = function(x){
    epi.tests(dat = x,
              method = "wilson",
              conf.level = 0.95)
  })) %>%

  ## reformat output, filter diag. measures of interest
  mutate(diag.measures = map(.x = out.EpiR, .f = function(x){
    x <- x %>%
      summary() %>%
      as_tibble(rownames = "name.raw") %>%
      mutate()
    if(f.check.version(x = packageVersion(pkg = "epiR"))){
      x <- x %>%
        mutate(name.raw = statistic) %>%
        select(-statistic)
    }
    x %>%
      left_join(y = .match, by = c("name.raw")) %>%
      filter(name.raw %in% c("ap", "tp", "se", "sp", "diag.or", "pv.pos", "pv.neg", "lr.pos", "lr.neg",
  })) %>%

  ## ensure boundaries of estimates and CI
  mutate(diag.measures = map(.x = diag.measures, .f = function(x){
    x %>%
      mutate(across(.cols = c(est, lower, upper), .fns = function(x){
        if_else(condition = bounded, true = pmin(1, pmax(0, x)), false = x)
      }))
  })) %>%

  ## put together output
  mutate(diag.measures.print = map(.x = diag.measures, .f = function(x){
    x %>%
      mutate(across(.cols = c("est", "lower", "upper"),
                    .fns = \(x){format(x = round(x = x, digits = digits.rounding + 1),
                                       scientific = FALSE,
                                       nsmall = digits.rounding + 1)})) %>%
      mutate("Estimate (95% CI)" = paste0(est, " (from ", lower, " to ", upper, ")")) %>%
      select(Measure, `Estimate (95% CI)`)
  }))
```

We restructure the results that are to be printed.

```r
aux.df.rows <- res.diagnostic.measures %>%
  select(Class, diag.measures.print) %>%
  mutate(Class = paste0("Class ", Class)) %>%
  unnest(cols = diag.measures.print) %>%
  mutate(row.nr = seq_len(n())) %>%
  group_by(Class) %>%
  summarize(min = min(row.nr),
            max = max(row.nr)) %>%
  ungroup()
```

We prepare the printing of the table.

```r
res.diagnostic.measures_print3 <- res.diagnostic.measures %>%
  select(Class, diag.measures.print) %>%
  unnest(cols = diag.measures.print) %>%
  select(-Class) %>%

  kable(caption = paste0("Estimate and 95\\% confidence intervals for prevalence
                         and different measures of accuracy."),
        label = "tab:ResultTable3",
        booktabs = TRUE,
        longtable = TRUE,
        linesep = c("")) %>%
  kable_styling(
    font_size = 7,
    repeat_header_method = "replace",
    repeat_header_text = paste0("Estimate and 95\\% confidence intervals for prevalence
                                and different measures of accuracy. (continued)"),
    latex_options = c("striped", "repeat_header", "hold_position")
  ) %>%

  group_rows(group_label = aux.df.rows$Class[1],
             start_row = aux.df.rows$min[1],
             end_row = aux.df.rows$max[1]) %>%
  group_rows(group_label = aux.df.rows$Class[2],
             start_row = aux.df.rows$min[2],
             end_row = aux.df.rows$max[2]) %>%
  group_rows(group_label = aux.df.rows$Class[3],
             start_row = aux.df.rows$min[3],
             end_row = aux.df.rows$max[3]) %>%
  group_rows(group_label = aux.df.rows$Class[4],
             start_row = aux.df.rows$min[4],
             end_row = aux.df.rows$max[4]) %>%
  collapse_rows(columns = 1, latex_hline = "major", valign = "middle")
```

The next table shows point estimate and 95% confidence intervals for prevalence and different measures of diagnostic accuracy.

```r
res.diagnostic.measures_print3
```

Table 22: Estimate and 95% confidence intervals for prevalence and different measures of accuracy.

Measure	Estimate (95% CI)
**Class albopictus**	
Apparent prevalence	0.309 (from 0.254 to 0.371)

Table 22: Estimate and 95% confidence intervals for prevalence and different measures of accuracy. (continued)

Measure	Estimate (95% CI)
True prevalence	0.254 (from 0.203 to 0.313)
Sensitivity	0.800 (from 0.682 to 0.882)
Specificity	0.858 (from 0.799 to 0.902)
Correctly classified proportion	0.843 (from 0.791 to 0.884)
Diagnostic odds ratio	24.160 (from 11.287 to 51.717)
Positive predictive value	0.658 (from 0.543 to 0.756)
Negative predictive value	0.926 (from 0.876 to 0.957)
Positive likelihood ratio	5.632 (from 3.834 to 8.273)
Negative likelihood ratio	0.233 (from 0.140 to 0.388)
**Class geniculatus**	
Apparent prevalence	0.267 (from 0.215 to 0.327)
True prevalence	0.246 (from 0.195 to 0.304)
Sensitivity	0.828 (from 0.711 to 0.904)
Specificity	0.916 (from 0.866 to 0.948)
Correctly classified proportion	0.894 (from 0.848 to 0.927)
Diagnostic odds ratio	52.160 (from 22.018 to 123.566)
Positive predictive value	0.762 (from 0.644 to 0.850)
Negative predictive value	0.942 (from 0.897 to 0.968)
Positive likelihood ratio	9.821 (from 5.967 to 16.164)
Negative likelihood ratio	0.188 (from 0.107 to 0.331)
**Class japonicus**	
Apparent prevalence	0.250 (from 0.199 to 0.309)
True prevalence	0.246 (from 0.195 to 0.304)
Sensitivity	0.483 (from 0.359 to 0.608)
Specificity	0.826 (from 0.763 to 0.875)
Correctly classified proportion	0.742 (from 0.682 to 0.793)
Diagnostic odds ratio	4.426 (from 2.323 to 8.431)
Positive predictive value	0.475 (from 0.353 to 0.600)
Negative predictive value	0.831 (from 0.768 to 0.879)
Positive likelihood ratio	2.772 (from 1.828 to 4.203)
Negative likelihood ratio	0.626 (from 0.484 to 0.810)
**Class koreicus**	
Apparent prevalence	0.174 (from 0.131 to 0.227)
True prevalence	0.254 (from 0.203 to 0.313)
Sensitivity	0.550 (from 0.425 to 0.669)
Specificity	0.955 (from 0.913 to 0.977)
Correctly classified proportion	0.852 (from 0.801 to 0.891)
Diagnostic odds ratio	25.667 (from 10.723 to 61.434)
Positive predictive value	0.805 (from 0.660 to 0.898)
Negative predictive value	0.862 (from 0.806 to 0.903)
Positive likelihood ratio	12.100 (from 5.921 to 24.726)
Negative likelihood ratio	0.471 (from 0.356 to 0.625)

We save the created objects in a list.

```
df.groups_list_dataset[["Quality low"]] <- `d.disc.eggs.l_Quality low`
df.groups_list_diag.measures[["Quality low"]] <- res.diagnostic.measures
```

We clean up the programming environment.

```
rm(tab, nr.obs, l.confusion.matrix,
   l.confusion.matrix.print, res.diagnostic.measures,
   aux.df.rows)
```

7.4 We look at *Quality medium*

7.4.1 Dataset

```
## Dataset
`d.disc.eggs.l_Quality medium` <- d.disc.eggs.l %>% filter(`group` == "Quality medium")
## Number of observations
(nr.obs <- nrow(`d.disc.eggs.l_Quality medium`))
```

```
[1] 240
```

This dataset contains 240 observations.

7.4.2 Missing values

Next, we give an overview of the missing values.

```
`d.disc.eggs.l_Quality medium` %>%
  select(`answer.rater`, `MALDI-TOF determination`) %>%
  table(useNA = "always")
```

```
             MALDI-TOF determination
answer.rater  albopictus geniculatus japonicus koreicus <NA>
  albopictus          52           5        10        6    0
  geniculatus          4          51         9        0    0
  japonicus            1           2        37       28    0
  koreicus             0           1         3       25    0
  <NA>                 3           1         1        1    0
```

```
## Calculate the number of missing values in both index/reference test:
nr.NA <- `d.disc.eggs.l_Quality medium` %>%
  select(`answer.rater`, `MALDI-TOF determination`) %>%
  (\(x){sum(is.na(x))})
## Calculate the number of observations that will be removed:
(nr.deleted.obs <- `d.disc.eggs.l_Quality medium` %>%
    filter(is.na(`answer.rater`) | is.na(`MALDI-TOF determination`)) %>%
    nrow())
```

```
[1] 6
```

```
`d.disc.eggs.l_Quality medium` <- `d.disc.eggs.l_Quality medium` %>%
  filter(!is.na(`answer.rater`) & !is.na(`MALDI-TOF determination`))
```

There were 6 observations (rows) deleted due to missing values.

7.4.3 Confusion Matrix

We calculate the confusion matrices in the following steps.

```
tab <- xtabs(formula = ~ `answer.rater` + `MALDI-TOF determination`,
             data = `d.disc.eggs.l_Quality medium`)
tab %>%
  f.create.confusion.matrix() %>%
  as_tibble(rownames = "answer.rater") %>%
  kable(caption = paste0("Overall cross table."),
        label = "tab:CrossTable4",
```

```
        booktabs = TRUE,
        longtable = TRUE,
        linesep = c("")) %>%
  kable_styling(font_size = 7,
                latex_options = c("striped", "repeat_header", "hold_position")) %>%
  row_spec(row = 5, bold = TRUE) %>%
  column_spec(column = 6, bold = TRUE) %>%
  add_header_above(c("", "MALDI-TOF determination" = 4, ""))
```

Table 23: Overall cross table.

	MALDI-TOF determination				
answer.rater	albopictus	geniculatus	japonicus	koreicus	Total
albopictus	52	5	10	6	**73**
geniculatus	4	51	9	0	**64**
japonicus	1	2	37	28	**68**
koreicus	0	1	3	25	**29**
**Total**	**57**	**59**	**59**	**59**	**234**

Example how to read this table:

- There were 52 cases of *albopictus* that were correctly classified as *albopictus*, 4 cases of *albopictus* were wrongly classified as *geniculatus*.
- There were 51 cases of *geniculatus* that were correctly classified as *geniculatus*, 5 cases of *geniculatus* were wrongly classified as *albopictus*.

We present the same table in percentages. Note that all columns add to 100%.

```
tab <- xtabs(formula = ~ `answer.rater` + `MALDI-TOF determination`,
             data = `d.disc.eggs.l_Quality medium`)
prop.table(x = tab, margin = 2) %>%
  '*'(100) %>%
  rbind(., colSums(.)) %>%
  f.round(digits = digits.rounding) %>%
  kable(caption = paste0("Overall cross table in percentages. All columns add up to 100\\%."),
        label = "tab:CrossTablePercentage4",
        booktabs = TRUE,
        longtable = TRUE,
        linesep = c("")) %>%
  kable_styling(font_size = 7,
                latex_options = c("striped", "repeat_header", "hold_position")) %>%
  row_spec(row = 5, bold = TRUE) %>%
  add_header_above(c("", "MALDI-TOF determination" = 4))
```

Table 24: Overall cross table in percentages. All columns add up to 100%.

	MALDI-TOF determination			
	albopictus	geniculatus	japonicus	koreicus
albopictus	91.23	8.47	16.95	10.17
geniculatus	7.02	86.44	15.25	0.00
japonicus	1.75	3.39	62.71	47.46
koreicus	0.00	1.69	5.08	42.37
	**100.00**	**100.00**	**100.00**	**100.00**

```
levels.chr <- levels(`d.disc.eggs.l_Quality medium`$`answer.rater`)
if(length(levels.chr) == 2){
```

```
  classes <- c("")
}else{
  classes <- levels.chr
}

## calculate tables
if(identical(classes, c(""))){
  l.tab <- list(xtabs(formula = ~ `answer.rater` + `MALDI-TOF determination`,
                      data = `d.disc.eggs.l_Quality medium`))
}else{
  l.tab <- lapply(X = classes, FUN = function(x){
    xtabs(formula = ~ `answer.rater` + `MALDI-TOF determination`,
          data = `d.disc.eggs.l_Quality medium` %>%
            mutate(across(.cols = c(`answer.rater`, `MALDI-TOF determination`), .fns = function(y){
              ## we merge categories into the category "neg."
              y %>%
                fct_collapse("neg." = setdiff(levels(`d.disc.eggs.l_Quality medium`$`answer.rater`), x))
                fct_relevel(x)
            })))
  })
}
## calculate confusion matrix
l.confusion.matrix <- lapply(X = l.tab,
                             FUN = f.create.confusion.matrix)
## calculate tables for printing
l.confusion.matrix.print <- lapply(X = l.confusion.matrix,
                                   FUN = f.create.confusion.matrix.print)
```

The next tables show the confusion matrices for the index test ("answer.rater") against the reference test ("MALDI-TOF determination").

```
for(class in seq_along(classes)){
  l.confusion.matrix.print[[class]] %>%
    as_tibble(rownames = "answer.rater") %>%
    kable(caption = paste0("Confusion matrix for class ‘",
                           levels(`d.disc.eggs.l_Quality medium`$`answer.rater`)[class], "’.
          Category ‘", levels(`d.disc.eggs.l_Quality medium`$`answer.rater`)[class],
          "’ is considered a positive result.
          Category ‘neg.’ is considered a negative result and
          contains the levels ",
          paste0(setdiff(levels(`d.disc.eggs.l_Quality medium`$`answer.rater`),
                         classes[class]), collapse = ", "), "."),
          label = paste0("tab:ConfusionMatrix4Class", class),
          booktabs = TRUE,
          longtable = TRUE,
          linesep = c("")) %>%
    kable_styling(font_size = 7,
                  latex_options = c("striped", "repeat_header", "hold_position")) %>%
    row_spec(row = nrow(l.confusion.matrix.print[[class]]), bold = TRUE) %>%
    column_spec(column = ncol(l.confusion.matrix.print[[class]]) + 1, bold = TRUE) %>%
    add_header_above(c("", "MALDI-TOF determination" =
                         ncol(l.confusion.matrix.print[[class]]) - 1, "")) %>%
    print()
```

```
}
```

Table 25: Confusion matrix for class ‘albopictus’. Category ‘albopictus’ is considered a positive result. Category ‘neg.’ is considered a negative result and contains the levels geniculatus, japonicus, koreicus.

	MALDI-TOF determination		
answer.rater	albopictus	neg.	Total
albopictus	52 (TP)	21 (FP)	**73**
neg.	5 (FN)	156 (TN)	**161**
**Total**	**57**	**177**	**234**

Table 26: Confusion matrix for class ‘geniculatus’. Category ‘geniculatus’ is considered a positive result. Category ‘neg.’ is considered a negative result and contains the levels albopictus, japonicus, koreicus.

	MALDI-TOF determination		
answer.rater	geniculatus	neg.	Total
geniculatus	51 (TP)	13 (FP)	**64**
neg.	8 (FN)	162 (TN)	**170**
**Total**	**59**	**175**	**234**

Table 27: Confusion matrix for class ‘japonicus’. Category ‘japonicus’ is considered a positive result. Category ‘neg.’ is considered a negative result and contains the levels albopictus, geniculatus, koreicus.

	MALDI-TOF determination		
answer.rater	japonicus	neg.	Total
japonicus	37 (TP)	31 (FP)	**68**
neg.	22 (FN)	144 (TN)	**166**
**Total**	**59**	**175**	**234**

Table 28: Confusion matrix for class ‘koreicus’. Category ‘koreicus’ is considered a positive result. Category ‘neg.’ is considered a negative result and contains the levels albopictus, geniculatus, japonicus.

	MALDI-TOF determination		
answer.rater	koreicus	neg.	Total
koreicus	25 (TP)	4 (FP)	**29**
neg.	34 (FN)	171 (TN)	**205**
**Total**	**59**	**175**	**234**

7.4.4 Measures of diagnostic accuracy

We now calculate measures of diagnostic accuracy.

```
res.diagnostic.measures <- tibble("Class" = classes) %>%
  mutate(tabs = l.tab) %>%

  ## epiR wants it in the format: c(TP, FP, FN, TN)
  mutate(confusion.vector = map(.x = tabs, .f = function(x){
    c("tp" = x[1, 1], "fp" = x[1, 2], "fn" = x[2, 1], "tn" = x[2, 2])
  })) %>%

  ## Calculate measures of diagnostic accuracy
  mutate(out.EpiR = map(.x = confusion.vector, .f = function(x){
    epi.tests(dat = x,
              method = "wilson",
              conf.level = 0.95)
  })) %>%

  ## reformat output, filter diag. measures of interest
  mutate(diag.measures = map(.x = out.EpiR, .f = function(x){
    x <- x %>%
      summary() %>%
      as_tibble(rownames = "name.raw") %>%
      mutate()
    if(f.check.version(x = packageVersion(pkg = "epiR"))){
      x <- x %>%
        mutate(name.raw = statistic) %>%
        select(-statistic)
    }
    x %>%
      left_join(y = .match, by = c("name.raw")) %>%
      filter(name.raw %in% c("ap", "tp", "se", "sp", "diag.or", "pv.pos", "pv.neg", "lr.pos", "lr.neg",
  })) %>%

  ## ensure boundaries of estimates and CI
  mutate(diag.measures = map(.x = diag.measures, .f = function(x){
    x %>%
      mutate(across(.cols = c(est, lower, upper), .fns = function(x){
        if_else(condition = bounded, true = pmin(1, pmax(0, x)), false = x)
      }))
  })) %>%

  ## put together output
  mutate(diag.measures.print = map(.x = diag.measures, .f = function(x){
    x %>%
      mutate(across(.cols = c("est", "lower", "upper"),
                    .fns = \(x){format(x = round(x = x, digits = digits.rounding + 1),
                                       scientific = FALSE,
                                       nsmall = digits.rounding + 1)})) %>%
      mutate("Estimate (95% CI)" = paste0(est, " (from ", lower, " to ", upper, ")")) %>%
      select(Measure, `Estimate (95% CI)`)
  }))
```

We restructure the results that are to be printed.

```r
aux.df.rows <- res.diagnostic.measures %>%
  select(Class, diag.measures.print) %>%
  mutate(Class = paste0("Class ", Class)) %>%
  unnest(cols = diag.measures.print) %>%
  mutate(row.nr = seq_len(n())) %>%
  group_by(Class) %>%
  summarize(min = min(row.nr),
            max = max(row.nr)) %>%
  ungroup()
```

We prepare the printing of the table.

```r
res.diagnostic.measures_print4 <- res.diagnostic.measures %>%
  select(Class, diag.measures.print) %>%
  unnest(cols = diag.measures.print) %>%
  select(-Class) %>%

  kable(caption = paste0("Estimate and 95\\% confidence intervals for prevalence
                         and different measures of accuracy."),
        label = "tab:ResultTable4",
        booktabs = TRUE,
        longtable = TRUE,
        linesep = c("")) %>%
  kable_styling(
    font_size = 7,
    repeat_header_method = "replace",
    repeat_header_text = paste0("Estimate and 95\\% confidence intervals for prevalence
                                and different measures of accuracy. (continued)"),
    latex_options = c("striped", "repeat_header", "hold_position")
  ) %>%

  group_rows(group_label = aux.df.rows$Class[1],
             start_row = aux.df.rows$min[1],
             end_row = aux.df.rows$max[1]) %>%
  group_rows(group_label = aux.df.rows$Class[2],
             start_row = aux.df.rows$min[2],
             end_row = aux.df.rows$max[2]) %>%
  group_rows(group_label = aux.df.rows$Class[3],
             start_row = aux.df.rows$min[3],
             end_row = aux.df.rows$max[3]) %>%
  group_rows(group_label = aux.df.rows$Class[4],
             start_row = aux.df.rows$min[4],
             end_row = aux.df.rows$max[4]) %>%
  collapse_rows(columns = 1, latex_hline = "major", valign = "middle")
```

The next table shows point estimate and 95% confidence intervals for prevalence and different measures of diagnostic accuracy.

```r
res.diagnostic.measures_print4
```

Table 29: Estimate and 95% confidence intervals for prevalence and different measures of accuracy.

Measure	Estimate (95% CI)
**Class albopictus**	
Apparent prevalence	0.312 (from 0.256 to 0.374)

Table 29: Estimate and 95% confidence intervals for prevalence and different measures of accuracy. (continued)

Measure	Estimate (95% CI)
True prevalence	0.244 (from 0.193 to 0.302)
Sensitivity	0.912 (from 0.811 to 0.962)
Specificity	0.881 (from 0.825 to 0.921)
Correctly classified proportion	0.889 (from 0.842 to 0.923)
Diagnostic odds ratio	77.257 (from 27.732 to 215.228)
Positive predictive value	0.712 (from 0.600 to 0.803)
Negative predictive value	0.969 (from 0.929 to 0.987)
Positive likelihood ratio	7.689 (from 5.105 to 11.581)
Negative likelihood ratio	0.100 (from 0.043 to 0.230)
**Class geniculatus**	
Apparent prevalence	0.274 (from 0.220 to 0.334)
True prevalence	0.252 (from 0.201 to 0.311)
Sensitivity	0.864 (from 0.755 to 0.930)
Specificity	0.926 (from 0.877 to 0.956)
Correctly classified proportion	0.910 (from 0.867 to 0.941)
Diagnostic odds ratio	79.442 (from 31.180 to 202.409)
Positive predictive value	0.797 (from 0.683 to 0.877)
Negative predictive value	0.953 (from 0.910 to 0.976)
Positive likelihood ratio	11.636 (from 6.831 to 19.822)
Negative likelihood ratio	0.146 (from 0.077 to 0.279)
**Class japonicus**	
Apparent prevalence	0.291 (from 0.236 to 0.352)
True prevalence	0.252 (from 0.201 to 0.311)
Sensitivity	0.627 (from 0.500 to 0.739)
Specificity	0.823 (from 0.760 to 0.872)
Correctly classified proportion	0.774 (from 0.716 to 0.822)
Diagnostic odds ratio	7.812 (from 4.058 to 15.040)
Positive predictive value	0.544 (from 0.427 to 0.657)
Negative predictive value	0.867 (from 0.807 to 0.911)
Positive likelihood ratio	3.540 (from 2.433 to 5.151)
Negative likelihood ratio	0.453 (from 0.323 to 0.635)
**Class koreicus**	
Apparent prevalence	0.124 (from 0.088 to 0.172)
True prevalence	0.252 (from 0.201 to 0.311)
Sensitivity	0.424 (from 0.306 to 0.551)
Specificity	0.977 (from 0.943 to 0.991)
Correctly classified proportion	0.838 (from 0.785 to 0.879)
Diagnostic odds ratio	31.434 (from 10.279 to 96.128)
Positive predictive value	0.862 (from 0.694 to 0.945)
Negative predictive value	0.834 (from 0.777 to 0.879)
Positive likelihood ratio	18.538 (from 6.729 to 51.071)
Negative likelihood ratio	0.590 (from 0.473 to 0.735)

We save the created objects in a list.

```
df.groups_list_dataset[["Quality medium"]] <- `d.disc.eggs.l_Quality medium`
df.groups_list_diag.measures[["Quality medium"]] <- res.diagnostic.measures
```

We clean up the programming environment.

```
rm(tab, nr.obs, l.confusion.matrix,
   l.confusion.matrix.print, res.diagnostic.measures,
   aux.df.rows)
```

7.5 We look at *Rater level expert*

7.5.1 Dataset

```
## Dataset
`d.disc.eggs.l_Rater level expert` <- d.disc.eggs.l %>% filter(`group` == "Rater level expert")
## Number of observations
(nr.obs <- nrow(`d.disc.eggs.l_Rater level expert`))
```

```
[1] 600
```

This dataset contains 600 observations.

7.5.2 Missing values

Next, we give an overview of the missing values.

```
`d.disc.eggs.l_Rater level expert` %>%
  select(`answer.rater`, `MALDI-TOF determination`) %>%
  table(useNA = "always")
```

```
             MALDI-TOF determination
answer.rater  albopictus geniculatus japonicus koreicus <NA>
  albopictus         126          15        28        5    0
  geniculatus         12         124        17        2    0
  japonicus            7           2        82       58    0
  koreicus             0           4        19       83    0
  <NA>                 5           5         4        2    0
```

```
## Calculate the number of missing values in both index/reference test:
nr.NA <- `d.disc.eggs.l_Rater level expert` %>%
  select(`answer.rater`, `MALDI-TOF determination`) %>%
  (\(x){sum(is.na(x))})
## Calculate the number of observations that will be removed:
(nr.deleted.obs <- `d.disc.eggs.l_Rater level expert` %>%
    filter(is.na(`answer.rater`) | is.na(`MALDI-TOF determination`)) %>%
    nrow())
```

```
[1] 16
```

```
`d.disc.eggs.l_Rater level expert` <- `d.disc.eggs.l_Rater level expert` %>%
  filter(!is.na(`answer.rater`) & !is.na(`MALDI-TOF determination`))
```

There were 16 observations (rows) deleted due to missing values.

7.5.3 Confusion Matrix

We calculate the confusion matrices in the following steps.

```
tab <- xtabs(formula = ~ `answer.rater` + `MALDI-TOF determination`,
             data = `d.disc.eggs.l_Rater level expert`)
tab %>%
  f.create.confusion.matrix() %>%
  as_tibble(rownames = "answer.rater") %>%
  kable(caption = paste0("Overall cross table."),
        label = "tab:CrossTable5",
```

```
         booktabs = TRUE,
         longtable = TRUE,
         linesep = c("")) %>%
  kable_styling(font_size = 7,
                latex_options = c("striped", "repeat_header", "hold_position")) %>%
  row_spec(row = 5, bold = TRUE) %>%
  column_spec(column = 6, bold = TRUE) %>%
  add_header_above(c("", "MALDI-TOF determination" = 4, ""))
```

Table 30: Overall cross table.

	MALDI-TOF determination				
answer.rater	albopictus	geniculatus	japonicus	koreicus	Total
albopictus	126	15	28	5	**174**
geniculatus	12	124	17	2	**155**
japonicus	7	2	82	58	**149**
koreicus	0	4	19	83	**106**
**Total**	**145**	**145**	**146**	**148**	**584**

Example how to read this table:

- There were 126 cases of *albopictus* that were correctly classified as *albopictus*, 12 cases of *albopictus* were wrongly classified as *geniculatus*.
- There were 124 cases of *geniculatus* that were correctly classified as *geniculatus*, 15 cases of *geniculatus* were wrongly classified as *albopictus*.

We present the same table in percentages. Note that all columns add to 100%.

```
tab <- xtabs(formula = ~ `answer.rater` + `MALDI-TOF determination`,
             data = `d.disc.eggs.l_Rater level expert`)
prop.table(x = tab, margin = 2) %>%
  '*'(100) %>%
  rbind(., colSums(.)) %>%
  f.round(digits = digits.rounding) %>%
  kable(caption = paste0("Overall cross table in percentages. All columns add up to 100\\%."),
        label = "tab:CrossTablePercentage5",
        booktabs = TRUE,
        longtable = TRUE,
        linesep = c("")) %>%
  kable_styling(font_size = 7,
                latex_options = c("striped", "repeat_header", "hold_position")) %>%
  row_spec(row = 5, bold = TRUE) %>%
  add_header_above(c("", "MALDI-TOF determination" = 4))
```

Table 31: Overall cross table in percentages. All columns add up to 100%.

	MALDI-TOF determination			
	albopictus	geniculatus	japonicus	koreicus
albopictus	86.90	10.34	19.18	3.38
geniculatus	8.28	85.52	11.64	1.35
japonicus	4.83	1.38	56.16	39.19
koreicus	0.00	2.76	13.01	56.08
	**100.00**	**100.00**	**100.00**	**100.00**

```
levels.chr <- levels(`d.disc.eggs.l_Rater level expert`$`answer.rater`)
if(length(levels.chr) == 2){
```

```
  classes <- c("")
}else{
  classes <- levels.chr
}

## calculate tables
if(identical(classes, c(""))){
  l.tab <- list(xtabs(formula = ~ `answer.rater` + `MALDI-TOF determination`,
                      data = `d.disc.eggs.l_Rater level expert`))
}else{
  l.tab <- lapply(X = classes, FUN = function(x){
    xtabs(formula = ~ `answer.rater` + `MALDI-TOF determination`,
          data = `d.disc.eggs.l_Rater level expert` %>%
            mutate(across(.cols = c(`answer.rater`, `MALDI-TOF determination`), .fns = function(y){
              ## we merge categories into the category "neg."
              y %>%
                fct_collapse("neg." = setdiff(levels(`d.disc.eggs.l_Rater level expert`$`answer.rater`)
                fct_relevel(x)
            })))
  })
}
## calculate confusion matrix
l.confusion.matrix <- lapply(X = l.tab,
                             FUN = f.create.confusion.matrix)
## calculate tables for printing
l.confusion.matrix.print <- lapply(X = l.confusion.matrix,
                                   FUN = f.create.confusion.matrix.print)
```

The next tables show the confusion matrices for the index test ("answer.rater") against the reference test ("MALDI-TOF determination").

```
for(class in seq_along(classes)){
  l.confusion.matrix.print[[class]] %>%
    as_tibble(rownames = "answer.rater") %>%
    kable(caption = paste0("Confusion matrix for class ‘",
                           levels(`d.disc.eggs.l_Rater level expert`$`answer.rater`)[class], "’.
          Category ‘", levels(`d.disc.eggs.l_Rater level expert`$`answer.rater`)[class],
          "’ is considered a positive result.
          Category ‘neg.’ is considered a negative result and
          contains the levels ",
          paste0(setdiff(levels(`d.disc.eggs.l_Rater level expert`$`answer.rater`),
                         classes[class]), collapse = ", "), "."),
          label = paste0("tab:ConfusionMatrix5Class", class),
          booktabs = TRUE,
          longtable = TRUE,
          linesep = c("")) %>%
    kable_styling(font_size = 7,
                  latex_options = c("striped", "repeat_header", "hold_position")) %>%
    row_spec(row = nrow(l.confusion.matrix.print[[class]]), bold = TRUE) %>%
    column_spec(column = ncol(l.confusion.matrix.print[[class]]) + 1, bold = TRUE) %>%
    add_header_above(c("", "MALDI-TOF determination" =
                         ncol(l.confusion.matrix.print[[class]]) - 1, "")) %>%
    print()
```

```
}
```

Table 32: Confusion matrix for class 'albopictus'. Category 'albopictus' is considered a positive result. Category 'neg.' is considered a negative result and contains the levels geniculatus, japonicus, koreicus.

	MALDI-TOF determination		
answer.rater	albopictus	neg.	Total
albopictus	126 (TP)	48 (FP)	**174**
neg.	19 (FN)	391 (TN)	**410**
**Total**	**145**	**439**	**584**

Table 33: Confusion matrix for class 'geniculatus'. Category 'geniculatus' is considered a positive result. Category 'neg.' is considered a negative result and contains the levels albopictus, japonicus, koreicus.

	MALDI-TOF determination		
answer.rater	geniculatus	neg.	Total
geniculatus	124 (TP)	31 (FP)	**155**
neg.	21 (FN)	408 (TN)	**429**
**Total**	**145**	**439**	**584**

Table 34: Confusion matrix for class 'japonicus'. Category 'japonicus' is considered a positive result. Category 'neg.' is considered a negative result and contains the levels albopictus, geniculatus, koreicus.

	MALDI-TOF determination		
answer.rater	japonicus	neg.	Total
japonicus	82 (TP)	67 (FP)	**149**
neg.	64 (FN)	371 (TN)	**435**
**Total**	**146**	**438**	**584**

Table 35: Confusion matrix for class 'koreicus'. Category 'koreicus' is considered a positive result. Category 'neg.' is considered a negative result and contains the levels albopictus, geniculatus, japonicus.

	MALDI-TOF determination		
answer.rater	koreicus	neg.	Total
koreicus	83 (TP)	23 (FP)	**106**
neg.	65 (FN)	413 (TN)	**478**
**Total**	**148**	**436**	**584**

7.5.4 Measures of diagnostic accuracy

We now calculate measures of diagnostic accuracy.

```
res.diagnostic.measures <- tibble("Class" = classes) %>%
  mutate(tabs = l.tab) %>%

  ## epiR wants it in the format: c(TP, FP, FN, TN)
  mutate(confusion.vector = map(.x = tabs, .f = function(x){
    c("tp" = x[1, 1], "fp" = x[1, 2], "fn" = x[2, 1], "tn" = x[2, 2])
  })) %>%

  ## Calculate measures of diagnostic accuracy
  mutate(out.EpiR = map(.x = confusion.vector, .f = function(x){
    epi.tests(dat = x,
              method = "wilson",
              conf.level = 0.95)
  })) %>%

  ## reformat output, filter diag. measures of interest
  mutate(diag.measures = map(.x = out.EpiR, .f = function(x){
    x <- x %>%
      summary() %>%
      as_tibble(rownames = "name.raw") %>%
      mutate()
    if(f.check.version(x = packageVersion(pkg = "epiR"))){
      x <- x %>%
        mutate(name.raw = statistic) %>%
        select(-statistic)
    }
    x %>%
      left_join(y = .match, by = c("name.raw")) %>%
      filter(name.raw %in% c("ap", "tp", "se", "sp", "diag.or", "pv.pos", "pv.neg", "lr.pos", "lr.neg",
  })) %>%

  ## ensure boundaries of estimates and CI
  mutate(diag.measures = map(.x = diag.measures, .f = function(x){
    x %>%
      mutate(across(.cols = c(est, lower, upper), .fns = function(x){
        if_else(condition = bounded, true = pmin(1, pmax(0, x)), false = x)
      }))
  })) %>%

  ## put together output
  mutate(diag.measures.print = map(.x = diag.measures, .f = function(x){
    x %>%
      mutate(across(.cols = c("est", "lower", "upper"),
                    .fns = \(x){format(x = round(x = x, digits = digits.rounding + 1),
                                       scientific = FALSE,
                                       nsmall = digits.rounding + 1)})) %>%
      mutate("Estimate (95% CI)" = paste0(est, " (from ", lower, " to ", upper, ")")) %>%
      select(Measure, `Estimate (95% CI)`)
  }))
```

We restructure the results that are to be printed.

```r
aux.df.rows <- res.diagnostic.measures %>%
  select(Class, diag.measures.print) %>%
  mutate(Class = paste0("Class ", Class)) %>%
  unnest(cols = diag.measures.print) %>%
  mutate(row.nr = seq_len(n())) %>%
  group_by(Class) %>%
  summarize(min = min(row.nr),
            max = max(row.nr)) %>%
  ungroup()
```

We prepare the printing of the table.

```r
res.diagnostic.measures_print5 <- res.diagnostic.measures %>%
  select(Class, diag.measures.print) %>%
  unnest(cols = diag.measures.print) %>%
  select(-Class) %>%

  kable(caption = paste0("Estimate and 95\\% confidence intervals for prevalence
                         and different measures of accuracy."),
        label = "tab:ResultTable5",
        booktabs = TRUE,
        longtable = TRUE,
        linesep = c("")) %>%
  kable_styling(
    font_size = 7,
    repeat_header_method = "replace",
    repeat_header_text = paste0("Estimate and 95\\% confidence intervals for prevalence
                                and different measures of accuracy. (continued)"),
    latex_options = c("striped", "repeat_header", "hold_position")
  ) %>%

  group_rows(group_label = aux.df.rows$Class[1],
             start_row = aux.df.rows$min[1],
             end_row = aux.df.rows$max[1]) %>%
  group_rows(group_label = aux.df.rows$Class[2],
             start_row = aux.df.rows$min[2],
             end_row = aux.df.rows$max[2]) %>%
  group_rows(group_label = aux.df.rows$Class[3],
             start_row = aux.df.rows$min[3],
             end_row = aux.df.rows$max[3]) %>%
  group_rows(group_label = aux.df.rows$Class[4],
             start_row = aux.df.rows$min[4],
             end_row = aux.df.rows$max[4]) %>%
  collapse_rows(columns = 1, latex_hline = "major", valign = "middle")
```

The next table shows point estimate and 95% confidence intervals for prevalence and different measures of diagnostic accuracy.

```r
res.diagnostic.measures_print5
```

Table 36: Estimate and 95% confidence intervals for prevalence and different measures of accuracy.

Measure	Estimate (95% CI)
**Class albopictus**	
Apparent prevalence	0.298 (from 0.262 to 0.336)

Table 36: Estimate and 95% confidence intervals for prevalence and different measures of accuracy. (continued)

Measure	Estimate (95% CI)
True prevalence	0.248 (from 0.215 to 0.285)
Sensitivity	0.869 (from 0.804 to 0.914)
Specificity	0.891 (from 0.858 to 0.917)
Correctly classified proportion	0.885 (from 0.857 to 0.909)
Diagnostic odds ratio	54.020 (from 30.613 to 95.322)
Positive predictive value	0.724 (from 0.653 to 0.785)
Negative predictive value	0.954 (from 0.929 to 0.970)
Positive likelihood ratio	7.947 (from 6.040 to 10.456)
Negative likelihood ratio	0.147 (from 0.097 to 0.224)
**Class geniculatus**	
Apparent prevalence	0.265 (from 0.231 to 0.303)
True prevalence	0.248 (from 0.215 to 0.285)
Sensitivity	0.855 (from 0.789 to 0.903)
Specificity	0.929 (from 0.902 to 0.950)
Correctly classified proportion	0.911 (from 0.885 to 0.931)
Diagnostic odds ratio	77.714 (from 43.111 to 140.093)
Positive predictive value	0.800 (from 0.730 to 0.855)
Negative predictive value	0.951 (from 0.926 to 0.968)
Positive likelihood ratio	12.110 (from 8.569 to 17.115)
Negative likelihood ratio	0.156 (from 0.105 to 0.232)
**Class japonicus**	
Apparent prevalence	0.255 (from 0.221 to 0.292)
True prevalence	0.250 (from 0.217 to 0.287)
Sensitivity	0.562 (from 0.481 to 0.640)
Specificity	0.847 (from 0.810 to 0.878)
Correctly classified proportion	0.776 (from 0.740 to 0.808)
Diagnostic odds ratio	7.095 (from 4.672 to 10.774)
Positive predictive value	0.550 (from 0.470 to 0.628)
Negative predictive value	0.853 (from 0.816 to 0.883)
Positive likelihood ratio	3.672 (from 2.823 to 4.776)
Negative likelihood ratio	0.518 (from 0.429 to 0.624)
**Class koreicus**	
Apparent prevalence	0.182 (from 0.152 to 0.215)
True prevalence	0.253 (from 0.220 to 0.290)
Sensitivity	0.561 (from 0.480 to 0.638)
Specificity	0.947 (from 0.922 to 0.965)
Correctly classified proportion	0.849 (from 0.818 to 0.876)
Diagnostic odds ratio	22.929 (from 13.486 to 38.984)
Positive predictive value	0.783 (from 0.695 to 0.851)
Negative predictive value	0.864 (from 0.830 to 0.892)
Positive likelihood ratio	10.631 (from 6.967 to 16.221)
Negative likelihood ratio	0.464 (from 0.386 to 0.557)

We save the created objects in a list.

```
df.groups_list_dataset[["Rater level expert"]] <- `d.disc.eggs.l_Rater level expert`
df.groups_list_diag.measures[["Rater level expert"]] <- res.diagnostic.measures
```

We clean up the programming environment.

```
rm(tab, nr.obs, l.confusion.matrix,
   l.confusion.matrix.print, res.diagnostic.measures,
   aux.df.rows)
```

7.6 We look at *Rater level non expert*

7.6.1 Dataset

```
## Dataset
`d.disc.eggs.l_Rater level non expert` <- d.disc.eggs.l %>% filter(`group` == "Rater level non expert")
## Number of observations
(nr.obs <- nrow(`d.disc.eggs.l_Rater level non expert`))
```

```
[1] 120
```

This dataset contains 120 observations.

7.6.2 Missing values

Next, we give an overview of the missing values.

```
`d.disc.eggs.l_Rater level non expert` %>%
  select(`answer.rater`, `MALDI-TOF determination`) %>%
  table(useNA = "always")
```

```
             MALDI-TOF determination
answer.rater  albopictus geniculatus japonicus koreicus <NA>
  albopictus          29           2         4        2    0
  geniculatus          1          27         2        0    0
  japonicus            0           1        21        9    0
  koreicus             0           0         3       17    0
  <NA>                 0           0         0        2    0
```

```
## Calculate the number of missing values in both index/reference test:
nr.NA <- `d.disc.eggs.l_Rater level non expert` %>%
  select(`answer.rater`, `MALDI-TOF determination`) %>%
  (\(x){sum(is.na(x))})
## Calculate the number of observations that will be removed:
(nr.deleted.obs <- `d.disc.eggs.l_Rater level non expert` %>%
    filter(is.na(`answer.rater`) | is.na(`MALDI-TOF determination`)) %>%
    nrow())
```

```
[1] 2
```

```
`d.disc.eggs.l_Rater level non expert` <- `d.disc.eggs.l_Rater level non expert` %>%
  filter(!is.na(`answer.rater`) & !is.na(`MALDI-TOF determination`))
```

There were 2 observations (rows) deleted due to missing values.

7.6.3 Confusion Matrix

We calculate the confusion matrices in the following steps.

```
tab <- xtabs(formula = ~ `answer.rater` + `MALDI-TOF determination`,
             data = `d.disc.eggs.l_Rater level non expert`)
tab %>%
  f.create.confusion.matrix() %>%
  as_tibble(rownames = "answer.rater") %>%
  kable(caption = paste0("Overall cross table."),
        label = "tab:CrossTable6",
```

```
        booktabs = TRUE,
        longtable = TRUE,
        linesep = c("")) %>%
  kable_styling(font_size = 7,
                latex_options = c("striped", "repeat_header", "hold_position")) %>%
  row_spec(row = 5, bold = TRUE) %>%
  column_spec(column = 6, bold = TRUE) %>%
  add_header_above(c("", "MALDI-TOF determination" = 4, ""))
```

Table 37: Overall cross table.

	MALDI-TOF determination				
answer.rater	albopictus	geniculatus	japonicus	koreicus	Total
albopictus	29	2	4	2	**37**
geniculatus	1	27	2	0	**30**
japonicus	0	1	21	9	**31**
koreicus	0	0	3	17	**20**
**Total**	**30**	**30**	**30**	**28**	**118**

Example how to read this table:

- There were 29 cases of *albopictus* that were correctly classified as *albopictus*, 1 cases of *albopictus* were wrongly classified as *geniculatus*.
- There were 27 cases of *geniculatus* that were correctly classified as *geniculatus*, 2 cases of *geniculatus* were wrongly classified as *albopictus*.

We present the same table in percentages. Note that all columns add to 100%.

```
tab <- xtabs(formula = ~ `answer.rater` + `MALDI-TOF determination`,
             data = `d.disc.eggs.l_Rater level non expert`)
prop.table(x = tab, margin = 2) %>%
  '*'(100) %>%
  rbind(., colSums(.)) %>%
  f.round(digits = digits.rounding) %>%
  kable(caption = paste0("Overall cross table in percentages. All columns add up to 100\\%."),
        label = "tab:CrossTablePercentage6",
        booktabs = TRUE,
        longtable = TRUE,
        linesep = c("")) %>%
  kable_styling(font_size = 7,
                latex_options = c("striped", "repeat_header", "hold_position")) %>%
  row_spec(row = 5, bold = TRUE) %>%
  add_header_above(c("", "MALDI-TOF determination" = 4))
```

Table 38: Overall cross table in percentages. All columns add up to 100%.

	MALDI-TOF determination			
	albopictus	geniculatus	japonicus	koreicus
albopictus	96.67	6.67	13.33	7.14
geniculatus	3.33	90.00	6.67	0.00
japonicus	0.00	3.33	70.00	32.14
koreicus	0.00	0.00	10.00	60.71
	**100.00**	**100.00**	**100.00**	**100.00**

```
levels.chr <- levels(`d.disc.eggs.l_Rater level non expert`$`answer.rater`)
if(length(levels.chr) == 2){
```

```
  classes <- c("")
}else{
  classes <- levels.chr
}

## calculate tables
if(identical(classes, c(""))){
  l.tab <- list(xtabs(formula = ~ `answer.rater` + `MALDI-TOF determination`,
                      data = `d.disc.eggs.l_Rater level non expert`))
}else{
  l.tab <- lapply(X = classes, FUN = function(x){
    xtabs(formula = ~ `answer.rater` + `MALDI-TOF determination`,
          data = `d.disc.eggs.l_Rater level non expert` %>%
            mutate(across(.cols = c(`answer.rater`, `MALDI-TOF determination`), .fns = function(y){
              ## we merge categories into the category "neg."
              y %>%
                fct_collapse("neg." = setdiff(levels(`d.disc.eggs.l_Rater level non expert`$`answer.rat
                fct_relevel(x)
            })))
  })
}
## calculate confusion matrix
l.confusion.matrix <- lapply(X = l.tab,
                             FUN = f.create.confusion.matrix)
## calculate tables for printing
l.confusion.matrix.print <- lapply(X = l.confusion.matrix,
                                   FUN = f.create.confusion.matrix.print)
```

The next tables show the confusion matrices for the index test ("answer.rater") against the reference test ("MALDI-TOF determination").

```
for(class in seq_along(classes)){
  l.confusion.matrix.print[[class]] %>%
    as_tibble(rownames = "answer.rater") %>%
    kable(caption = paste0("Confusion matrix for class ‘",
                           levels(`d.disc.eggs.l_Rater level non expert`$`answer.rater`)[class], "’.
          Category ‘", levels(`d.disc.eggs.l_Rater level non expert`$`answer.rater`)[class],
          "’ is considered a positive result.
          Category ‘neg.’ is considered a negative result and
          contains the levels ",
          paste0(setdiff(levels(`d.disc.eggs.l_Rater level non expert`$`answer.rater`),
                         classes[class]), collapse = ", "), "."),
          label = paste0("tab:ConfusionMatrix6Class", class),
          booktabs = TRUE,
          longtable = TRUE,
          linesep = c("")) %>%
    kable_styling(font_size = 7,
                  latex_options = c("striped", "repeat_header", "hold_position")) %>%
    row_spec(row = nrow(l.confusion.matrix.print[[class]]), bold = TRUE) %>%
    column_spec(column = ncol(l.confusion.matrix.print[[class]]) + 1, bold = TRUE) %>%
    add_header_above(c("", "MALDI-TOF determination" =
                         ncol(l.confusion.matrix.print[[class]]) - 1, "")) %>%
    print()
```

```
}
```

Table 39: Confusion matrix for class 'albopictus'. Category 'albopictus' is considered a positive result. Category 'neg.' is considered a negative result and contains the levels geniculatus, japonicus, koreicus.

	MALDI-TOF determination		
answer.rater	albopictus	neg.	Total
albopictus	29 (TP)	8 (FP)	**37**
neg.	1 (FN)	80 (TN)	**81**
**Total**	**30**	**88**	**118**

Table 40: Confusion matrix for class 'geniculatus'. Category 'geniculatus' is considered a positive result. Category 'neg.' is considered a negative result and contains the levels albopictus, japonicus, koreicus.

	MALDI-TOF determination		
answer.rater	geniculatus	neg.	Total
geniculatus	27 (TP)	3 (FP)	**30**
neg.	3 (FN)	85 (TN)	**88**
**Total**	**30**	**88**	**118**

Table 41: Confusion matrix for class 'japonicus'. Category 'japonicus' is considered a positive result. Category 'neg.' is considered a negative result and contains the levels albopictus, geniculatus, koreicus.

	MALDI-TOF determination		
answer.rater	japonicus	neg.	Total
japonicus	21 (TP)	10 (FP)	**31**
neg.	9 (FN)	78 (TN)	**87**
**Total**	**30**	**88**	**118**

Table 42: Confusion matrix for class 'koreicus'. Category 'koreicus' is considered a positive result. Category 'neg.' is considered a negative result and contains the levels albopictus, geniculatus, japonicus.

	MALDI-TOF determination		
answer.rater	koreicus	neg.	Total
koreicus	17 (TP)	3 (FP)	**20**
neg.	11 (FN)	87 (TN)	**98**
**Total**	**28**	**90**	**118**

7.6.4 Measures of diagnostic accuracy

We now calculate measures of diagnostic accuracy.

```
res.diagnostic.measures <- tibble("Class" = classes) %>%
  mutate(tabs = l.tab) %>%

  ## epiR wants it in the format: c(TP, FP, FN, TN)
  mutate(confusion.vector = map(.x = tabs, .f = function(x){
    c("tp" = x[1, 1], "fp" = x[1, 2], "fn" = x[2, 1], "tn" = x[2, 2])
  })) %>%

  ## Calculate measures of diagnostic accuracy
  mutate(out.EpiR = map(.x = confusion.vector, .f = function(x){
    epi.tests(dat = x,
              method = "wilson",
              conf.level = 0.95)
  })) %>%

  ## reformat output, filter diag. measures of interest
  mutate(diag.measures = map(.x = out.EpiR, .f = function(x){
    x <- x %>%
      summary() %>%
      as_tibble(rownames = "name.raw") %>%
      mutate()
    if(f.check.version(x = packageVersion(pkg = "epiR"))){
      x <- x %>%
        mutate(name.raw = statistic) %>%
        select(-statistic)
    }
    x %>%
      left_join(y = .match, by = c("name.raw")) %>%
      filter(name.raw %in% c("ap", "tp", "se", "sp", "diag.or", "pv.pos", "pv.neg", "lr.pos", "lr.neg",
  })) %>%

  ## ensure boundaries of estimates and CI
  mutate(diag.measures = map(.x = diag.measures, .f = function(x){
    x %>%
      mutate(across(.cols = c(est, lower, upper), .fns = function(x){
        if_else(condition = bounded, true = pmin(1, pmax(0, x)), false = x)
      }))
  })) %>%

  ## put together output
  mutate(diag.measures.print = map(.x = diag.measures, .f = function(x){
    x %>%
      mutate(across(.cols = c("est", "lower", "upper"),
                    .fns = \(x){format(x = round(x = x, digits = digits.rounding + 1),
                                       scientific = FALSE,
                                       nsmall = digits.rounding + 1)})) %>%
      mutate("Estimate (95% CI)" = paste0(est, " (from ", lower, " to ", upper, ")")) %>%
      select(Measure, `Estimate (95% CI)`)
  }))
```

We restructure the results that are to be printed.

```
aux.df.rows <- res.diagnostic.measures %>%
  select(Class, diag.measures.print) %>%
  mutate(Class = paste0("Class ", Class)) %>%
  unnest(cols = diag.measures.print) %>%
  mutate(row.nr = seq_len(n())) %>%
  group_by(Class) %>%
  summarize(min = min(row.nr),
            max = max(row.nr)) %>%
  ungroup()
```

We prepare the printing of the table.

```
res.diagnostic.measures_print6 <- res.diagnostic.measures %>%
  select(Class, diag.measures.print) %>%
  unnest(cols = diag.measures.print) %>%
  select(-Class) %>%

  kable(caption = paste0("Estimate and 95\\% confidence intervals for prevalence
                         and different measures of accuracy."),
        label = "tab:ResultTable6",
        booktabs = TRUE,
        longtable = TRUE,
        linesep = c("")) %>%
  kable_styling(
    font_size = 7,
    repeat_header_method = "replace",
    repeat_header_text = paste0("Estimate and 95\\% confidence intervals for prevalence
                                and different measures of accuracy. (continued)"),
    latex_options = c("striped", "repeat_header", "hold_position")
  ) %>%

  group_rows(group_label = aux.df.rows$Class[1],
             start_row = aux.df.rows$min[1],
             end_row = aux.df.rows$max[1]) %>%
  group_rows(group_label = aux.df.rows$Class[2],
             start_row = aux.df.rows$min[2],
             end_row = aux.df.rows$max[2]) %>%
  group_rows(group_label = aux.df.rows$Class[3],
             start_row = aux.df.rows$min[3],
             end_row = aux.df.rows$max[3]) %>%
  group_rows(group_label = aux.df.rows$Class[4],
             start_row = aux.df.rows$min[4],
             end_row = aux.df.rows$max[4]) %>%
  collapse_rows(columns = 1, latex_hline = "major", valign = "middle")
```

The next table shows point estimate and 95% confidence intervals for prevalence and different measures of diagnostic accuracy.

```
res.diagnostic.measures_print6
```

Table 43: Estimate and 95% confidence intervals for prevalence and different measures of accuracy.

Measure	Estimate (95% CI)
**Class albopictus**	
Apparent prevalence	0.314 (from 0.237 to 0.402)

Table 43: Estimate and 95% confidence intervals for prevalence and different measures of accuracy. (continued)

Measure	Estimate (95% CI)
True prevalence	0.254 (from 0.184 to 0.340)
Sensitivity	0.967 (from 0.833 to 0.994)
Specificity	0.909 (from 0.831 to 0.953)
Correctly classified proportion	0.924 (from 0.861 to 0.959)
Diagnostic odds ratio	290.000 (from 34.746 to 2420.433)
Positive predictive value	0.784 (from 0.628 to 0.886)
Negative predictive value	0.988 (from 0.933 to 0.998)
Positive likelihood ratio	10.633 (from 5.474 to 20.657)
Negative likelihood ratio	0.037 (from 0.005 to 0.252)
**Class geniculatus**	
Apparent prevalence	0.254 (from 0.184 to 0.340)
True prevalence	0.254 (from 0.184 to 0.340)
Sensitivity	0.900 (from 0.744 to 0.965)
Specificity	0.966 (from 0.905 to 0.988)
Correctly classified proportion	0.949 (from 0.893 to 0.976)
Diagnostic odds ratio	255.000 (from 48.590 to 1338.234)
Positive predictive value	0.900 (from 0.744 to 0.965)
Negative predictive value	0.966 (from 0.905 to 0.988)
Positive likelihood ratio	26.400 (from 8.627 to 80.792)
Negative likelihood ratio	0.104 (from 0.035 to 0.303)
**Class japonicus**	
Apparent prevalence	0.263 (from 0.192 to 0.349)
True prevalence	0.254 (from 0.184 to 0.340)
Sensitivity	0.700 (from 0.521 to 0.833)
Specificity	0.886 (from 0.803 to 0.937)
Correctly classified proportion	0.839 (from 0.762 to 0.894)
Diagnostic odds ratio	18.200 (from 6.554 to 50.540)
Positive predictive value	0.677 (from 0.501 to 0.814)
Negative predictive value	0.897 (from 0.815 to 0.945)
Positive likelihood ratio	6.160 (from 3.285 to 11.552)
Negative likelihood ratio	0.338 (from 0.195 to 0.588)
**Class koreicus**	
Apparent prevalence	0.169 (from 0.112 to 0.247)
True prevalence	0.237 (from 0.170 to 0.322)
Sensitivity	0.607 (from 0.424 to 0.764)
Specificity	0.967 (from 0.907 to 0.989)
Correctly classified proportion	0.881 (from 0.811 to 0.928)
Diagnostic odds ratio	44.818 (from 11.294 to 177.853)
Positive predictive value	0.850 (from 0.640 to 0.948)
Negative predictive value	0.888 (from 0.810 to 0.936)
Positive likelihood ratio	18.214 (from 5.757 to 57.626)
Negative likelihood ratio	0.406 (from 0.256 to 0.645)

We save the created objects in a list.

```
df.groups_list_dataset[["Rater level non expert"]] <- `d.disc.eggs.l_Rater level non expert`
df.groups_list_diag.measures[["Rater level non expert"]] <- res.diagnostic.measures
```

We clean up the programming environment.

```
rm(tab, nr.obs, l.confusion.matrix,
   l.confusion.matrix.print, res.diagnostic.measures,
   aux.df.rows)
```

8 Per person

We calculate the different measures of diagnostic accuracy also on per person level. We will not present the individual confusion matrices but only the estimates and confidence intervals of the diagnostic accuracy measures since this would result in a very large output.

First we extract from the longitudinal dataset the 'Overall' dataset containing the information in the basic form.

```
d.disc.eggs <- d.disc.eggs.l %>%
  filter(type == "Overall")

## checks
head(d.disc.eggs)
```

```
# A tibble: 6 x 27
  id    question filename   answe~1   CNT Pictu~2 Pictu~3 Canton egg n~4 optic~5
  <fct>    <int> <chr>      <fct>   <dbl> <chr>   <chr>   <chr>  <chr>   <chr>
1 1            1 Workshop2~ albopi~    73 A12     A12_GR~ Griso~ GR-RVD~ albopi~
2 1            2 Workshop2~ japoni~    17 J10     J10_TI~ Ticino TI-GRV~ japoni~
3 1            3 Workshop2~ koreic~   222 K58     K58_TI~ Ticino TI-MOI~ koreic~
4 1            4 Workshop2~ genicu~   317 G19     G19_GR~ Griso~ GR-RVD~ genicu~
5 1            5 Workshop2~ genicu~   303 G05     G05_VD~ Vaud   VD-AIG~ genicu~
6 1            6 Workshop2~ japoni~    30 J14     J14_TI~ Ticino TI-TTO~ japoni~
# ... with 17 more variables: `Chorion quality` <fct>, `File MALDI-TOF` <chr>,
#   `MALDI-TOF determination` <fct>, `%` <chr>, `obs - light green star` <chr>,
#   count <dbl>, ...17 <chr>, `Country (in capitals)` <chr>,
#   `name of questionnaire` <chr>, name.of.questionnaire.modified <chr>,
#   `time spent` <dttm>, group.expert <fct>, exclude.modified <dbl>,
#   time_minutes <dbl>, comment <chr>, type <chr>, group <chr>, and abbreviated
#   variable names 1: answer.rater, 2: Picture_ID, 3: `Picture file name`, ...
```

```
dim(d.disc.eggs)
```

```
[1] 720  27
```

```
str(d.disc.eggs)
```

```
tibble [720 x 27] (S3: tbl_df/tbl/data.frame)
 $ id                                  : Factor w/ 30 levels "1","2","3","4",..: 1 1 1 1 1 1 1 1 1 1 .
 $ question                            : int [1:720] 1 2 3 4 5 6 7 8 9 10 ...
 $ filename                            : chr [1:720] "Workshop2/PDFs/Report_participant1.pdf" "Worksho
 $ answer.rater                        : Factor w/ 4 levels "albopictus","geniculatus",..: 1 3 4 2 2 3
 $ CNT                                 : num [1:720] 73 17 222 317 303 30 165 229 172 27 ...
 $ Picture_ID                          : chr [1:720] "A12" "J10" "K58" "G19" ...
 $ Picture file name                   : chr [1:720] "A12_GR-RVD-017a_c_1" "J10_TI-GRV-002_c_3" "K58_T
 $ Canton                              : chr [1:720] "Grisons" "Ticino" "Ticino" "Grisons" ...
 $ egg name                            : chr [1:720] "GR-RVD-017a_c_1" "TI-GRV-002_c_3" "TI-MOI-009b_a
 $ optical determination by the operator: chr [1:720] "albopictus" "japonicus" "koreicus" "geniculatus"
 $ Chorion quality                     : Factor w/ 3 levels "high","medium",..: 1 3 3 2 1 2 2 3 1 3 ..
 $ File MALDI-TOF                      : chr [1:720] "LMA_Zanz2020_0013_1F4[c]" "LMA_Zanz2020_0011_1C2
 $ MALDI-TOF determination             : Factor w/ 4 levels "albopictus","geniculatus",..: 1 3 4 2 2 3
 $ %                                   : chr [1:720] "99.9" "99.9" "99.9" "99.9" ...
 $ obs - light green star              : chr [1:720] "no" "no" "no" "yes" ...
 $ count                               : num [1:720] 1 1 1 1 1 1 1 1 1 1 ...
 $ ...17                               : chr [1:720] "1" "1" "1" "1" ...
```

```
 $ Country (in capitals)                 : chr [1:720] "AUSTRIA" "AUSTRIA" "AUSTRIA" "AUSTRIA" ...
 $ name of questionnaire                 : chr [1:720] "Report_participant1" "Report_participant1" "Repo
 $ name.of.questionnaire.modified        : chr [1:720] "Report_participant1" "Report_participant1" "Repo
 $ time spent                            : POSIXct[1:720], format: "1899-12-31 00:09:25" "1899-12-31 00:
 $ group.expert                          : Factor w/ 2 levels "expert","non expert": 1 1 1 1 1 1 1 1 1 1
 $ exclude.modified                      : num [1:720] 0 0 0 0 0 0 0 0 0 0 ...
 $ time_minutes                          : num [1:720] 9.42 9.42 9.42 9.42 9.42 ...
 $ comment                               : chr [1:720] NA NA NA NA ...
 $ type                                  : chr [1:720] "Overall" "Overall" "Overall" "Overall" ...
 $ group                                 : chr [1:720] "Overall" "Overall" "Overall" "Overall" ...
```

```r
## ok
```

Note that the analysis on person level is still balanced w.r.t. to the species. We briefly repeat it here.

```r
xtabs(formula = ~ id + `MALDI-TOF determination`,
      data = d.disc.eggs)
```

```
    MALDI-TOF determination
id   albopictus geniculatus japonicus koreicus
  1           6           6         6        6
  2           6           6         6        6
  3           6           6         6        6
  4           6           6         6        6
  5           6           6         6        6
  6           6           6         6        6
  7           6           6         6        6
  8           6           6         6        6
  9           6           6         6        6
  10          6           6         6        6
  11          6           6         6        6
  12          6           6         6        6
  13          6           6         6        6
  14          6           6         6        6
  15          6           6         6        6
  16          6           6         6        6
  17          6           6         6        6
  18          6           6         6        6
  19          6           6         6        6
  20          6           6         6        6
  21          6           6         6        6
  22          6           6         6        6
  23          6           6         6        6
  24          6           6         6        6
  25          6           6         6        6
  26          6           6         6        6
  27          6           6         6        6
  28          6           6         6        6
  29          6           6         6        6
  30          6           6         6        6
```

Further it is also balanced w.r.t. to the quality of the images.

```r
xtabs(formula = ~ id + `MALDI-TOF determination` + `Chorion quality`,
      data = d.disc.eggs)
```

, , Chorion quality = high

	MALDI-TOF determination			
id	albopictus	geniculatus	japonicus	koreicus
1	2	2	2	2
2	2	2	2	2
3	2	2	2	2
4	2	2	2	2
5	2	2	2	2
6	2	2	2	2
7	2	2	2	2
8	2	2	2	2
9	2	2	2	2
10	2	2	2	2
11	2	2	2	2
12	2	2	2	2
13	2	2	2	2
14	2	2	2	2
15	2	2	2	2
16	2	2	2	2
17	2	2	2	2
18	2	2	2	2
19	2	2	2	2
20	2	2	2	2
21	2	2	2	2
22	2	2	2	2
23	2	2	2	2
24	2	2	2	2
25	2	2	2	2
26	2	2	2	2
27	2	2	2	2
28	2	2	2	2
29	2	2	2	2
30	2	2	2	2

, , Chorion quality = medium

	MALDI-TOF determination			
id	albopictus	geniculatus	japonicus	koreicus
1	2	2	2	2
2	2	2	2	2
3	2	2	2	2
4	2	2	2	2
5	2	2	2	2
6	2	2	2	2
7	2	2	2	2
8	2	2	2	2
9	2	2	2	2
10	2	2	2	2
11	2	2	2	2
12	2	2	2	2
13	2	2	2	2
14	2	2	2	2
15	2	2	2	2

```
  16           2            2          2        2
  17           2            2          2        2
  18           2            2          2        2
  19           2            2          2        2
  20           2            2          2        2
  21           2            2          2        2
  22           2            2          2        2
  23           2            2          2        2
  24           2            2          2        2
  25           2            2          2        2
  26           2            2          2        2
  27           2            2          2        2
  28           2            2          2        2
  29           2            2          2        2
  30           2            2          2        2

, , Chorion quality = low

    MALDI-TOF determination
id   albopictus geniculatus japonicus koreicus
  1            2            2          2        2
  2            2            2          2        2
  3            2            2          2        2
  4            2            2          2        2
  5            2            2          2        2
  6            2            2          2        2
  7            2            2          2        2
  8            2            2          2        2
  9            2            2          2        2
  10           2            2          2        2
  11           2            2          2        2
  12           2            2          2        2
  13           2            2          2        2
  14           2            2          2        2
  15           2            2          2        2
  16           2            2          2        2
  17           2            2          2        2
  18           2            2          2        2
  19           2            2          2        2
  20           2            2          2        2
  21           2            2          2        2
  22           2            2          2        2
  23           2            2          2        2
  24           2            2          2        2
  25           2            2          2        2
  26           2            2          2        2
  27           2            2          2        2
  28           2            2          2        2
  29           2            2          2        2
  30           2            2          2        2
```

We use the same computing machinery as before.

We set up the usual variables used in the previous calculations.

```r
levels.chr <- levels(`d.disc.eggs`$`answer.rater`)
(classes <- levels.chr)
```

```
[1] "albopictus"  "geniculatus" "japonicus"   "koreicus"
```

We want to calculate all measures of diagnostic accuracy for each individual rater. For that we nest for *id* which represents the id of a rater. This means that we obtain for each rater id a dataset containing all necessary information to calculate the different measures. The idea is to apply the computational framework we used above on each created dataset via the *map()* function which is, roughly speaking, like a for-loop.

```r
d.disc.eggs_person <- d.disc.eggs %>%

  ## we include the variable *group.expert* since this is perfectly corresponds to the
  ## id and we can use this variable later on
  group_by(id, group.expert) %>%
  nest() %>%
  ungroup() %>%

  print()
```

```
# A tibble: 30 x 3
   id    group.expert data
   <fct> <fct>        <list>
 1 1     expert       <tibble [24 x 25]>
 2 2     expert       <tibble [24 x 25]>
 3 3     expert       <tibble [24 x 25]>
 4 4     non expert   <tibble [24 x 25]>
 5 5     expert       <tibble [24 x 25]>
 6 6     expert       <tibble [24 x 25]>
 7 7     expert       <tibble [24 x 25]>
 8 8     expert       <tibble [24 x 25]>
 9 9     expert       <tibble [24 x 25]>
10 10    expert       <tibble [24 x 25]>
# ... with 20 more rows
```

We now generate a confusion matrix, for each id-dataset separately.

```r
d.disc.eggs_person <- d.disc.eggs_person %>%

  mutate(confusion.mat = map(.x = data, .f = \(i.data){

    l.tab <- lapply(X = classes, FUN = function(x){
      xtabs(formula = ~ `answer.rater` + `MALDI-TOF determination`,
            data = i.data %>%
              mutate(across(.cols = c(`answer.rater`, `MALDI-TOF determination`), .fns = function(y){
                ## we merge categories into the category "neg."
                y %>%
                  fct_collapse("neg." = setdiff(levels(i.data$`answer.rater`), x)) %>%
                  fct_relevel(x)
              })))
    })

    return(l.tab)
  }))

## checks:
```

```
d.disc.eggs_person
```

```
# A tibble: 30 x 4
   id    group.expert data                confusion.mat
   <fct> <fct>        <list>              <list>
 1 1     expert       <tibble [24 x 25]> <list [4]>
 2 2     expert       <tibble [24 x 25]> <list [4]>
 3 3     expert       <tibble [24 x 25]> <list [4]>
 4 4     non expert   <tibble [24 x 25]> <list [4]>
 5 5     expert       <tibble [24 x 25]> <list [4]>
 6 6     expert       <tibble [24 x 25]> <list [4]>
 7 7     expert       <tibble [24 x 25]> <list [4]>
 8 8     expert       <tibble [24 x 25]> <list [4]>
 9 9     expert       <tibble [24 x 25]> <list [4]>
10 10    expert       <tibble [24 x 25]> <list [4]>
# ... with 20 more rows
```

```
## checks: we look at the confusion matrices for id 1, i.e. for all 4 species
d.disc.eggs_person[1, "confusion.mat"][[1]]
```

```
[[1]]
[[1]][[1]]
            MALDI-TOF determination
answer.rater albopictus neg.
  albopictus          6    0
  neg.                0   18

[[1]][[2]]
             MALDI-TOF determination
answer.rater  geniculatus neg.
  geniculatus           6    0
  neg.                  0   18

[[1]][[3]]
            MALDI-TOF determination
answer.rater japonicus neg.
   japonicus         6    3
   neg.              0   15

[[1]][[4]]
            MALDI-TOF determination
answer.rater koreicus neg.
    koreicus        3    0
    neg.            3   18
```

```
## looks good
```

In the next step, we calculate the actual measures of diagnostic accuracy. The individual steps are:

- 1) Extract from the confusion matrices the vector with true positive, false postive, false negative and true negative, since the function *epi.tests()* from package *epiR* needs it in that format.
- 2) Apply the function *epi.tests()*.
- 3) Reformatting/cleaning the output from *epi.tests()*.
- 4) Ensuring that the boundaries of the confidence intervals are within 0 and 1.

- 5) Reformatting the output for printing (e.g. rounding).

```
d.disc.eggs_person <- d.disc.eggs_person %>%
  mutate(res.diag.measures = map(.x = confusion.mat, .f = \(i.confusion.mat){


    res.diagnostic.measures <- tibble("Class" = classes) %>%
      mutate(tabs = i.confusion.mat) %>%

      ## 1) epiR wants it in the format: c(TP, FP, FN, TN)
      mutate(confusion.vector = map(.x = tabs, .f = function(x){
        c("tp" = x[1, 1], "fp" = x[1, 2], "fn" = x[2, 1], "tn" = x[2, 2])
      })) %>%

      ## 2) Calculate measures of diagnostic accuracy
      mutate(out.EpiR = map(.x = confusion.vector, .f = function(x){
        epi.tests(dat = x,
                  method = "wilson",
                  conf.level = 0.95)
      })) %>%

      ## 3) reformat output, filter diag. measures of interest
      mutate(diag.measures = map(.x = out.EpiR, .f = function(x){
        x <- x %>%
          summary() %>%
          as_tibble(rownames = "name.raw") %>%
          mutate()
        if(f.check.version(x = packageVersion(pkg = "epiR"))){
          x <- x %>%
            mutate(name.raw = statistic) %>%
            select(-statistic)
        }
        x %>%
          left_join(y = .match, by = c("name.raw")) %>%
          filter(name.raw %in% c("ap", "tp", "se", "sp", "diag.or", "pv.pos", "pv.neg", "lr.pos", "lr.n[illegible]
      })) %>%

      ## 4) ensure boundaries of estimates and CI
      mutate(diag.measures = map(.x = diag.measures, .f = function(x){
        x %>%
          mutate(across(.cols = c(est, lower, upper), .fns = function(x){
            if_else(condition = bounded, true = pmin(1, pmax(0, x)), false = x)
          }))
      })) %>%


      ## 5) put together output
      mutate(diag.measures.print = map(.x = diag.measures, .f = function(x){
        x %>%
          mutate(across(.cols = c("est", "lower", "upper"),
                        .fns = \(x){format(x = round(x = x, digits = digits.rounding + 1),
                                           scientific = FALSE,
                                           nsmall = digits.rounding + 1)})) %>%
          mutate("Estimate (95% CI)" = paste0(est, " (from ", lower, " to ", upper, ")")) %>%
```

```
        select(Measure, `Estimate (95% CI)`)
    }))


    return(res.diagnostic.measures)
  }))
```

We now can unnest the results

```
## unnest on id-level
d.disc.eggs_person.un <- d.disc.eggs_person %>%
  unnest(cols = res.diag.measures) %>%
  print()
```

```
# A tibble: 120 x 10
   id    group~1 data     confu~2 Class tabs         confu~3 out.EpiR   diag.m~4
   <fct> <fct>   <list>   <list>  <chr> <list>       <list>  <list>     <list>
 1 1     expert  <tibble> <list>  albo~ <xtabs[...]> <int>   <epi.tsts> <tibble>
 2 1     expert  <tibble> <list>  geni~ <xtabs[...]> <int>   <epi.tsts> <tibble>
 3 1     expert  <tibble> <list>  japo~ <xtabs[...]> <int>   <epi.tsts> <tibble>
 4 1     expert  <tibble> <list>  kore~ <xtabs[...]> <int>   <epi.tsts> <tibble>
 5 2     expert  <tibble> <list>  albo~ <xtabs[...]> <int>   <epi.tsts> <tibble>
 6 2     expert  <tibble> <list>  geni~ <xtabs[...]> <int>   <epi.tsts> <tibble>
 7 2     expert  <tibble> <list>  japo~ <xtabs[...]> <int>   <epi.tsts> <tibble>
 8 2     expert  <tibble> <list>  kore~ <xtabs[...]> <int>   <epi.tsts> <tibble>
 9 3     expert  <tibble> <list>  albo~ <xtabs[...]> <int>   <epi.tsts> <tibble>
10 3     expert  <tibble> <list>  geni~ <xtabs[...]> <int>   <epi.tsts> <tibble>
# ... with 110 more rows, 1 more variable: diag.measures.print <list>, and
#   abbreviated variable names 1: group.expert, 2: confusion.mat,
#   3: confusion.vector, 4: diag.measures
```

```
## unnest on diagnostic-accuracy-measure-level
d.disc.eggs_person.un <- d.disc.eggs_person.un %>%
  unnest(cols = diag.measures) %>%
  print()
```

```
# A tibble: 1,200 x 15
   id    group.~1 data     confu~2 Class tabs         confu~3 out.EpiR   name.~4
   <fct> <fct>    <list>   <list>  <chr> <list>       <list>  <list>     <chr>
 1 1     expert   <tibble> <list>  albo~ <xtabs[...]> <int>   <epi.tsts> ap
 2 1     expert   <tibble> <list>  albo~ <xtabs[...]> <int>   <epi.tsts> tp
 3 1     expert   <tibble> <list>  albo~ <xtabs[...]> <int>   <epi.tsts> se
 4 1     expert   <tibble> <list>  albo~ <xtabs[...]> <int>   <epi.tsts> sp
 5 1     expert   <tibble> <list>  albo~ <xtabs[...]> <int>   <epi.tsts> diag.ac
 6 1     expert   <tibble> <list>  albo~ <xtabs[...]> <int>   <epi.tsts> diag.or
 7 1     expert   <tibble> <list>  albo~ <xtabs[...]> <int>   <epi.tsts> pv.pos
 8 1     expert   <tibble> <list>  albo~ <xtabs[...]> <int>   <epi.tsts> pv.neg
 9 1     expert   <tibble> <list>  albo~ <xtabs[...]> <int>   <epi.tsts> lr.pos
10 1     expert   <tibble> <list>  albo~ <xtabs[...]> <int>   <epi.tsts> lr.neg
# ... with 1,190 more rows, 6 more variables: est <dbl>, lower <dbl>,
#   upper <dbl>, Measure <chr>, bounded <lgl>, diag.measures.print <list>, and
#   abbreviated variable names 1: group.expert, 2: confusion.mat,
#   3: confusion.vector, 4: name.raw
```

The amount of information is very large, we therefore directly present it in a figure. We further only show

measures that are bounded (e.g. sensitivity, specificity, etc.)

```
d.disc.eggs_person.un %>%
  mutate(Class = paste0("Ae. ", Class)) %>%
  filter(bounded) %>%
  filter( ! (Measure %in% c("Apparent prevalence", "True prevalence"))) %>%

  ggplot(aes(x = reorder(id, est), y = est)) +
  aes(color = group.expert) +

  facet_grid(Measure ~ Class, scales = "free", labeller = label_wrap_gen()) +
  geom_point() +
  geom_hline(yintercept = c(0, 1), linetype = "dashed") +
  geom_errorbar(aes(ymin = lower, ymax = upper)) +
  theme(legend.position = "bottom") +
  scale_x_discrete(guide = guide_axis(n.dodge = 3)) +
  theme(axis.text.x = element_blank()) + ## to remove the IDs (for publication)
  labs(x = "",
       y = "Estimate and 95% CI",
       color = "")
```

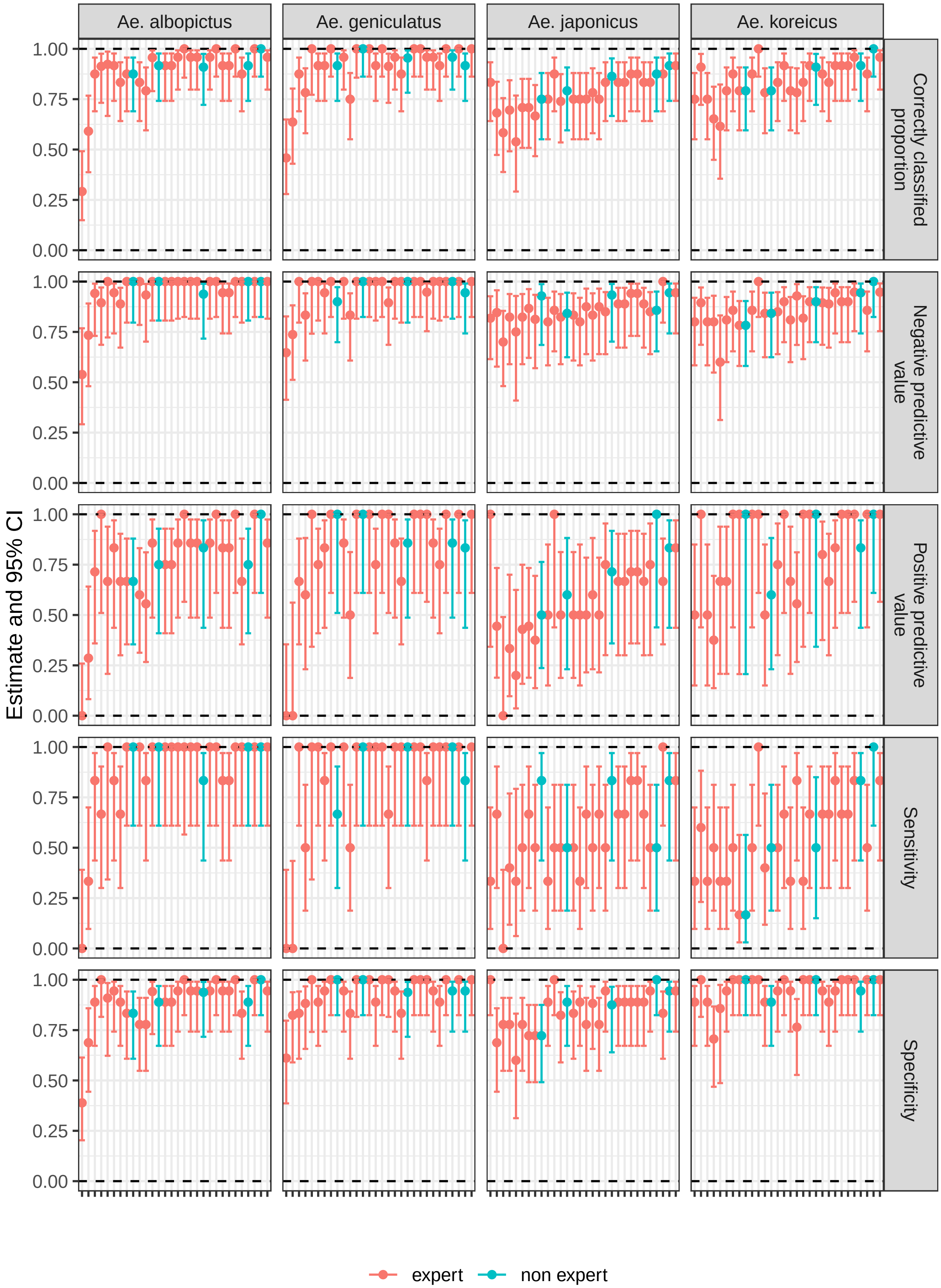

The numbering on the x-axis shows the individual IDs of the raters. To increase the clarity of the numbering,

the IDs were displayed on 3 different levels. The first IDs displayed are therefore 19, 6, 13, 8, 15, 5 and so on.

Note that the IDs are roughly ordered for the size of the estimate, this ordering makes only partly sense since the estimates are across different species and different measures of accuracy.

We saw in the previous figure two outliers for *albopictus* and *geniculatus*. We investigate these outliers.

```
d.disc.eggs %>%
  filter(id == 19) %>%
  arrange(`MALDI-TOF determination`) %>%

  select(id, group.expert, question, answer.rater, `MALDI-TOF determination`) %>%
  # print(n = Inf)
  kable(
    caption = paste0('Rating answers ("answer.rater") of rater 19. Correct species classifications are s
    label = "tab:OutlierInvestigation19",
    booktabs = TRUE,
    longtable = TRUE,
    linesep = c('')) %>%
  kable_styling(font_size = 7,
                latex_options = c("striped", "repeat_header", "hold_position"))
```

Table 44: Rating answers ("answer.rater") of rater 19. Correct species classifications are shown in variable "MALDI-TOF determination". Table is sorted for the variable "MALDI-TOF determination".

id	group.expert	question	answer.rater	MALDI-TOF determination
19	expert	1	geniculatus	albopictus
19	expert	5	geniculatus	albopictus
19	expert	7	geniculatus	albopictus
19	expert	11	geniculatus	albopictus
19	expert	17	geniculatus	albopictus
19	expert	23	geniculatus	albopictus
19	expert	6	albopictus	geniculatus
19	expert	10	albopictus	geniculatus
19	expert	12	albopictus	geniculatus
19	expert	18	albopictus	geniculatus
19	expert	20	albopictus	geniculatus
19	expert	22	albopictus	geniculatus
19	expert	3	koreicus	japonicus
19	expert	4	albopictus	japonicus
19	expert	8	japonicus	japonicus
19	expert	9	koreicus	japonicus
19	expert	15	albopictus	japonicus
19	expert	19	japonicus	japonicus
19	expert	2	albopictus	koreicus
19	expert	13	albopictus	koreicus
19	expert	14	geniculatus	koreicus
19	expert	16	koreicus	koreicus
19	expert	21	albopictus	koreicus
19	expert	24	koreicus	koreicus

ID 19 completely confused *geniculatus* with *albopictus*.

```
d.disc.eggs %>%
  filter(id == 6) %>%
  arrange(`MALDI-TOF determination`) %>%

  select(id, group.expert, question, answer.rater, `MALDI-TOF determination`) %>%
  # print(n = Inf)
```

```r
kable(
  caption = paste0('Rating answers ("answer.rater") of rater 6. Correct species classifications are sl
  label = "tab:OutlierInvestigation6",
  booktabs = TRUE,
  longtable = TRUE,
  linesep = c('')) %>%
kable_styling(font_size = 7,
              latex_options = c("striped", "repeat_header", "hold_position"))
```

Table 45: Rating answers ("answer.rater") of rater 6. Correct species classifications are shown in variable "MALDI-TOF determination". Table is sorted for the variable "MALDI-TOF determination".

id	group.expert	question	answer.rater	MALDI-TOF determination
6	expert	3	geniculatus	albopictus
6	expert	6	albopictus	albopictus
6	expert	12	albopictus	albopictus
6	expert	17	japonicus	albopictus
6	expert	22	japonicus	albopictus
6	expert	23	japonicus	albopictus
6	expert	4	japonicus	geniculatus
6	expert	5	albopictus	geniculatus
6	expert	14	albopictus	geniculatus
6	expert	16	albopictus	geniculatus
6	expert	18	NA	geniculatus
6	expert	24	albopictus	geniculatus
6	expert	1	geniculatus	japonicus
6	expert	2	japonicus	japonicus
6	expert	8	geniculatus	japonicus
6	expert	9	japonicus	japonicus
6	expert	13	japonicus	japonicus
6	expert	21	japonicus	japonicus
6	expert	7	koreicus	koreicus
6	expert	10	japonicus	koreicus
6	expert	11	koreicus	koreicus
6	expert	15	NA	koreicus
6	expert	19	koreicus	koreicus
6	expert	20	albopictus	koreicus

ID 6 got all *geniculatus* wrong.

This sounds positive overall: many raters got for *albopictus* and *geniculatus* all or most ratings correctly, despite some pictures having not ‘high’ quality. Moreover, persons like rater 6 and 19 would probably be able to improve largely with some feedback.

9 Graphical overview

We display an overview over all bounded measures of diagnostic accuracy. We start with the same overview as done in the analysis for the first workshop and then continue to summarize the additional results.

First, we collect all information into one dataframe.

```
d.overview.measures <- bind_rows(df.groups_list_diag.measures, .id = "id") %>%
  unnest(diag.measures)
# str(d.overview.measures)

d.overview.measures <- d.overview.measures %>%
  mutate(id = factor(x = id,
                     levels = c("Overall",
                                "Quality high", "Quality medium", "Quality low",
                                "Rater level expert", "Rater level non expert"),
                     labels = c("Overall",
                                "Quality high", "Quality medium", "Quality low",
                                "Rater level expert", "Rater level non expert")))
## assign factor levels
```

9.1 Compare groups (overall, quality, rater level)

In this section, we focus on comparing the groups (overall, quality, rater level) separately for each species.

```
d.overview.measures %>%
  mutate(Class = paste0("Ae. ", Class)) %>%
  # filter(Measure %in% c("Sensitivity", "Specificity")) %>%

  filter(bounded) %>%
  filter( ! (Measure %in% c("Apparent prevalence", "True prevalence"))) %>%

  ggplot(aes(x = id, y = est, color = id)) +
  facet_grid(Measure ~ Class, scales = "free", labeller = label_wrap_gen()) +
  geom_point() +
  geom_hline(yintercept = c(0, 1), linetype = "dashed") +
  geom_errorbar(aes(ymin = lower, ymax = upper)) +
  theme(axis.text.x = element_text(angle = 45, vjust = 1, hjust = 1)) +
  # theme(strip.text.x = element_text(size = 6)) +
  theme(legend.position = "none") +
  labs(x = "", y = "Estimate and 95% CI") ## title = "Overview - comparing groups",
```

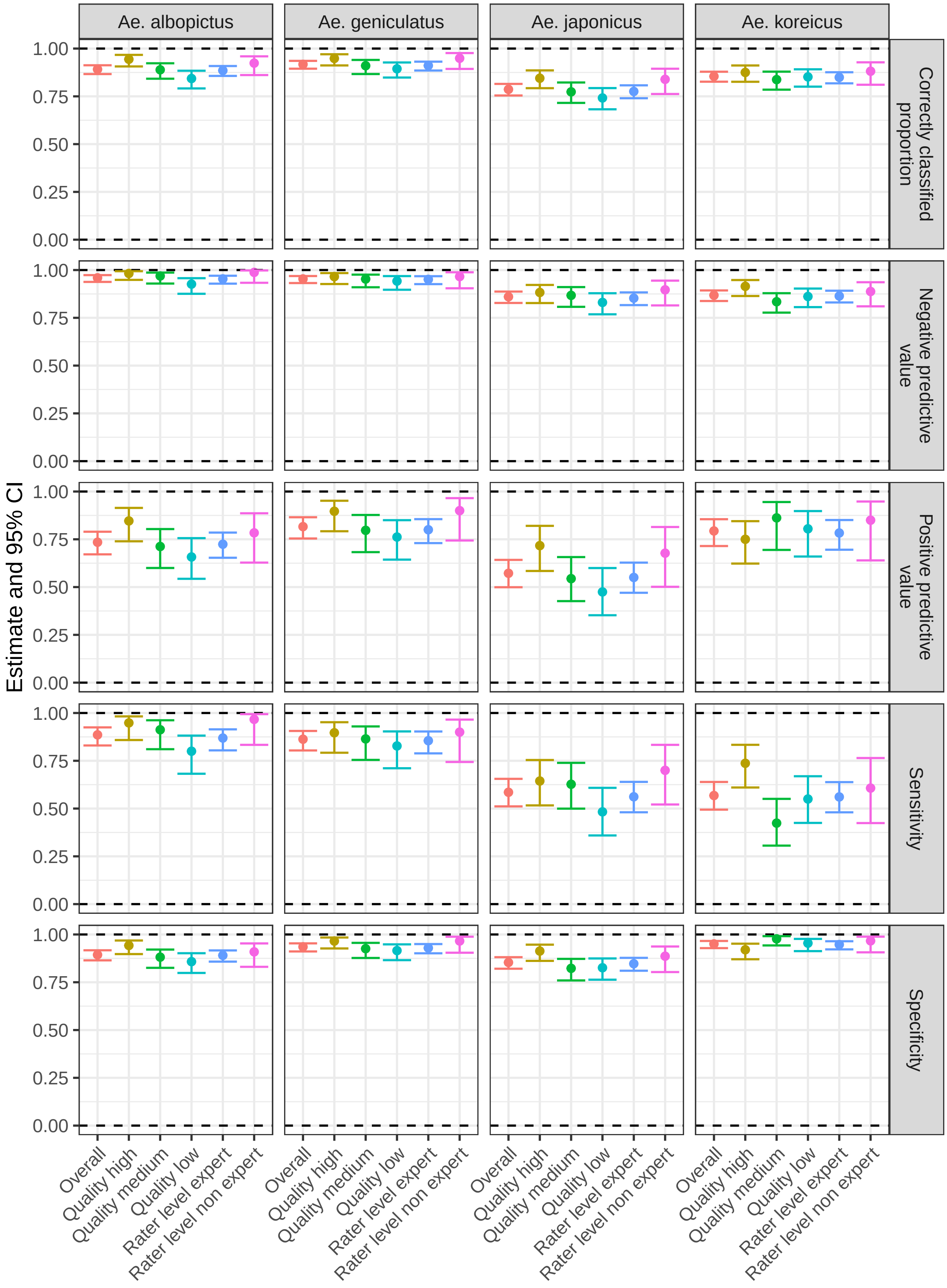

For the publication, we create a reduced plot, showing only sensitivity and specificity.

```
d.overview.measures %>%
  mutate(Class = paste0("Ae. ", Class)) %>%
  filter(Measure %in% c("Sensitivity", "Specificity")) %>%

  filter(bounded) %>%
  filter( ! (Measure %in% c("Apparent prevalence", "True prevalence"))) %>%

  ggplot(aes(x = id, y = est, color = id)) +
  facet_grid(Measure ~ Class, scales = "free", labeller = label_bquote(col = italic(.(Class)))) + # , l
  geom_point() +
  geom_hline(yintercept = c(0, 1), linetype = "dashed") +
  geom_errorbar(aes(ymin = lower, ymax = upper)) +
  theme(axis.text.x = element_text(angle = 45, vjust = 1, hjust = 1)) +
  # theme(strip.text.x = element_text(size = 6)) +
  theme(legend.position = "none") +
  labs(x = "", y = "Estimate and 95% CI") ## title = "Overview - comparing groups",
```

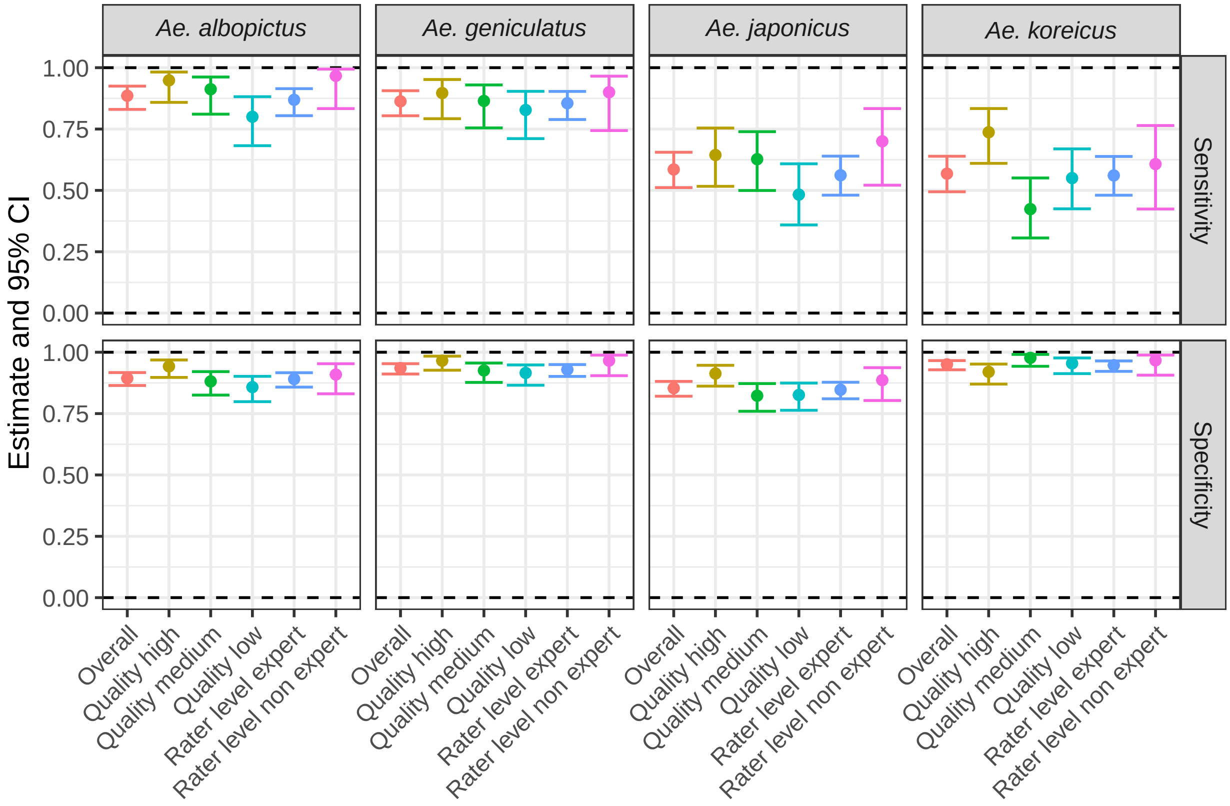

9.2 Compare species

In this section, we focus on comparing the four different species.

```
d.overview.measures %>%
  mutate(Class = paste0("Ae. ", Class)) %>%
  # filter(Measure %in% c("Sensitivity", "Specificity")) %>%

  filter(bounded) %>%
  filter( ! (Measure %in% c("Apparent prevalence", "True prevalence"))) %>%
```

```r
ggplot(aes(x = Class, y = est, color = Class)) +
## we fix the labelling linebreak dependent on the axis
# facet_grid(Measure ~ id, scales = "free", labeller = label_wrap_gen()) +
facet_grid(Measure ~ id, scales = "free",
           labeller = labeller(Measure = label_wrap_gen(),
                               id = label_wrap_gen(width = 14))) +
geom_point() +
geom_hline(yintercept = c(0, 1), linetype = "dashed") +
geom_errorbar(aes(ymin = lower, ymax = upper)) +
theme(axis.text.x = element_text(angle = 45, vjust = 1, hjust = 1)) +
# theme(strip.text.x = element_text(size = 6)) +
theme(legend.position = "none") +
labs(x = "", y = "Estimate and 95% CI") ## title = "Overview - comparing species",
```

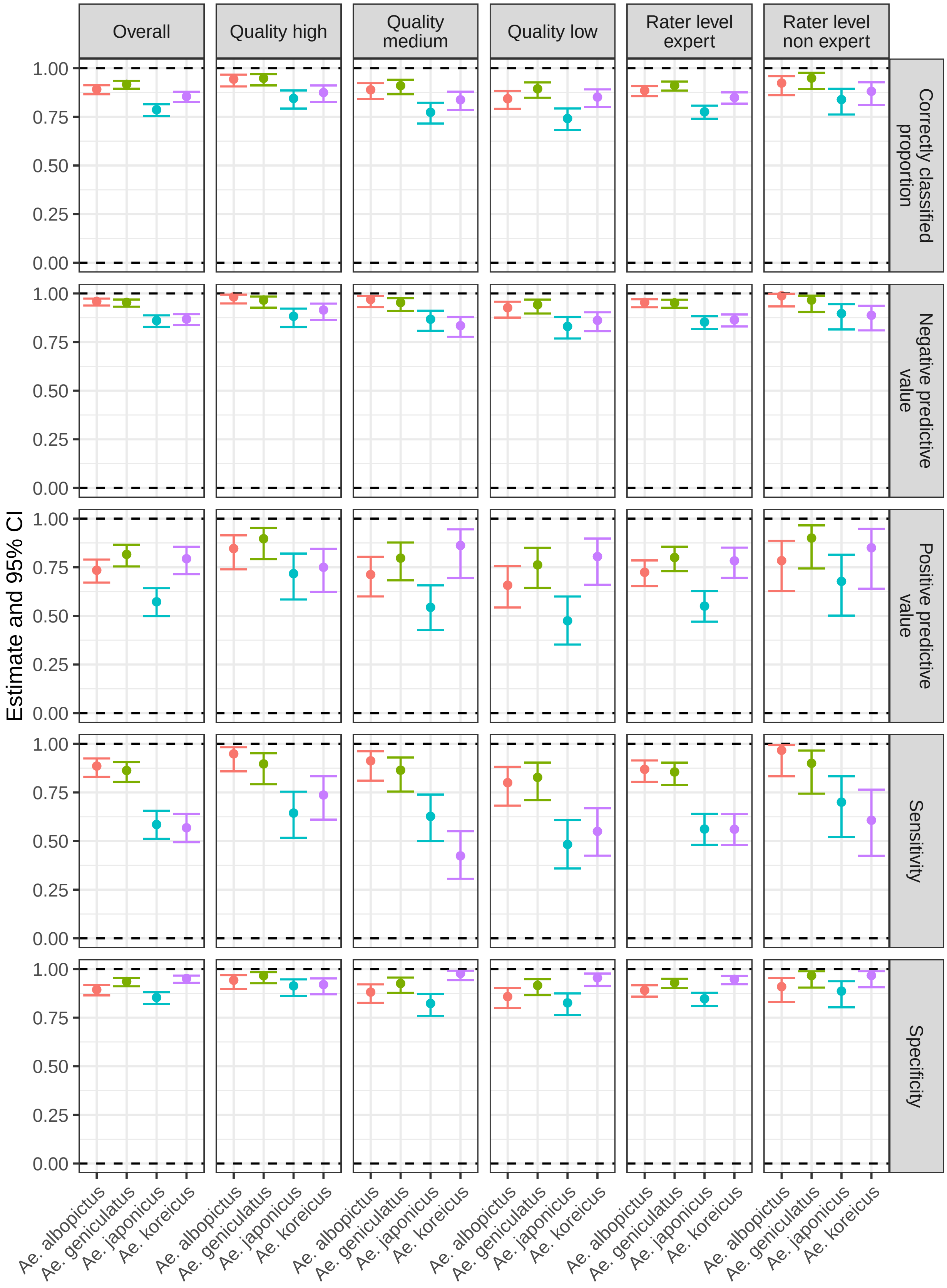

For the publication, we create a reduced plot, showing only sensitivity and specificity.

```r
d.overview.measures %>%
  mutate(Class = paste0("Ae. ", Class)) %>%
  filter(Measure %in% c("Sensitivity", "Specificity")) %>%

  filter(bounded) %>%
  filter( ! (Measure %in% c("Apparent prevalence", "True prevalence"))) %>%

  ggplot(aes(x = Class, y = est, color = Class)) +
  ## we fix the labelling linebreak dependent on the axis
  # facet_grid(Measure ~ id, scales = "free", labeller = label_wrap_gen()) +
  facet_grid(Measure ~ id, scales = "free",
             labeller = labeller(Measure = label_wrap_gen(),
                                  id = label_wrap_gen(width = 14))) +  geom_point() +
  geom_hline(yintercept = c(0, 1), linetype = "dashed") +
  geom_errorbar(aes(ymin = lower, ymax = upper)) +
  theme(axis.text.x = element_text(angle = 45, vjust = 1, hjust = 1)) +
  # theme(strip.text.x = element_text(size = 6)) +
  theme(legend.position = "none") +
  labs(x = "", y = "Estimate and 95% CI") ## title = "Overview - comparing species",
```

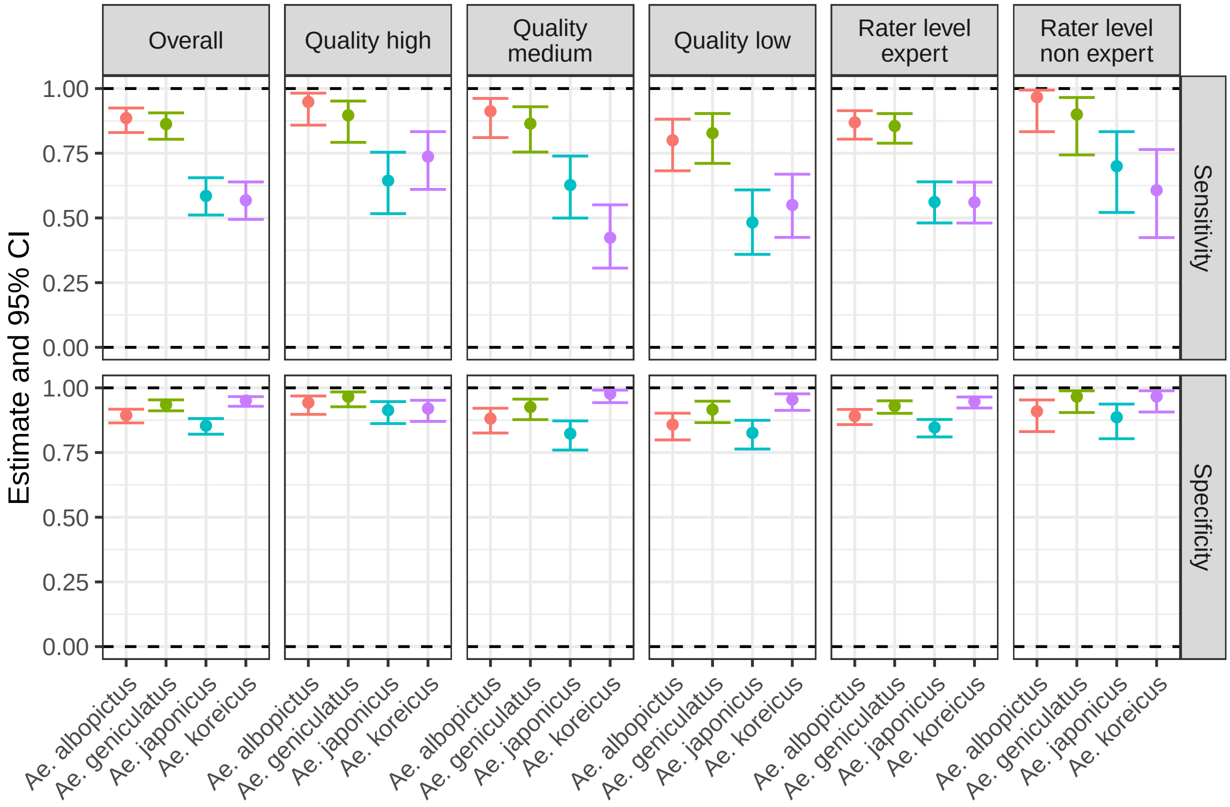

9.3 Compare rater levels

In this section, we focus on comparing the two rater levels (expert, non-expert).

```r
d.overview.measures %>%
  mutate(Class = paste0("Ae. ", Class)) %>%
  # filter(Measure %in% c("Sensitivity", "Specificity")) %>%
```

```
## now we have to filter "Overall"
filter(id %in% c("Rater level expert", "Rater level non expert")) %>%
filter(bounded) %>%
filter( ! (Measure %in% c("Apparent prevalence", "True prevalence"))) %>%
# print() %>%

ggplot(aes(x = id, y = est, color = id)) +
facet_grid(Measure ~ Class, scales = "free", labeller = label_wrap_gen()) +
geom_point() +
geom_hline(yintercept = c(0, 1), linetype = "dashed") +
geom_errorbar(aes(ymin = lower, ymax = upper)) +
theme(axis.text.x = element_text(angle = 45, vjust = 1, hjust = 1)) +
# theme(strip.text.x = element_text(size = 6)) +
theme(legend.position = "none") +
labs(x = "", y = "Estimate and 95% CI") ## title = "Overview - comparing species",
```

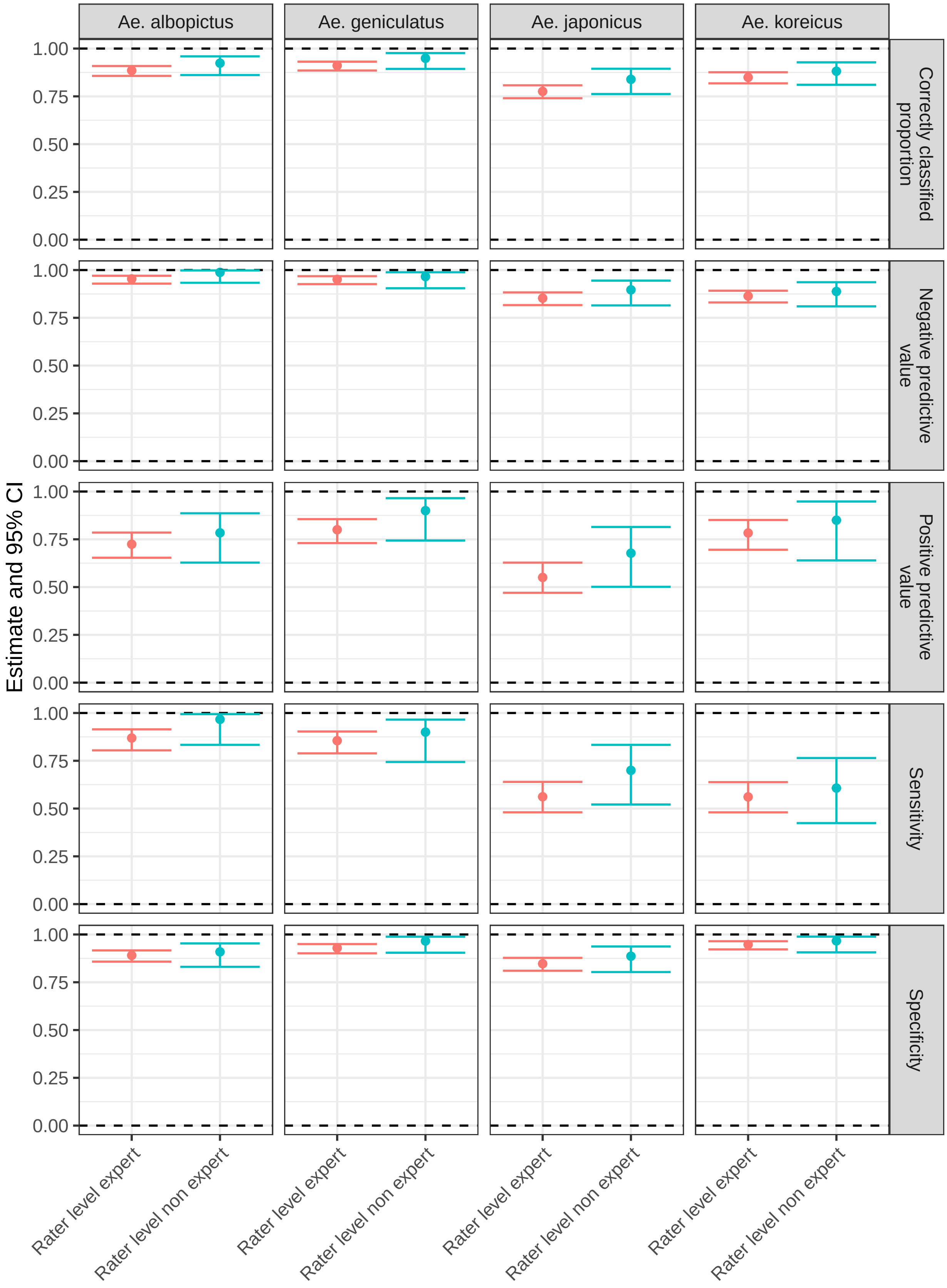
Ae. albopictus
Ae. geniculatus
Ae. japonicus
Ae. koreicus
Correctly classified proportion
Negative predictive value
Positive predictive value
Sensitivity
Specificity
Estimate and 95% CI
1.00
0.75
0.50
0.25
0.00
Rater level expert
Rater level non expert

9.4 Compare quality levels

In this section, we focus on comparing the three quality levels (high, medium, low).

```
d.overview.measures %>%
  mutate(Class = paste0("Ae. ", Class)) %>%
  # filter(Measure %in% c("Sensitivity", "Specificity")) %>%

  ## now we have to filter "Overall"
  filter(id %in% c("Quality high", "Quality medium", "Quality low")) %>%
  filter(bounded) %>%
  filter( ! (Measure %in% c("Apparent prevalence", "True prevalence"))) %>%
  # print() %>%

  ggplot(aes(x = id, y = est, color = id)) +
  facet_grid(Measure ~ Class, scales = "free", labeller = label_wrap_gen()) +
  geom_point() +
  geom_hline(yintercept = c(0, 1), linetype = "dashed") +
  geom_errorbar(aes(ymin = lower, ymax = upper)) +
  theme(axis.text.x = element_text(angle = 45, vjust = 1, hjust = 1)) +
  # theme(strip.text.x = element_text(size = 6)) +
  theme(legend.position = "none") +
  labs(x = "", y = "Estimate and 95% CI") ## title = "Overview - comparing species",
```

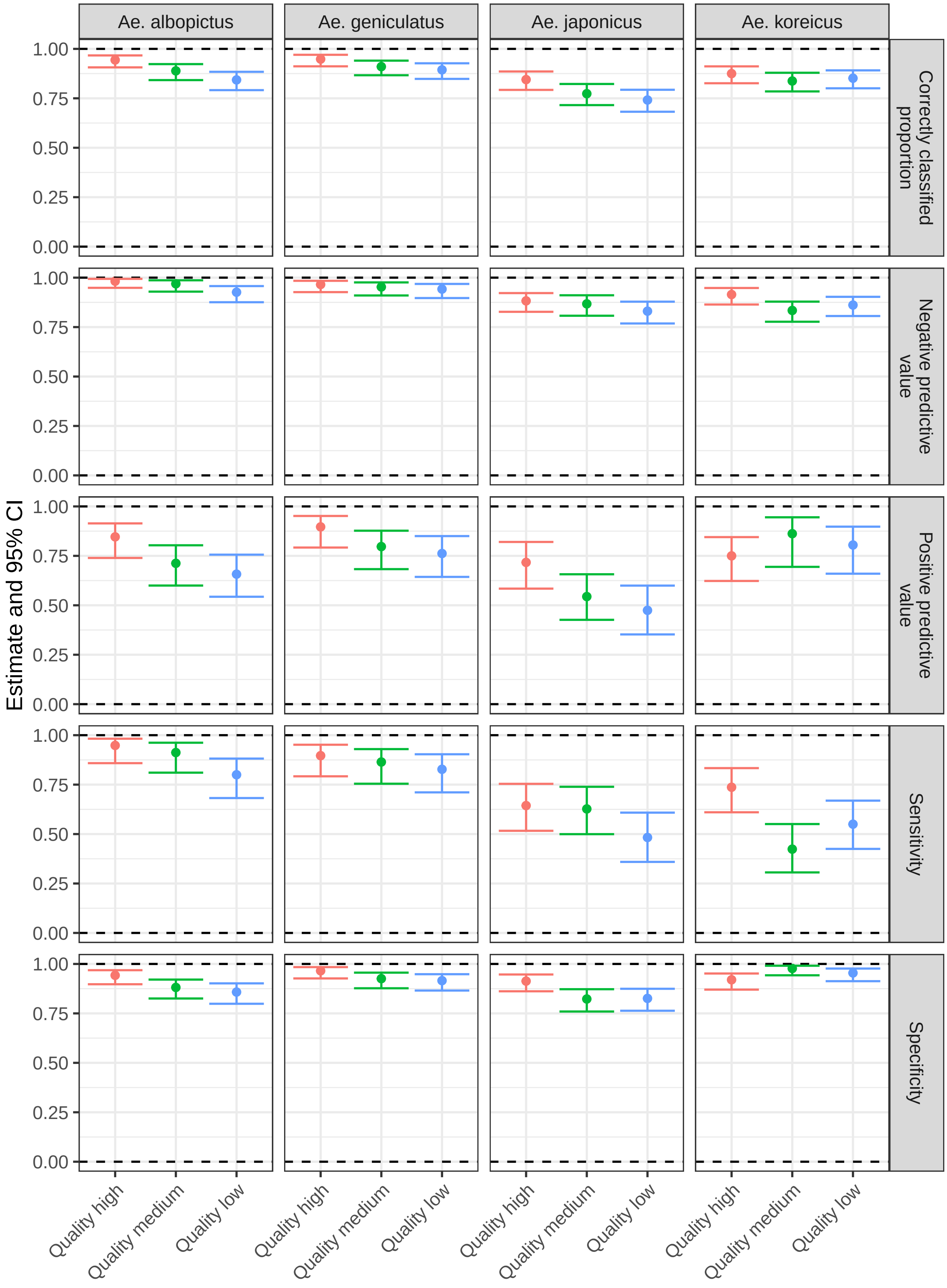
Ae. albopictus
Ae. geniculatus
Ae. japonicus
Ae. koreicus
Correctly classified proportion
Negative predictive value
Positive predictive value
Sensitivity
Specificity
Estimate and 95% CI
1.00
0.75
0.50
0.25
0.00
Quality high
Quality medium
Quality low

9.5 Compare per person

In this section, we focus on comparing the individual persons (raters).

We repeat the previous figure.

```
d.disc.eggs_person.un %>%
  mutate(Class = paste0("Ae. ", Class)) %>%
  filter(bounded) %>%
  filter( ! (Measure %in% c("Apparent prevalence", "True prevalence"))) %>%

  ggplot(aes(x = reorder(id, est), y = est)) +
  aes(color = group.expert) +

  facet_grid(Measure ~ Class, scales = "free", labeller = label_wrap_gen()) +
  geom_point() +
  geom_hline(yintercept = c(0, 1), linetype = "dashed") +
  geom_errorbar(aes(ymin = lower, ymax = upper)) +
  theme(legend.position = "bottom") +
  scale_x_discrete(guide = guide_axis(n.dodge = 3)) +
  theme(axis.text.x = element_blank()) + ## to remove the IDs (for publication)
  labs(x = "",
       y = "Estimate and 95% CI",
       color = "")
```

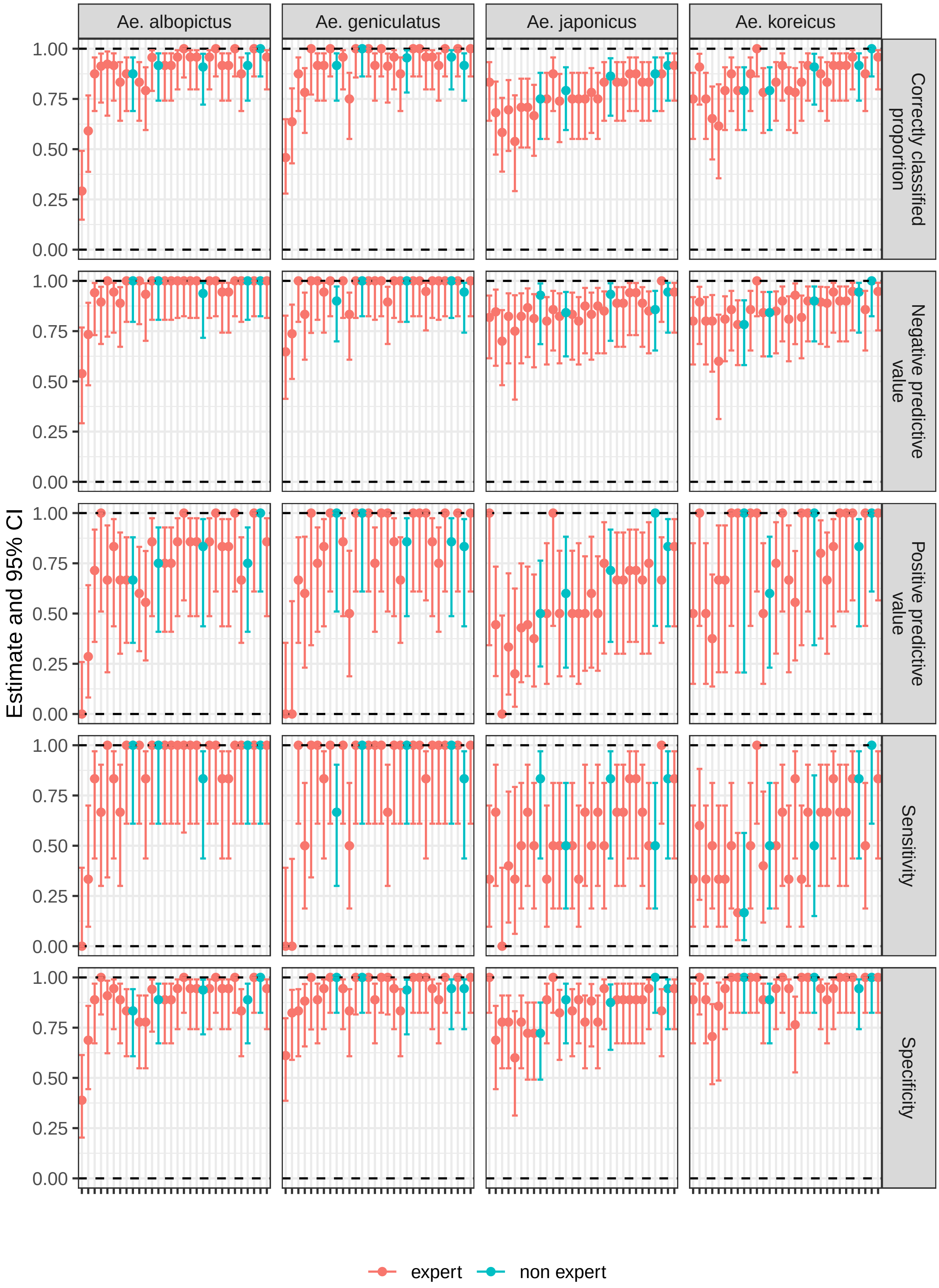
Ae. albopictus
Ae. geniculatus
Ae. japonicus
Ae. koreicus
Correctly classified proportion
Negative predictive value
Positive predictive value
Sensitivity
Specificity
Estimate and 95% CI
1.00
0.75
0.50
0.25
0.00
expert
non expert

10 Time spent on rating

We assess the time spent for classifying all 24 pictures (unit is minutes).

```
## We calculate some summary statistics over it
d.disc.eggs.l %>%
  distinct(id, .keep_all = TRUE) %>%
  pull(time_minutes) %>%
  summary()
```

```
   Min. 1st Qu.  Median    Mean 3rd Qu.    Max.
    3.2    10.0    11.6    11.6    13.0    30.0
```

Raters spent on average 11.6 minutes for all 24 images.

We visualize the time spent

```
d.disc.eggs.l %>%
  distinct(id, .keep_all = TRUE) %>%
  ggplot(aes(x = time_minutes)) +
  geom_rug(alpha = 0.3) +
  scale_x_continuous(limits = c(0, NA)) +
  geom_density()
```

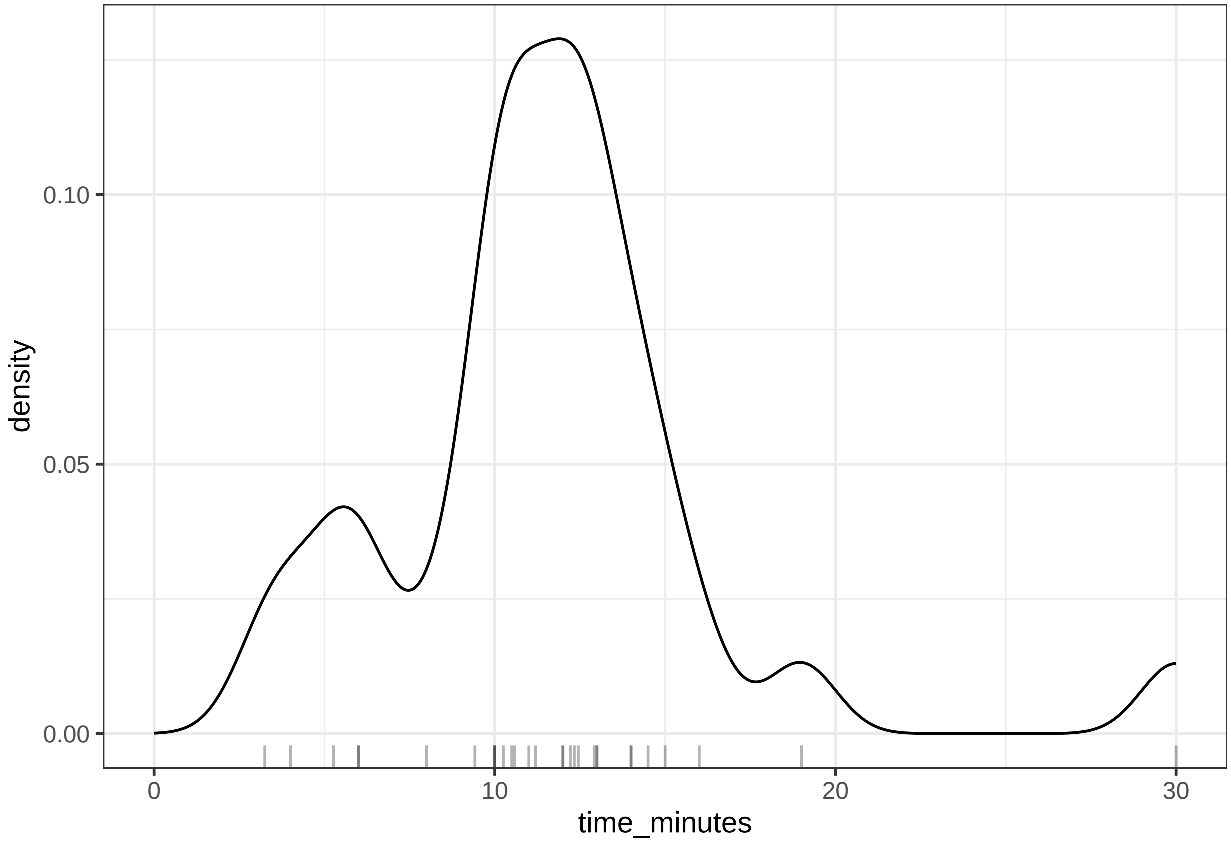


```
d.disc.eggs.l %>%
  distinct(id, .keep_all = TRUE) %>%
  ggplot(aes(x = "", y = time_minutes)) +
  geom_boxplot() +
  ggbeeswarm::geom_beeswarm(groupOnX = TRUE)
```

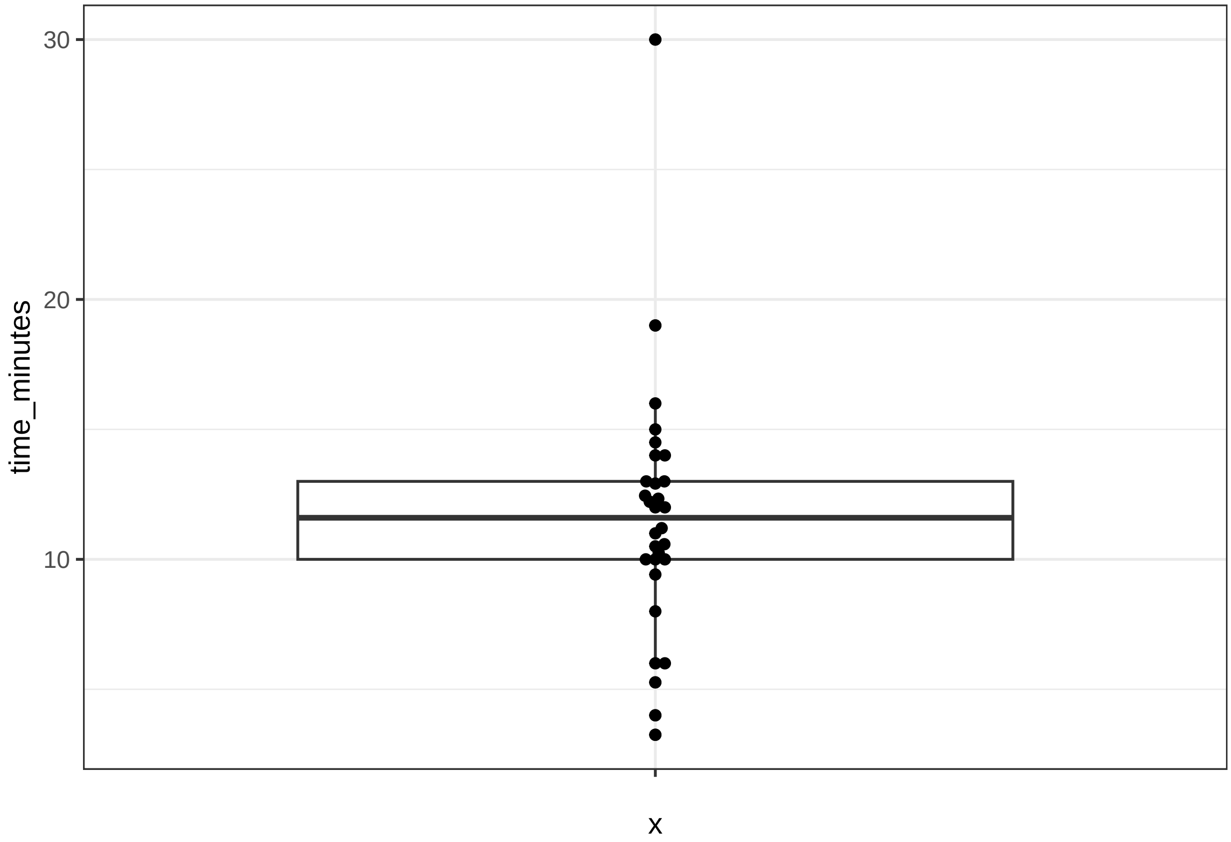

The median time spent was larger in the first project, but not considerably.

–>

11 Session information

```r
sessionInfo()
```

```
R version 4.2.2 (2022-10-31 ucrt)
Platform: x86_64-w64-mingw32/x64 (64-bit)
Running under: Windows 10 x64 (build 19045)

Matrix products: default

locale:
[1] LC_COLLATE=German_Switzerland.utf8  LC_CTYPE=German_Switzerland.utf8
[3] LC_MONETARY=German_Switzerland.utf8 LC_NUMERIC=C
[5] LC_TIME=German_Switzerland.utf8

attached base packages:
[1] stats     graphics  grDevices utils     datasets  methods   base

other attached packages:
 [1] binom_1.1-1.1    openxlsx_4.2.5.1 tidyr_1.2.1      forcats_0.5.2
 [5] purrr_0.3.5      epiR_2.0.53      survival_3.4-0   ggplot2_3.4.0
 [9] kableExtra_1.3.4 dplyr_1.0.10     checkpoint_1.0.2 knitr_1.40

loaded via a namespace (and not attached):
 [1] Rcpp_1.0.9         svglite_2.1.0      lubridate_1.9.0    lattice_0.20-45
 [5] class_7.3-20       zoo_1.8-11         assertthat_0.2.1   digest_0.6.30
 [9] utf8_1.2.2         R6_2.5.1           evaluate_0.18      e1071_1.7-12
[13] httr_1.4.4         pillar_1.8.1       gdtools_0.2.4      rlang_1.0.6
[17] uuid_1.1-0         rstudioapi_0.14    data.table_1.14.4  Matrix_1.5-1
[21] flextable_0.8.3    rmarkdown_2.18     labeling_0.4.2     splines_4.2.2
[25] webshot_0.5.4      stringr_1.4.1      pander_0.6.5       munsell_0.5.0
[29] proxy_0.4-27       vipor_0.4.5        compiler_4.2.2     xfun_0.34
[33] pkgconfig_2.0.3    systemfonts_1.0.4  base64enc_0.1-3    ggbeeswarm_0.6.0
[37] htmltools_0.5.3    tidyselect_1.2.0   tibble_3.1.8       fansi_1.0.3
[41] viridisLite_0.4.1  withr_2.5.0        sf_1.0-9           grid_4.2.2
[45] gtable_0.3.1       lifecycle_1.0.3    DBI_1.1.3          magrittr_2.0.3
[49] BiasedUrn_2.0.8    units_0.8-0        scales_1.2.1       KernSmooth_2.23-20
[53] zip_2.2.2          cli_3.4.1          stringi_1.7.8      farver_2.1.1
[57] xml2_1.3.3         ellipsis_0.3.2     generics_0.1.3     vctrs_0.5.0
[61] tools_4.2.2        beeswarm_0.4.0     glue_1.6.2         officer_0.4.4
[65] fastmap_1.1.0      yaml_2.3.6         timechange_0.1.1   colorspace_2.0-3
[69] classInt_0.4-8     rvest_1.0.3
```
