# Supplementary material for: Optical recognition of the eggs of four Aedine mosquito species (Aedes albopictus, Aedes geniculatus, Aedes japonicus, and Aedes koreicus)
Source: PLoS One. 2023 Nov 1;18(11):e0293568. doi: 10.1371/journal.pone.0293568 (PMC10619821; doi:10.1371/journal.pone.0293568)
Supplement: S2 Text — https://doi.org/10.6084/m9.figshare.24368251. (PDF) [file pone.0293568.s016.pdf]

Graphical Analysis: Discriminate Eggs Project

Workshop 1

Author: Klaus Steigmiller | Zurich Data Scientists
Reviewer: Dr. Claude Renaux | Zurich Data Scientists

October 1, 2023

Contents

1 Freezing Package versions **3**

2 Load packages **3**

3 Settings **3**

4 Statistical Methods **3**

5 Getting data **5**

6 Preparation **5**

7 Analysis **7**

7.1 We look at *Overall* . . . 8
7.1.1 Dataset . . . 8
7.1.2 Missing values . . . 8
7.1.3 Confusion Matrix . . . 8
7.1.4 Measures of diagnostic accuracy . . . 12
7.2 We look at *Quality high* . . . 16
7.2.1 Dataset . . . 16
7.2.2 Missing values . . . 16
7.2.3 Confusion Matrix . . . 16
7.2.4 Measures of diagnostic accuracy . . . 20
7.3 We look at *Quality medium* . . . 24
7.3.1 Dataset . . . 24
7.3.2 Missing values . . . 24
7.3.3 Confusion Matrix . . . 24
7.3.4 Measures of diagnostic accuracy . . . 28
7.4 We look at *Quality low* . . . 32
7.4.1 Dataset . . . 32
7.4.2 Missing values . . . 32
7.4.3 Confusion Matrix . . . 32
7.4.4 Measures of diagnostic accuracy . . . 36
7.5 We look at *Rater level expert* . . . 40
7.5.1 Dataset . . . 40
7.5.2 Missing values . . . 40
7.5.3 Confusion Matrix . . . 40
7.5.4 Measures of diagnostic accuracy . . . 44

7.6 We look at *Rater level non expert* . . . 48
7.6.1 Dataset . . . 48
7.6.2 Missing values . . . 48
7.6.3 Confusion Matrix . . . 48
7.6.4 Measures of diagnostic accuracy . . . 52

8 Graphical overview **55**
8.1 Compare groups (overall, quality, rater level) . . . 55
8.2 Compare species . . . 57
8.3 Compare rater levels . . . 60
8.4 Compare quality levels . . . 63

9 Time spent on rating **65**

10 Session information **67**

1 Freezing Package versions

```r
## (messages are omitted in this chunk)
##
library(checkpoint)
checkpoint(snapshot_date = "2022-11-15")
```

2 Load packages

```r
## (messages are omitted from this chunk)
##
library(dplyr)
library(kableExtra)
library(ggplot2)
library(epiR)
library(kableExtra)
library(purrr)
library(forcats)
library(tidyr)
library(openxlsx)
```

3 Settings

Global settings:

```r
theme_set(theme_bw())

if (!dir.exists("Prepared_data_and_models")) {
  dir.create("Prepared_data_and_models")
}
```

4 Statistical Methods

Definitions:

- Sensitivity: is probability of testing positive if the condition is really present, i.e. the proportion of true positives.
- Specificity: is probability of testing negative if the patient has not the condition, i.e. the proportion of true negatives.
- PPV (positive predictive value): is the probability of having the condition if the test is positive. This measure is affected by the prevalence of the condition.
- NPV (negative predictive value): is the probability of not having the condition if the test is negative. This measure is affected by the prevalence of the condition.
- LR+ (positive likelihood ratio): is the ratio of sensitivity over (1-specificity). It summarizes how many times it is more/less likely to have a positive test finding when having the condition compared to not having the condition.
- LR- (negative likelihood ratio): is the ratio of (1-sensitivity) over specificity. It summarizes how many times it is more/less likely to have a negative test finding when having the condition compared to not having the condition
- Diagnostic accuracy: proportion of all tests that give a correct result.
- Diagnostic odds ratio is defined as how much more likely will the test make a correct diagnosis than an incorrect diagnosis in patients with the disease.

- The number needed to diagnose: the number of paitents that need to be tested to give one correct positive test.
- Youdens index is defined as 'sensitivy + specificity -1'. Youdens index ranges from -1 to $+1$ with values closer to 1 if both sensitivity and specificity are high (i.e., close to 1).

Method "wilson" was used for the confidence intervals for sensitivity, specificity, and positive and negative predictive value. Details for this method and the other choices of confidence intervals can be found on https://rdrr.io/cran/epiR/man/epi.tests.html.

Missing values are handled by *complete case analysis* (pairwise deletion of index/reference test observations).

5 Getting data

```
d.disc.eggs <- readRDS(file = paste0("Prepared_data_and_models/",
                                      "0_PreDataPreparationd.disc.eggs_multiple.long.rds"))
```

6 Preparation

We set the variable "answer.rater" as the index test. We set the variable "MALDI-TOF determination" as the reference test.

```
reference.test.chr <- "MALDI-TOF determination"
index.test.chr <- "answer.rater"
reference.vec <- d.disc.eggs$`MALDI-TOF determination`
index.vec <- d.disc.eggs$`answer.rater`
```

We first prepare the data

```
## auxillary object to re-format output
.match <- structure(list(
  name.raw = c("ap", "tp", "se", "sp", "diag.or", "nndx",
               "youden", "pv.pos", "pv.neg", "lr.pos", "lr.neg", "p.rout", "p.rin",
               "p.tpdn", "p.tndp", "p.dntp", "p.dptn", "diag.ac"),
  Measure = c("Apparent prevalence", "True prevalence", "Sensitivity", "Specificity",
              "Diagnostic odds ratio", "Number needed to diagnose", "Youdens index",
              "Positive predictive value", "Negative predictive value",
              "Positive likelihood ratio", "Negative likelihood ratio",
              "the proportion of subjects with the outcome ruled out",
              "the proportion of subjects with the outcome ruled in",
              "False T+ proportion for trueD-", "False T- proportion for trueD+",
              "False T+ proportion for T+", "False T- proportion for T-",
              "Correctly classified proportion" ),
  bounded = c(TRUE, TRUE, TRUE, TRUE, FALSE, FALSE, FALSE, TRUE, TRUE,
              FALSE, FALSE, TRUE, TRUE, TRUE, TRUE, TRUE, TRUE, TRUE)),
  class = c("tbl_df", "tbl", "data.frame"), row.names = c(NA, -18L))
```

```
.match %>%
  kable(caption = paste0("Auxiliary coding table to assign the appropriate abbreviations to measure typ
        label = "tab:TableMatch",
        booktabs = TRUE,
        longtable = TRUE,
        linesep = c("")) %>%
  kable_styling(font_size = 7,
                latex_options = c("striped", "repeat_header", "hold_position"))
```

Table 1: Auxiliary coding table to assign the appropriate abbreviations to measure types.

name.raw	Measure	bounded
ap	Apparent prevalence	TRUE
tp	True prevalence	TRUE
se	Sensitivity	TRUE
sp	Specificity	TRUE
diag.or	Diagnostic odds ratio	FALSE
nndx	Number needed to diagnose	FALSE
youden	Youdens index	FALSE
pv.pos	Positive predictive value	TRUE

Table 1: Auxiliary coding table to assign the appropriate abbreviations to measure types. *(continued)*

name.raw	Measure	bounded
pv.neg	Negative predictive value	TRUE
lr.pos	Positive likelihood ratio	FALSE
lr.neg	Negative likelihood ratio	FALSE
p.rout	the proportion of subjects with the outcome ruled out	TRUE
p.rin	the proportion of subjects with the outcome ruled in	TRUE
p.tpdn	False T+ proportion for trueD-	TRUE
p.tndp	False T- proportion for trueD+	TRUE
p.dntp	False T+ proportion for T+	TRUE
p.dptn	False T- proportion for T-	TRUE
diag.ac	Correctly classified proportion	TRUE

Specify the number of digits to which the results should be rounded.

```
digits.rounding <- 2
```

We define some auxillary functions to create the Confusion Matrix.

```
## function to round results
f.round <- function(x, digits.rounding){
  format(x = round(x = x, digits = digits.rounding),
         scientific = FALSE,
         nsmall = digits.rounding)
}
## function to create the confusion matrix
f.create.confusion.matrix <- function(x){
  ConfusionMatrix <- cbind(x, rowSums(x))
  ConfusionMatrix <- rbind(ConfusionMatrix, colSums(ConfusionMatrix))
  colnames(ConfusionMatrix) <- c(colnames(x), "Total")
  rownames(ConfusionMatrix) <- c(rownames(x), "Total")
  return(ConfusionMatrix)
}
## function to restructure confusion matrix for printing
f.create.confusion.matrix.print <- function(x, data = c("TP", "FN", "FP", "TN")){
  ConfusionMatrix.print <- x
  ConfusionMatrix.print[1:2, 1:2] <-
    paste0(ConfusionMatrix.print[1:2, 1:2],
           " (", array(data = data, dim = c(2, 2)), ")")
  ConfusionMatrix.print
}
## auxillary function for checking package version:
f.check.version <- function(x){
  max(grep(pattern = x, x = sort(c(x, c("2.0.50"))))) > 1
}
```

We fix/check the factor levels of index and reference test.

```
## collect all levels/categories
levels.combined <- unique(union(reference.vec, index.vec))
if_reference.vec_notfactor <- class(reference.vec) != "factor"
if_index.vec_notfactor <- class(index.vec) != "factor"
if(if_reference.vec_notfactor){
  levels.combined <- unique(union(levels.combined, levels(reference.vec)))
}
if(if_index.vec_notfactor){
```

```
  levels.combined <- unique(union(levels.combined, levels(index.vec)))
}
levels.combined <- na.omit(levels.combined)

if(if_reference.vec_notfactor){
  reference.vec <- factor(x = reference.vec, levels = levels.combined)
}
if(if_index.vec_notfactor){
  index.vec <- factor(x = index.vec, levels = levels.combined)
}
```

```
## relevel factor variables (needed for *True Positve*, etc.)
reference.vec <- stats::relevel(x = reference.vec, ref = "albopictus")
index.vec <- stats::relevel(x = index.vec, ref = "albopictus")

## re-assign to data.frame
d.disc.eggs[, "MALDI-TOF determination", drop = TRUE] <- reference.vec
d.disc.eggs[, "answer.rater", drop = TRUE] <- index.vec
```

7 Analysis

In this section, we calculate the confusion matrix, followed by measures of diagnostic accuracy.

First, we create lists to store the calculated objects.

```
df.groups_list_dataset <- list()
df.groups_list_diag.measures <- list()
```

7.1 We look at *Overall*

7.1.1 Dataset

```
## Dataset
`d.disc.eggs_Overall` <- d.disc.eggs %>% filter(`group` == "Overall")
## Number of observations
(nr.obs <- nrow(`d.disc.eggs_Overall`))
```

```
[1] 720
```

This dataset contains 720 observations.

7.1.2 Missing values

Next, we give an overview of the missing values.

```
`d.disc.eggs_Overall` %>%
  select(`answer.rater`, `MALDI-TOF determination`) %>%
  table(useNA = "always")
```

```
             MALDI-TOF determination
answer.rater  albopictus koreicus geniculatus japonicus <NA>
  albopictus         167        0           8        17    0
  koreicus             0       89           3        23    0
  geniculatus          4        9         165        25    0
  japonicus            6       78           2       110    0
  <NA>                 3        4           2         5    0
```

```
## Calculate the number of missing values in both index/reference test:
nr.NA <- `d.disc.eggs_Overall` %>%
  select(`answer.rater`, `MALDI-TOF determination`) %>%
  (\(x){sum(is.na(x))})
## Calculate the number of observations that will be removed:
(nr.deleted.obs <- `d.disc.eggs_Overall` %>%
    filter(is.na(`answer.rater`) | is.na(`MALDI-TOF determination`)) %>%
    nrow())
```

```
[1] 14
```

```
`d.disc.eggs_Overall` <- `d.disc.eggs_Overall` %>%
  filter(!is.na(`answer.rater`) & !is.na(`MALDI-TOF determination`))
```

There were 14 observations (rows) deleted due to missing values.

7.1.3 Confusion Matrix

We calculate the confusion matrices in the following steps.

```
tab <- xtabs(formula = ~ `answer.rater` + `MALDI-TOF determination`,
             data = `d.disc.eggs_Overall`)
tab %>%
  f.create.confusion.matrix() %>%
  as_tibble(rownames = "answer.rater") %>%
  kable(caption = paste0("Overall cross table."),
        label = "tab:CrossTable1",
```

```
        booktabs = TRUE,
        longtable = TRUE,
        linesep = c("")) %>%
  kable_styling(font_size = 7,
                latex_options = c("striped", "repeat_header", "hold_position")) %>%
  row_spec(row = 5, bold = T) %>%
  column_spec(column = 6, bold = T) %>%
  add_header_above(c("", "MALDI-TOF determination" = 4, ""))
```

Table 2: Overall cross table.

	MALDI-TOF determination				
answer.rater	albopictus	koreicus	geniculatus	japonicus	Total
albopictus	167	0	8	17	**192**
koreicus	0	89	3	23	**115**
geniculatus	4	9	165	25	**203**
japonicus	6	78	2	110	**196**
**Total**	**177**	**176**	**178**	**175**	**706**

Example how to read this table:

- There were 167 cases of *albopictus* that were correctly classified as *albopictus*, 0 cases of *albopictus* were wrongly classified as *koreicus*.
- There were 89 cases of *koreicus* that were correctly classified as *koreicus*, 0 cases of *koreicus* were wrongly classified as *albopictus*.

We present the same table in percentages. Note that all columns add to 100%.

```
tab <- xtabs(formula = ~ `answer.rater` + `MALDI-TOF determination`,
             data = `d.disc.eggs_Overall`)
prop.table(x = tab, margin = 2) %>%
  '*'(100) %>%
  rbind(., colSums(.)) %>%
  f.round(digits = digits.rounding) %>%
  kable(caption = paste0("Overall cross table in percentages. All columns add up to 100\\%."),
        label = "tab:CrossTablePercentage1",
        booktabs = TRUE,
        longtable = TRUE,
        linesep = c("")) %>%
  kable_styling(font_size = 7,
                latex_options = c("striped", "repeat_header", "hold_position")) %>%
  row_spec(row = 5, bold = T) %>%
  add_header_above(c("", "MALDI-TOF determination" = 4))
```

Table 3: Overall cross table in percentages. All columns add up to 100%.

	MALDI-TOF determination			
	albopictus	koreicus	geniculatus	japonicus
albopictus	94.35	0.00	4.49	9.71
koreicus	0.00	50.57	1.69	13.14
geniculatus	2.26	5.11	92.70	14.29
japonicus	3.39	44.32	1.12	62.86
	**100.00**	**100.00**	**100.00**	**100.00**

```
levels.chr <- levels(`d.disc.eggs_Overall`$`answer.rater`)
if(length(levels.chr) == 2){
```

```
  classes <- c("")
}else{
  classes <- levels.chr
}

## calculate tables
if(identical(classes, c(""))){
  l.tab <- list(xtabs(formula = ~ `answer.rater` + `MALDI-TOF determination`,
                      data = `d.disc.eggs_Overall`))
}else{
  l.tab <- lapply(X = classes, FUN = function(x){
    xtabs(formula = ~ `answer.rater` + `MALDI-TOF determination`,
          data = `d.disc.eggs_Overall` %>%
            mutate(across(.cols = c(`answer.rater`, `MALDI-TOF determination`), .fns = function(y){
              ## we merge categories into the category "neg."
              y %>%
                fct_collapse("neg." = setdiff(levels(`d.disc.eggs_Overall`$`answer.rater`), x)) %>%
                fct_relevel(x)
            })))
  })
}
## calculate confusion matrix
l.confusion.matrix <- lapply(X = l.tab,
                             FUN = f.create.confusion.matrix)
## calculate tables for printing
l.confusion.matrix.print <- lapply(X = l.confusion.matrix,
                                   FUN = f.create.confusion.matrix.print)
```

The next tables show the confusion matrices for the index test ("answer.rater") against the reference test ("MALDI-TOF determination").

```
for(class in seq_along(classes)){
  l.confusion.matrix.print[[class]] %>%
    as_tibble(rownames = "answer.rater") %>%
    kable(caption = paste0("Confusion matrix for class ‘",
                           levels(`d.disc.eggs_Overall`$`answer.rater`)[class], "’.
          Category ‘", levels(`d.disc.eggs_Overall`$`answer.rater`)[class],
          "’ is considered a positive result.
          Category ‘neg.’ is considered a negative result and
          contains the levels ",
          paste0(setdiff(levels(`d.disc.eggs_Overall`$`answer.rater`),
                         classes[class]), collapse = ", "), "."),
          label = paste0("tab:ConfusionMatrix1Class", class),
          booktabs = TRUE,
          longtable = TRUE,
          linesep = c("")) %>%
    kable_styling(font_size = 7,
                  latex_options = c("striped", "repeat_header", "hold_position")) %>%
    row_spec(row = nrow(l.confusion.matrix.print[[class]]), bold = T) %>%
    column_spec(column = ncol(l.confusion.matrix.print[[class]]) + 1, bold = T) %>%
    add_header_above(c("", "MALDI-TOF determination" =
                         ncol(l.confusion.matrix.print[[class]]) - 1, "")) %>%
    print()
```

```
}
```

Table 4: Confusion matrix for class 'albopictus'. Category 'albopictus' is considered a positive result. Category 'neg.' is considered a negative result and contains the levels koreicus, geniculatus, japonicus.

	MALDI-TOF determination		
answer.rater	albopictus	neg.	Total
albopictus	167 (TP)	25 (FP)	**192**
neg.	10 (FN)	504 (TN)	**514**
**Total**	**177**	**529**	**706**

Table 5: Confusion matrix for class 'koreicus'. Category 'koreicus' is considered a positive result. Category 'neg.' is considered a negative result and contains the levels albopictus, geniculatus, japonicus.

	MALDI-TOF determination		
answer.rater	koreicus	neg.	Total
koreicus	89 (TP)	26 (FP)	**115**
neg.	87 (FN)	504 (TN)	**591**
**Total**	**176**	**530**	**706**

Table 6: Confusion matrix for class 'geniculatus'. Category 'geniculatus' is considered a positive result. Category 'neg.' is considered a negative result and contains the levels albopictus, koreicus, japonicus.

	MALDI-TOF determination		
answer.rater	geniculatus	neg.	Total
geniculatus	165 (TP)	38 (FP)	**203**
neg.	13 (FN)	490 (TN)	**503**
**Total**	**178**	**528**	**706**

Table 7: Confusion matrix for class 'japonicus'. Category 'japonicus' is considered a positive result. Category 'neg.' is considered a negative result and contains the levels albopictus, koreicus, geniculatus.

	MALDI-TOF determination		
answer.rater	japonicus	neg.	Total
japonicus	110 (TP)	86 (FP)	**196**
neg.	65 (FN)	445 (TN)	**510**
**Total**	**175**	**531**	**706**

7.1.4 Measures of diagnostic accuracy

We now calculate measures of diagnostic accuracy.

```
res.diagnostic.measures <- tibble("Class" = classes) %>%
  mutate(tabs = l.tab) %>%

  ## epiR wants it in the format: c(TP, FP, FN, TN)
  mutate(confusion.vector = map(.x = tabs, .f = function(x){
    c("tp" = x[1, 1], "fp" = x[1, 2], "fn" = x[2, 1], "tn" = x[2, 2])
  })) %>%

  ## Calculate measures of diagnostic accuracy
  mutate(out.EpiR = map(.x = confusion.vector, .f = function(x){
    epi.tests(dat = x,
              method = "wilson",
              conf.level = 0.95)
  })) %>%

  ## reformat output, filter diag. measures of interest
  mutate(diag.measures = map(.x = out.EpiR, .f = function(x){
    x <- x %>%
      summary() %>%
      as_tibble(rownames = "name.raw") %>%
      mutate()
    if(f.check.version(x = packageVersion(pkg = "epiR"))){
      x <- x %>%
        mutate(name.raw = statistic) %>%
        select(-statistic)
    }
    x %>%
      left_join(y = .match, by = c("name.raw")) %>%
      filter(name.raw %in% c("ap", "tp", "se", "sp", "diag.or", "pv.pos", "pv.neg", "lr.pos", "lr.neg",
  })) %>%

  ## ensure boundaries of estimates and CI
  mutate(diag.measures = map(.x = diag.measures, .f = function(x){
    x %>%
      mutate(across(.cols = c(est, lower, upper), .fns = function(x){
        if_else(condition = bounded, true = pmin(1, pmax(0, x)), false = x)
      }))
  })) %>%

  ## put together output
  mutate(diag.measures.print = map(.x = diag.measures, .f = function(x){
    x %>%
      mutate(across(.cols = c("est", "lower", "upper"),
                    .fns = \(x){format(x = round(x = x, digits = digits.rounding + 1),
                                       scientific = FALSE,
                                       nsmall = digits.rounding + 1)})) %>%
      mutate("Estimate (95% CI)" = paste0(est, " (from ", lower, " to ", upper, ")")) %>%
      select(Measure, `Estimate (95% CI)`)
  }))
```

We restructure the results that are to be printed.

```
aux.df.rows <- res.diagnostic.measures %>%
  select(Class, diag.measures.print) %>%
  mutate(Class = paste0("Class ", Class)) %>%
  unnest(cols = diag.measures.print) %>%
  mutate(row.nr = seq_len(n())) %>%
  group_by(Class) %>%
  summarize(min = min(row.nr),
            max = max(row.nr)) %>%
  ungroup()
```

We prepare the printing of the table.

```
res.diagnostic.measures_print1 <- res.diagnostic.measures %>%
  select(Class, diag.measures.print) %>%
  unnest(cols = diag.measures.print) %>%
  select(-Class) %>%

  kable(caption = paste0("Estimate and 95\\% confidence intervals for prevalence
                         and different measures of accuracy."),
        label = "tab:ResultTable1",
        booktabs = TRUE,
        longtable = TRUE,
        linesep = c("")) %>%
  kable_styling(
    font_size = 7,
    repeat_header_method = "replace",
    repeat_header_text = paste0("Estimate and 95\\% confidence intervals for prevalence
                                and different measures of accuracy. (continued)"),
    latex_options = c("striped", "repeat_header", "hold_position")
  ) %>%

  group_rows(group_label = aux.df.rows$Class[1],
             start_row = aux.df.rows$min[1],
             end_row = aux.df.rows$max[1]) %>%
  group_rows(group_label = aux.df.rows$Class[2],
             start_row = aux.df.rows$min[2],
             end_row = aux.df.rows$max[2]) %>%
  group_rows(group_label = aux.df.rows$Class[3],
             start_row = aux.df.rows$min[3],
             end_row = aux.df.rows$max[3]) %>%
  group_rows(group_label = aux.df.rows$Class[4],
             start_row = aux.df.rows$min[4],
             end_row = aux.df.rows$max[4]) %>%
  collapse_rows(columns = 1, latex_hline = "major", valign = "middle")
```

The next table shows point estimate and 95% confidence intervals for prevalence and different measures of diagnostic accuracy.

```
res.diagnostic.measures_print1
```

Table 8: Estimate and 95% confidence intervals for prevalence and different measures of accuracy.

Measure	Estimate (95% CI)
**Class albopictus**	
Apparent prevalence	0.272 (from 0.240 to 0.306)

Table 8: Estimate and 95% confidence intervals for prevalence and different measures of accuracy. (continued)

Measure	Estimate (95% CI)
True prevalence	0.251 (from 0.220 to 0.284)
Sensitivity	0.944 (from 0.899 to 0.969)
Specificity	0.953 (from 0.931 to 0.968)
Correctly classified proportion	0.950 (from 0.932 to 0.964)
Diagnostic odds ratio	336.672 (from 158.407 to 715.550)
Positive predictive value	0.870 (from 0.815 to 0.910)
Negative predictive value	0.981 (from 0.965 to 0.989)
Positive likelihood ratio	19.965 (from 13.594 to 29.320)
Negative likelihood ratio	0.059 (from 0.032 to 0.108)
**Class koreicus**	
Apparent prevalence	0.163 (from 0.137 to 0.192)
True prevalence	0.249 (from 0.219 to 0.283)
Sensitivity	0.506 (from 0.432 to 0.579)
Specificity	0.951 (from 0.929 to 0.966)
Correctly classified proportion	0.840 (from 0.811 to 0.865)
Diagnostic odds ratio	19.830 (from 12.117 to 32.455)
Positive predictive value	0.774 (from 0.689 to 0.841)
Negative predictive value	0.853 (from 0.822 to 0.879)
Positive likelihood ratio	10.308 (from 6.894 to 15.413)
Negative likelihood ratio	0.520 (from 0.447 to 0.604)
**Class geniculatus**	
Apparent prevalence	0.288 (from 0.255 to 0.322)
True prevalence	0.252 (from 0.221 to 0.285)
Sensitivity	0.927 (from 0.879 to 0.957)
Specificity	0.928 (from 0.903 to 0.947)
Correctly classified proportion	0.928 (from 0.906 to 0.945)
Diagnostic odds ratio	163.664 (from 85.099 to 314.760)
Positive predictive value	0.813 (from 0.754 to 0.860)
Negative predictive value	0.974 (from 0.956 to 0.985)
Positive likelihood ratio	12.880 (from 9.456 to 17.544)
Negative likelihood ratio	0.079 (from 0.047 to 0.133)
**Class japonicus**	
Apparent prevalence	0.278 (from 0.246 to 0.312)
True prevalence	0.248 (from 0.217 to 0.281)
Sensitivity	0.629 (from 0.555 to 0.697)
Specificity	0.838 (from 0.804 to 0.867)
Correctly classified proportion	0.786 (from 0.754 to 0.815)
Diagnostic odds ratio	8.757 (from 5.966 to 12.854)
Positive predictive value	0.561 (from 0.491 to 0.629)
Negative predictive value	0.873 (from 0.841 to 0.899)
Positive likelihood ratio	3.881 (from 3.101 to 4.858)
Negative likelihood ratio	0.443 (from 0.364 to 0.539)

We save the created objects in a list.

```
df.groups_list_dataset[["Overall"]] <- `d.disc.eggs_Overall`
df.groups_list_diag.measures[["Overall"]] <- res.diagnostic.measures
```

We clean up the programming environment.

```
rm(tab, nr.obs, l.confusion.matrix,
   l.confusion.matrix.print, res.diagnostic.measures,
   aux.df.rows)
```

7.2 We look at *Quality high*

7.2.1 Dataset

```
## Dataset
`d.disc.eggs_Quality high` <- d.disc.eggs %>% filter(`group` == "Quality high")
## Number of observations
(nr.obs <- nrow(`d.disc.eggs_Quality high`))
```

```
[1] 240
```

This dataset contains 240 observations.

7.2.2 Missing values

Next, we give an overview of the missing values.

```
`d.disc.eggs_Quality high` %>%
  select(`answer.rater`, `MALDI-TOF determination`) %>%
  table(useNA = "always")
```

```
             MALDI-TOF determination
answer.rater  albopictus koreicus geniculatus japonicus <NA>
  albopictus          59        0           1         2    0
  koreicus             0       43           1        10    0
  geniculatus          0        0          58         9    0
  japonicus            1       17           0        38    0
  <NA>                 0        0           0         1    0
```

```
## Calculate the number of missing values in both index/reference test:
nr.NA <- `d.disc.eggs_Quality high` %>%
  select(`answer.rater`, `MALDI-TOF determination`) %>%
  (\(x){sum(is.na(x))})
## Calculate the number of observations that will be removed:
(nr.deleted.obs <- `d.disc.eggs_Quality high` %>%
    filter(is.na(`answer.rater`) | is.na(`MALDI-TOF determination`)) %>%
    nrow())
```

```
[1] 1
```

```
`d.disc.eggs_Quality high` <- `d.disc.eggs_Quality high` %>%
  filter(!is.na(`answer.rater`) & !is.na(`MALDI-TOF determination`))
```

There were 1 observations (rows) deleted due to missing values.

7.2.3 Confusion Matrix

We calculate the confusion matrices in the following steps.

```
tab <- xtabs(formula = ~ `answer.rater` + `MALDI-TOF determination`,
             data = `d.disc.eggs_Quality high`)
tab %>%
  f.create.confusion.matrix() %>%
  as_tibble(rownames = "answer.rater") %>%
  kable(caption = paste0("Overall cross table."),
        label = "tab:CrossTable2",
```

```
        booktabs = TRUE,
        longtable = TRUE,
        linesep = c("")) %>%
  kable_styling(font_size = 7,
                latex_options = c("striped", "repeat_header", "hold_position")) %>%
  row_spec(row = 5, bold = T) %>%
  column_spec(column = 6, bold = T) %>%
  add_header_above(c("", "MALDI-TOF determination" = 4, ""))
```

Table 9: Overall cross table.

	MALDI-TOF determination				
answer.rater	albopictus	koreicus	geniculatus	japonicus	Total
albopictus	59	0	1	2	**62**
koreicus	0	43	1	10	**54**
geniculatus	0	0	58	9	**67**
japonicus	1	17	0	38	**56**
**Total**	**60**	**60**	**60**	**59**	**239**

Example how to read this table:

- There were 59 cases of *albopictus* that were correctly classified as *albopictus*, 0 cases of *albopictus* were wrongly classified as *koreicus*.
- There were 43 cases of *koreicus* that were correctly classified as *koreicus*, 0 cases of *koreicus* were wrongly classified as *albopictus*.

We present the same table in percentages. Note that all columns add to 100%.

```
tab <- xtabs(formula = ~ `answer.rater` + `MALDI-TOF determination`,
             data = `d.disc.eggs_Quality high`)
prop.table(x = tab, margin = 2) %>%
  '*'(100) %>%
  rbind(., colSums(.)) %>%
  f.round(digits = digits.rounding) %>%
  kable(caption = paste0("Overall cross table in percentages. All columns add up to 100\\%."),
        label = "tab:CrossTablePercentage2",
        booktabs = TRUE,
        longtable = TRUE,
        linesep = c("")) %>%
  kable_styling(font_size = 7,
                latex_options = c("striped", "repeat_header", "hold_position")) %>%
  row_spec(row = 5, bold = T) %>%
  add_header_above(c("", "MALDI-TOF determination" = 4))
```

Table 10: Overall cross table in percentages. All columns add up to 100%.

	MALDI-TOF determination			
	albopictus	koreicus	geniculatus	japonicus
albopictus	98.33	0.00	1.67	3.39
koreicus	0.00	71.67	1.67	16.95
geniculatus	0.00	0.00	96.67	15.25
japonicus	1.67	28.33	0.00	64.41
	**100.00**	**100.00**	**100.00**	**100.00**

```
levels.chr <- levels(`d.disc.eggs_Quality high`$`answer.rater`)
if(length(levels.chr) == 2){
```

```r
  classes <- c("")
}else{
  classes <- levels.chr
}

## calculate tables
if(identical(classes, c(""))){
  l.tab <- list(xtabs(formula = ~ `answer.rater` + `MALDI-TOF determination`,
                      data = `d.disc.eggs_Quality high`))
}else{
  l.tab <- lapply(X = classes, FUN = function(x){
    xtabs(formula = ~ `answer.rater` + `MALDI-TOF determination`,
          data = `d.disc.eggs_Quality high` %>%
            mutate(across(.cols = c(`answer.rater`, `MALDI-TOF determination`), .fns = function(y){
              ## we merge categories into the category "neg."
              y %>%
                fct_collapse("neg." = setdiff(levels(`d.disc.eggs_Quality high`$`answer.rater`), x)) %>%
                fct_relevel(x)
            })))
  })
}
## calculate confusion matrix
l.confusion.matrix <- lapply(X = l.tab,
                             FUN = f.create.confusion.matrix)
## calculate tables for printing
l.confusion.matrix.print <- lapply(X = l.confusion.matrix,
                                   FUN = f.create.confusion.matrix.print)
```

The next tables show the confusion matrices for the index test ("answer.rater") against the reference test ("MALDI-TOF determination").

```r
for(class in seq_along(classes)){
  l.confusion.matrix.print[[class]] %>%
    as_tibble(rownames = "answer.rater") %>%
    kable(caption = paste0("Confusion matrix for class ‘",
                           levels(`d.disc.eggs_Quality high`$`answer.rater`)[class], "’.
          Category ‘", levels(`d.disc.eggs_Quality high`$`answer.rater`)[class],
          "’ is considered a positive result.
          Category ‘neg.’ is considered a negative result and
          contains the levels ",
          paste0(setdiff(levels(`d.disc.eggs_Quality high`$`answer.rater`),
                         classes[class]), collapse = ", "), "."),
          label = paste0("tab:ConfusionMatrix2Class", class),
          booktabs = TRUE,
          longtable = TRUE,
          linesep = c("")) %>%
    kable_styling(font_size = 7,
                  latex_options = c("striped", "repeat_header", "hold_position")) %>%
    row_spec(row = nrow(l.confusion.matrix.print[[class]]), bold = T) %>%
    column_spec(column = ncol(l.confusion.matrix.print[[class]]) + 1, bold = T) %>%
    add_header_above(c("", "MALDI-TOF determination" =
                         ncol(l.confusion.matrix.print[[class]]) - 1, "")) %>%
    print()
```

```
}
```

Table 11: Confusion matrix for class 'albopictus'. Category 'albopictus' is considered a positive result. Category 'neg.' is considered a negative result and contains the levels koreicus, geniculatus, japonicus.

	MALDI-TOF determination		
answer.rater	albopictus	neg.	Total
albopictus	59 (TP)	3 (FP)	**62**
neg.	1 (FN)	176 (TN)	**177**
**Total**	**60**	**179**	**239**

Table 12: Confusion matrix for class 'koreicus'. Category 'koreicus' is considered a positive result. Category 'neg.' is considered a negative result and contains the levels albopictus, geniculatus, japonicus.

	MALDI-TOF determination		
answer.rater	koreicus	neg.	Total
koreicus	43 (TP)	11 (FP)	**54**
neg.	17 (FN)	168 (TN)	**185**
**Total**	**60**	**179**	**239**

Table 13: Confusion matrix for class 'geniculatus'. Category 'geniculatus' is considered a positive result. Category 'neg.' is considered a negative result and contains the levels albopictus, koreicus, japonicus.

	MALDI-TOF determination		
answer.rater	geniculatus	neg.	Total
geniculatus	58 (TP)	9 (FP)	**67**
neg.	2 (FN)	170 (TN)	**172**
**Total**	**60**	**179**	**239**

Table 14: Confusion matrix for class 'japonicus'. Category 'japonicus' is considered a positive result. Category 'neg.' is considered a negative result and contains the levels albopictus, koreicus, geniculatus.

	MALDI-TOF determination		
answer.rater	japonicus	neg.	Total
japonicus	38 (TP)	18 (FP)	**56**
neg.	21 (FN)	162 (TN)	**183**
**Total**	**59**	**180**	**239**

7.2.4 Measures of diagnostic accuracy

We now calculate measures of diagnostic accuracy.

```
res.diagnostic.measures <- tibble("Class" = classes) %>%
  mutate(tabs = l.tab) %>%

  ## epiR wants it in the format: c(TP, FP, FN, TN)
  mutate(confusion.vector = map(.x = tabs, .f = function(x){
    c("tp" = x[1, 1], "fp" = x[1, 2], "fn" = x[2, 1], "tn" = x[2, 2])
  })) %>%

  ## Calculate measures of diagnostic accuracy
  mutate(out.EpiR = map(.x = confusion.vector, .f = function(x){
    epi.tests(dat = x,
              method = "wilson",
              conf.level = 0.95)
  })) %>%

  ## reformat output, filter diag. measures of interest
  mutate(diag.measures = map(.x = out.EpiR, .f = function(x){
    x <- x %>%
      summary() %>%
      as_tibble(rownames = "name.raw") %>%
      mutate()
    if(f.check.version(x = packageVersion(pkg = "epiR"))){
      x <- x %>%
        mutate(name.raw = statistic) %>%
        select(-statistic)
    }
    x %>%
      left_join(y = .match, by = c("name.raw")) %>%
      filter(name.raw %in% c("ap", "tp", "se", "sp", "diag.or", "pv.pos", "pv.neg", "lr.pos", "lr.neg",
  })) %>%

  ## ensure boundaries of estimates and CI
  mutate(diag.measures = map(.x = diag.measures, .f = function(x){
    x %>%
      mutate(across(.cols = c(est, lower, upper), .fns = function(x){
        if_else(condition = bounded, true = pmin(1, pmax(0, x)), false = x)
      }))
  })) %>%

  ## put together output
  mutate(diag.measures.print = map(.x = diag.measures, .f = function(x){
    x %>%
      mutate(across(.cols = c("est", "lower", "upper"),
                    .fns = \(x){format(x = round(x = x, digits = digits.rounding + 1),
                                       scientific = FALSE,
                                       nsmall = digits.rounding + 1)})) %>%
      mutate("Estimate (95% CI)" = paste0(est, " (from ", lower, " to ", upper, ")")) %>%
      select(Measure, `Estimate (95% CI)`)
  }))
```

We restructure the results that are to be printed.

```
aux.df.rows <- res.diagnostic.measures %>%
  select(Class, diag.measures.print) %>%
  mutate(Class = paste0("Class ", Class)) %>%
  unnest(cols = diag.measures.print) %>%
  mutate(row.nr = seq_len(n())) %>%
  group_by(Class) %>%
  summarize(min = min(row.nr),
            max = max(row.nr)) %>%
  ungroup()
```

We prepare the printing of the table.

```
res.diagnostic.measures_print2 <- res.diagnostic.measures %>%
  select(Class, diag.measures.print) %>%
  unnest(cols = diag.measures.print) %>%
  select(-Class) %>%

  kable(caption = paste0("Estimate and 95\\% confidence intervals for prevalence
                         and different measures of accuracy."),
        label = "tab:ResultTable2",
        booktabs = TRUE,
        longtable = TRUE,
        linesep = c("")) %>%
  kable_styling(
    font_size = 7,
    repeat_header_method = "replace",
    repeat_header_text = paste0("Estimate and 95\\% confidence intervals for prevalence
                                and different measures of accuracy. (continued)"),
    latex_options = c("striped", "repeat_header", "hold_position")
  ) %>%

  group_rows(group_label = aux.df.rows$Class[1],
             start_row = aux.df.rows$min[1],
             end_row = aux.df.rows$max[1]) %>%
  group_rows(group_label = aux.df.rows$Class[2],
             start_row = aux.df.rows$min[2],
             end_row = aux.df.rows$max[2]) %>%
  group_rows(group_label = aux.df.rows$Class[3],
             start_row = aux.df.rows$min[3],
             end_row = aux.df.rows$max[3]) %>%
  group_rows(group_label = aux.df.rows$Class[4],
             start_row = aux.df.rows$min[4],
             end_row = aux.df.rows$max[4]) %>%
  collapse_rows(columns = 1, latex_hline = "major", valign = "middle")
```

The next table shows point estimate and 95% confidence intervals for prevalence and different measures of diagnostic accuracy.

```
res.diagnostic.measures_print2
```

Table 15: Estimate and 95% confidence intervals for prevalence and different measures of accuracy.

Measure	Estimate (95% CI)
**Class albopictus**	
Apparent prevalence	0.259 (from 0.208 to 0.318)

Table 15: Estimate and 95% confidence intervals for prevalence and different measures of accuracy. (continued)

Measure	Estimate (95% CI)
True prevalence	0.251 (from 0.200 to 0.310)
Sensitivity	0.983 (from 0.911 to 0.997)
Specificity	0.983 (from 0.952 to 0.994)
Correctly classified proportion	0.983 (from 0.958 to 0.993)
Diagnostic odds ratio	3461.333 (from 353.227 to 33918.181)
Positive predictive value	0.952 (from 0.867 to 0.983)
Negative predictive value	0.994 (from 0.969 to 0.999)
Positive likelihood ratio	58.672 (from 19.095 to 180.280)
Negative likelihood ratio	0.017 (from 0.002 to 0.118)
**Class koreicus**	
Apparent prevalence	0.226 (from 0.178 to 0.283)
True prevalence	0.251 (from 0.200 to 0.310)
Sensitivity	0.717 (from 0.592 to 0.815)
Specificity	0.939 (from 0.893 to 0.965)
Correctly classified proportion	0.883 (from 0.836 to 0.918)
Diagnostic odds ratio	38.631 (from 16.860 to 88.513)
Positive predictive value	0.796 (from 0.671 to 0.882)
Negative predictive value	0.908 (from 0.858 to 0.942)
Positive likelihood ratio	11.662 (from 6.438 to 21.127)
Negative likelihood ratio	0.302 (from 0.202 to 0.452)
**Class geniculatus**	
Apparent prevalence	0.280 (from 0.227 to 0.340)
True prevalence	0.251 (from 0.200 to 0.310)
Sensitivity	0.967 (from 0.886 to 0.991)
Specificity	0.950 (from 0.907 to 0.973)
Correctly classified proportion	0.954 (from 0.919 to 0.974)
Diagnostic odds ratio	547.778 (from 115.005 to 2609.105)
Positive predictive value	0.866 (from 0.764 to 0.928)
Negative predictive value	0.988 (from 0.959 to 0.997)
Positive likelihood ratio	19.226 (from 10.154 to 36.404)
Negative likelihood ratio	0.035 (from 0.009 to 0.137)
**Class japonicus**	
Apparent prevalence	0.234 (from 0.185 to 0.292)
True prevalence	0.247 (from 0.196 to 0.305)
Sensitivity	0.644 (from 0.517 to 0.754)
Specificity	0.900 (from 0.847 to 0.936)
Correctly classified proportion	0.837 (from 0.785 to 0.878)
Diagnostic odds ratio	16.286 (from 7.912 to 33.522)
Positive predictive value	0.679 (from 0.548 to 0.786)
Negative predictive value	0.885 (from 0.831 to 0.924)
Positive likelihood ratio	6.441 (from 3.995 to 10.383)
Negative likelihood ratio	0.395 (from 0.280 to 0.559)

We save the created objects in a list.

```
df.groups_list_dataset[["Quality high"]] <- `d.disc.eggs_Quality high`
df.groups_list_diag.measures[["Quality high"]] <- res.diagnostic.measures
```

We clean up the programming environment.

```
rm(tab, nr.obs, l.confusion.matrix,
   l.confusion.matrix.print, res.diagnostic.measures,
   aux.df.rows)
```

7.3 We look at *Quality medium*

7.3.1 Dataset

```
## Dataset
`d.disc.eggs_Quality medium` <- d.disc.eggs %>% filter(`group` == "Quality medium")
## Number of observations
(nr.obs <- nrow(`d.disc.eggs_Quality medium`))
```

```
[1] 240
```

This dataset contains 240 observations.

7.3.2 Missing values

Next, we give an overview of the missing values.

```
`d.disc.eggs_Quality medium` %>%
  select(`answer.rater`, `MALDI-TOF determination`) %>%
  table(useNA = "always")
```

```
             MALDI-TOF determination
answer.rater  albopictus koreicus geniculatus japonicus <NA>
  albopictus          57        0           3         4    0
  koreicus             0       19           2         5    0
  geniculatus          1        2          55        10    0
  japonicus            1       37           0        40    0
  <NA>                 1        2           0         1    0
```

```
## Calculate the number of missing values in both index/reference test:
nr.NA <- `d.disc.eggs_Quality medium` %>%
  select(`answer.rater`, `MALDI-TOF determination`) %>%
  (\(x){sum(is.na(x))})
## Calculate the number of observations that will be removed:
(nr.deleted.obs <- `d.disc.eggs_Quality medium` %>%
    filter(is.na(`answer.rater`) | is.na(`MALDI-TOF determination`)) %>%
    nrow())
```

```
[1] 4
```

```
`d.disc.eggs_Quality medium` <- `d.disc.eggs_Quality medium` %>%
  filter(!is.na(`answer.rater`) & !is.na(`MALDI-TOF determination`))
```

There were 4 observations (rows) deleted due to missing values.

7.3.3 Confusion Matrix

We calculate the confusion matrices in the following steps.

```
tab <- xtabs(formula = ~ `answer.rater` + `MALDI-TOF determination`,
             data = `d.disc.eggs_Quality medium`)
tab %>%
  f.create.confusion.matrix() %>%
  as_tibble(rownames = "answer.rater") %>%
  kable(caption = paste0("Overall cross table."),
        label = "tab:CrossTable3",
```

```
        booktabs = TRUE,
        longtable = TRUE,
        linesep = c("")) %>%
  kable_styling(font_size = 7,
                latex_options = c("striped", "repeat_header", "hold_position")) %>%
  row_spec(row = 5, bold = T) %>%
  column_spec(column = 6, bold = T) %>%
  add_header_above(c("", "MALDI-TOF determination" = 4, ""))
```

Table 16: Overall cross table.

	MALDI-TOF determination				
answer.rater	albopictus	koreicus	geniculatus	japonicus	Total
albopictus	57	0	3	4	**64**
koreicus	0	19	2	5	**26**
geniculatus	1	2	55	10	**68**
japonicus	1	37	0	40	**78**
**Total**	**59**	**58**	**60**	**59**	**236**

Example how to read this table:

- There were 57 cases of *albopictus* that were correctly classified as *albopictus*, 0 cases of *albopictus* were wrongly classified as *koreicus*.
- There were 19 cases of *koreicus* that were correctly classified as *koreicus*, 0 cases of *koreicus* were wrongly classified as *albopictus*.

We present the same table in percentages. Note that all columns add to 100%.

```
tab <- xtabs(formula = ~ `answer.rater` + `MALDI-TOF determination`,
             data = `d.disc.eggs_Quality medium`)
prop.table(x = tab, margin = 2) %>%
  '*'(100) %>%
  rbind(., colSums(.)) %>%
  f.round(digits = digits.rounding) %>%
  kable(caption = paste0("Overall cross table in percentages. All columns add up to 100\\%."),
        label = "tab:CrossTablePercentage3",
        booktabs = TRUE,
        longtable = TRUE,
        linesep = c("")) %>%
  kable_styling(font_size = 7,
                latex_options = c("striped", "repeat_header", "hold_position")) %>%
  row_spec(row = 5, bold = T) %>%
  add_header_above(c("", "MALDI-TOF determination" = 4))
```

Table 17: Overall cross table in percentages. All columns add up to 100%.

	MALDI-TOF determination			
	albopictus	koreicus	geniculatus	japonicus
albopictus	96.61	0.00	5.00	6.78
koreicus	0.00	32.76	3.33	8.47
geniculatus	1.69	3.45	91.67	16.95
japonicus	1.69	63.79	0.00	67.80
	**100.00**	**100.00**	**100.00**	**100.00**

```
levels.chr <- levels(`d.disc.eggs_Quality medium`$`answer.rater`)
if(length(levels.chr) == 2){
```

```
  classes <- c("")
}else{
  classes <- levels.chr
}

## calculate tables
if(identical(classes, c(""))){
  l.tab <- list(xtabs(formula = ~ `answer.rater` + `MALDI-TOF determination`,
                      data = `d.disc.eggs_Quality medium`))
}else{
  l.tab <- lapply(X = classes, FUN = function(x){
    xtabs(formula = ~ `answer.rater` + `MALDI-TOF determination`,
          data = `d.disc.eggs_Quality medium` %>%
            mutate(across(.cols = c(`answer.rater`, `MALDI-TOF determination`), .fns = function(y){
              ## we merge categories into the category "neg."
              y %>%
                fct_collapse("neg." = setdiff(levels(`d.disc.eggs_Quality medium`$`answer.rater`), x)) %
                fct_relevel(x)
            })))
  })
}
## calculate confusion matrix
l.confusion.matrix <- lapply(X = l.tab,
                             FUN = f.create.confusion.matrix)
## calculate tables for printing
l.confusion.matrix.print <- lapply(X = l.confusion.matrix,
                                   FUN = f.create.confusion.matrix.print)
```

The next tables show the confusion matrices for the index test ("answer.rater") against the reference test ("MALDI-TOF determination").

```
for(class in seq_along(classes)){
  l.confusion.matrix.print[[class]] %>%
    as_tibble(rownames = "answer.rater") %>%
    kable(caption = paste0("Confusion matrix for class ‘",
                           levels(`d.disc.eggs_Quality medium`$`answer.rater`)[class], "’.
          Category ‘", levels(`d.disc.eggs_Quality medium`$`answer.rater`)[class],
          "’ is considered a positive result.
          Category ‘neg.’ is considered a negative result and
          contains the levels ",
          paste0(setdiff(levels(`d.disc.eggs_Quality medium`$`answer.rater`),
                         classes[class]), collapse = ", "), "."),
          label = paste0("tab:ConfusionMatrix3Class", class),
          booktabs = TRUE,
          longtable = TRUE,
          linesep = c("")) %>%
    kable_styling(font_size = 7,
                  latex_options = c("striped", "repeat_header", "hold_position")) %>%
    row_spec(row = nrow(l.confusion.matrix.print[[class]]), bold = T) %>%
    column_spec(column = ncol(l.confusion.matrix.print[[class]]) + 1, bold = T) %>%
    add_header_above(c("", "MALDI-TOF determination" =
                         ncol(l.confusion.matrix.print[[class]]) - 1, "")) %>%
    print()
```

```
}
```

Table 18: Confusion matrix for class 'albopictus'. Category 'albopictus' is considered a positive result. Category 'neg.' is considered a negative result and contains the levels koreicus, geniculatus, japonicus.

	MALDI-TOF determination		
answer.rater	albopictus	neg.	Total
albopictus	57 (TP)	7 (FP)	**64**
neg.	2 (FN)	170 (TN)	**172**
**Total**	**59**	**177**	**236**

Table 19: Confusion matrix for class 'koreicus'. Category 'koreicus' is considered a positive result. Category 'neg.' is considered a negative result and contains the levels albopictus, geniculatus, japonicus.

	MALDI-TOF determination		
answer.rater	koreicus	neg.	Total
koreicus	19 (TP)	7 (FP)	**26**
neg.	39 (FN)	171 (TN)	**210**
**Total**	**58**	**178**	**236**

Table 20: Confusion matrix for class 'geniculatus'. Category 'geniculatus' is considered a positive result. Category 'neg.' is considered a negative result and contains the levels albopictus, koreicus, japonicus.

	MALDI-TOF determination		
answer.rater	geniculatus	neg.	Total
geniculatus	55 (TP)	13 (FP)	**68**
neg.	5 (FN)	163 (TN)	**168**
**Total**	**60**	**176**	**236**

Table 21: Confusion matrix for class 'japonicus'. Category 'japonicus' is considered a positive result. Category 'neg.' is considered a negative result and contains the levels albopictus, koreicus, geniculatus.

	MALDI-TOF determination		
answer.rater	japonicus	neg.	Total
japonicus	40 (TP)	38 (FP)	**78**
neg.	19 (FN)	139 (TN)	**158**
**Total**	**59**	**177**	**236**

7.3.4 Measures of diagnostic accuracy

We now calculate measures of diagnostic accuracy.

```
res.diagnostic.measures <- tibble("Class" = classes) %>%
  mutate(tabs = l.tab) %>%

  ## epiR wants it in the format: c(TP, FP, FN, TN)
  mutate(confusion.vector = map(.x = tabs, .f = function(x){
    c("tp" = x[1, 1], "fp" = x[1, 2], "fn" = x[2, 1], "tn" = x[2, 2])
  })) %>%

  ## Calculate measures of diagnostic accuracy
  mutate(out.EpiR = map(.x = confusion.vector, .f = function(x){
    epi.tests(dat = x,
              method = "wilson",
              conf.level = 0.95)
  })) %>%

  ## reformat output, filter diag. measures of interest
  mutate(diag.measures = map(.x = out.EpiR, .f = function(x){
    x <- x %>%
      summary() %>%
      as_tibble(rownames = "name.raw") %>%
      mutate()
    if(f.check.version(x = packageVersion(pkg = "epiR"))){
      x <- x %>%
        mutate(name.raw = statistic) %>%
        select(-statistic)
    }
    x %>%
      left_join(y = .match, by = c("name.raw")) %>%
      filter(name.raw %in% c("ap", "tp", "se", "sp", "diag.or", "pv.pos", "pv.neg", "lr.pos", "lr.neg",
  })) %>%

  ## ensure boundaries of estimates and CI
  mutate(diag.measures = map(.x = diag.measures, .f = function(x){
    x %>%
      mutate(across(.cols = c(est, lower, upper), .fns = function(x){
        if_else(condition = bounded, true = pmin(1, pmax(0, x)), false = x)
      }))
  })) %>%

  ## put together output
  mutate(diag.measures.print = map(.x = diag.measures, .f = function(x){
    x %>%
      mutate(across(.cols = c("est", "lower", "upper"),
                    .fns = \(x){format(x = round(x = x, digits = digits.rounding + 1),
                                       scientific = FALSE,
                                       nsmall = digits.rounding + 1)})) %>%
      mutate("Estimate (95% CI)" = paste0(est, " (from ", lower, " to ", upper, ")")) %>%
      select(Measure, `Estimate (95% CI)`)
  }))
```

We restructure the results that are to be printed.

```
aux.df.rows <- res.diagnostic.measures %>%
  select(Class, diag.measures.print) %>%
  mutate(Class = paste0("Class ", Class)) %>%
  unnest(cols = diag.measures.print) %>%
  mutate(row.nr = seq_len(n())) %>%
  group_by(Class) %>%
  summarize(min = min(row.nr),
            max = max(row.nr)) %>%
  ungroup()
```

We prepare the printing of the table.

```
res.diagnostic.measures_print3 <- res.diagnostic.measures %>%
  select(Class, diag.measures.print) %>%
  unnest(cols = diag.measures.print) %>%
  select(-Class) %>%

  kable(caption = paste0("Estimate and 95\\% confidence intervals for prevalence
                         and different measures of accuracy."),
        label = "tab:ResultTable3",
        booktabs = TRUE,
        longtable = TRUE,
        linesep = c("")) %>%
  kable_styling(
    font_size = 7,
    repeat_header_method = "replace",
    repeat_header_text = paste0("Estimate and 95\\% confidence intervals for prevalence
                                and different measures of accuracy. (continued)"),
    latex_options = c("striped", "repeat_header", "hold_position")
  ) %>%

  group_rows(group_label = aux.df.rows$Class[1],
             start_row = aux.df.rows$min[1],
             end_row = aux.df.rows$max[1]) %>%
  group_rows(group_label = aux.df.rows$Class[2],
             start_row = aux.df.rows$min[2],
             end_row = aux.df.rows$max[2]) %>%
  group_rows(group_label = aux.df.rows$Class[3],
             start_row = aux.df.rows$min[3],
             end_row = aux.df.rows$max[3]) %>%
  group_rows(group_label = aux.df.rows$Class[4],
             start_row = aux.df.rows$min[4],
             end_row = aux.df.rows$max[4]) %>%
  collapse_rows(columns = 1, latex_hline = "major", valign = "middle")
```

The next table shows point estimate and 95% confidence intervals for prevalence and different measures of diagnostic accuracy.

```
res.diagnostic.measures_print3
```

Table 22: Estimate and 95% confidence intervals for prevalence and different measures of accuracy.

Measure	Estimate (95% CI)
**Class albopictus**	
Apparent prevalence	0.271 (from 0.218 to 0.331)

Table 22: Estimate and 95% confidence intervals for prevalence and different measures of accuracy. (continued)

Measure	Estimate (95% CI)
True prevalence	0.250 (from 0.199 to 0.309)
Sensitivity	0.966 (from 0.885 to 0.991)
Specificity	0.960 (from 0.921 to 0.981)
Correctly classified proportion	0.962 (from 0.929 to 0.980)
Diagnostic odds ratio	692.143 (from 139.763 to 3427.670)
Positive predictive value	0.891 (from 0.791 to 0.946)
Negative predictive value	0.988 (from 0.959 to 0.997)
Positive likelihood ratio	24.429 (from 11.801 to 50.568)
Negative likelihood ratio	0.035 (from 0.009 to 0.138)
**Class koreicus**	
Apparent prevalence	0.110 (from 0.076 to 0.157)
True prevalence	0.246 (from 0.195 to 0.304)
Sensitivity	0.328 (from 0.221 to 0.456)
Specificity	0.961 (from 0.921 to 0.981)
Correctly classified proportion	0.805 (from 0.750 to 0.851)
Diagnostic odds ratio	11.901 (from 4.678 to 30.277)
Positive predictive value	0.731 (from 0.539 to 0.863)
Negative predictive value	0.814 (from 0.756 to 0.861)
Positive likelihood ratio	8.330 (from 3.690 to 18.807)
Negative likelihood ratio	0.700 (from 0.583 to 0.840)
**Class geniculatus**	
Apparent prevalence	0.288 (from 0.234 to 0.349)
True prevalence	0.254 (from 0.203 to 0.313)
Sensitivity	0.917 (from 0.819 to 0.964)
Specificity	0.926 (from 0.878 to 0.956)
Correctly classified proportion	0.924 (from 0.883 to 0.951)
Diagnostic odds ratio	137.923 (from 47.038 to 404.410)
Positive predictive value	0.809 (from 0.700 to 0.885)
Negative predictive value	0.970 (from 0.932 to 0.987)
Positive likelihood ratio	12.410 (from 7.314 to 21.056)
Negative likelihood ratio	0.090 (from 0.039 to 0.208)
**Class japonicus**	
Apparent prevalence	0.331 (from 0.274 to 0.393)
True prevalence	0.250 (from 0.199 to 0.309)
Sensitivity	0.678 (from 0.551 to 0.783)
Specificity	0.785 (from 0.719 to 0.839)
Correctly classified proportion	0.758 (from 0.700 to 0.809)
Diagnostic odds ratio	7.701 (from 4.006 to 14.802)
Positive predictive value	0.513 (from 0.404 to 0.621)
Negative predictive value	0.880 (from 0.820 to 0.922)
Positive likelihood ratio	3.158 (from 2.265 to 4.402)
Negative likelihood ratio	0.410 (from 0.281 to 0.599)

We save the created objects in a list.

```
df.groups_list_dataset[["Quality medium"]] <- `d.disc.eggs_Quality medium`
df.groups_list_diag.measures[["Quality medium"]] <- res.diagnostic.measures
```

We clean up the programming environment.

```
rm(tab, nr.obs, l.confusion.matrix,
   l.confusion.matrix.print, res.diagnostic.measures,
   aux.df.rows)
```

7.4 We look at *Quality low*

7.4.1 Dataset

```
## Dataset
`d.disc.eggs_Quality low` <- d.disc.eggs %>% filter(`group` == "Quality low")
## Number of observations
(nr.obs <- nrow(`d.disc.eggs_Quality low`))
```

```
[1] 240
```

This dataset contains 240 observations.

7.4.2 Missing values

Next, we give an overview of the missing values.

```
`d.disc.eggs_Quality low` %>%
  select(`answer.rater`, `MALDI-TOF determination`) %>%
  table(useNA = "always")
```

```
             MALDI-TOF determination
answer.rater  albopictus koreicus geniculatus japonicus <NA>
  albopictus          51        0           4        11    0
  koreicus             0       27           0         8    0
  geniculatus          3        7          52         6    0
  japonicus            4       24           2        32    0
  <NA>                 2        2           2         3    0
```

```
## Calculate the number of missing values in both index/reference test:
nr.NA <- `d.disc.eggs_Quality low` %>%
  select(`answer.rater`, `MALDI-TOF determination`) %>%
  (\(x){sum(is.na(x))})
## Calculate the number of observations that will be removed:
(nr.deleted.obs <- `d.disc.eggs_Quality low` %>%
    filter(is.na(`answer.rater`) | is.na(`MALDI-TOF determination`)) %>%
    nrow())
```

```
[1] 9
```

```
`d.disc.eggs_Quality low` <- `d.disc.eggs_Quality low` %>%
  filter(!is.na(`answer.rater`) & !is.na(`MALDI-TOF determination`))
```

There were 9 observations (rows) deleted due to missing values.

7.4.3 Confusion Matrix

We calculate the confusion matrices in the following steps.

```
tab <- xtabs(formula = ~ `answer.rater` + `MALDI-TOF determination`,
             data = `d.disc.eggs_Quality low`)
tab %>%
  f.create.confusion.matrix() %>%
  as_tibble(rownames = "answer.rater") %>%
  kable(caption = paste0("Overall cross table."),
        label = "tab:CrossTable4",
```

```
        booktabs = TRUE,
        longtable = TRUE,
        linesep = c("")) %>%
  kable_styling(font_size = 7,
                latex_options = c("striped", "repeat_header", "hold_position")) %>%
  row_spec(row = 5, bold = T) %>%
  column_spec(column = 6, bold = T) %>%
  add_header_above(c("", "MALDI-TOF determination" = 4, ""))
```

Table 23: Overall cross table.

	MALDI-TOF determination				
answer.rater	albopictus	koreicus	geniculatus	japonicus	Total
albopictus	51	0	4	11	**66**
koreicus	0	27	0	8	**35**
geniculatus	3	7	52	6	**68**
japonicus	4	24	2	32	**62**
**Total**	**58**	**58**	**58**	**57**	**231**

Example how to read this table:

- There were 51 cases of *albopictus* that were correctly classified as *albopictus*, 0 cases of *albopictus* were wrongly classified as *koreicus*.
- There were 27 cases of *koreicus* that were correctly classified as *koreicus*, 0 cases of *koreicus* were wrongly classified as *albopictus*.

We present the same table in percentages. Note that all columns add to 100%.

```
tab <- xtabs(formula = ~ `answer.rater` + `MALDI-TOF determination`,
             data = `d.disc.eggs_Quality low`)
prop.table(x = tab, margin = 2) %>%
  '*'(100) %>%
  rbind(., colSums(.)) %>%
  f.round(digits = digits.rounding) %>%
  kable(caption = paste0("Overall cross table in percentages. All columns add up to 100\\%."),
        label = "tab:CrossTablePercentage4",
        booktabs = TRUE,
        longtable = TRUE,
        linesep = c("")) %>%
  kable_styling(font_size = 7,
                latex_options = c("striped", "repeat_header", "hold_position")) %>%
  row_spec(row = 5, bold = T) %>%
  add_header_above(c("", "MALDI-TOF determination" = 4))
```

Table 24: Overall cross table in percentages. All columns add up to 100%.

	MALDI-TOF determination			
	albopictus	koreicus	geniculatus	japonicus
albopictus	87.93	0.00	6.90	19.30
koreicus	0.00	46.55	0.00	14.04
geniculatus	5.17	12.07	89.66	10.53
japonicus	6.90	41.38	3.45	56.14
	**100.00**	**100.00**	**100.00**	**100.00**

```
levels.chr <- levels(`d.disc.eggs_Quality low`$`answer.rater`)
if(length(levels.chr) == 2){
```

```
  classes <- c("")
}else{
  classes <- levels.chr
}

## calculate tables
if(identical(classes, c(""))){
  l.tab <- list(xtabs(formula = ~ `answer.rater` + `MALDI-TOF determination`,
                      data = `d.disc.eggs_Quality low`))
}else{
  l.tab <- lapply(X = classes, FUN = function(x){
    xtabs(formula = ~ `answer.rater` + `MALDI-TOF determination`,
          data = `d.disc.eggs_Quality low` %>%
            mutate(across(.cols = c(`answer.rater`, `MALDI-TOF determination`), .fns = function(y){
              ## we merge categories into the category "neg."
              y %>%
                fct_collapse("neg." = setdiff(levels(`d.disc.eggs_Quality low`$`answer.rater`), x)) %>%
                fct_relevel(x)
            })))
  })
}
## calculate confusion matrix
l.confusion.matrix <- lapply(X = l.tab,
                             FUN = f.create.confusion.matrix)
## calculate tables for printing
l.confusion.matrix.print <- lapply(X = l.confusion.matrix,
                                   FUN = f.create.confusion.matrix.print)
```

The next tables show the confusion matrices for the index test ("answer.rater") against the reference test ("MALDI-TOF determination").

```
for(class in seq_along(classes)){
  l.confusion.matrix.print[[class]] %>%
    as_tibble(rownames = "answer.rater") %>%
    kable(caption = paste0("Confusion matrix for class ‘",
                           levels(`d.disc.eggs_Quality low`$`answer.rater`)[class], "’.
          Category ‘", levels(`d.disc.eggs_Quality low`$`answer.rater`)[class],
          "’ is considered a positive result.
          Category ‘neg.’ is considered a negative result and
          contains the levels ",
          paste0(setdiff(levels(`d.disc.eggs_Quality low`$`answer.rater`),
                         classes[class]), collapse = ", "), "."),
          label = paste0("tab:ConfusionMatrix4Class", class),
          booktabs = TRUE,
          longtable = TRUE,
          linesep = c("")) %>%
    kable_styling(font_size = 7,
                  latex_options = c("striped", "repeat_header", "hold_position")) %>%
    row_spec(row = nrow(l.confusion.matrix.print[[class]]), bold = T) %>%
    column_spec(column = ncol(l.confusion.matrix.print[[class]]) + 1, bold = T) %>%
    add_header_above(c("", "MALDI-TOF determination" =
                         ncol(l.confusion.matrix.print[[class]]) - 1, "")) %>%
    print()
```

```
}
```

Table 25: Confusion matrix for class ‘albopictus’. Category ‘albopictus’ is considered a positive result. Category ‘neg.’ is considered a negative result and contains the levels koreicus, geniculatus, japonicus.

	MALDI-TOF determination		
answer.rater	albopictus	neg.	Total
albopictus	51 (TP)	15 (FP)	**66**
neg.	7 (FN)	158 (TN)	**165**
**Total**	**58**	**173**	**231**

Table 26: Confusion matrix for class ‘koreicus’. Category ‘koreicus’ is considered a positive result. Category ‘neg.’ is considered a negative result and contains the levels albopictus, geniculatus, japonicus.

	MALDI-TOF determination		
answer.rater	koreicus	neg.	Total
koreicus	27 (TP)	8 (FP)	**35**
neg.	31 (FN)	165 (TN)	**196**
**Total**	**58**	**173**	**231**

Table 27: Confusion matrix for class ‘geniculatus’. Category ‘geniculatus’ is considered a positive result. Category ‘neg.’ is considered a negative result and contains the levels albopictus, koreicus, japonicus.

	MALDI-TOF determination		
answer.rater	geniculatus	neg.	Total
geniculatus	52 (TP)	16 (FP)	**68**
neg.	6 (FN)	157 (TN)	**163**
**Total**	**58**	**173**	**231**

Table 28: Confusion matrix for class ‘japonicus’. Category ‘japonicus’ is considered a positive result. Category ‘neg.’ is considered a negative result and contains the levels albopictus, koreicus, geniculatus.

	MALDI-TOF determination		
answer.rater	japonicus	neg.	Total
japonicus	32 (TP)	30 (FP)	**62**
neg.	25 (FN)	144 (TN)	**169**
**Total**	**57**	**174**	**231**

7.4.4 Measures of diagnostic accuracy

We now calculate measures of diagnostic accuracy.

```
res.diagnostic.measures <- tibble("Class" = classes) %>%
  mutate(tabs = l.tab) %>%

  ## epiR wants it in the format: c(TP, FP, FN, TN)
  mutate(confusion.vector = map(.x = tabs, .f = function(x){
    c("tp" = x[1, 1], "fp" = x[1, 2], "fn" = x[2, 1], "tn" = x[2, 2])
  })) %>%

  ## Calculate measures of diagnostic accuracy
  mutate(out.EpiR = map(.x = confusion.vector, .f = function(x){
    epi.tests(dat = x,
              method = "wilson",
              conf.level = 0.95)
  })) %>%

  ## reformat output, filter diag. measures of interest
  mutate(diag.measures = map(.x = out.EpiR, .f = function(x){
    x <- x %>%
      summary() %>%
      as_tibble(rownames = "name.raw") %>%
      mutate()
    if(f.check.version(x = packageVersion(pkg = "epiR"))){
      x <- x %>%
        mutate(name.raw = statistic) %>%
        select(-statistic)
    }
    x %>%
      left_join(y = .match, by = c("name.raw")) %>%
      filter(name.raw %in% c("ap", "tp", "se", "sp", "diag.or", "pv.pos", "pv.neg", "lr.pos", "lr.neg",
  })) %>%

  ## ensure boundaries of estimates and CI
  mutate(diag.measures = map(.x = diag.measures, .f = function(x){
    x %>%
      mutate(across(.cols = c(est, lower, upper), .fns = function(x){
        if_else(condition = bounded, true = pmin(1, pmax(0, x)), false = x)
      }))
  })) %>%

  ## put together output
  mutate(diag.measures.print = map(.x = diag.measures, .f = function(x){
    x %>%
      mutate(across(.cols = c("est", "lower", "upper"),
                    .fns = \(x){format(x = round(x = x, digits = digits.rounding + 1),
                                       scientific = FALSE,
                                       nsmall = digits.rounding + 1)})) %>%
      mutate("Estimate (95% CI)" = paste0(est, " (from ", lower, " to ", upper, ")")) %>%
      select(Measure, `Estimate (95% CI)`)
  }))
```

We restructure the results that are to be printed.

```
aux.df.rows <- res.diagnostic.measures %>%
  select(Class, diag.measures.print) %>%
  mutate(Class = paste0("Class ", Class)) %>%
  unnest(cols = diag.measures.print) %>%
  mutate(row.nr = seq_len(n())) %>%
  group_by(Class) %>%
  summarize(min = min(row.nr),
            max = max(row.nr)) %>%
  ungroup()
```

We prepare the printing of the table.

```
res.diagnostic.measures_print4 <- res.diagnostic.measures %>%
  select(Class, diag.measures.print) %>%
  unnest(cols = diag.measures.print) %>%
  select(-Class) %>%

  kable(caption = paste0("Estimate and 95\\% confidence intervals for prevalence
                         and different measures of accuracy."),
        label = "tab:ResultTable4",
        booktabs = TRUE,
        longtable = TRUE,
        linesep = c("")) %>%
  kable_styling(
    font_size = 7,
    repeat_header_method = "replace",
    repeat_header_text = paste0("Estimate and 95\\% confidence intervals for prevalence
                                and different measures of accuracy. (continued)"),
    latex_options = c("striped", "repeat_header", "hold_position")
  ) %>%

  group_rows(group_label = aux.df.rows$Class[1],
             start_row = aux.df.rows$min[1],
             end_row = aux.df.rows$max[1]) %>%
  group_rows(group_label = aux.df.rows$Class[2],
             start_row = aux.df.rows$min[2],
             end_row = aux.df.rows$max[2]) %>%
  group_rows(group_label = aux.df.rows$Class[3],
             start_row = aux.df.rows$min[3],
             end_row = aux.df.rows$max[3]) %>%
  group_rows(group_label = aux.df.rows$Class[4],
             start_row = aux.df.rows$min[4],
             end_row = aux.df.rows$max[4]) %>%
  collapse_rows(columns = 1, latex_hline = "major", valign = "middle")
```

The next table shows point estimate and 95% confidence intervals for prevalence and different measures of diagnostic accuracy.

```
res.diagnostic.measures_print4
```

Table 29: Estimate and 95% confidence intervals for prevalence and different measures of accuracy.

Measure	Estimate (95% CI)
**Class albopictus**	
Apparent prevalence	0.286 (from 0.231 to 0.347)

Table 29: Estimate and 95% confidence intervals for prevalence and different measures of accuracy. (continued)

Measure	Estimate (95% CI)
True prevalence	0.251 (from 0.200 to 0.311)
Sensitivity	0.879 (from 0.771 to 0.940)
Specificity	0.913 (from 0.862 to 0.947)
Correctly classified proportion	0.905 (from 0.860 to 0.936)
Diagnostic odds ratio	76.743 (from 29.648 to 198.645)
Positive predictive value	0.773 (from 0.658 to 0.857)
Negative predictive value	0.958 (from 0.915 to 0.979)
Positive likelihood ratio	10.141 (from 6.195 to 16.603)
Negative likelihood ratio	0.132 (from 0.066 to 0.265)
**Class koreicus**	
Apparent prevalence	0.152 (from 0.111 to 0.203)
True prevalence	0.251 (from 0.200 to 0.311)
Sensitivity	0.466 (from 0.343 to 0.592)
Specificity	0.954 (from 0.911 to 0.976)
Correctly classified proportion	0.831 (from 0.778 to 0.874)
Diagnostic odds ratio	17.964 (from 7.471 to 43.192)
Positive predictive value	0.771 (from 0.610 to 0.879)
Negative predictive value	0.842 (from 0.784 to 0.886)
Positive likelihood ratio	10.067 (from 4.848 to 20.905)
Negative likelihood ratio	0.560 (from 0.440 to 0.714)
**Class geniculatus**	
Apparent prevalence	0.294 (from 0.239 to 0.356)
True prevalence	0.251 (from 0.200 to 0.311)
Sensitivity	0.897 (from 0.792 to 0.952)
Specificity	0.908 (from 0.855 to 0.942)
Correctly classified proportion	0.905 (from 0.860 to 0.936)
Diagnostic odds ratio	85.042 (from 31.622 to 228.703)
Positive predictive value	0.765 (from 0.651 to 0.850)
Negative predictive value	0.963 (from 0.922 to 0.983)
Positive likelihood ratio	9.694 (from 6.029 to 15.586)
Negative likelihood ratio	0.114 (from 0.053 to 0.244)
**Class japonicus**	
Apparent prevalence	0.268 (from 0.215 to 0.329)
True prevalence	0.247 (from 0.196 to 0.306)
Sensitivity	0.561 (from 0.433 to 0.682)
Specificity	0.828 (from 0.765 to 0.876)
Correctly classified proportion	0.762 (from 0.703 to 0.812)
Diagnostic odds ratio	6.144 (from 3.193 to 11.823)
Positive predictive value	0.516 (from 0.394 to 0.636)
Negative predictive value	0.852 (from 0.791 to 0.898)
Positive likelihood ratio	3.256 (from 2.186 to 4.849)
Negative likelihood ratio	0.530 (from 0.392 to 0.716)

We save the created objects in a list.

```r
df.groups_list_dataset[["Quality low"]] <- `d.disc.eggs_Quality low`
df.groups_list_diag.measures[["Quality low"]] <- res.diagnostic.measures
```

We clean up the programming environment.

```r
rm(tab, nr.obs, l.confusion.matrix,
   l.confusion.matrix.print, res.diagnostic.measures,
   aux.df.rows)
```

7.5 We look at *Rater level expert*

7.5.1 Dataset

```
## Dataset
`d.disc.eggs_Rater level expert` <- d.disc.eggs %>% filter(`group` == "Rater level expert")
## Number of observations
(nr.obs <- nrow(`d.disc.eggs_Rater level expert`))
```

```
[1] 240
```

This dataset contains 240 observations.

7.5.2 Missing values

Next, we give an overview of the missing values.

```
`d.disc.eggs_Rater level expert` %>%
  select(`answer.rater`, `MALDI-TOF determination`) %>%
  table(useNA = "always")
```

```
             MALDI-TOF determination
answer.rater  albopictus koreicus geniculatus japonicus <NA>
  albopictus          55        0           4         7    0
  koreicus             0       30           0         8    0
  geniculatus          1        2          56         7    0
  japonicus            1       25           0        36    0
  <NA>                 3        3           0         2    0
```

```
## Calculate the number of missing values in both index/reference test:
nr.NA <- `d.disc.eggs_Rater level expert` %>%
  select(`answer.rater`, `MALDI-TOF determination`) %>%
  (\(x){sum(is.na(x))})
## Calculate the number of observations that will be removed:
(nr.deleted.obs <- `d.disc.eggs_Rater level expert` %>%
    filter(is.na(`answer.rater`) | is.na(`MALDI-TOF determination`)) %>%
    nrow())
```

```
[1] 8
```

```
`d.disc.eggs_Rater level expert` <- `d.disc.eggs_Rater level expert` %>%
  filter(!is.na(`answer.rater`) & !is.na(`MALDI-TOF determination`))
```

There were 8 observations (rows) deleted due to missing values.

7.5.3 Confusion Matrix

We calculate the confusion matrices in the following steps.

```
tab <- xtabs(formula = ~ `answer.rater` + `MALDI-TOF determination`,
             data = `d.disc.eggs_Rater level expert`)
tab %>%
  f.create.confusion.matrix() %>%
  as_tibble(rownames = "answer.rater") %>%
  kable(caption = paste0("Overall cross table."),
        label = "tab:CrossTable5",
```

```
        booktabs = TRUE,
        longtable = TRUE,
        linesep = c("")) %>%
  kable_styling(font_size = 7,
                latex_options = c("striped", "repeat_header", "hold_position")) %>%
  row_spec(row = 5, bold = T) %>%
  column_spec(column = 6, bold = T) %>%
  add_header_above(c("", "MALDI-TOF determination" = 4, ""))
```

Table 30: Overall cross table.

	MALDI-TOF determination				
answer.rater	albopictus	koreicus	geniculatus	japonicus	Total
albopictus	55	0	4	7	**66**
koreicus	0	30	0	8	**38**
geniculatus	1	2	56	7	**66**
japonicus	1	25	0	36	**62**
**Total**	**57**	**57**	**60**	**58**	**232**

Example how to read this table:

- There were 55 cases of *albopictus* that were correctly classified as *albopictus*, 0 cases of *albopictus* were wrongly classified as *koreicus*.
- There were 30 cases of *koreicus* that were correctly classified as *koreicus*, 0 cases of *koreicus* were wrongly classified as *albopictus*.

We present the same table in percentages. Note that all columns add to 100%.

```
tab <- xtabs(formula = ~ `answer.rater` + `MALDI-TOF determination`,
             data = `d.disc.eggs_Rater level expert`)
prop.table(x = tab, margin = 2) %>%
  '*'(100) %>%
  rbind(., colSums(.)) %>%
  f.round(digits = digits.rounding) %>%
  kable(caption = paste0("Overall cross table in percentages. All columns add up to 100\\%."),
        label = "tab:CrossTablePercentage5",
        booktabs = TRUE,
        longtable = TRUE,
        linesep = c("")) %>%
  kable_styling(font_size = 7,
                latex_options = c("striped", "repeat_header", "hold_position")) %>%
  row_spec(row = 5, bold = T) %>%
  add_header_above(c("", "MALDI-TOF determination" = 4))
```

Table 31: Overall cross table in percentages. All columns add up to 100%.

	MALDI-TOF determination			
	albopictus	koreicus	geniculatus	japonicus
albopictus	96.49	0.00	6.67	12.07
koreicus	0.00	52.63	0.00	13.79
geniculatus	1.75	3.51	93.33	12.07
japonicus	1.75	43.86	0.00	62.07
	**100.00**	**100.00**	**100.00**	**100.00**

```
levels.chr <- levels(`d.disc.eggs_Rater level expert`$`answer.rater`)
if(length(levels.chr) == 2){
```

```
  classes <- c("")
}else{
  classes <- levels.chr
}

## calculate tables
if(identical(classes, c(""))){
  l.tab <- list(xtabs(formula = ~ `answer.rater` + `MALDI-TOF determination`,
                      data = `d.disc.eggs_Rater level expert`))
}else{
  l.tab <- lapply(X = classes, FUN = function(x){
    xtabs(formula = ~ `answer.rater` + `MALDI-TOF determination`,
          data = `d.disc.eggs_Rater level expert` %>%
            mutate(across(.cols = c(`answer.rater`, `MALDI-TOF determination`), .fns = function(y){
              ## we merge categories into the category "neg."
              y %>%
                fct_collapse("neg." = setdiff(levels(`d.disc.eggs_Rater level expert`$`answer.rater`), x
                fct_relevel(x)
            })))
  })
}
## calculate confusion matrix
l.confusion.matrix <- lapply(X = l.tab,
                             FUN = f.create.confusion.matrix)
## calculate tables for printing
l.confusion.matrix.print <- lapply(X = l.confusion.matrix,
                                   FUN = f.create.confusion.matrix.print)
```

The next tables show the confusion matrices for the index test ("answer.rater") against the reference test ("MALDI-TOF determination").

```
for(class in seq_along(classes)){
  l.confusion.matrix.print[[class]] %>%
    as_tibble(rownames = "answer.rater") %>%
    kable(caption = paste0("Confusion matrix for class ‘",
                           levels(`d.disc.eggs_Rater level expert`$`answer.rater`)[class], "’.
          Category ‘", levels(`d.disc.eggs_Rater level expert`$`answer.rater`)[class],
          "’ is considered a positive result.
          Category ‘neg.’ is considered a negative result and
          contains the levels ",
          paste0(setdiff(levels(`d.disc.eggs_Rater level expert`$`answer.rater`),
                         classes[class]), collapse = ", "), "."),
          label = paste0("tab:ConfusionMatrix5Class", class),
          booktabs = TRUE,
          longtable = TRUE,
          linesep = c("")) %>%
    kable_styling(font_size = 7,
                  latex_options = c("striped", "repeat_header", "hold_position")) %>%
    row_spec(row = nrow(l.confusion.matrix.print[[class]]), bold = T) %>%
    column_spec(column = ncol(l.confusion.matrix.print[[class]]) + 1, bold = T) %>%
    add_header_above(c("", "MALDI-TOF determination" =
                         ncol(l.confusion.matrix.print[[class]]) - 1, "")) %>%
    print()
```

```
}
```

Table 32: Confusion matrix for class 'albopictus'. Category 'albopictus' is considered a positive result. Category 'neg.' is considered a negative result and contains the levels koreicus, geniculatus, japonicus.

	MALDI-TOF determination		
answer.rater	albopictus	neg.	Total
albopictus	55 (TP)	11 (FP)	**66**
neg.	2 (FN)	164 (TN)	**166**
**Total**	**57**	**175**	**232**

Table 33: Confusion matrix for class 'koreicus'. Category 'koreicus' is considered a positive result. Category 'neg.' is considered a negative result and contains the levels albopictus, geniculatus, japonicus.

	MALDI-TOF determination		
answer.rater	koreicus	neg.	Total
koreicus	30 (TP)	8 (FP)	**38**
neg.	27 (FN)	167 (TN)	**194**
**Total**	**57**	**175**	**232**

Table 34: Confusion matrix for class 'geniculatus'. Category 'geniculatus' is considered a positive result. Category 'neg.' is considered a negative result and contains the levels albopictus, koreicus, japonicus.

	MALDI-TOF determination		
answer.rater	geniculatus	neg.	Total
geniculatus	56 (TP)	10 (FP)	**66**
neg.	4 (FN)	162 (TN)	**166**
**Total**	**60**	**172**	**232**

Table 35: Confusion matrix for class 'japonicus'. Category 'japonicus' is considered a positive result. Category 'neg.' is considered a negative result and contains the levels albopictus, koreicus, geniculatus.

	MALDI-TOF determination		
answer.rater	japonicus	neg.	Total
japonicus	36 (TP)	26 (FP)	**62**
neg.	22 (FN)	148 (TN)	**170**
**Total**	**58**	**174**	**232**

7.5.4 Measures of diagnostic accuracy

We now calculate measures of diagnostic accuracy.

```r
res.diagnostic.measures <- tibble("Class" = classes) %>%
  mutate(tabs = l.tab) %>%

  ## epiR wants it in the format: c(TP, FP, FN, TN)
  mutate(confusion.vector = map(.x = tabs, .f = function(x){
    c("tp" = x[1, 1], "fp" = x[1, 2], "fn" = x[2, 1], "tn" = x[2, 2])
  })) %>%

  ## Calculate measures of diagnostic accuracy
  mutate(out.EpiR = map(.x = confusion.vector, .f = function(x){
    epi.tests(dat = x,
              method = "wilson",
              conf.level = 0.95)
  })) %>%

  ## reformat output, filter diag. measures of interest
  mutate(diag.measures = map(.x = out.EpiR, .f = function(x){
    x <- x %>%
      summary() %>%
      as_tibble(rownames = "name.raw") %>%
      mutate()
    if(f.check.version(x = packageVersion(pkg = "epiR"))){
      x <- x %>%
        mutate(name.raw = statistic) %>%
        select(-statistic)
    }
    x %>%
      left_join(y = .match, by = c("name.raw")) %>%
      filter(name.raw %in% c("ap", "tp", "se", "sp", "diag.or", "pv.pos", "pv.neg", "lr.pos", "lr.neg",
  })) %>%

  ## ensure boundaries of estimates and CI
  mutate(diag.measures = map(.x = diag.measures, .f = function(x){
    x %>%
      mutate(across(.cols = c(est, lower, upper), .fns = function(x){
        if_else(condition = bounded, true = pmin(1, pmax(0, x)), false = x)
      }))
  })) %>%

  ## put together output
  mutate(diag.measures.print = map(.x = diag.measures, .f = function(x){
    x %>%
      mutate(across(.cols = c("est", "lower", "upper"),
                    .fns = \(x){format(x = round(x = x, digits = digits.rounding + 1),
                                       scientific = FALSE,
                                       nsmall = digits.rounding + 1)})) %>%
      mutate("Estimate (95% CI)" = paste0(est, " (from ", lower, " to ", upper, ")")) %>%
      select(Measure, `Estimate (95% CI)`)
  }))
```

We restructure the results that are to be printed.

```
aux.df.rows <- res.diagnostic.measures %>%
  select(Class, diag.measures.print) %>%
  mutate(Class = paste0("Class ", Class)) %>%
  unnest(cols = diag.measures.print) %>%
  mutate(row.nr = seq_len(n())) %>%
  group_by(Class) %>%
  summarize(min = min(row.nr),
            max = max(row.nr)) %>%
  ungroup()
```

We prepare the printing of the table.

```
res.diagnostic.measures_print5 <- res.diagnostic.measures %>%
  select(Class, diag.measures.print) %>%
  unnest(cols = diag.measures.print) %>%
  select(-Class) %>%

  kable(caption = paste0("Estimate and 95\\% confidence intervals for prevalence
                         and different measures of accuracy."),
        label = "tab:ResultTable5",
        booktabs = TRUE,
        longtable = TRUE,
        linesep = c("")) %>%
  kable_styling(
    font_size = 7,
    repeat_header_method = "replace",
    repeat_header_text = paste0("Estimate and 95\\% confidence intervals for prevalence
                                and different measures of accuracy. (continued)"),
    latex_options = c("striped", "repeat_header", "hold_position")
  ) %>%

  group_rows(group_label = aux.df.rows$Class[1],
             start_row = aux.df.rows$min[1],
             end_row = aux.df.rows$max[1]) %>%
  group_rows(group_label = aux.df.rows$Class[2],
             start_row = aux.df.rows$min[2],
             end_row = aux.df.rows$max[2]) %>%
  group_rows(group_label = aux.df.rows$Class[3],
             start_row = aux.df.rows$min[3],
             end_row = aux.df.rows$max[3]) %>%
  group_rows(group_label = aux.df.rows$Class[4],
             start_row = aux.df.rows$min[4],
             end_row = aux.df.rows$max[4]) %>%
  collapse_rows(columns = 1, latex_hline = "major", valign = "middle")
```

The next table shows point estimate and 95% confidence intervals for prevalence and different measures of diagnostic accuracy.

```
res.diagnostic.measures_print5
```

Table 36: Estimate and 95% confidence intervals for prevalence and different measures of accuracy.

Measure	Estimate (95% CI)
**Class albopictus**	
Apparent prevalence	0.284 (from 0.230 to 0.346)

Table 36: Estimate and 95% confidence intervals for prevalence and different measures of accuracy. (continued)

Measure	Estimate (95% CI)
True prevalence	0.246 (from 0.195 to 0.305)
Sensitivity	0.965 (from 0.881 to 0.990)
Specificity	0.937 (from 0.891 to 0.965)
Correctly classified proportion	0.944 (from 0.907 to 0.967)
Diagnostic odds ratio	410.000 (from 88.136 to 1907.282)
Positive predictive value	0.833 (from 0.726 to 0.904)
Negative predictive value	0.988 (from 0.957 to 0.997)
Positive likelihood ratio	15.351 (from 8.645 to 27.259)
Negative likelihood ratio	0.037 (from 0.010 to 0.146)
**Class koreicus**	
Apparent prevalence	0.164 (from 0.122 to 0.217)
True prevalence	0.246 (from 0.195 to 0.305)
Sensitivity	0.526 (from 0.399 to 0.650)
Specificity	0.954 (from 0.912 to 0.977)
Correctly classified proportion	0.849 (from 0.797 to 0.889)
Diagnostic odds ratio	23.194 (from 9.626 to 55.891)
Positive predictive value	0.789 (from 0.637 to 0.889)
Negative predictive value	0.861 (from 0.805 to 0.903)
Positive likelihood ratio	11.513 (from 5.602 to 23.661)
Negative likelihood ratio	0.496 (from 0.377 to 0.654)
**Class geniculatus**	
Apparent prevalence	0.284 (from 0.230 to 0.346)
True prevalence	0.259 (from 0.207 to 0.319)
Sensitivity	0.933 (from 0.841 to 0.974)
Specificity	0.942 (from 0.896 to 0.968)
Correctly classified proportion	0.940 (from 0.901 to 0.964)
Diagnostic odds ratio	226.800 (from 68.401 to 752.005)
Positive predictive value	0.848 (from 0.743 to 0.916)
Negative predictive value	0.976 (from 0.940 to 0.991)
Positive likelihood ratio	16.053 (from 8.764 to 29.406)
Negative likelihood ratio	0.071 (from 0.027 to 0.183)
**Class japonicus**	
Apparent prevalence	0.267 (from 0.214 to 0.328)
True prevalence	0.250 (from 0.199 to 0.309)
Sensitivity	0.621 (from 0.492 to 0.734)
Specificity	0.851 (from 0.790 to 0.896)
Correctly classified proportion	0.793 (from 0.736 to 0.840)
Diagnostic odds ratio	9.315 (from 4.745 to 18.286)
Positive predictive value	0.581 (from 0.457 to 0.695)
Negative predictive value	0.871 (from 0.812 to 0.913)
Positive likelihood ratio	4.154 (from 2.763 to 6.244)
Negative likelihood ratio	0.446 (from 0.319 to 0.623)

We save the created objects in a list.

```r
df.groups_list_dataset[["Rater level expert"]] <- `d.disc.eggs_Rater level expert`
df.groups_list_diag.measures[["Rater level expert"]] <- res.diagnostic.measures
```

We clean up the programming environment.

```r
rm(tab, nr.obs, l.confusion.matrix,
   l.confusion.matrix.print, res.diagnostic.measures,
   aux.df.rows)
```

7.6 We look at *Rater level non expert*

7.6.1 Dataset

```
## Dataset
`d.disc.eggs_Rater level non expert` <- d.disc.eggs %>% filter(`group` == "Rater level non expert")
## Number of observations
(nr.obs <- nrow(`d.disc.eggs_Rater level non expert`))
```

```
[1] 480
```

This dataset contains 480 observations.

7.6.2 Missing values

Next, we give an overview of the missing values.

```
`d.disc.eggs_Rater level non expert` %>%
  select(`answer.rater`, `MALDI-TOF determination`) %>%
  table(useNA = "always")
```

```
             MALDI-TOF determination
answer.rater  albopictus koreicus geniculatus japonicus <NA>
  albopictus         112        0           4        10    0
  koreicus             0       59           3        15    0
  geniculatus          3        7         109        18    0
  japonicus            5       53           2        74    0
  <NA>                 0        1           2         3    0
```

```
## Calculate the number of missing values in both index/reference test:
nr.NA <- `d.disc.eggs_Rater level non expert` %>%
  select(`answer.rater`, `MALDI-TOF determination`) %>%
  (\(x){sum(is.na(x))})
## Calculate the number of observations that will be removed:
(nr.deleted.obs <- `d.disc.eggs_Rater level non expert` %>%
    filter(is.na(`answer.rater`) | is.na(`MALDI-TOF determination`)) %>%
    nrow())
```

```
[1] 6
```

```
`d.disc.eggs_Rater level non expert` <- `d.disc.eggs_Rater level non expert` %>%
  filter(!is.na(`answer.rater`) & !is.na(`MALDI-TOF determination`))
```

There were 6 observations (rows) deleted due to missing values.

7.6.3 Confusion Matrix

We calculate the confusion matrices in the following steps.

```
tab <- xtabs(formula = ~ `answer.rater` + `MALDI-TOF determination`,
             data = `d.disc.eggs_Rater level non expert`)
tab %>%
  f.create.confusion.matrix() %>%
  as_tibble(rownames = "answer.rater") %>%
  kable(caption = paste0("Overall cross table."),
        label = "tab:CrossTable6",
```

```
        booktabs = TRUE,
        longtable = TRUE,
        linesep = c("")) %>%
  kable_styling(font_size = 7,
                latex_options = c("striped", "repeat_header", "hold_position")) %>%
  row_spec(row = 5, bold = T) %>%
  column_spec(column = 6, bold = T) %>%
  add_header_above(c("", "MALDI-TOF determination" = 4, ""))
```

Table 37: Overall cross table.

	MALDI-TOF determination				
answer.rater	albopictus	koreicus	geniculatus	japonicus	Total
albopictus	112	0	4	10	**126**
koreicus	0	59	3	15	**77**
geniculatus	3	7	109	18	**137**
japonicus	5	53	2	74	**134**
**Total**	**120**	**119**	**118**	**117**	**474**

Example how to read this table:

- There were 112 cases of *albopictus* that were correctly classified as *albopictus*, 0 cases of *albopictus* were wrongly classified as *koreicus*.
- There were 59 cases of *koreicus* that were correctly classified as *koreicus*, 0 cases of *koreicus* were wrongly classified as *albopictus*.

We present the same table in percentages. Note that all columns add to 100%.

```
tab <- xtabs(formula = ~ `answer.rater` + `MALDI-TOF determination`,
             data = `d.disc.eggs_Rater level non expert`)
prop.table(x = tab, margin = 2) %>%
  '*'(100) %>%
  rbind(., colSums(.)) %>%
  f.round(digits = digits.rounding) %>%
  kable(caption = paste0("Overall cross table in percentages. All columns add up to 100\\%."),
        label = "tab:CrossTablePercentage6",
        booktabs = TRUE,
        longtable = TRUE,
        linesep = c("")) %>%
  kable_styling(font_size = 7,
                latex_options = c("striped", "repeat_header", "hold_position")) %>%
  row_spec(row = 5, bold = T) %>%
  add_header_above(c("", "MALDI-TOF determination" = 4))
```

Table 38: Overall cross table in percentages. All columns add up to 100%.

	MALDI-TOF determination			
	albopictus	koreicus	geniculatus	japonicus
albopictus	93.33	0.00	3.39	8.55
koreicus	0.00	49.58	2.54	12.82
geniculatus	2.50	5.88	92.37	15.38
japonicus	4.17	44.54	1.69	63.25
	**100.00**	**100.00**	**100.00**	**100.00**

```
levels.chr <- levels(`d.disc.eggs_Rater level non expert`$`answer.rater`)
if(length(levels.chr) == 2){
```

```
  classes <- c("")
}else{
  classes <- levels.chr
}

## calculate tables
if(identical(classes, c(""))){
  l.tab <- list(xtabs(formula = ~ `answer.rater` + `MALDI-TOF determination`,
                      data = `d.disc.eggs_Rater level non expert`))
}else{
  l.tab <- lapply(X = classes, FUN = function(x){
    xtabs(formula = ~ `answer.rater` + `MALDI-TOF determination`,
          data = `d.disc.eggs_Rater level non expert` %>%
            mutate(across(.cols = c(`answer.rater`, `MALDI-TOF determination`), .fns = function(y){
              ## we merge categories into the category "neg."
              y %>%
                fct_collapse("neg." = setdiff(levels(`d.disc.eggs_Rater level non expert`$`answer.rater
                fct_relevel(x)
            })))
  })
}
## calculate confusion matrix
l.confusion.matrix <- lapply(X = l.tab,
                             FUN = f.create.confusion.matrix)
## calculate tables for printing
l.confusion.matrix.print <- lapply(X = l.confusion.matrix,
                                   FUN = f.create.confusion.matrix.print)
```

The next tables show the confusion matrices for the index test ("answer.rater") against the reference test ("MALDI-TOF determination").

```
for(class in seq_along(classes)){
  l.confusion.matrix.print[[class]] %>%
    as_tibble(rownames = "answer.rater") %>%
    kable(caption = paste0("Confusion matrix for class ‘",
                           levels(`d.disc.eggs_Rater level non expert`$`answer.rater`)[class], "’.
          Category ‘", levels(`d.disc.eggs_Rater level non expert`$`answer.rater`)[class],
          "’ is considered a positive result.
          Category ‘neg.’ is considered a negative result and
          contains the levels ",
          paste0(setdiff(levels(`d.disc.eggs_Rater level non expert`$`answer.rater`),
                         classes[class]), collapse = ", "), "."),
          label = paste0("tab:ConfusionMatrix6Class", class),
          booktabs = TRUE,
          longtable = TRUE,
          linesep = c("")) %>%
    kable_styling(font_size = 7,
                  latex_options = c("striped", "repeat_header", "hold_position")) %>%
    row_spec(row = nrow(l.confusion.matrix.print[[class]]), bold = T) %>%
    column_spec(column = ncol(l.confusion.matrix.print[[class]]) + 1, bold = T) %>%
    add_header_above(c("", "MALDI-TOF determination" =
                         ncol(l.confusion.matrix.print[[class]]) - 1, "")) %>%
    print()
```

```
}
```

Table 39: Confusion matrix for class ‘albopictus’. Category ‘albopictus’ is considered a positive result. Category ‘neg.’ is considered a negative result and contains the levels koreicus, geniculatus, japonicus.

	MALDI-TOF determination		
answer.rater	albopictus	neg.	Total
albopictus	112 (TP)	14 (FP)	**126**
neg.	8 (FN)	340 (TN)	**348**
**Total**	**120**	**354**	**474**

Table 40: Confusion matrix for class ‘koreicus’. Category ‘koreicus’ is considered a positive result. Category ‘neg.’ is considered a negative result and contains the levels albopictus, geniculatus, japonicus.

	MALDI-TOF determination		
answer.rater	koreicus	neg.	Total
koreicus	59 (TP)	18 (FP)	**77**
neg.	60 (FN)	337 (TN)	**397**
**Total**	**119**	**355**	**474**

Table 41: Confusion matrix for class ‘geniculatus’. Category ‘geniculatus’ is considered a positive result. Category ‘neg.’ is considered a negative result and contains the levels albopictus, koreicus, japonicus.

	MALDI-TOF determination		
answer.rater	geniculatus	neg.	Total
geniculatus	109 (TP)	28 (FP)	**137**
neg.	9 (FN)	328 (TN)	**337**
**Total**	**118**	**356**	**474**

Table 42: Confusion matrix for class ‘japonicus’. Category ‘japonicus’ is considered a positive result. Category ‘neg.’ is considered a negative result and contains the levels albopictus, koreicus, geniculatus.

	MALDI-TOF determination		
answer.rater	japonicus	neg.	Total
japonicus	74 (TP)	60 (FP)	**134**
neg.	43 (FN)	297 (TN)	**340**
**Total**	**117**	**357**	**474**

7.6.4 Measures of diagnostic accuracy

We now calculate measures of diagnostic accuracy.

```
res.diagnostic.measures <- tibble("Class" = classes) %>%
  mutate(tabs = l.tab) %>%

  ## epiR wants it in the format: c(TP, FP, FN, TN)
  mutate(confusion.vector = map(.x = tabs, .f = function(x){
    c("tp" = x[1, 1], "fp" = x[1, 2], "fn" = x[2, 1], "tn" = x[2, 2])
  })) %>%

  ## Calculate measures of diagnostic accuracy
  mutate(out.EpiR = map(.x = confusion.vector, .f = function(x){
    epi.tests(dat = x,
              method = "wilson",
              conf.level = 0.95)
  })) %>%

  ## reformat output, filter diag. measures of interest
  mutate(diag.measures = map(.x = out.EpiR, .f = function(x){
    x <- x %>%
      summary() %>%
      as_tibble(rownames = "name.raw") %>%
      mutate()
    if(f.check.version(x = packageVersion(pkg = "epiR"))){
      x <- x %>%
        mutate(name.raw = statistic) %>%
        select(-statistic)
    }
    x %>%
      left_join(y = .match, by = c("name.raw")) %>%
      filter(name.raw %in% c("ap", "tp", "se", "sp", "diag.or", "pv.pos", "pv.neg", "lr.pos", "lr.neg",
  })) %>%

  ## ensure boundaries of estimates and CI
  mutate(diag.measures = map(.x = diag.measures, .f = function(x){
    x %>%
      mutate(across(.cols = c(est, lower, upper), .fns = function(x){
        if_else(condition = bounded, true = pmin(1, pmax(0, x)), false = x)
      }))
  })) %>%

  ## put together output
  mutate(diag.measures.print = map(.x = diag.measures, .f = function(x){
    x %>%
      mutate(across(.cols = c("est", "lower", "upper"),
                    .fns = \(x){format(x = round(x = x, digits = digits.rounding + 1),
                                       scientific = FALSE,
                                       nsmall = digits.rounding + 1)})) %>%
      mutate("Estimate (95% CI)" = paste0(est, " (from ", lower, " to ", upper, ")")) %>%
      select(Measure, `Estimate (95% CI)`)
  }))
```

We restructure the results that are to be printed.

```r
aux.df.rows <- res.diagnostic.measures %>%
  select(Class, diag.measures.print) %>%
  mutate(Class = paste0("Class ", Class)) %>%
  unnest(cols = diag.measures.print) %>%
  mutate(row.nr = seq_len(n())) %>%
  group_by(Class) %>%
  summarize(min = min(row.nr),
            max = max(row.nr)) %>%
  ungroup()
```

We prepare the printing of the table.

```r
res.diagnostic.measures_print6 <- res.diagnostic.measures %>%
  select(Class, diag.measures.print) %>%
  unnest(cols = diag.measures.print) %>%
  select(-Class) %>%

  kable(caption = paste0("Estimate and 95\\% confidence intervals for prevalence
                         and different measures of accuracy."),
        label = "tab:ResultTable6",
        booktabs = TRUE,
        longtable = TRUE,
        linesep = c("")) %>%
  kable_styling(
    font_size = 7,
    repeat_header_method = "replace",
    repeat_header_text = paste0("Estimate and 95\\% confidence intervals for prevalence
                                and different measures of accuracy. (continued)"),
    latex_options = c("striped", "repeat_header", "hold_position")
  ) %>%

  group_rows(group_label = aux.df.rows$Class[1],
             start_row = aux.df.rows$min[1],
             end_row = aux.df.rows$max[1]) %>%
  group_rows(group_label = aux.df.rows$Class[2],
             start_row = aux.df.rows$min[2],
             end_row = aux.df.rows$max[2]) %>%
  group_rows(group_label = aux.df.rows$Class[3],
             start_row = aux.df.rows$min[3],
             end_row = aux.df.rows$max[3]) %>%
  group_rows(group_label = aux.df.rows$Class[4],
             start_row = aux.df.rows$min[4],
             end_row = aux.df.rows$max[4]) %>%
  collapse_rows(columns = 1, latex_hline = "major", valign = "middle")
```

The next table shows point estimate and 95% confidence intervals for prevalence and different measures of diagnostic accuracy.

```r
res.diagnostic.measures_print6
```

Table 43: Estimate and 95% confidence intervals for prevalence and different measures of accuracy.

Measure	Estimate (95% CI)
**Class albopictus**	
Apparent prevalence	0.266 (from 0.228 to 0.307)

Table 43: Estimate and 95% confidence intervals for prevalence and different measures of accuracy. (continued)

Measure	Estimate (95% CI)
True prevalence	0.253 (from 0.216 to 0.294)
Sensitivity	0.933 (from 0.874 to 0.966)
Specificity	0.960 (from 0.935 to 0.976)
Correctly classified proportion	0.954 (from 0.931 to 0.969)
Diagnostic odds ratio	340.000 (from 138.993 to 831.696)
Positive predictive value	0.889 (from 0.822 to 0.933)
Negative predictive value	0.977 (from 0.955 to 0.988)
Positive likelihood ratio	23.600 (from 14.093 to 39.521)
Negative likelihood ratio	0.069 (from 0.036 to 0.136)
**Class koreicus**	
Apparent prevalence	0.162 (from 0.132 to 0.198)
True prevalence	0.251 (from 0.214 to 0.292)
Sensitivity	0.496 (from 0.408 to 0.584)
Specificity	0.949 (from 0.921 to 0.968)
Correctly classified proportion	0.835 (from 0.799 to 0.866)
Diagnostic odds ratio	18.410 (from 10.155 to 33.376)
Positive predictive value	0.766 (from 0.660 to 0.847)
Negative predictive value	0.849 (from 0.810 to 0.881)
Positive likelihood ratio	9.778 (from 6.019 to 15.885)
Negative likelihood ratio	0.531 (from 0.444 to 0.636)
**Class geniculatus**	
Apparent prevalence	0.289 (from 0.250 to 0.331)
True prevalence	0.249 (from 0.212 to 0.290)
Sensitivity	0.924 (from 0.861 to 0.959)
Specificity	0.921 (from 0.889 to 0.945)
Correctly classified proportion	0.922 (from 0.894 to 0.943)
Diagnostic odds ratio	141.873 (from 64.928 to 310.003)
Positive predictive value	0.796 (from 0.720 to 0.855)
Negative predictive value	0.973 (from 0.950 to 0.986)
Positive likelihood ratio	11.745 (from 8.200 to 16.822)
Negative likelihood ratio	0.083 (from 0.044 to 0.155)
**Class japonicus**	
Apparent prevalence	0.283 (from 0.244 to 0.325)
True prevalence	0.247 (from 0.210 to 0.288)
Sensitivity	0.632 (from 0.542 to 0.714)
Specificity	0.832 (from 0.790 to 0.867)
Correctly classified proportion	0.783 (from 0.743 to 0.817)
Diagnostic odds ratio	8.519 (from 5.339 to 13.591)
Positive predictive value	0.552 (from 0.468 to 0.634)
Negative predictive value	0.874 (from 0.834 to 0.905)
Positive likelihood ratio	3.763 (from 2.876 to 4.925)
Negative likelihood ratio	0.442 (from 0.347 to 0.563)

We save the created objects in a list.

```
df.groups_list_dataset[["Rater level non expert"]] <- `d.disc.eggs_Rater level non expert`
df.groups_list_diag.measures[["Rater level non expert"]] <- res.diagnostic.measures
```

We clean up the programming environment.

```
rm(tab, nr.obs, l.confusion.matrix,
   l.confusion.matrix.print, res.diagnostic.measures,
   aux.df.rows)
```

8 Graphical overview

We display an overview over all bounded measures of diagnostic accuracy.

First, we collect all information into one dataframe.

```
d.overview.measures <- bind_rows(df.groups_list_diag.measures, .id = "id") %>%
  unnest(diag.measures)
# str(d.overview.measures)

d.overview.measures <- d.overview.measures %>%
  mutate(id = factor(x = id,
                     levels = c("Overall",
                                "Quality high", "Quality medium", "Quality low",
                                "Rater level expert", "Rater level non expert"),
                     labels = c("Overall",
                                "Quality high", "Quality medium", "Quality low",
                                "Rater level expert", "Rater level non expert")))
## assign factor levels
```

8.1 Compare groups (overall, quality, rater level)

In this section, we focus on comparing the groups (overall, quality, rater level) separately for each species.

```
d.overview.measures %>%
  mutate(Class = paste0("Ae. ", Class)) %>%
  # filter(Measure %in% c("Sensitivity", "Specificity")) %>%

  filter(bounded) %>%
  filter( ! (Measure %in% c("Apparent prevalence", "True prevalence"))) %>%
  # print() %>%

  ggplot(aes(x = id, y = est, color = id)) +
  facet_grid(Measure ~ Class, scales = "free", labeller = label_wrap_gen()) +
  geom_point() +
  geom_hline(yintercept = c(0, 1), linetype = "dashed") +
  geom_errorbar(aes(ymin = lower, ymax = upper)) +
  theme(axis.text.x = element_text(angle = 45, vjust = 1, hjust = 1)) +
  # theme(strip.text.x = element_text(size = 6)) +
  theme(legend.position = "none") +
  labs(x = "", y = "Estimate and 95% CI")
```

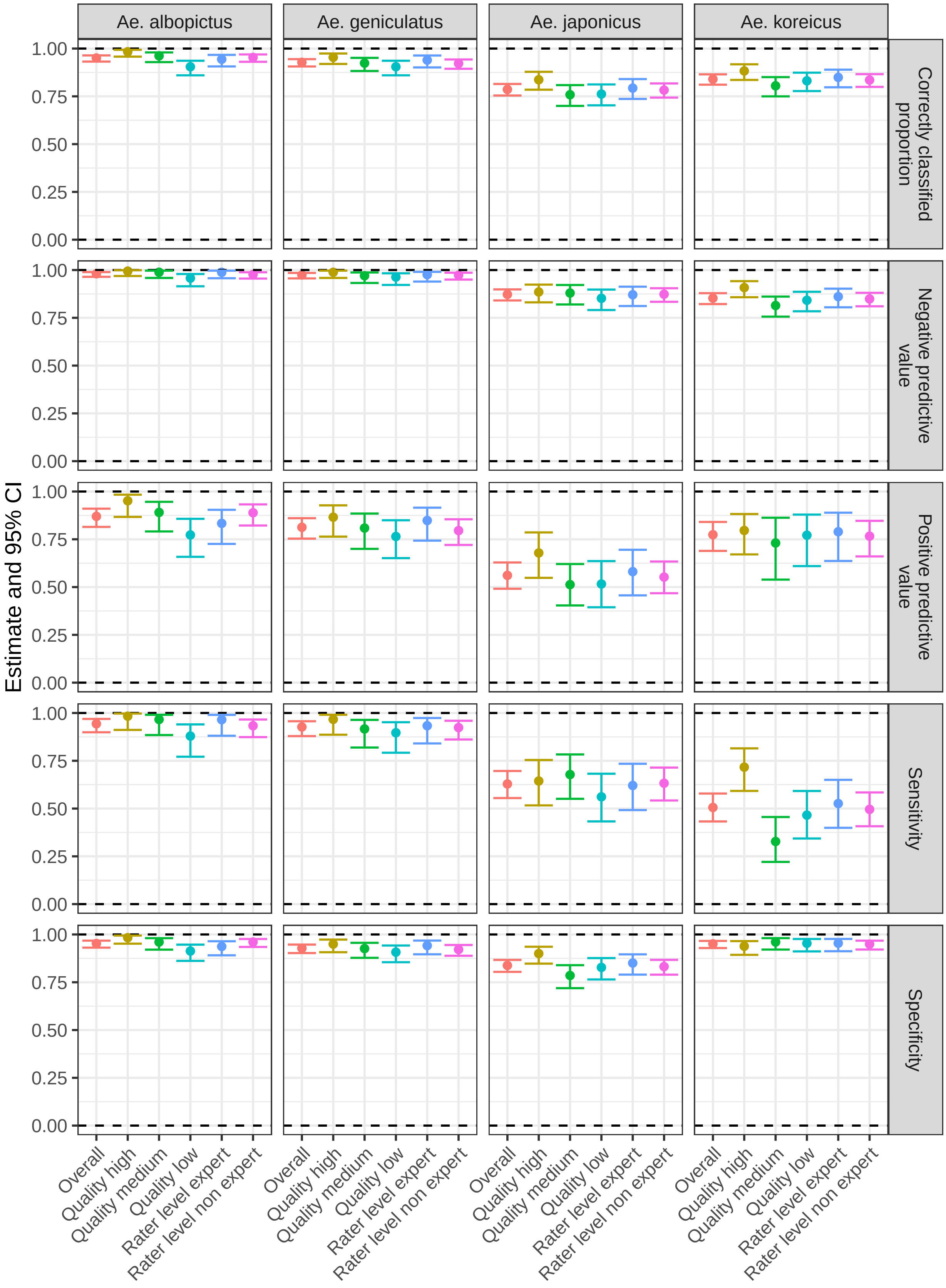

For the publication, we create a reduced plot, showing only sensitivity and specificity.

```
d.overview.measures %>%
  mutate(Class = paste0("Ae. ", Class)) %>%
  filter(Measure %in% c("Sensitivity", "Specificity")) %>%

  filter(bounded) %>%
  filter( ! (Measure %in% c("Apparent prevalence", "True prevalence"))) %>%
  # print() %>%

  ggplot(aes(x = id, y = est, color = id)) +
  facet_grid(Measure ~ Class, scales = "free", labeller = label_bquote(col = italic(.(Class)))) + # , l
  geom_point() +
  geom_hline(yintercept = c(0, 1), linetype = "dashed") +
  geom_errorbar(aes(ymin = lower, ymax = upper)) +
  theme(axis.text.x = element_text(angle = 45, vjust = 1, hjust = 1)) +
  # theme(strip.text.x = element_text(size = 6)) +
  theme(legend.position = "none") +
  labs(x = "", y = "Estimate and 95% CI")
```

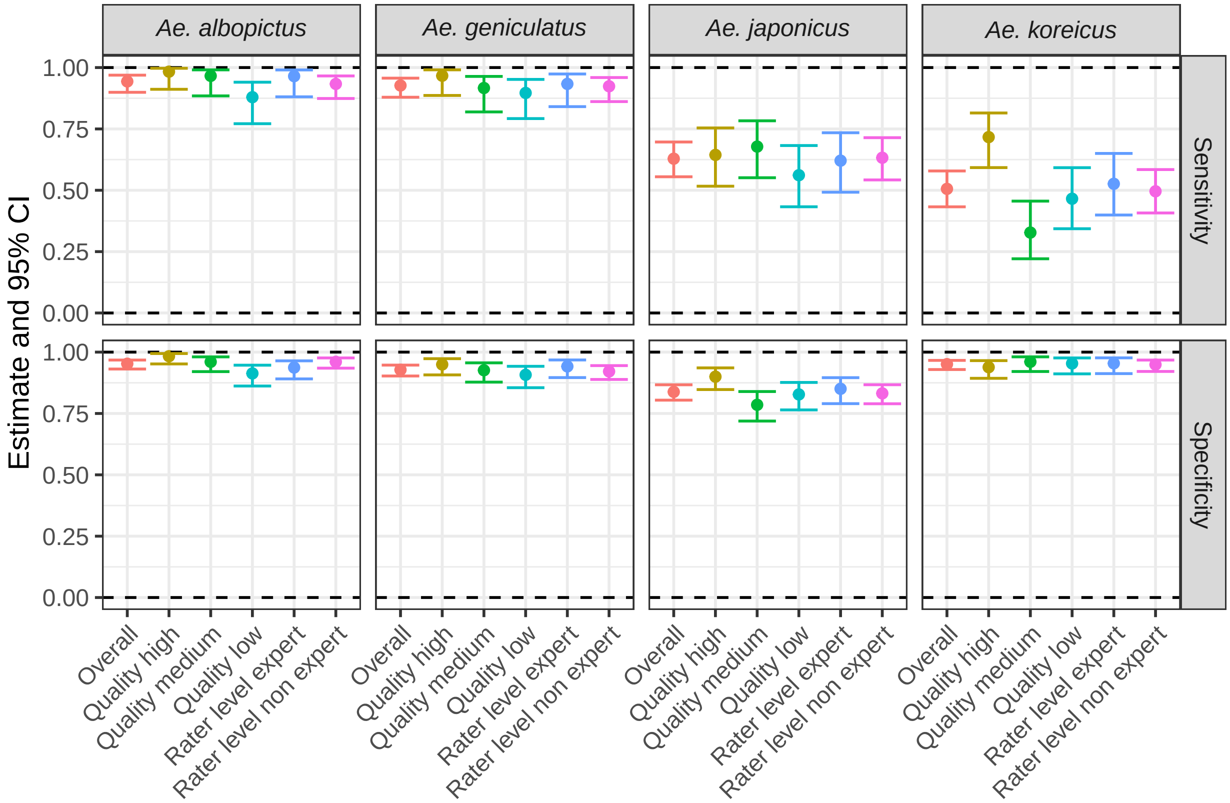

8.2 Compare species

In this section, we focus on comparing the four different species.

```
d.overview.measures %>%
  mutate(Class = paste0("Ae. ", Class)) %>%
  # filter(Measure %in% c("Sensitivity", "Specificity")) %>%

  filter(bounded) %>%
  filter( ! (Measure %in% c("Apparent prevalence", "True prevalence"))) %>%
```

```
# print() %>%

ggplot(aes(x = Class, y = est, color = Class)) +
## we fix the labelling linebreak dependent on the axis
# facet_grid(Measure ~ id, scales = "free", labeller = label_wrap_gen()) +
facet_grid(Measure ~ id, scales = "free",
           labeller = labeller(Measure = label_wrap_gen(),
                               id = label_wrap_gen(width = 14))) +
geom_point() +
geom_hline(yintercept = c(0, 1), linetype = "dashed") +
geom_errorbar(aes(ymin = lower, ymax = upper)) +
theme(axis.text.x = element_text(angle = 45, vjust = 1, hjust = 1)) +
# theme(strip.text.x = element_text(size = 6)) +
theme(legend.position = "none") +
labs(x = "", y = "Estimate and 95% CI") ## title = "Overview - comparing species",
```

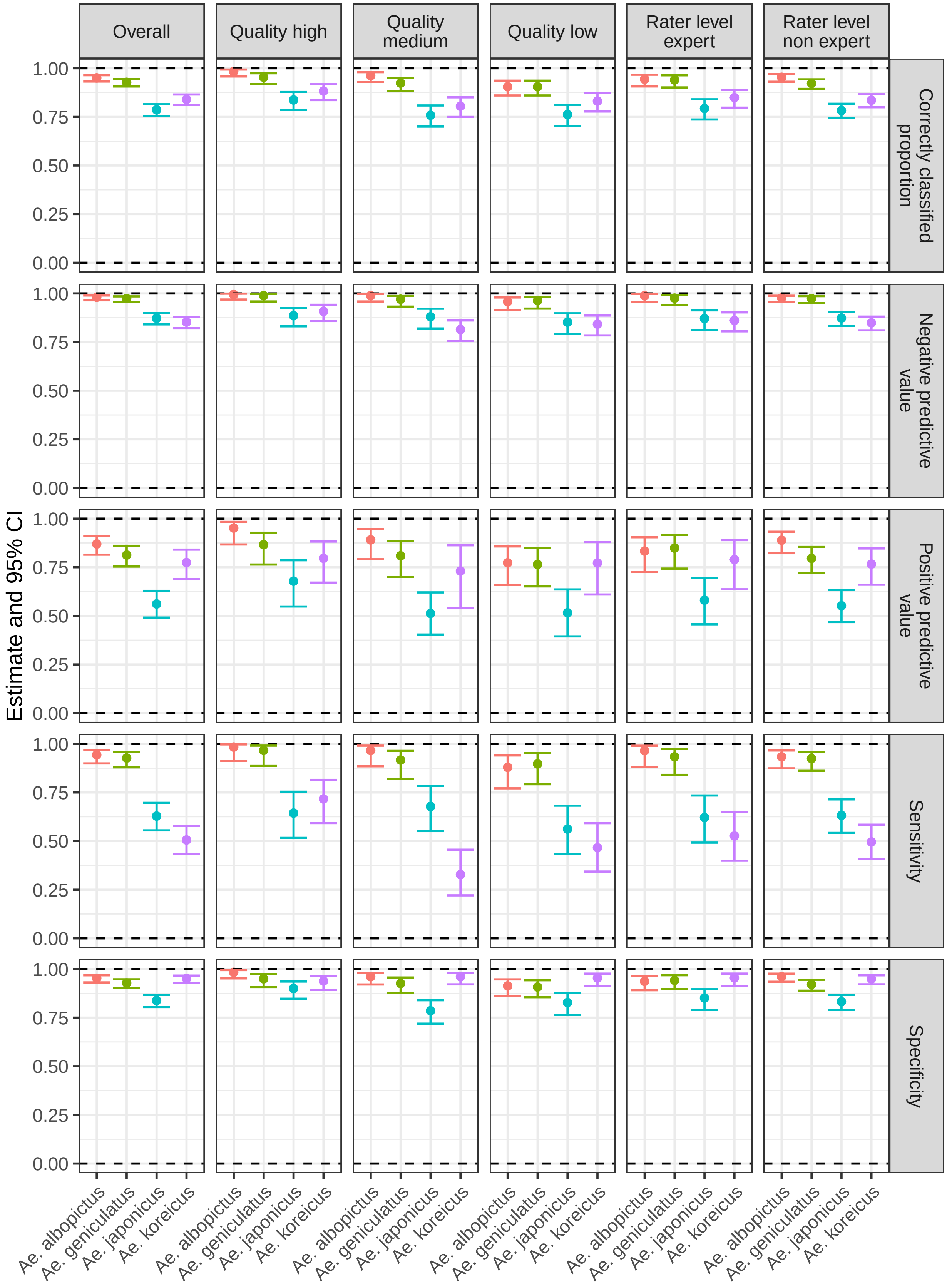

For the publication, we create a reduced plot, showing only sensitivity and specificity.

```r
d.overview.measures %>%
  mutate(Class = paste0("Ae. ", Class)) %>%
  filter(Measure %in% c("Sensitivity", "Specificity")) %>%

  filter(bounded) %>%
  filter( ! (Measure %in% c("Apparent prevalence", "True prevalence"))) %>%
  # print() %>%

  ggplot(aes(x = Class, y = est, color = Class)) +
  ## we fix the labelling linebreak dependent on the axis
  # facet_grid(Measure ~ id, scales = "free", labeller = label_wrap_gen()) +
  facet_grid(Measure ~ id, scales = "free",
             labeller = labeller(Measure = label_wrap_gen(),
                                 id = label_wrap_gen(width = 14))) +
  geom_point() +
  geom_hline(yintercept = c(0, 1), linetype = "dashed") +
  geom_errorbar(aes(ymin = lower, ymax = upper)) +
  theme(axis.text.x = element_text(angle = 45, vjust = 1, hjust = 1)) +
  # theme(strip.text.x = element_text(size = 6)) +
  theme(legend.position = "none") +
  labs(x = "", y = "Estimate and 95% CI") ## title = "Overview - comparing species",
```

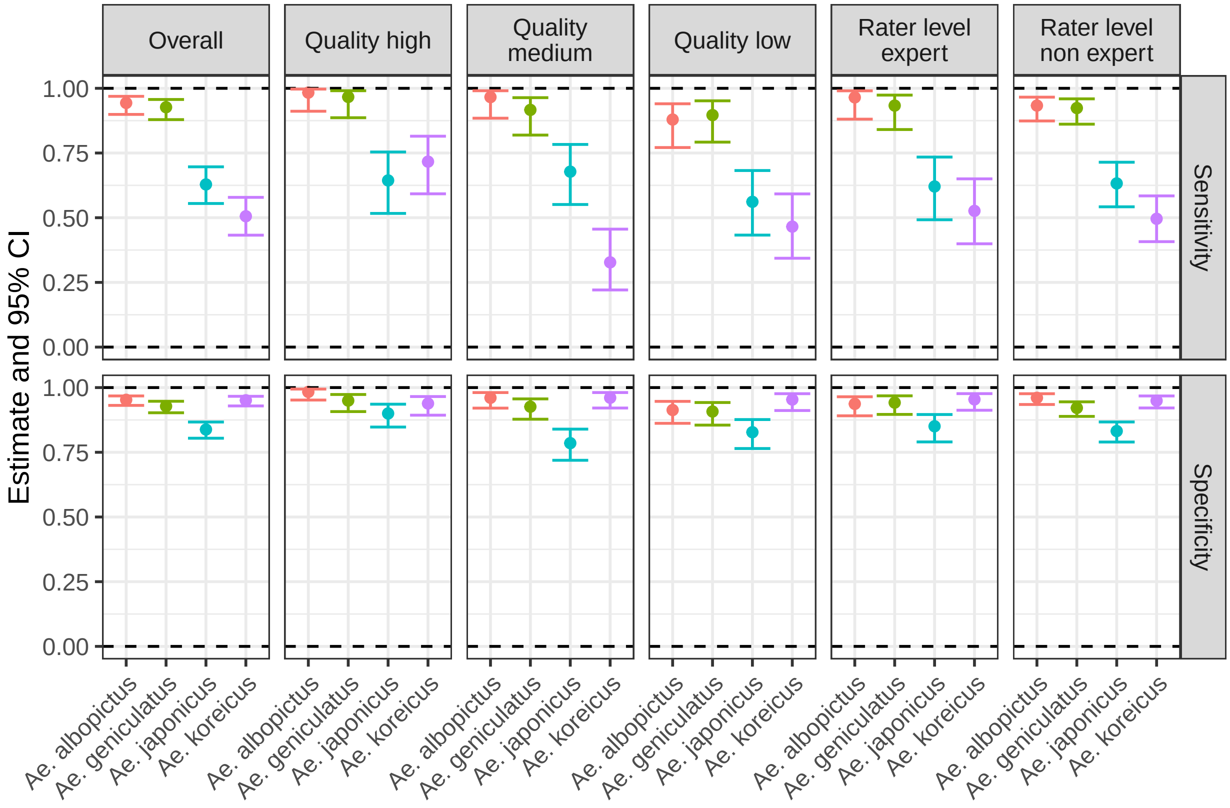

8.3 Compare rater levels

In this section, we focus on comparing the two rater levels (expert, non-expert).

```r
d.overview.measures %>%
  mutate(Class = paste0("Ae. ", Class)) %>%
```

```r
# filter(Measure %in% c("Sensitivity", "Specificity")) %>%

## now we have to filter "Overall"
filter(id %in% c("Rater level expert", "Rater level non expert")) %>%
filter(bounded) %>%
filter( ! (Measure %in% c("Apparent prevalence", "True prevalence"))) %>%
# print() %>%

ggplot(aes(x = id, y = est, color = id)) +
facet_grid(Measure ~ Class, scales = "free", labeller = label_wrap_gen()) +
geom_point() +
geom_hline(yintercept = c(0, 1), linetype = "dashed") +
geom_errorbar(aes(ymin = lower, ymax = upper)) +
theme(axis.text.x = element_text(angle = 45, vjust = 1, hjust = 1)) +
# theme(strip.text.x = element_text(size = 6)) +
theme(legend.position = "none") +
labs(x = "", y = "Estimate and 95% CI") ## title = "Overview - comparing rater levels",
```

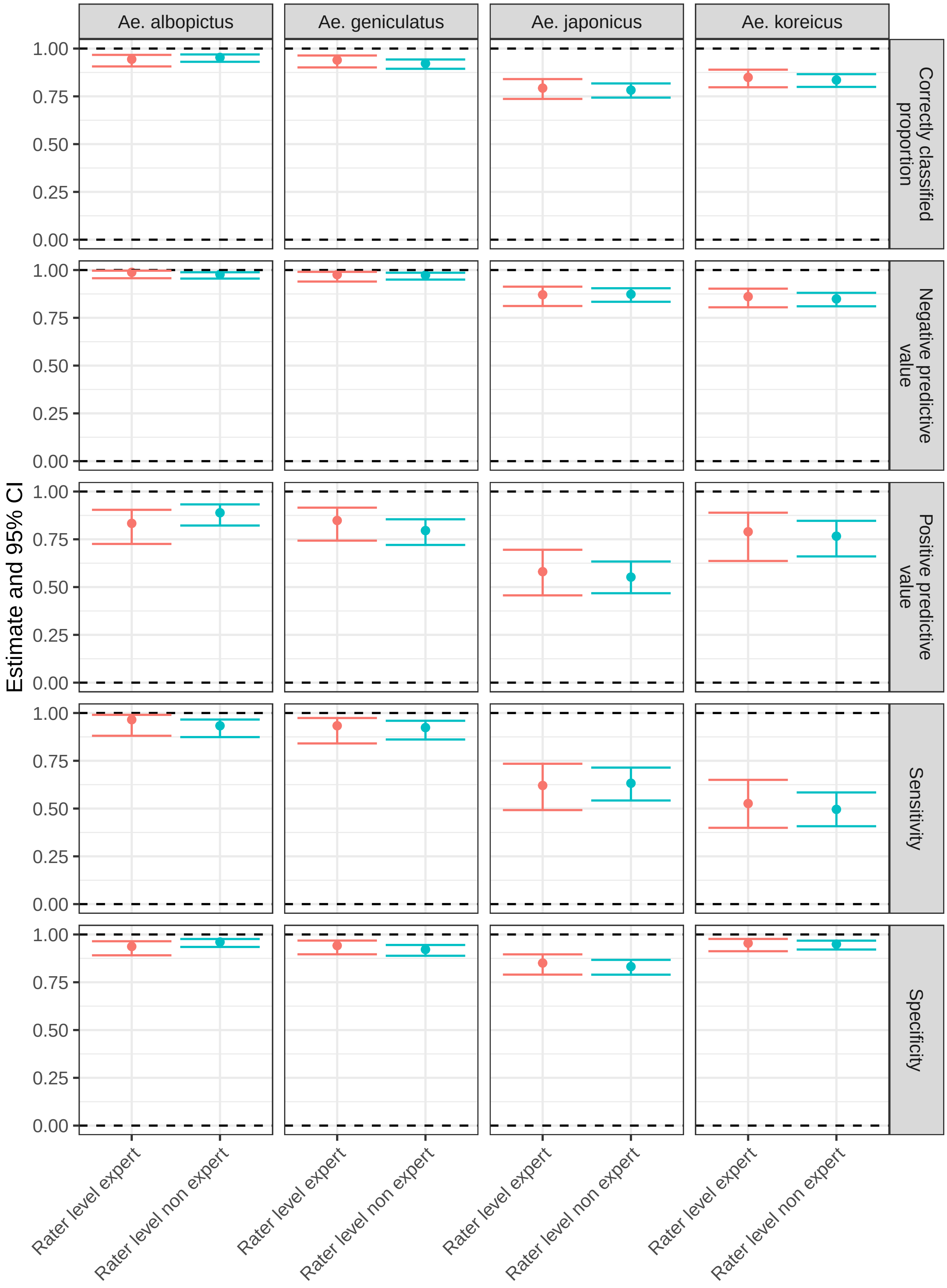

Ae. albopictus
Ae. geniculatus
Ae. japonicus
Ae. koreicus
Correctly classified proportion
Negative predictive value
Positive predictive value
Sensitivity
Specificity
Estimate and 95% CI
1.00
0.75
0.50
0.25
0.00
Rater level expert
Rater level non expert

8.4 Compare quality levels

In this section, we focus on comparing the three quality levels (high, medium, low).

```
d.overview.measures %>%

  ## now we have to filter "Overall"
  filter(id %in% c("Quality high", "Quality medium", "Quality low")) %>%
  filter(bounded) %>%
  filter( ! (Measure %in% c("Apparent prevalence", "True prevalence"))) %>%
  # print() %>%

  ggplot(aes(x = id, y = est, color = id)) +
  facet_grid(Measure ~ Class, scales = "free", labeller = label_wrap_gen()) +
  geom_point() +
  geom_hline(yintercept = c(0, 1), linetype = "dashed") +
  geom_errorbar(aes(ymin = lower, ymax = upper)) +
  theme(axis.text.x = element_text(angle = 45, vjust = 1, hjust = 1)) +
  # theme(strip.text.x = element_text(size = 6)) +
  theme(legend.position = "none") +
  labs(title = "Overview - quality levels", x = "", y = "Estimate and 95% CI")
```

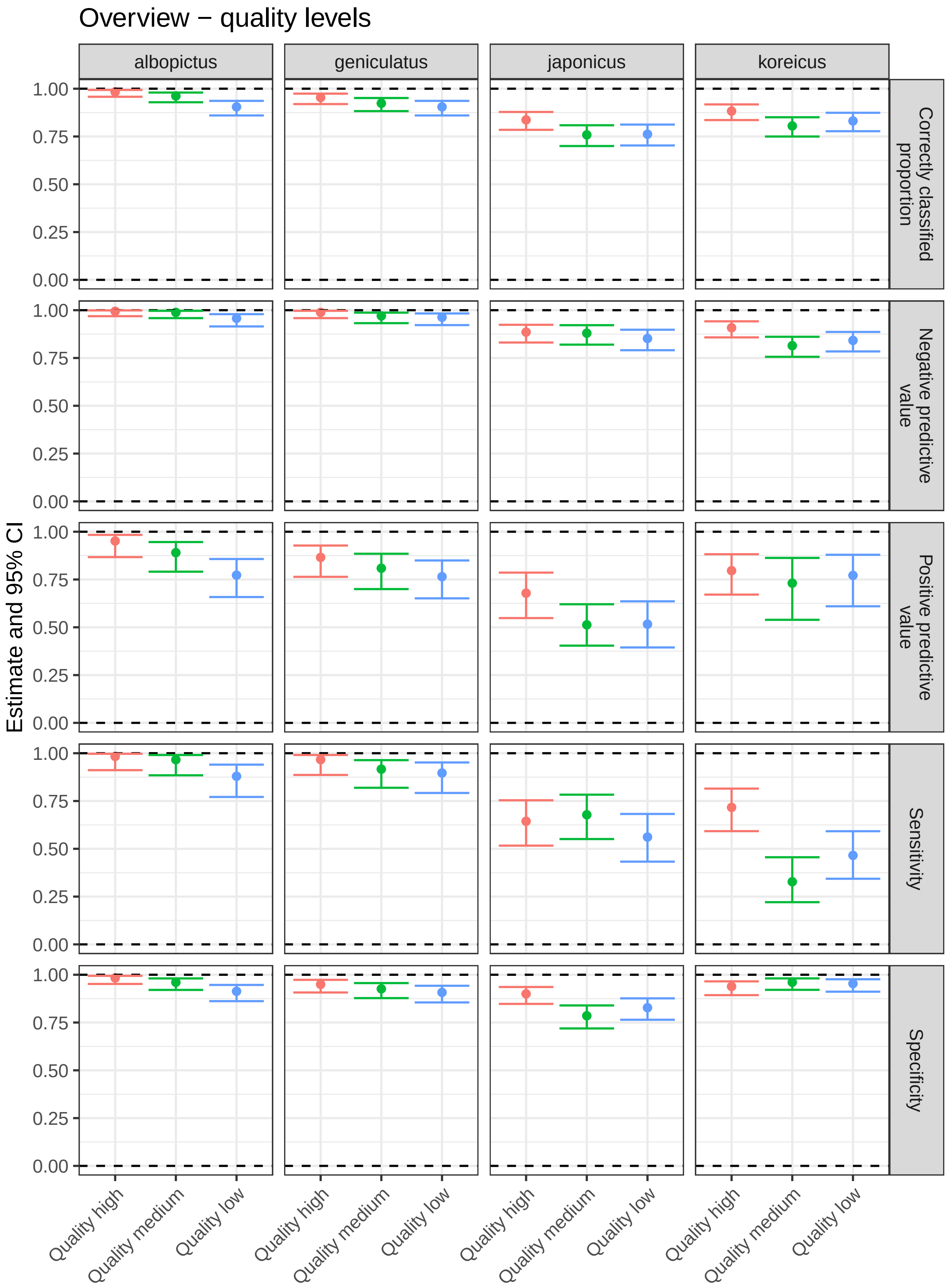
Overview − quality levels
albopictus
geniculatus
japonicus
koreicus
Correctly classified proportion
Negative predictive value
Positive predictive value
Sensitivity
Specificity
Estimate and 95% CI
0.00
0.25
0.50
0.75
1.00
Quality high
Quality medium
Quality low

9 Time spent on rating

We assess the time spent for classifying all 24 pictures (unit is minute).

```
## We calculate some summary statistics over it
d.disc.eggs %>%
  distinct(id, .keep_all = TRUE) %>%
  pull(time_minutes) %>%
  summary()
```

```
   Min. 1st Qu.  Median    Mean 3rd Qu.    Max.
    5.0     9.0    13.0    13.3    16.5    30.0
```

Raters spent on average `13.3` minutes for all 24 images.

We visualize the time spent

```
d.disc.eggs %>%
  distinct(id, .keep_all = TRUE) %>%
  ggplot(aes(x = time_minutes)) +
  geom_rug(alpha = 0.3) +
  scale_x_continuous(limits = c(0, NA)) +
  geom_density()
```

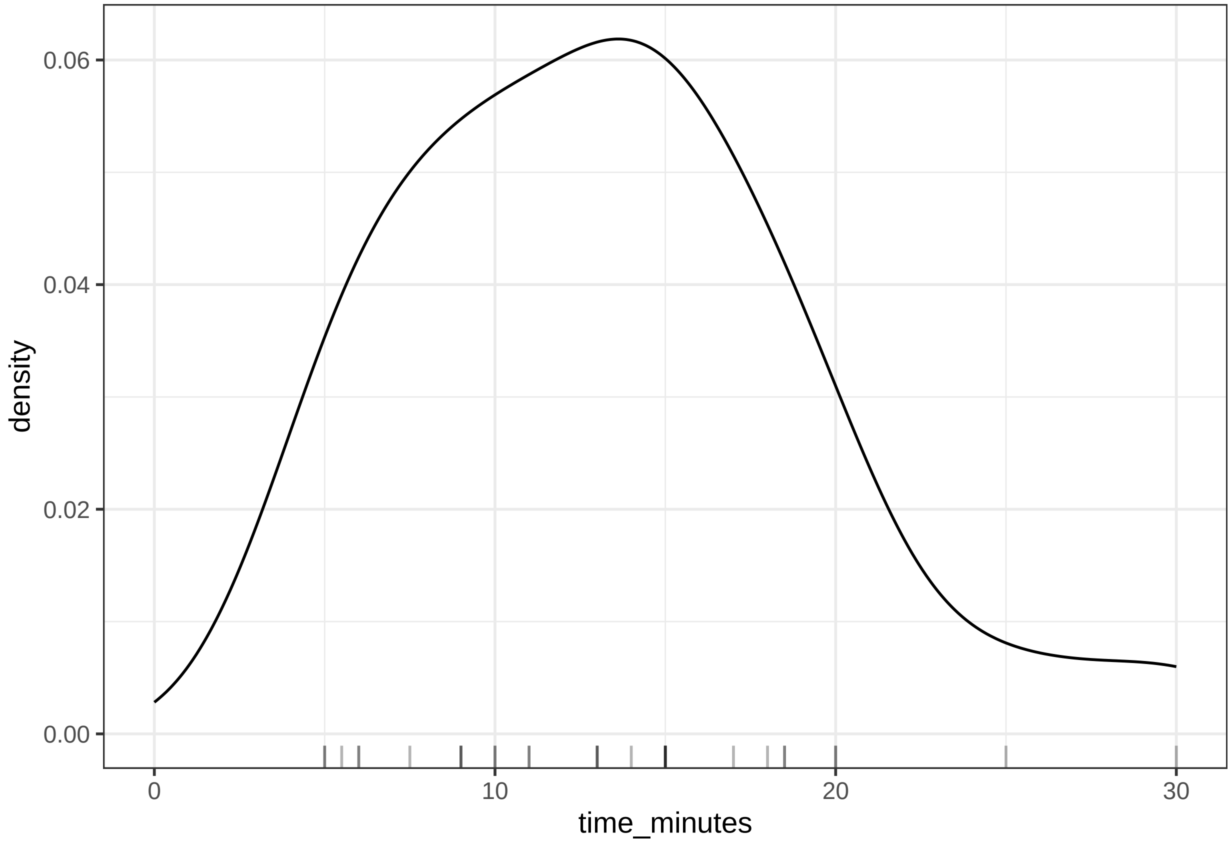


```
d.disc.eggs %>%
  distinct(id, .keep_all = TRUE) %>%
  ggplot(aes(x = "", y = time_minutes)) +
  geom_boxplot() +
  ggbeeswarm::geom_beeswarm(groupOnX = TRUE)
```

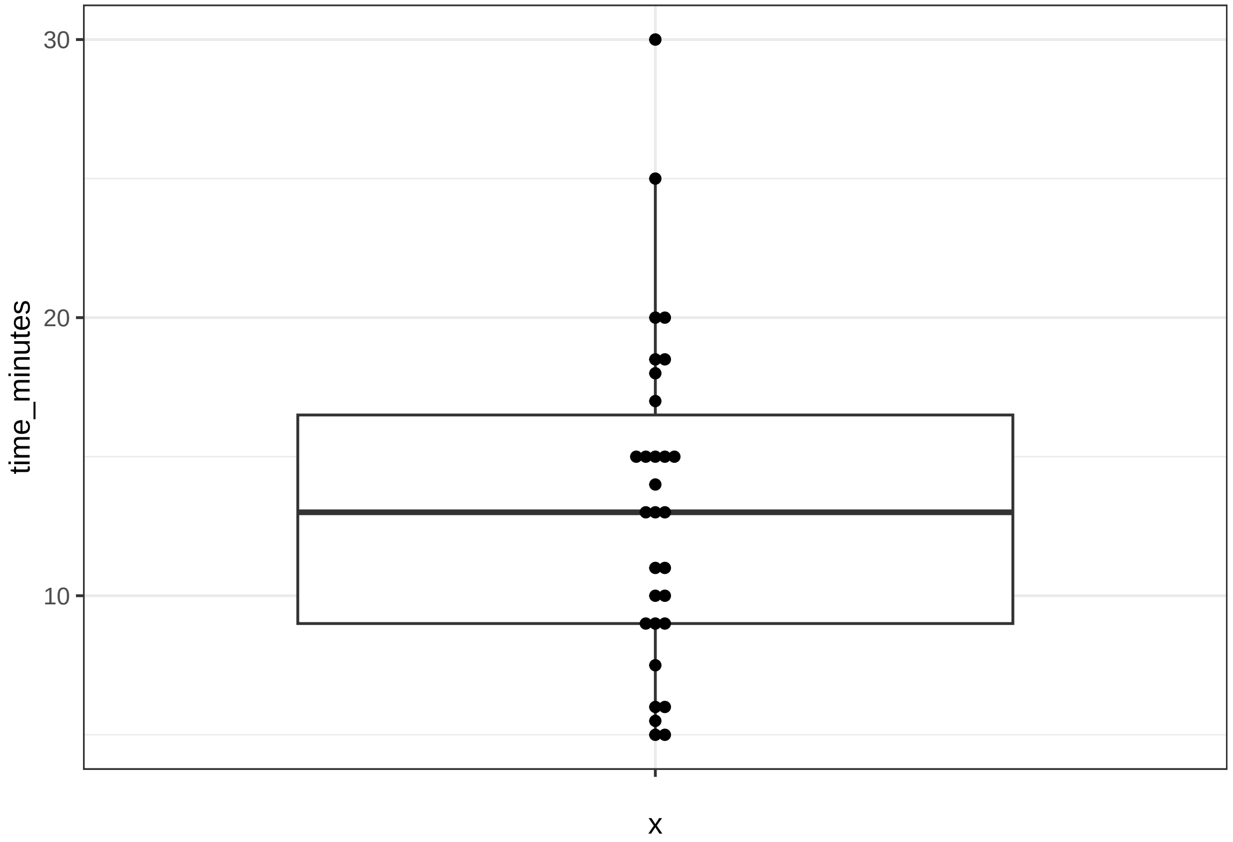

30
20
10
time_minutes
x

10 Session information

```r
sessionInfo()
```

```
R version 4.2.2 (2022-10-31 ucrt)
Platform: x86_64-w64-mingw32/x64 (64-bit)
Running under: Windows 10 x64 (build 19045)

Matrix products: default

locale:
[1] LC_COLLATE=German_Switzerland.utf8  LC_CTYPE=German_Switzerland.utf8
[3] LC_MONETARY=German_Switzerland.utf8 LC_NUMERIC=C
[5] LC_TIME=German_Switzerland.utf8

attached base packages:
[1] stats     graphics  grDevices utils     datasets  methods   base

other attached packages:
 [1] openxlsx_4.2.5.1 tidyr_1.2.1      forcats_0.5.2    purrr_0.3.5
 [5] epiR_2.0.53      survival_3.4-0   ggplot2_3.4.0    kableExtra_1.3.4
 [9] dplyr_1.0.10     checkpoint_1.0.2 knitr_1.40

loaded via a namespace (and not attached):
 [1] Rcpp_1.0.9         svglite_2.1.0      lubridate_1.9.0    lattice_0.20-45
 [5] class_7.3-20       zoo_1.8-11         assertthat_0.2.1   digest_0.6.30
 [9] utf8_1.2.2         R6_2.5.1           evaluate_0.18      e1071_1.7-12
[13] httr_1.4.4         pillar_1.8.1       gdtools_0.2.4      rlang_1.0.6
[17] uuid_1.1-0         rstudioapi_0.14    data.table_1.14.4  Matrix_1.5-1
[21] flextable_0.8.3    rmarkdown_2.18     labeling_0.4.2     splines_4.2.2
[25] webshot_0.5.4      stringr_1.4.1      pander_0.6.5       munsell_0.5.0
[29] proxy_0.4-27       vipor_0.4.5        compiler_4.2.2     xfun_0.34
[33] pkgconfig_2.0.3    systemfonts_1.0.4  base64enc_0.1-3    ggbeeswarm_0.6.0
[37] htmltools_0.5.3    tidyselect_1.2.0   tibble_3.1.8       fansi_1.0.3
[41] viridisLite_0.4.1  withr_2.5.0        sf_1.0-9           grid_4.2.2
[45] gtable_0.3.1       lifecycle_1.0.3    DBI_1.1.3          magrittr_2.0.3
[49] BiasedUrn_2.0.8    units_0.8-0        scales_1.2.1       KernSmooth_2.23-20
[53] zip_2.2.2          cli_3.4.1          stringi_1.7.8      farver_2.1.1
[57] xml2_1.3.3         ellipsis_0.3.2     generics_0.1.3     vctrs_0.5.0
[61] tools_4.2.2        beeswarm_0.4.0     glue_1.6.2         officer_0.4.4
[65] fastmap_1.1.0      yaml_2.3.6         timechange_0.1.1   colorspace_2.0-3
[69] classInt_0.4-8     rvest_1.0.3
```
