# Supplementary material for: Optical recognition of the eggs of four Aedine mosquito species (Aedes albopictus, Aedes geniculatus, Aedes japonicus, and Aedes koreicus)
Source: PLoS One. 2023 Nov 1;18(11):e0293568. doi: 10.1371/journal.pone.0293568 (PMC10619821; doi:10.1371/journal.pone.0293568)
Supplement: S3 Text — https://doi.org/10.6084/m9.figshare.24207933.v2. (PDF) [file pone.0293568.s017.pdf]

S1 Text. Definitions and short explanations of S1 and S2 Datasets

record: single observation.

report name: name of the questionnaire.

question: number of questions, from 1 to 24, per questionnaire.

answer.rater: name of the species given by the participant.

picture_ID: short code for the egg in the image.

picture file name: full name of the egg.

canton: place of origin of the egg.

egg name: complete name of the egg.

optical determination by the operator: species determination by the laboratory operator for the validation.

chorion quality: quality of the exochorion (high, medium and low).

file MALDI-TOF: name of the MALDI-TOF file.

MALDI-TOF determination: species determination with a MALDI-TOF mass spectrometer from Mabritec AG.

%: percentage of determination accuracy of the MALDI-TOF mass spectrometer; compare means that the spectrum obtained is compared with a database of spectra.

obs - light green star: if there is more than one egg per image, the egg to be considered is marked with a green star.

observer: level of entomological knowledge of the participant (expert and non-expert).

time_minutes: time spent filling in the questionnaire and expressed in decimals.

comment: comments from participants.
